# Hotspot in ferruginous rock may have serious implications in Brazilian conservation policy

**DOI:** 10.1038/s41598-022-18798-1

**Published:** 2022-09-01

**Authors:** Douglas Zeppelini, João Victor L. C. Oliveira, Estevam C. Araujo de Lima, Roniere A. Brito, Aila S. Ferreira, Luis C. Stievano, Nathan P. Brito, Misael A. Oliveira-Neto, Bruna C. H. Lopes

**Affiliations:** 1 Laboratório de Sistemática de Collembola e Conservação – Coleção de Referência de Fauna de Solo – CCBSA – Universidade Estadual da Paraíba, Campus V, João Pessoa, PB 58070-450 Brazil; 2 Programa de Pós-graduação em Ciências Biológicas – Zoologia, Universidade Federal da Paraíba, João Pessoa, PB Brazil

**Keywords:** Conservation biology, Entomology

## Abstract

A hotspot of subterranean Collembola in ferruginous rock caves and Mesovoid Shallow Substratum is revealed by the analysis of pseudocryptic diversity. The diversity is accessed by detailed description of chaetotaxy and slight variation in morphology of 11 new species of *Trogolaphysa* Mills, 1938 (Collembola, Paronellidae, Paronellinae) and the 50 previously recorded species of springtails from caves, using optical and electronic microscopy. When combined with recent subterranean surveys, our results show an important reservoir of cave diversity in the Mesovoid Shallow Substratum. Contrastingly the conservation policy for subterranean fauna in metallogenic areas in Brazil prioritizes the caves instead the cave species, which may be extremely detrimental to the fauna in the shallow subterranean habitats not accessible to humans.

## Introduction

Some areas are subject of intense fauna diversification, the term “hotspot” is used to indicate relatively small areas with high and exclusive diversity, though there are different interpretations about what is the threshold which defines such an area. Hotspots may be defined by combining the richness, endemism, extension, and threats to the area in focus^1^, however some approaches to subterranean fauna use an arbitrary cutoff of 20 restricted endemic species with no regards to environmental threats^2^. More recently two South American subterranean hotspots were defined based in the richness of restricted endemic fauna, and fully addressed the conservation aspects of the surroundings of the cave systems^3^.

The species diversity of cave restricted fauna, with limited subterranean distribution and some degree of troglomorphism, known as troglobites^4^, is positively correlated to the extension of the cave and the presence of perennial pools, and sometimes negatively correlated to the presence of streams, which can cause disturbance in the habitats and import a more diverse troglophile fauna^5^. Therefore, the more diverse troglobitic fauna is supposed to be found in larger caves, which are often formed in limestone rock, this is corroborated by the cave fauna hotspots found in limestone cave systems in Southeastern and Northeastern Brazil^3,6^.

However, there are different shallow subterranean habitats (SSH), which are spaces that extend through and across the soil and weathered rock matrix^7^. The mesovoid shallow substratum (MSS)^7–9^ seems to be the SSH that best fits the characteristics observed in ferruginous subterranean habitats, even though it differs from the exact original definition for MSS^8^. These underground spaces connect, and somehow extends the cave habitat far beyond the human reach and might as well be a climate refuge to epigeic fauna^10,11^.

The Brazilian States of Minas Gerais (Southeast) and Para (North) represent the more important metallogenic areas in the country and concentrate the mining activities and commodities production. The iron ore lithology presents a profusion of small and shallow caves, subterranean spaces and crevices that functions as MSS^12^, providing habitat for a variety of species, including troglobites^13,14^. Previous studies found higher average relative richness, and distinctiveness in ferruginous rock^12^, than in other lithologies.

Unpublished data from caves and MSS in ferruginous rock, brings 87 morphotypes of Collembola with some degree of troglomorphism, most of them potential new species, from Minas Gerais (73) and Para (14). A total of 38 species have been described so far (32 from Minas Gerai*s*, six from Para), including 22 troglobites (Table 1). Recent studies in Brazil have surveyed hundreds of cave species, from sponges to vertebrates, more than 250 already described^3,14–17^, great part of it focused on large caves in karstic lithology^6,18,19^.

**Table 1:** Collembola species described from caves and MSS in Brazil.

Species	Ecological status*	Lithology	State
*Acherontides eleonorea* Palacios-Vargas & Gnaspini-Netto, 1992	Troglobite	Li^1^	SP
*Acherontides serrasapoensis* Lima, Stievano & Zeppelini, 2019	Troglophile	Ir^1,2,3^	MG
*Arrhopalites mendoncae* Brito, Lima & Zeppelini, 2019	Troglobite	Ir, Li^1^	MG
*Arrhopalites alambariensis* Zeppelini, 2006	Troglobite	Li^1^	SP
*Arrhopalites amorimi* Palacios-Vargas & Zeppelini, 1995a	Troglobite	Li^1^	SP
*Arrhopalites botuveraensis* Zeppelini, 2006	Troglobite	Li^1^	SC
*Arrhopalites glabrofasciatus* Zeppelini, Brito & Lima, 2018	Troglobite	Ir, Li^1^	MG
*Arrhopalites gnaspinii* Palacios-Vargas & Zeppelini, 1995a	Troglobite	Li^1^	SP
*Arrhopalites heteroculatus* Zeppelini, 2006	Troglobite	Li^1^	SP
*Arrhopalites lawrencei* Palacios-Vargas & Zeppelini, 1995a	Troglobite	Li^1^	SP
*Arrhopalites paranaenses* Zeppelini, 2006	Troglobite	Li^1^	PR
*Coecobrya phoenix* Brito, Lima & Zeppelini, 2019	Troglobite	Ir, Li^1^	MG
*Cyphoderus caetetus* Zeppelini & Oliveira, 2016	Troglophile	Ir^1,2,3^	MG
*Cyphoderus mucrominimus* Oliveira, Alves & Zeppelini, 2017	Troglophile	Ir^1^	PA
*Cyphoderus mucrostrimenus* Oliveira, Alves & Zeppelini, 2017	Troglophile	Ir^1,2,3^	PA
*Cyphoderus palaciosi* Oliveira, Brito & Zeppelini, 2021	Troglophile	Ir^1,2,3^	MG
*Cyphoderus pataxo* Oliveira, Brito & Zeppelini, 2021	Troglophile	Ir^1,2,3^	MG
*Pararrhopalites papaveroi* (Zeppelini & Palacios-Vargas, 1999)	Troglobite	Li^1^	MS
*Pararrhopalites queirozi* Brito, Lima & Zeppelini, 2019	Troglobite	Li^1,2^	MG
*Pararrhopalites sideroicus* Zeppelini & Brito, 2014	Troglobite	Ir^1,2^	MG
*Pararrhopalites ubiquum* Zeppelini, Lima & Brito, 2018	Troglobite	Ir^1^	MG
*Pararrhopalites wallacei* (Palacios-Vargas & Zeppelini, 1995a)	Troglobite	Li^1^	SP
*Pseudosinella acantholabrata* Cipola, 2020	Troglophile	Ir^1,3^	MG
*Pseudosinella alfanjeunguiculata* Bellini, Cipola & Souza, 2020	Troglobite	Ir^1^	MG
*Pseudosinella ambigua* Zeppelini, Brito & Lima, 2018	Troglobite	Li^1^	MG
*Pseudosinella aphelabiata* Bellini, Cipola & Souza, 2020	Troglobite	Ir^1^	MG
*Pseudosinella brumadinhoensis* Cipola, 2020	Troglobite	Ir^2^	MG
*Pseudosinella chimerambigua* Oliveira, Lima & Cipola, 2020	Troglobite	Ir^1,2^	MG
*Pseudosinella diamantinensis* Bellini, Cipola & Souza, 2020	Troglobite	Ir^1^	MG
*Pseudosinella guanhaensis* Zeppelini, Brito & Lima, 2018	Troglobite	Gr^1^	MG
*Pseudosinella keni* Cipola, 2020	Troglobite	Ir^1^	MG
*Pseudosinella labiociliata* Cipola, 2020	Troglobite	Ir^1^	MG
*Pseudosinella labruspinata* Cipola, 2020	Troglobite	Ir^1,2^	MG
*Pseudosinella macrolignicephala* Oliveira, Lima & Cipola, 2020	Troglophile	Ir^1,2,3^	MG
*Pseudosinella marianensis* Bellini, Cipola & Souza, 2020	Troglophile	Ir^1,3^	MG
*Pseudosinella mitodentunguilata* Bellini, Cipola & Souza, 2020	Troglobite	Ir^1^	MG
*Pseudosinella neriae* Bellini, Cipola & Souza, 2020	Troglobite	Ir^1^	MG
*Pseudosinella paraensis* Cipola, 2020	Troglobite	Ir^1^	PA
*Pseudosinella parambigua* Oliveira, Lima & Cipola, 2020	Troglophile	Ir^1,2,3^	MG
*Pseudosinella phyllunguiculata* Oliveira, Lima & Cipola, 2020	Troglobite	Ir^1^	MG
*Pseudosinella prelabruscervata* Oliveira, Lima & Cipola, 2020	Troglobite	Ir^1^	MG
*Pseudosinella pusilla* Oliveira, Brito & Cipola, 2020	Troglobite	Ir^1^	PA
*P*s*eudosinella serpentinensis* Cipola, 2020	Troglobite	Ir^1^	MG
*Pseudosinella spurimarianensis* Bellini, Cipola & Souza, 2020	Troglophile	Ir^1,2,3^	MG
*Pseudosinella taurina* Cipola, 2020	Troglobite	Ir^1^	PA
*Pseudosinella unimacrochaetosa* Cipola, 2020	Troglophile	Ir^1,3^	MG
*Troglobius brasiliensis* Palacios-Vargas & Zeppelini, 1995a	Troglobite	Sn^1^	PA
*Troglobius ferroicus* Zeppelini, Silva & Palacios-Vargas, 2014	Troglobite	Ir^1^	MG
*Trogolaphysa aelleni* Yosii, 1988	Troglobite	Li^1^	SP
*Trogolaphysa barroca* **sp. nov**	Troglobite	Ir^1^	MG
*Trogolaphysa bellinii* **sp. nov**	Troglobite	Ir^1^	MG
*Trogolaphysa chapelensis* **sp. nov**	Troglobite	Ir^1^	MG
*Trogolaphysa crystallensis* **sp. nov**	Troglobite	Ir^1^	MG
*Trogolaphysa dandarae* **sp. nov**	Troglobite	Ir^1^	PA
*Trogolaphysa epitychia* **sp. nov**	Troglobite	Ir^1^	MG
*Trogolaphysa gisbertae* **sp. nov**	Troglobite	Ir^1^	PA
*Trogolaphysa hauseri* Yosii, 1988	Troglobite	Li^1^	SP
*Trogolaphysa lacerta* **sp. nov**	Troglobite	Ir^1^	MG
*Trogolaphysa mariecurieae* **sp. nov**	Troglophile	Ir^1,2,3^	MG
*Trogolaphysa sotoadamesi* **sp. nov**	Troglobite	Ir^1^	MG
*Trogolaphysa zampauloi* **sp. nov**	Troglobite	Li^1^	SP

Distributional States—Mato Grosso do Sul, MS. Minas Gerais, MG. Para, PA. Parana, PR. Santa Catarina, SC. São Paulo, SP. Lithology—Granitic rock, Gr. Iron rock, Ir. Limestone rock, Li. Sandstone, Sn. Cave^1^, MSS^2^, Surface^3^. *We consider troglobite all species with some degree of troglomorphism and known distribution restricted to subterranean habitats (for a discussion on troglobite definition see Sket^4^.

Here we present a group of 50 known species of Collembola found in Brazilian caves in different lithologies and add 11 new species of the genus *Trogolaphysa* Mills, 1938 with some degree of troglomorphism, from caves and MSS in ferruginous and limestone rock.

The genus *Trogolaphysa* has 69 described species worldwide, only eight have been recorded from Brazil so far: *T. aelleni* Yoshii, 1988; *T. ernesti* Cipola & Bellini, 2017; *T. formosensis* Silva & Bellini, 2015; *T. hauseri* Yoshii, 1988; *T. hirtipes* (Handschin, 1924), *T. millsi* Arlé, 1939; *T. piracurucaensis* Nunes & Bellini, 2018; and *T. tijucana* (Arlé & Guimarães, 1979). Our results depict an important hotspot for cave Collembola in the State of Minas Gerais, also corroborate the expected high species richness in ferruginous rock caves and MSS and shed some light to the impact of the MSS in the conservation policy as a refuge for subterranean diversity.

## Results

This study presents 11 new species of cave *Trogolaphysa* (Table 1), two new species from Para, eight from Minas Gerais from caves in metallogenic rock, and one new species from Sao Paulo, found in a limestone cave. Species were collected directly from organic debris in caves, the MSS was accessed through samplings in drilling holes. These results represent an increase of 25% in the previous 32 species of cave Collembola described for the State of Minas Gerais. Data from Para are still scarce, with only five previous records from iron rock caves and one from sandstone cave (Table 1). The species from Sao Paulo is from a different lithology, a much larger cave with narrower connections to the MSS. It represents a new record to add to the 17 known cave springtails from limestone caves in Brazil (Table 1).

There are 50 valid species of cave Collembola previously recorded from Brazil, 38 troglobites and 12 troglophiles. For limestone caves, there are 17 species from five different States at South and Southeastern Regions, all troglobites. There is a troglobitic species from sandstone cave in the State of Para at Northern Region, and one troglobite from granitic caves in Minas Gerais. All the 34 remaining records are from ferruginous rock caves and MSS, 29 from the Southeastern State of Minas Gerais (19 troglobites and 10 troglophiles), and five from Para (three troglobites and two troglophiles).

With the results presented here the total number of cave Collembola recorded for Brazil rises to 61 species, with 18 species from limestone caves, one species from sandstone cave, one from granitic cave, and iron caves and MSS with 44 known subterranean species. The State of Minas Gerais present the highest richness for cave Collembola in Brazil, with five species from limestone and one from granitic caves, and 37 records of species endemic to ferruginous rock shallow caves and MSS in Minas Gerais, this has important implications for the conservation areas policy in Brazil, which may apply to other ferruginous rock subterranean environments in tropical areas in the world.

## Discussion

### Ferruginous mesovoid shallow substratum

The iron ore deposits in Brazil present a semi continuous covering layer of fragmented hematite and lesser components cemented by limonite, called *Canga*. It is formed by weathering and lixiviation, and produce a labyrinthic complex of subterranean spaces, crevices, and tiny underground connections, depicting a habitat that is analogous to the MSS^12^.

In temperate zones the MSS plays a role as refuge for arthropod fauna, mainly at high altitudes where the cold weather can eliminate all the ectothermic fauna from the surface^10,11^. Similarly, seasonal migration movements are observed in the MSS for different taxa as response to hot dry summer^20,21^. When troglobitic fauna is concerned the MSS has a different role, cave restricted Collembola showed higher underground dispersal capacity than troglophiles^22^, therefore, the MSS can connect neighboring caves systems and extend their distribution range.

In Brazilian metallogenic rock, cave species richness is higher than in any other lithology^12^. The cave Collembola found in Brazil corroborates this assumption, from the total of 61 known cave species, one troglobite was recorded from sandstone caves and one from granitic cave, 18 species were recorded from limestone caves (all troglobites), and 44 species from iron caves and MSS (31 troglobites). Three troglobitic species were recorded from both limestone and iron caves, in both cases the caves are separated by large distances and the lithologies are disjunct. This incongruent and disjunct distribution is an indication of potentially unrecognized cryptic or overlooked species.

This is more relevant when considered that ferruginous rock represents only 0.15% of the Brazilian territory (carbonatic rock 3.1%), nearly 10,000 km^2^ (carbonatic rock 260.800 km^2^, Brazilian territory 8.516.000km^2^)^23^, and that most of the biospeleological research is focused on large caves, usually in limestone^6,18,19^. The high richness of species restricted to small shallow caves, indicates that MSS plays a role as an extension of the cave environment.

The State of Minas Gerais is the most diverse with 40 species of cave Collembola, the complex mosaicist lithology and the ecotone Cerrado Forest-Atlantic Forest are the main barriers associated to the richness of species restricted to caves and MSS. In this State, the iron rock subterranean habitats host 29 troglobites and provide habitat and refuge to 11 known troglophiles species.

**Figure 1:**
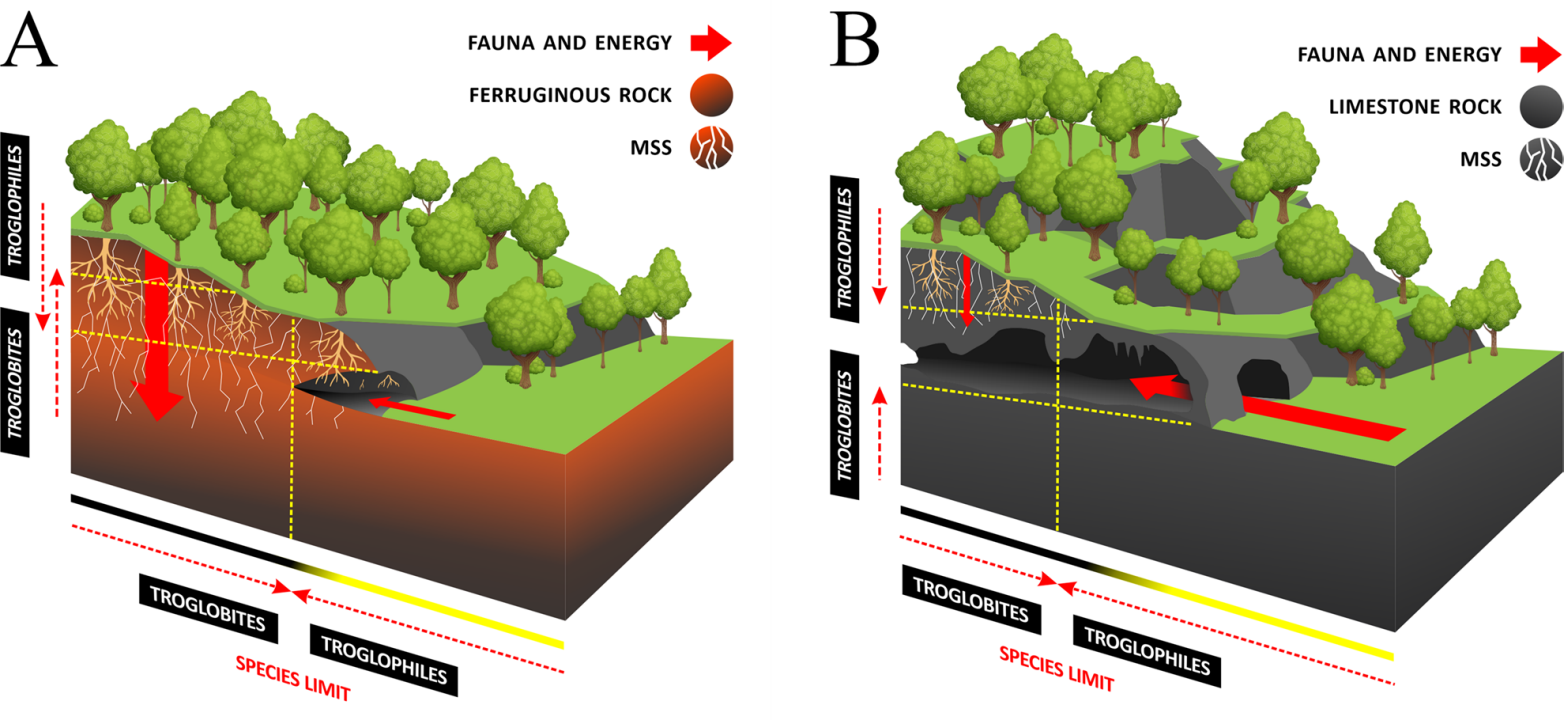
Schematic profile of ferruginous rock cave and limestone rock cave. (**A**) Ferruginous rock—small and shallow caves, abundant roots, reticulated MSS; fauna and energy come mostly from the above ground (solid red arrows), troglobites inhabit the MSS and reach the deep limits of the cave horizontally, and lower limits of the soil vertically; troglophiles inhabit the surrounding and the cave, eventually reaching shortly in the MSS horizontally, but overlapping the troglobitic limits in the MSS and lower limits of soil vertically (dotted red arrows). (**B**) Limestone rock—large caves, usually not reached by roots, sparse or absent MSS; fauna and energy come largely through the cave entrances (solid red arrows), troglobites inhabit the deep aphotic zone reaching the aphotic intermediary zone horizontally, not reaching the upper MSS and epikarst vertically; troglophiles inhabit the surroundings and the cave, eventually reaching the deep aphotic zone horizontally, sometimes restricted to the MSS and epikarst vertically (dotted red arrows). Yellow to black bar represents the light reach.

For caves in non-ferruginous lithologies, the size and number of entrances influence the species richness by giving the surface fauna access to the subterranean environment, and as a sink for organic matter input^5^. Contrastingly the caves in iron rock are small and shallow, often with few meters of horizontal development, the connections to the MSS are conspicuous and abundant, providing a rather continuous subterranean habitat. In this context, instead, the distribution of the troglobitic species suggests that the entrances of iron rock caves are the limits of the available subterranean habitat for troglobites inside-out, and of suitable habitats for troglophiles outside-in (Fig. 1). We can consider the entrances of these caves as windows of the MSS, the spatial limit of the subterranean environment which presents the minimum conditions to the survival of a troglobite, while partially inhibits the dispersion of troglophiles deeper in the MSS. Troglobites can disperse underground more efficiently than troglophiles, however troglophiles are more efficient than troglobites to disperse through the surface^22^. In ferruginous lithology the size of the cave and its entrance influences the species richness^5^, mainly because large iron caves can greatly affect the capacity of collectors and biologists to access the troglobitic fauna in the MSS, as the number of accessible connections to the MSS increases exponentially with the length of the cave in iron rock^12^.

Another contrast of ferruginous rock caves is that the biotrophic flow seems to be inverted (Fig. 1), in limestone caves the energy and fauna come from outside mostly through the cave entrance, and the fauna eventually speciate to become troglobitic, possibly restricted to the depths of a single cave. Despite the demonstrated existence of an epikarst, the particularities of the weathering process, the water percolation^24^, and different subterranean habitats as scree slopes and MSS^7,11^, limestone caves tend to be large and grow deep through dissolution of the rock by water during the genesis of the cave. The deeper the cave is, the lesser the permeability of the rock, the epikarst usually reaches about 15 m deep^24^.

In iron caves the fauna comes from the above ground through the MSS connections between surface and subterranean environments, the same happens with energy that comes with roots that reach the MSS abundantly^12^. The troglobites develop in the MSS and eventually reach the caves where it can be seen in its distribution limits, and the troglophiles go in the opposite direction, inhabiting the surface and going inside the caves to refuge from climate, but not going too far in the MSS (see Table 1, species marked with^1,3^).

### Pseudocryptic diversity

Large caves with deep aphotic zones, stable abiotic conditions, water pools, often hosting bat colonies, are correlated to high number of restricted species^12^, usually displaying classic troglomorphism as absence of eyes and body pigments, elongated appendages, increased body size^25^. In the ferruginous rock MSS the same troglomorphisms are present in most species, even though, we observed that some Entomobryoid Collembola are often reduced in size, with normal or shortened (even though always functional) appendages, similar to that of euedaphic fauna.

Cryptic species recognized from a single widespread species complex through barcode sequencing, revealed related morphological differences corresponding to the species separation^26^. To access this information it is necessary to expand the morphological refinement, some cryptic species are grouped together as result of limited selection of diagnostic characters. This is by definition pseudocryptic species, when “individuals can be identified from morphology providing sufficient care is taken, but are so similar that there is a high probability of misidentification, even by a competent scientist”^27^.

The species found in ferruginous caves and MSS are very similar in most of its macro morphology, differences are subtle, species recognition often must rely on minor details of chaetotaxy (Fig. 3, 4, 5, 6, 7, 8, 9, 10, 11, 12, 13, 14, 15, 16, 17, 18, 19, 20, 21, 22, 23, 24, 25, 26, 27, 28, 29, 30, 31, 32, 33, 34, 35, 36, 37, 38, 39, 40, 41, 42, 43, 44) and slight variations of morphological structures, often overlooked, as observed for the genera *Arrhopalites*, *Pararrhopalites*, *Pseudosinella* and *Trogolaphysa*. Such pseudocryptic diversity can only be accessed by specialized morphological scrutiny, molecular sequencing or a combination of both.

Whether we accept that cryptic diversity in Collembola cannot be explained by accelerated rates of molecular evolution^28^, it is likely that the diversity of subterranean Collembola in ferruginous MSS and caves, results of the combination of the effects of lithology arrangement, phytophysiognomy and climate fluctuation at local scale.

Finally, the recognition of cryptic or pseudocryptic species within presumed widespread allopatric species is crucial to efficiently develop management and conservation plans^22^ and reduce the underestimation of cave Collembola diversity.

### Subterranean collembola hotspot

A total of 61 species of cave Collembola were recorded in Brazil so far, 40 are records from the State of Minas Gerais, including 29 troglobites (Table 1), 26 of them are from iron caves and MSS as well as 11 troglophiles. For comparison we can consider two important subterranean hotspots in Limestone caves in the States of Sao Paulo and Bahia (Southeastern and Northeastern regions), which presented an overall species richness of 28 and 22 troglobites, respectively. These two caves are under different impact pressures, the former is in a protected area with controlled access, and the latter is under intense touristic exploitation^3^.

Myers et al.^1^ combined richness, endemism, distribution spam and threats to the area to define places of priority for conservation, called hotspots. The number of troglobites, with a full consideration of the threats or conservation conditions of the caves and surroundings was, also, recently used as criteria for defining hotspot^3,6^.

The ferruginous rock outcrops in Brazil are under a intense economic pressure, the mining industry represents an important part of the production of commodities as iron ore and steel. The high diversity and endemism of cave Collembola found in recent studies (Table 1), affecting directly the beta diversity of the areas considering the species are found nowhere else, and the continuous threat to the subterranean habitats formed in ferruginous rock, justify categorizing the ferruginous subterranean habitats as hotspot for cave Collembola in the State of Minas Gerais. It is important to remark that the diversity considered here is only for Collembola species, and that the studies mentioned above have a much higher phylogenetic diversity.

### Conservation policy implications

The ferruginous caves and the MSS represent sites of intense overlooked pseudocryptic diversification. Katz et al.^22^ observed that for Collembola in limestone areas the detection of short-range endemics, genetic isolation, and apparent cryptic diversity has major conservation implications.

The results we present here bring several considerations on conservation strategies and policies. The high diversity and endemism rate observed for cave Collembola, associated to threats to the subterranean environments as mining, deforestation, and urbanization flag these areas as maximum priority and interest for planning putative conservation areas^29^. These areas demand a multi-factor approach to successfully develop policies which optimize the diversity conservation, particularly subterranean diversity.

**Figure 2:**
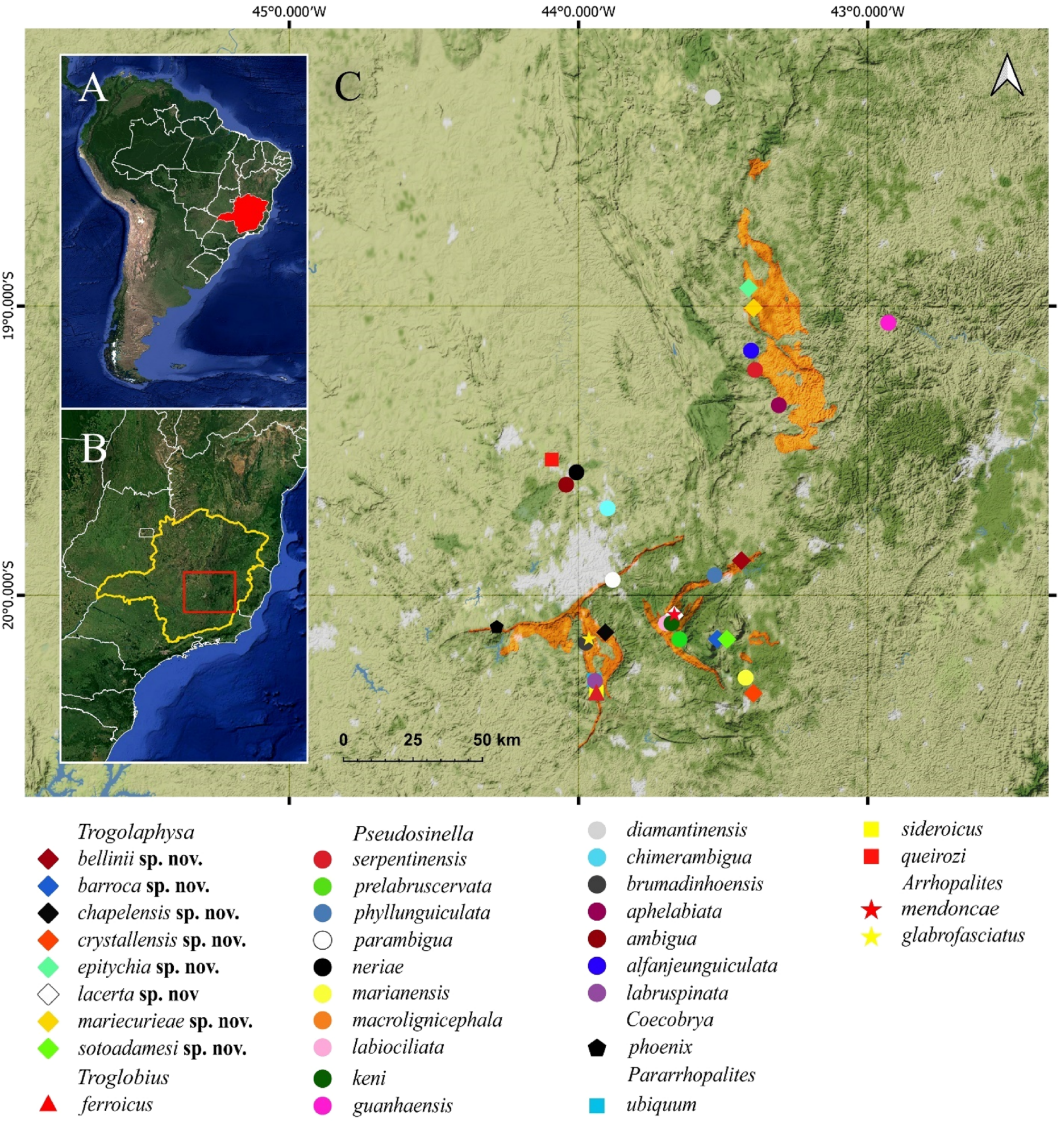
Subterranean species distributed in ferruginous rock in the state of Minas Gerais, Brazil. (**A**) South America with Brazilian borders and the state of Minas Gerais marked in solid red. (**B**) Minas Gerais state borders, red box in detail. (**C**) detail of the species distributed in ferruginous rock in the principal mining areas in the state of Minas Gerais. Ferruginous rock areas marked in bright orange. Urban and metropolitan areas marked in bright gray (note the Belo Horizonte metropolitan area, the state capital, just next to the minerary sites). Created using QGIS [Software GIS] version 3.16. QGIS Geographic Information System. Open Source Geospatial Foundation Project. http://qgis.osgeo.org, 2021.

Brazilian legislation has protective measures for caves, but allows the complete suppression of a cave for mining or other exploratory purpose, under a process for licensing the proposed activities. Even though some criteria are imposed, it fails in considering some important aspects of the cave structure in different lithology^12^. Under this perspective the whole extension of ferruginous (and carbonatic) rock deposits in Brazil are available for exploitation, with irreversible impact on the subterranean fauna. There is over than 9400 companies in activity in the country, producing about 235.000.000 ton/year of iron ore, the second biggest production in the world. More than 72% of the Brazilian iron ore reservoirs is located in the state of Minas Gerais, the locality of occurrence of 37 out of the 44 known species of Collembola found in ferruginous subterranean habitats in Brazil (Fig. 2).

Here we observed that the whole process needs a revision when comes to ferruginous rock, where the cave may not be the important spatial unit to preserve, instead, the high subterranean diversity areas must be surveyed, not only in caves but also in the MSS. It is possible that in some cases to protect a hill that harbors a thick layer of *Canga* with a troglobitic species rich MSS, would result more effective to preserve restricted subterranean fauna, than to protect a small and shallow cave with reduced troglobitic richness.

The state of Minas Gerais has 75 integral conservation units (defined by law), with maximum protection policy, however, these conservation units represent only 1.05% (~ 619,800 ha) of the state territory. There are other categories of conservation units, called of “sustainable use”, with much less restrictive policies. These categories of conservation units are much less effective to preserve epigean species, due to the diverse usages and practices in those areas. Nevertheless, the 19 sustainable use conservation units in the state of Minas Gerais (private conservation units excluded) correspond to 3.01% (~ 1,768,000 ha) of the state territory (http://www.ief.mg.gov.br/unidades-de-conservacao—accessed Sep/31/2021). Sustainable use conservation units have some criteria that prevents highly destructive activities, allowing some extractive crops, subsistence agriculture and tourism. These activities may be compatible with subterranean conservation through the MSS, therefore the conservation unit network can get some advantage trying to connect integral conservation units with sustainable use ones. It was proposed that the sampling for subterranean fauna in prospection drilling holes all over the area may bring important information about species richness and distribution, mainly if combined with cave and surface sampling^30^. This procedure, implemented in the process for licensing new high impact exploratory activities, can improve the conservation effectiveness of the conservation units and compensation areas, precisely define the role of the cave in the conservation plan, and shift the focus towards troglobitic species richness.

## Conclusions

Our results depict the ferruginous subterranean environment as an important hotspot for cave Collembola in the state of Minas Gerais, corroborating the expected high species richness in ferruginous rock caves and MSS. We also demonstrate that access pseudocryptic diversity as observed in the genera *Arrhopalites*, *Pararrhopalites*, *Pseudosinella* and *Trogolahysa* is mandatory for planning the conservation strategies for subterranean Collembola. The distribution of the species through the MSS can be favored by sustainable use conservation units, whether this fauna is surveyed along the licensing process. Finally, we conclude that the conservation planning for future conservation unit establishment must focus not only on caves but also in the MSS, accessing the fauna through sampling in prospection drilling holes. Protecting an area with high richness of endemic troglobites down in the MSS may be more effective than to protect a shallow cave when it comes to preserve troglobitic diversity.

## Methods

### Pseudocryptic diversity

The richness was the measure of the subterranean diversity, we surveyed all data about previous records for Brazilian Collembola cave species, ecological status, lithology, and distribution from the literature, and included 11 newly found pseudocryptic species from subterranean habitats in iron and limestone rock. The pseudocryptic species were verified by comparison of chaetotaxy and “micro-morphology” through optic and scanning microscopy of disjunct populations of a widespread morphotype. The imagery was compared under hypotheses of chaetotaxic and morphologic homology, previously defined by different authors. Those populations with consistent discrete chaetotaxic and morphologic patterns were assumed to be independent species, therefore they were taxonomically diagnosed, named, and ordered in a dichotomic identification key with all Brazilian species of the genus.

### Microscopy

Specimens were preserved in ethanol 70% and mounted on slides following Jordana et al.^31^, after clearing using Nesbitt’s solution for study under phase contrast microscope, line drawings were made with help of a drawing tube. For scanning electronic microscope (SEM) study, specimens were dehydrated by ethanol, dried in a critical point dryer, and covered in gold.

### Homology

The terminology used in the diagnoses for the hypotheses of homology followed: labial chaetotaxy after Gisin^32^ with additions of Zhang and Pan^33^, Fjellberg^34^ for labial palp papillae and maxillary palp; postlabial chaetotaxy after Chen and Christiansen^35^, with adaptations of Cipola et al.^36^ for **J** series; clypeal chaetotaxy after Yoshii and Suhardjono^37^; labral chaetotaxy after Cipola et al.^38^; unguiculus lamellae after Hüther^39^; Anterior dens chaetotaxy after Oliveira et al.^40^; Mari-Mutt^41^ for dorsal head chaetotaxy, with additions of Soto-Adames^42^; Szeptycki^43^ and Zhang and Deharveng^44^ for S-chaetotaxy; and Szeptycki^45^ for dorsal chaetotaxy, with additions and modifications provided by Soto-Adames^42^ and Zhang et al.^46^. Symbols used to depict the chaetotaxy are presented in Fig. 4A–C. Codes will be used in *italics* along the text to replace the morphological description of each chaeta and sensillum type. Additional information about morphology and chaetotaxy of discussed species was obtained from the literature.

### Abbreviations used in the diagnoses

Ant–antennal segment(s); b.c.–basal chaeta(e), t.a.–terminal appendage of the maxillary palp; l.p.–lateral process of labial papilla E, lpc–labial proximal chaeta(e); Th–thoracic segment; Abd–abdominal segment(s); Omt–trochanteral organ; a.e.–antero-external lamella, a.i.–antero-internal lamella, a.t.–unguis apical tooth, b.a.–basal anterior tooth of unguis, b.p.–basal posterior tooth of unguis, m.t.–unguis median tooth, p.i.–postero-internal lamella, p.e.–postero-external lamella; mac–macrochaeta(e), mes–mesochaeta(e), mic–microchaeta(e), ms–specialized microchaeta(e), psp–pseudopore(s), sens–specialized ordinary chaeta(e) (sensillum), MSS–Mesovoid Shallow Substratum.

### Ecological status

To avoid subjectivity and ambiguity to determine the ecological status of the species, we assumed to be a troglobite all the species with some degree of troglomorphism exclusively distributed in the subterranean environment, either caves, MSS, or both. Species distributed in the surface and subterranean habitats were assumed to be troglophiles.

### Identification Key for the known and new species of the genus ***Trogolaphysa*** recorded in Brazil


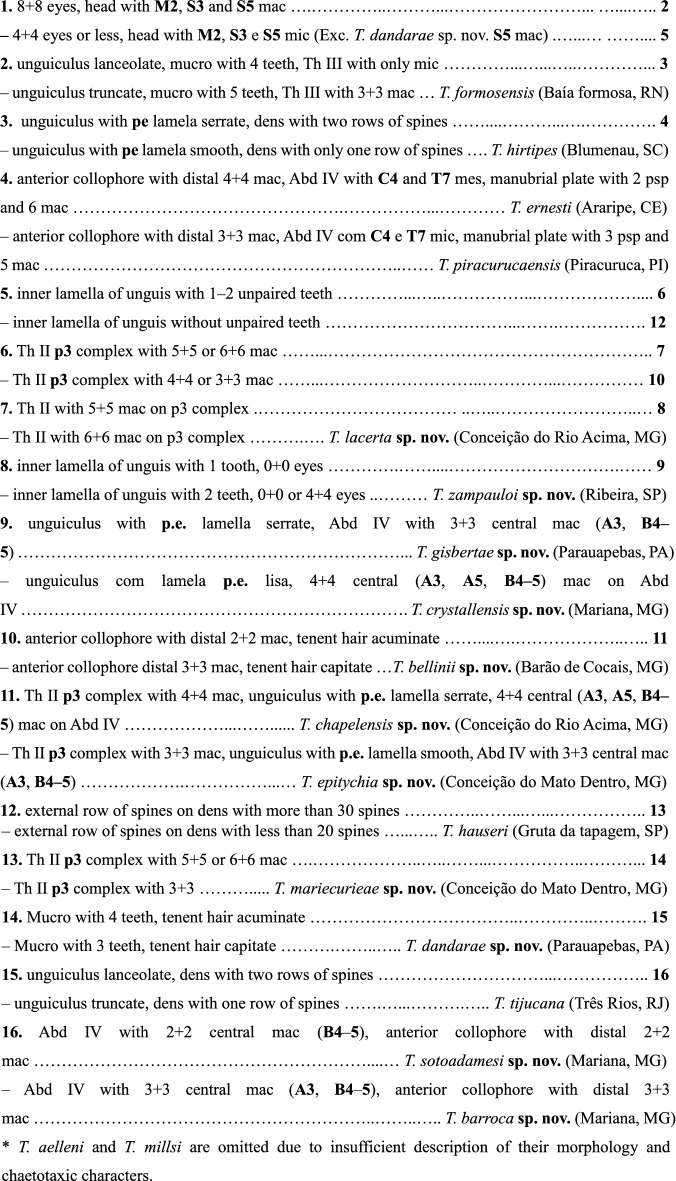


### Taxonomic diagnoses and morphological plates

Type materials are deposited in the *Coleção de Referência de Fauna de Solo*, *Universidade Estadual da Paraíba* (CRFS-UEPB) and *Museu Nacional Rio de Janeiro, Universidade Federal do Rio de Janeiro* (MNRJ-UFRJ).

- https://zoobank.org:pub:2C8F4446-0869-48A9-ABD7-14D4C4DCB0FA
- Additional records in Supplementary Material S1, taxonomic references in S2.
- Family Paronellidae Börner, 1906
- Subfamily Paronellinae Börner, 1906
- Tribe Paronellini sensu Zhang et al*.*, 2019
- Genus *Trogolaphysa* Mills, 1938
- (Figs. 3, 4, 5, 6, 7, 8, 9, 10, 11)

**Figure 3:**
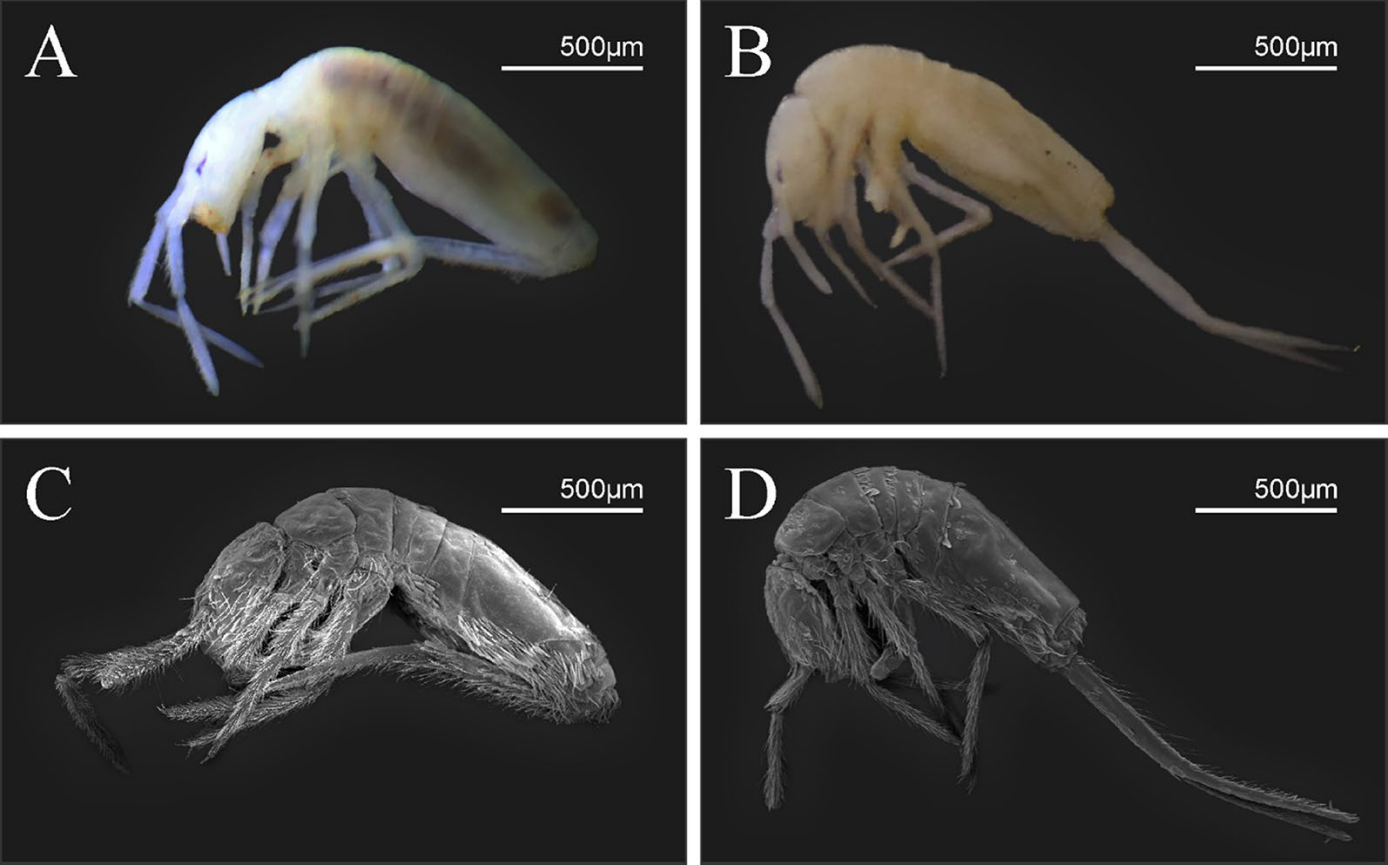
*Trogolaphysa* sp.: habitus lateral view. (**A**, **B**) specimen fixed in ethanol. (**C**, **D**) SEM photographs.

**Figure 4:**
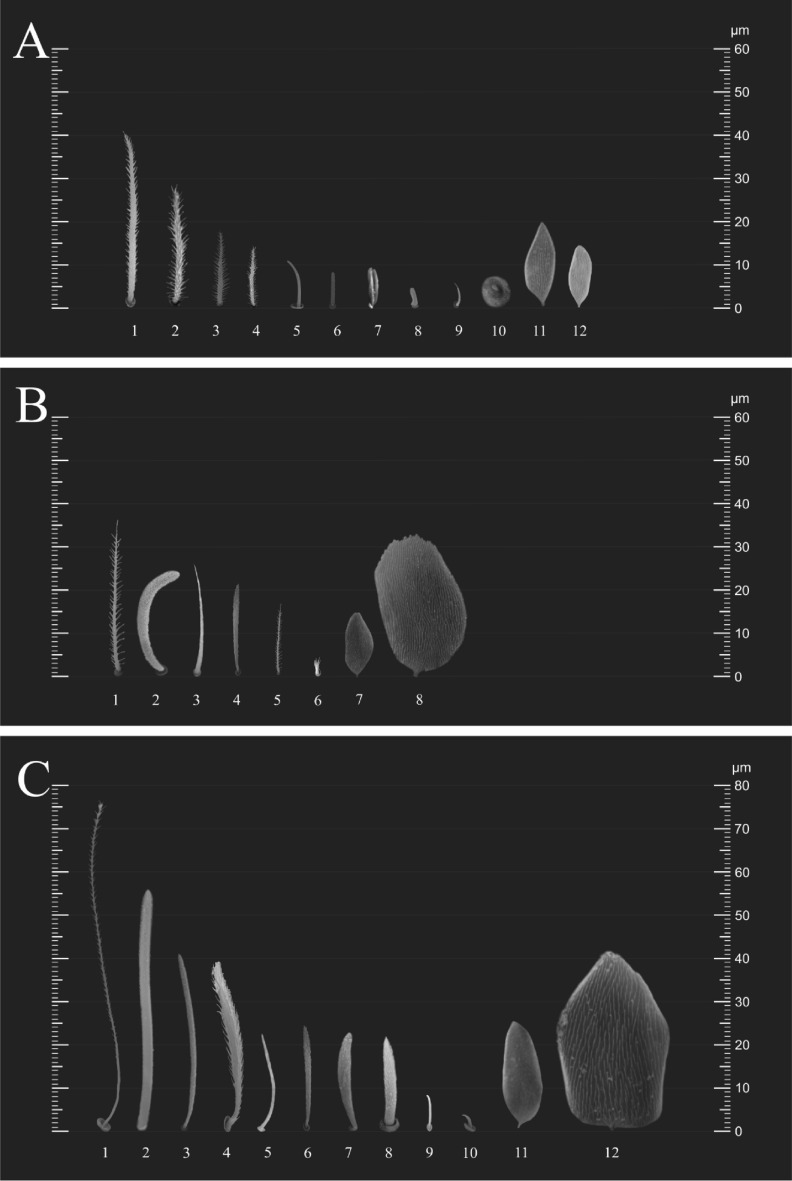
*Trogolaphysa* sp. SEM: general body chaetae. (**A**) Antennal chaetae, sensilla and scales: 1—macrochaeta with short ciliation, 2—macrochaeta with long ciliation, 3—microchaeta with long ciliation, 4—microchaeta with short ciliation, 5—finger-shaped sens, 6—wrinkly sens, 7—coffee bean shaped sens, 8—rod sens, 9—spine-like sens, 10—Ant IV subapical-organ, 11—lanceolate scale, 12—rounded scales. (**B**) Head chaetae and scales: 1—strait macrochaeta with long ciliation, 2—blunt macrochaeta, 3—smooth chaeta, 4—blunt chaeta, 5—strait microchaeta with long ciliation, 6—labial r microchaeta, 7—cephalic anterior scale, 8—cephalic posterior scale. (**C**) Body and appendages chaetae, sens and scales: 1—bothriotrichum, 2—blunt macrochaeta, 3—blunt mesochaeta, 4—dens external ciliate chaeta, 5—smooth microchaeta, 6—blunt microchaeta, 7—fan-shape chaeta, 8—dental spine, 9—‘al’ sens, 10—‘ms’ sens, 11—lanceolate scale, 12—intersegmental scale.

**Figure 5:**
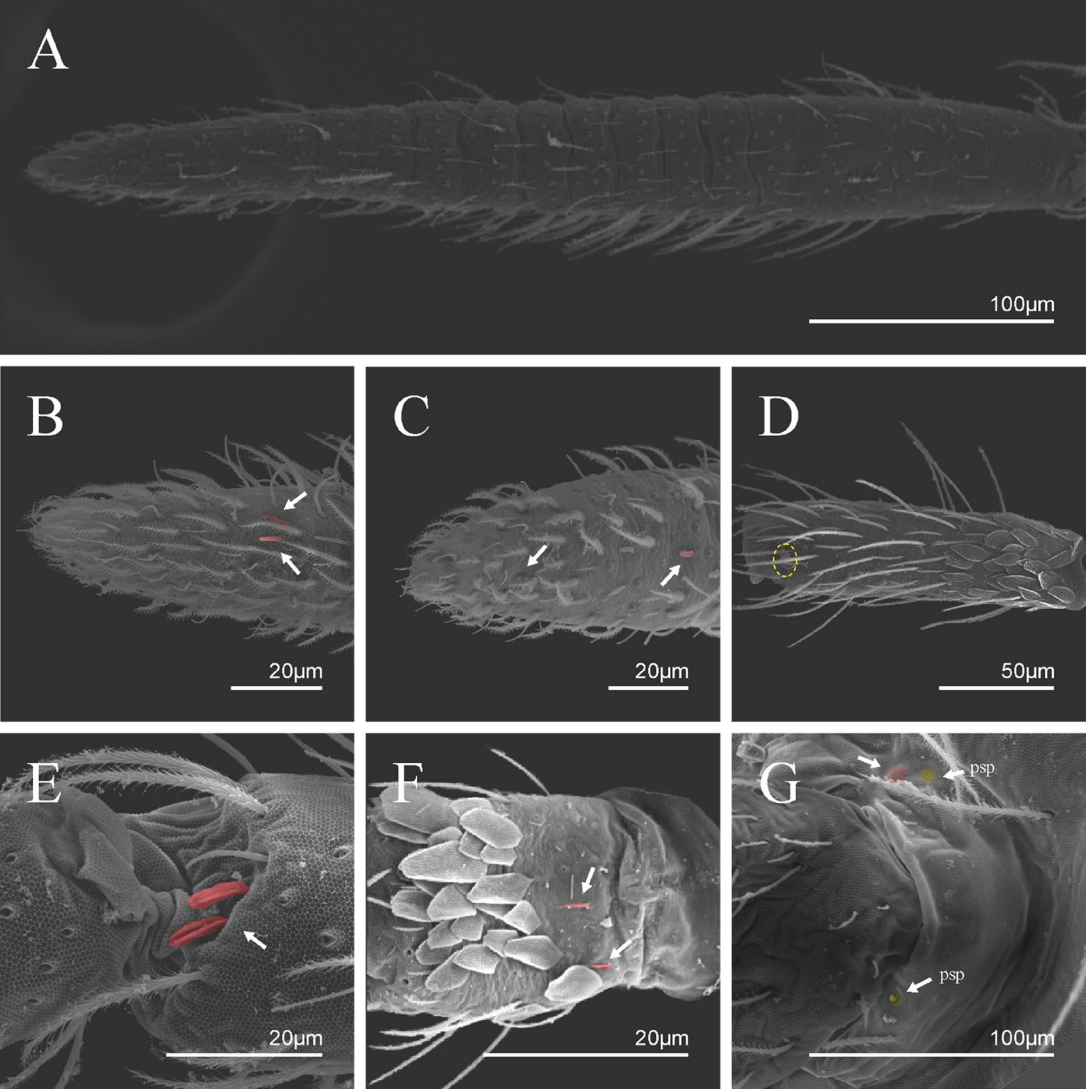
*Trogolaphysa* sp. SEM: antenna: (**A**) Ant IV dorsal view. (**B**) Ant IV apex dorsal view, arrow indicates finger-shaped and wrinkly sens. (**C**) Ant IV apex ventral view, left arrow indicates Ant IV subapical-organ, right arrow point one sensillum type A8. (**D**) Ant II dorsal view, dashed line indicates rod sens. (**E**) Detail of the sensilla of the Ant III apical organ (red). (**F**) Ant I dorsal view spine like sens (arrows indicate the sensilla in red). (**G**) Detail of the Ant I basal, arrow indicates psp and antenobasal organ (yellow and red respectively).

**Figure 6:**
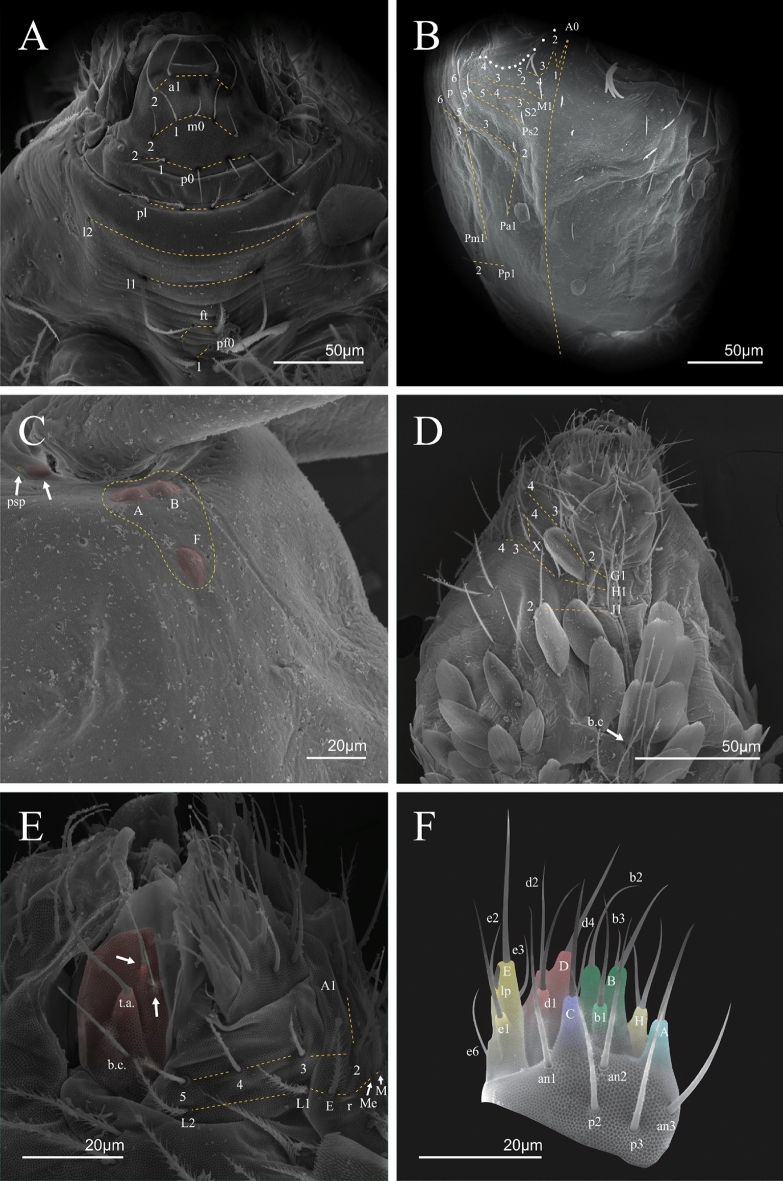
*Trogolaphysa* sp. SEM: head and mouthpart chaetotaxy. (**A**) clypeus, (**B**) dorsal head, (**C**) eyes (red) circled by dashed line, arrow indicates antenobasal organ and psp, (**D)** ventral head, (**E**) maxillary palp and sublobal plate (right side), (**F**) detail of maxillary palp.

**Figure 7:**
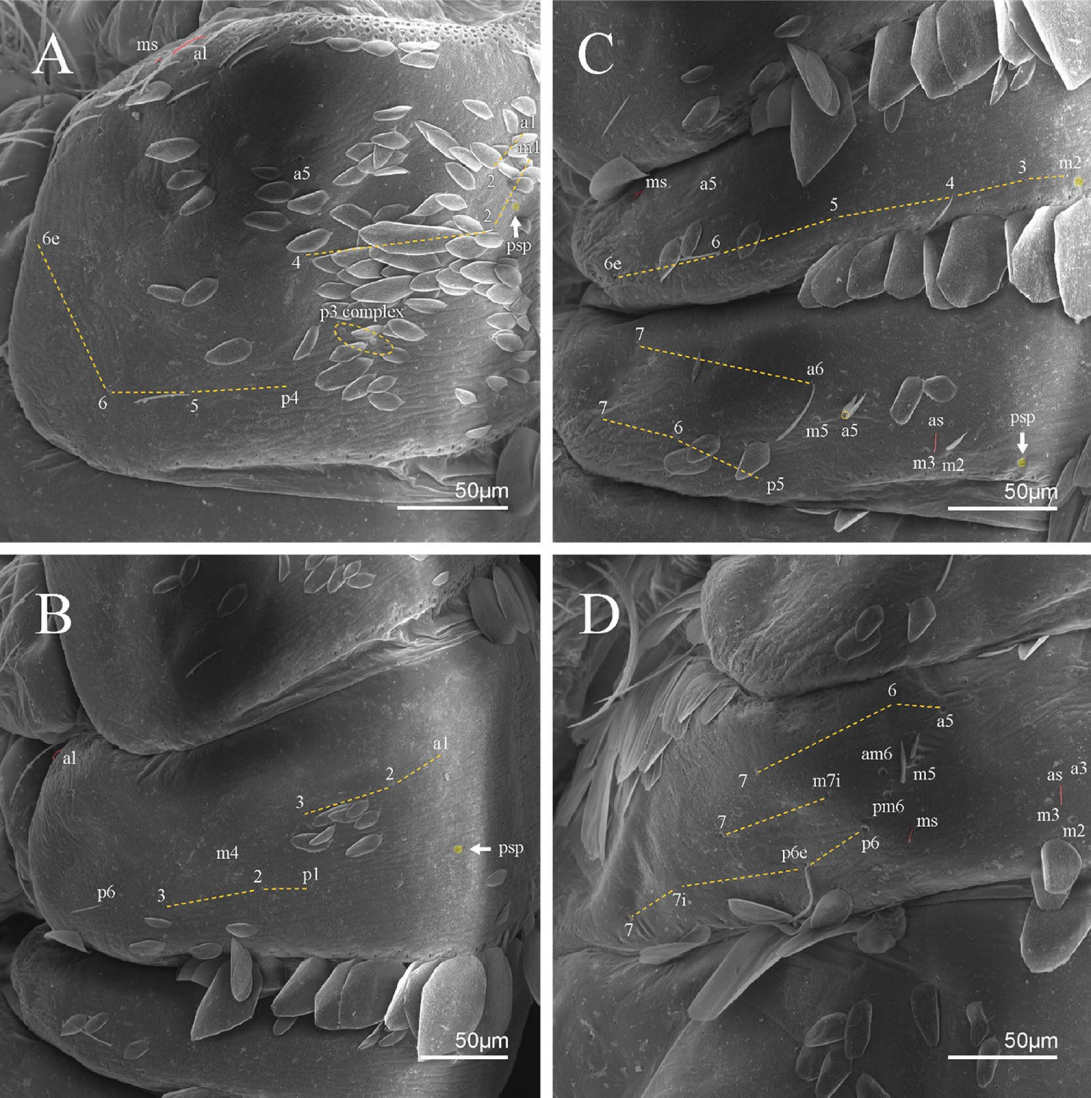
*Trogolaphysa* sp. SEM: thorax and abdomen dorsal chaetotaxy: (**A**) Th II, (**B**) Th III, (**C**) Abd I-II, (**D**) Abd III.

**Figure 8:**
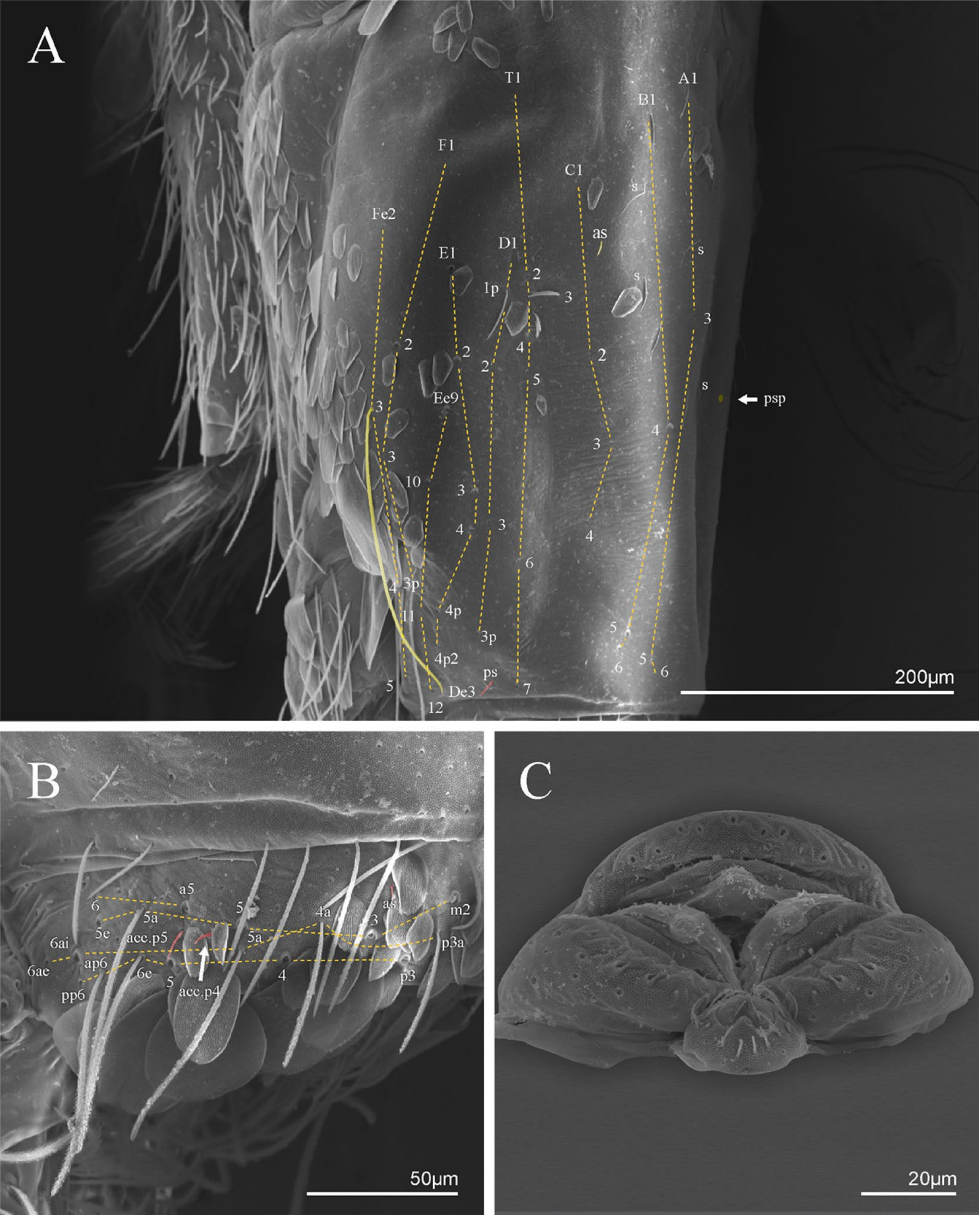
*Trogolaphysa* sp. SEM: (**A**) Abd IV dorsal chaetotaxy, (**B**) Abd V dorsal chaetotaxy, (**C**) anal pore and male genital papilla.

**Figure 9:**
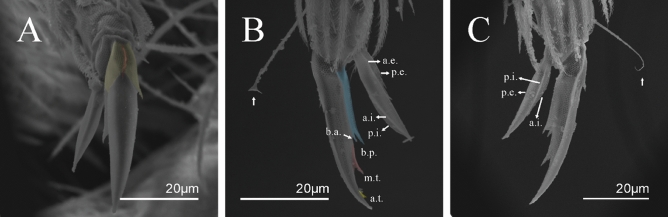
*Trogolaphysa.* sp. SEM: empodial complex III (**A**) external lamella of unguis with external teeth (pseudonychia, yellow), (**B**) unguis and unguiculus lateral view, unguis internal lamella with basal, medial and apical teeth (blue, red and yellow respectively), unguiculus with internal and external teeth, tenent hair capitate (white arrow), (**C**) lateral view, unguiculus lamellae, tenent hair acuminate (white arrow).

**Figure 10:**
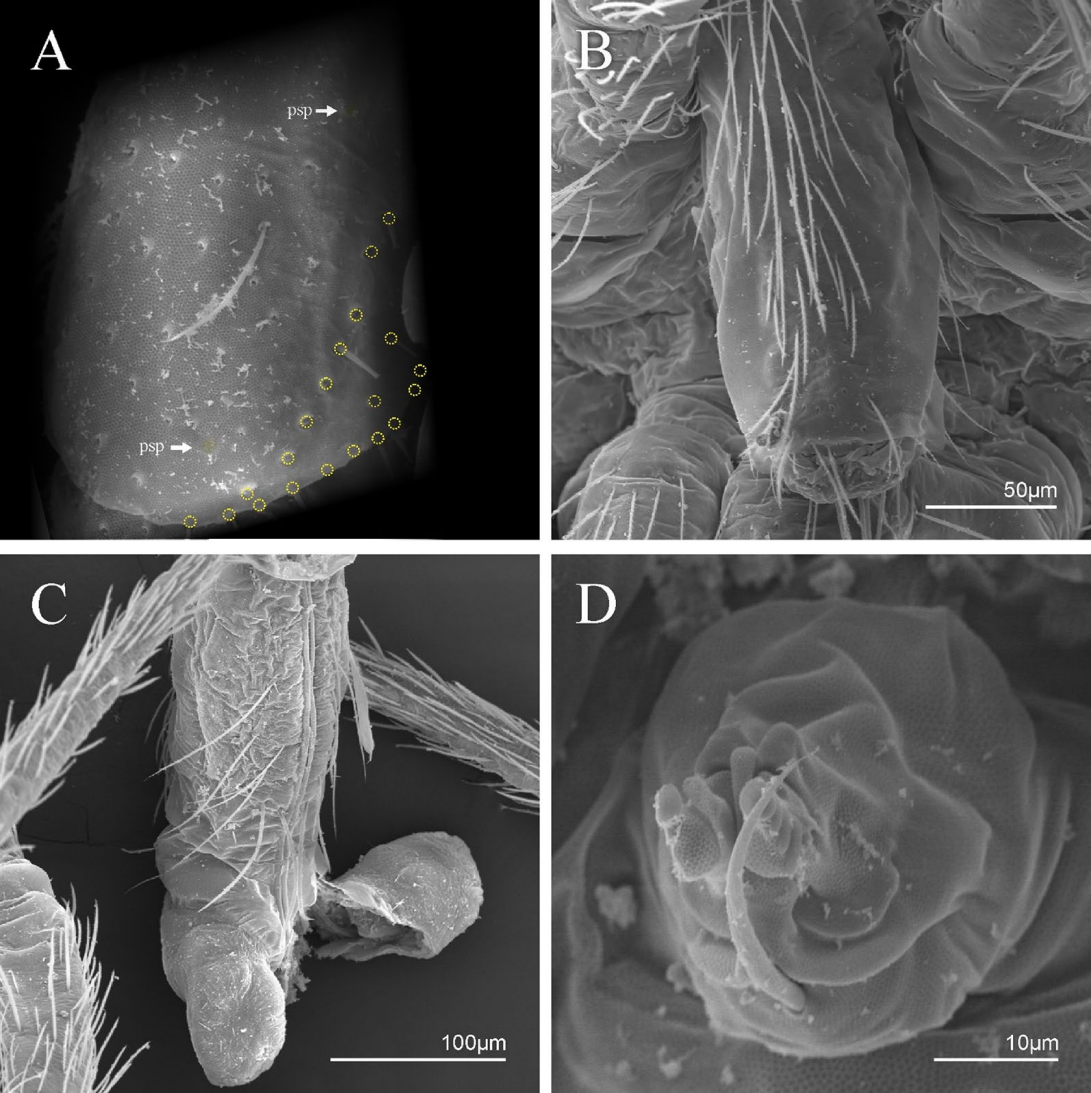
*Trogolaphysa* sp. SEM: appendages (**A**) Metatrochanteral organ with pseudopores (alveoli marked in yellow, white arrows indicate pseudopores), (**B**) ventral tube posterior chaetae, (**C**) ventral tube anterior chaetae, (**D**) Tenaculum.

**Figure 11:**
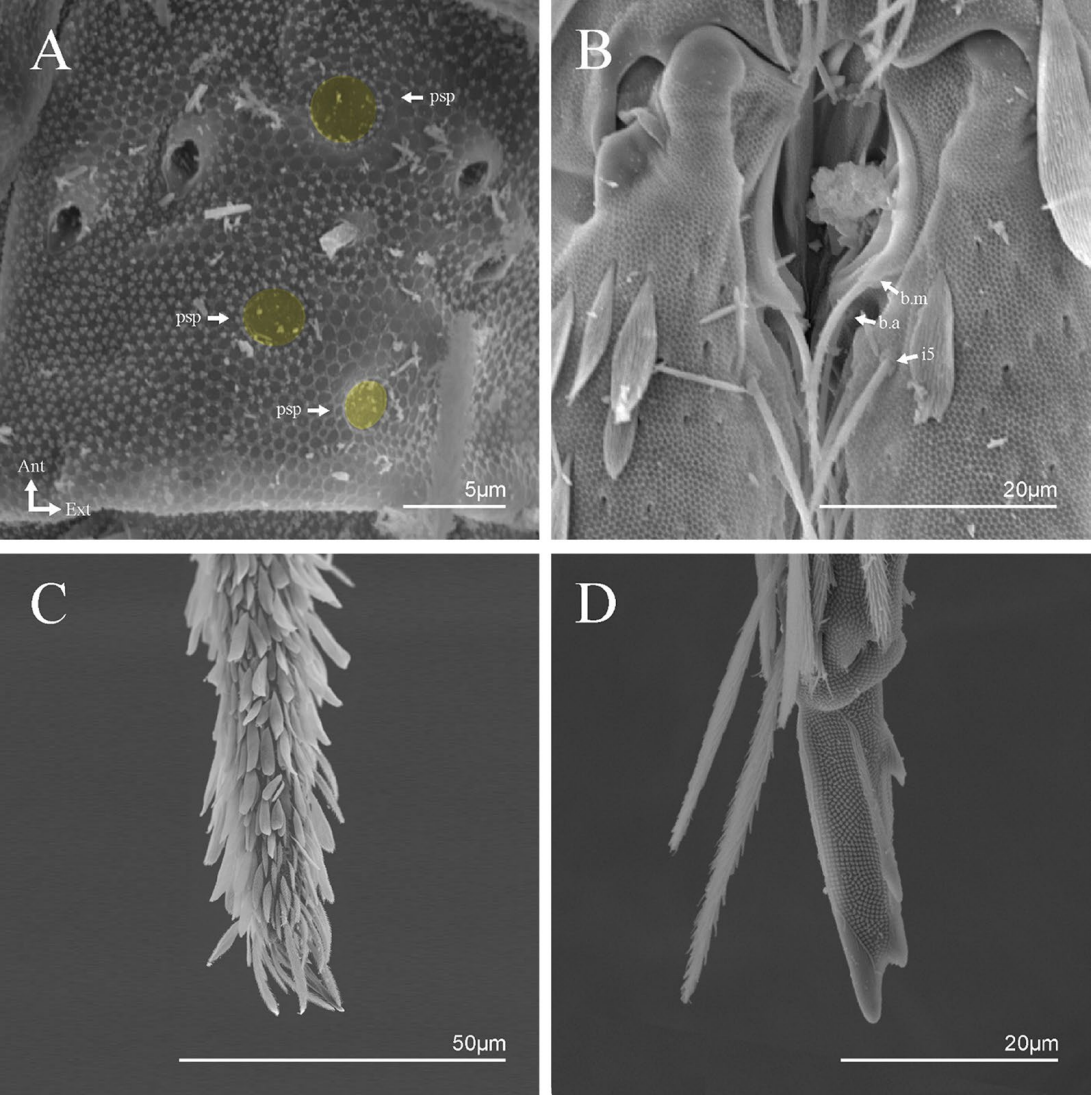
*Trogolaphysa* sp. SEM: furca. (**A**) manubrial plate pseudopores (yellow), (**B**) antero-proximal chaetae of dens, (**C**) dens anterior view, (**D**) mucro*.*

### Diagnosis

Habitus typical of this genus (Fig. 3A–D), hyaline scales presents on Ant. I–II, head, body, and ventral face of furcula (Figs. 3C–D, 4A–C, 5D, F, 7, 8, 11C), Ant IV smooth or annulated and never subdivided in two (Fig. 5A); eyes 0–8 (ex. Fig. 6C); prelabral and labral formula 4/5,5,4 (prelabral smooth or ciliate, pma smooth chaetae) (Fig. 6A); antennobasal-organ present (Fig. 6C); labial chaetae **L1**–**2** not reduced (Fig. 6E); sublobal plate of maxillary palp with 2 chaetae (Fig. 6E); Th II normally with **a5** mac and **p3** complex with variable number of mac, and Th III with **p3** mac present or abset (Fig. 7A, B), abdominal segments II–IV with 2, 3, 3 bothriotricha (Figs. 7C, D, 8A); unguis with three external lamellae and unguiculus with **p.e.** lamella serrate or smooth (Fig. 9A–C); trochanteral organ with 2–4 psp (Fig. 10A) collophore anterior side with 2–3 distal mac (Fig. 10C); tenaculum with four teeth on each branch and one anterior chaeta (Fig. 10D); manubrium without spines, manubrial plate with 2–3 **psp** (Fig. 11A); anterior proximal dens with **b.a.**, **b.m.** and **i5** chaetae (Fig. 11B); dens with 1–2 rows of spines; mucro square or rectangular but relatively short, with 3–5 teeth (Fig. 11D).

*Trogolaphysa bellinii* **sp. nov.** Oliveira, Lima & Zeppelini

**Figure 12:**
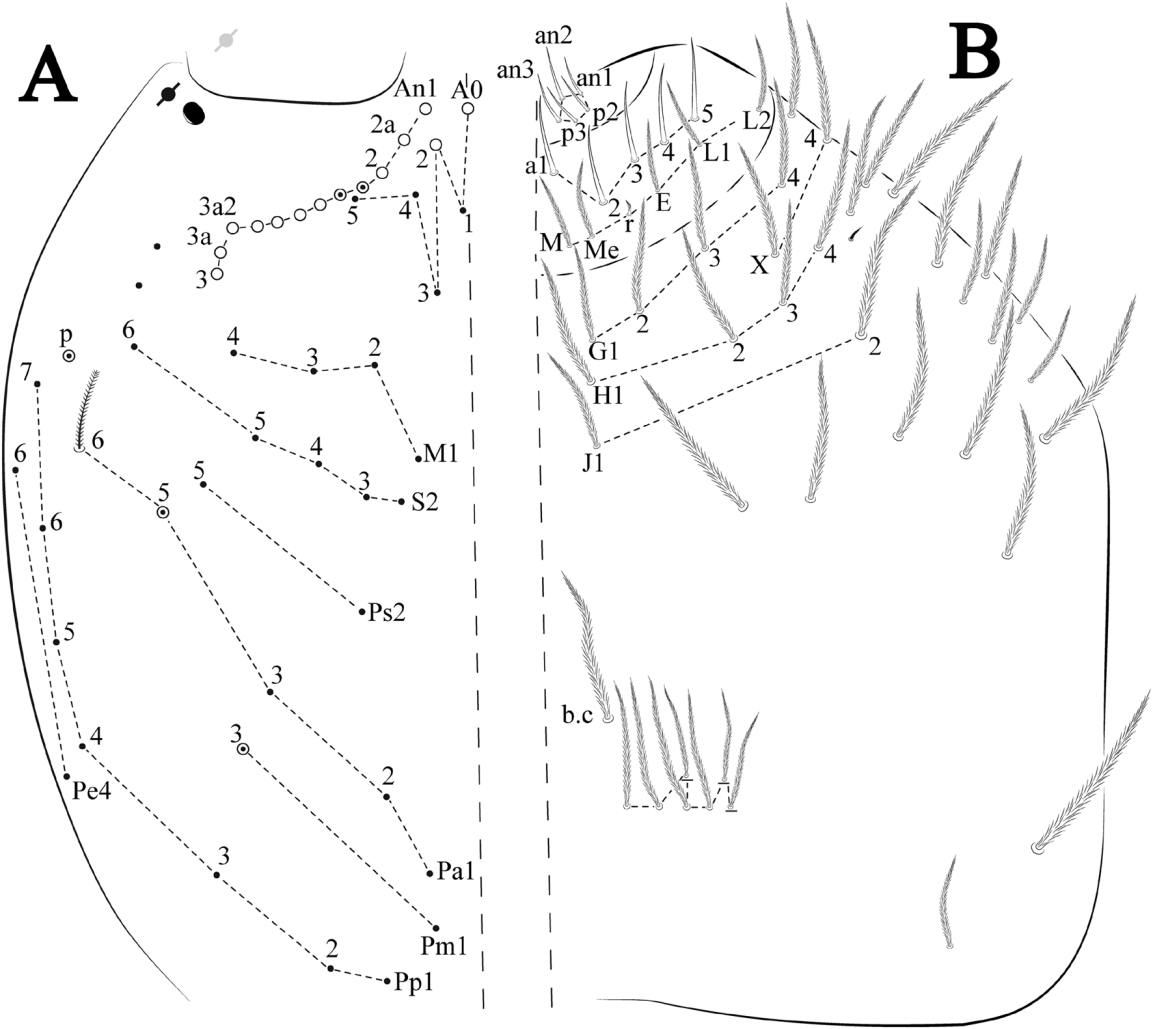
*Trogolaphysa bellinii* **sp. nov.**: (**A**) Head dorsal chaetotaxy, (**B**) labial proximal chaetae, basomedial and basolateral labial fields and postlabial chaetotaxy. Black cut circle, pseudopore; Gray cut circle pseudopore at the under surface.

**Figure 13:**
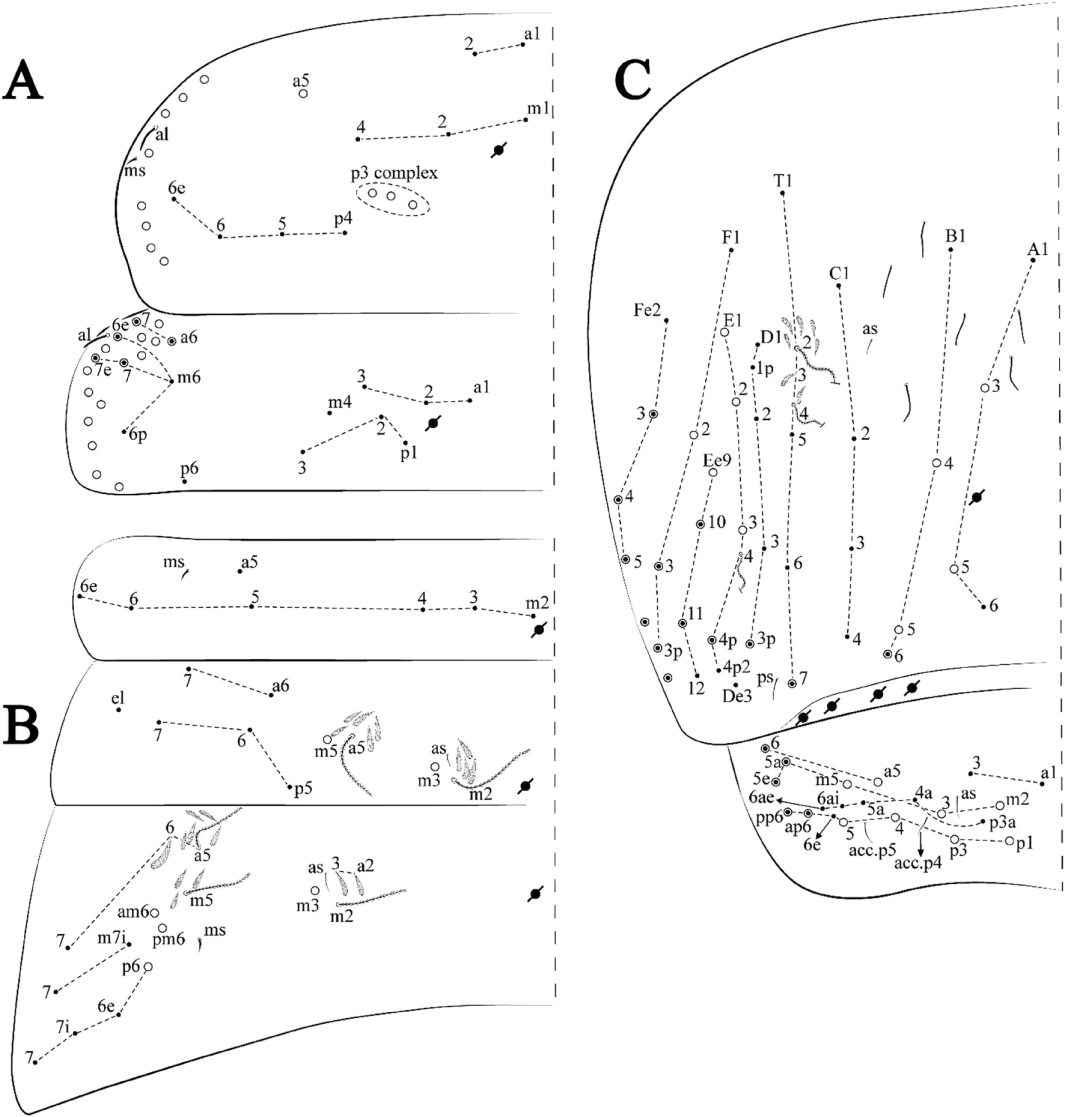
*Trogolaphysa bellinii* **sp. nov.**: Dorsal chaetotaxy: (**A**) Th II–III, (**B**) Abd I–III, (**C**) Abd IV–V.

**Figure 14:**
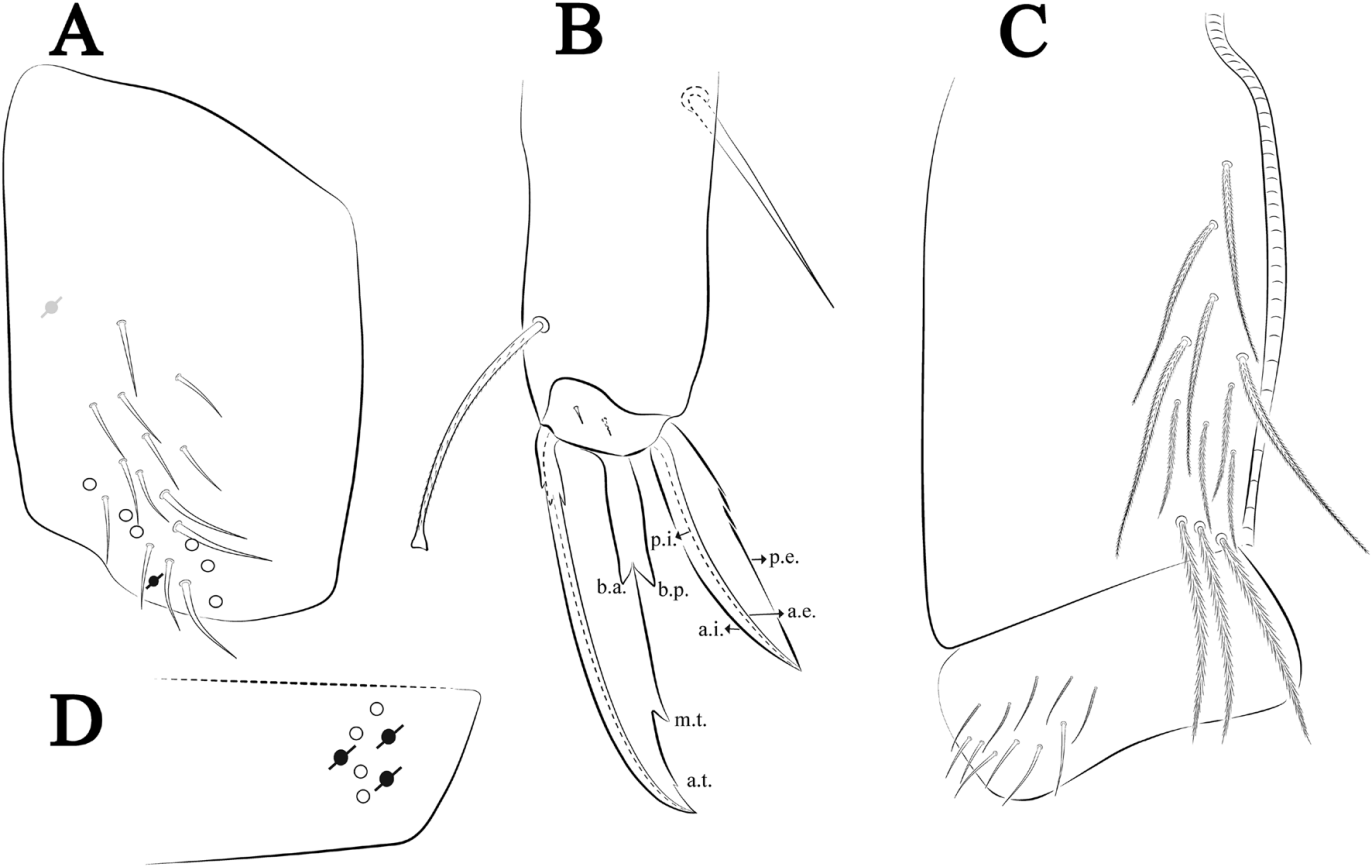
*Trogolaphysa bellinii* **sp. nov.**: (**A**) Trochanteral organ, (**B**) Distal tibiotarsus and empodial complex III (anterior view), (**C**) Manubrial plate, (**D**) Antero-lateral view of collophore chaetotaxy.

Figures 12, 13 and 14, Tables 1 and 2

**Table 2:** *Trogolaphysa* species of the Neotropical Region, comparative morphology.

	Type locality (country)	Habitat	Eyes	Head Dorsal mac	Th II P3 complex	Th III mac	Abd IV mac	Abd IV psp	Trochanteral organ	Unguis inner teeh	Tenent hair apex	Dens outer row spines	Dens inner row spines	Mucro
***T. aelleni*** Yoshii, 1988	Br	Cv	2	?	?	?	?	?	18	3	A	?	?	4
***T. barroca*** sp. nov	Br	Cv	0	A0, A2, Pa5	5	0	A3, B4-5	4	16–21	2	A	37–39	21–22	4
***T. belizeana*** Palacios-Vargas & Thibaud 1997	Be	Cv	0	A0, A2-3, M3, S3, S5, Pa5, Pm3	2	3	A4-5, B5	4^?^	18	2	A	30	31	3
***T. bellinii*** sp. nov	Br	Cv	0–2	A0, A2	3	0	A3, A5, B4-5	4	20	3–4	C	24	25	4
***T. bessoni*** Thibaud & Najt 1988	Ec	Cv	0	?	2	0	A5, B4, B5	?	19	2	A	25	20	4–5
***T. chapelensis*** sp. nov	Br	Cv	0	A0, A2	4	0	A3, A5, B4-5	9	23	3–4	A	> 70	30	4
***T. caripensis*** (Gruia, 1987)	Ve	Cv	0	A0, A2-3, M1-2, S2-3, S5, Pa5, Pm3	6	0	A3, A5, B4-5	?	21	3	A	30–25	30–25	4
***T. crystallensis*** sp. nov	Br	Cv	0	A0, A2	5	0	A3, A5, B4-5	3	18	3	A	58	28	4
***T. dandarae*** sp. nov	Br	Cv	0	A0, A2,S5, Pa5, Pm3	6	3	A3, B4-5	3	19	2	C	31–39	18–21	3
***T. ecuatorica*** (Palacios-Vargas, Ojeda & Christiansen, 1985)	Ec	Cv	0	?	?	?	?	?	2	2	A	45	45	5
***T. epitychia*** sp. nov	Br	Cv	0	A0, A2	3	0	A3, A5, B5	3	15	3	A	60	34	4
***T. ernesti*** Cipola & Bellini, 2017	Br	Lt	8	A0, A2-3	6	0	A3, A5, B4-5	?	49	4	C	21–27	23–30	4
***T. formosensis*** Silva & Bellini, 2015	Br	Lt	8	A0, A2, M2, S3, S5, Pa5	6	3	?	3	12	4	C	?	?	5
***T. gisbertae*** sp. nov	Br	Cv	0	A0, A2-3, Pa5, Pm3	5	0	A3, B4-5	1	25	3	A	38	21–24	4
***T. haitica*** (Palacios-Vargas, Ojeda & Christiansen, 1985)	Ha	Cv	0	?	?	?	?	?	22	2	A	30–38	30–38	4
***T. hauseri*** Yoshii, 1988	Br	Cv^?^	0	?	?	?	?	?	15	2	A	14	30	4
***T. hirtipes*** (Handschin, 1924)	Br	Tp	8^?^	?	?	?	?	?	?	4^?^	C	?	?	4
***T. hondurensis*** (Palacios-Vargas, Ojeda & Christiansen, 1985)	Ho	Cv	4	?	?	?	?	?	?	2	A	30–36	30–36	4
***T. jacobyi*** Soto-Adames & Taylor 2013	Be	Cv	0	A0, A2-3 ,M2, S3, S5 ,Pa5, Pm3	2	1	A5, B4, B5	?	25	3	A	?	36	3
***T. lacerta*** sp. nov	Br	Cv	0–3	A0, A2	6	0	A3, B4-5	5	24	4	A	50	37	4
***T. mariecurieae*** sp. nov	Br	Ms	0	A0, A2	3	0	A4 B5	4	15	3	C	40	22	4
***T. marimutti*** (Palacios-Vargas, Ojeda & Christiansen, 1985)	Me	Cv	0	?	?	?	?	?	15	3	A	50	50	4
***T. millsi*** Arlé, 1939	Br	Lt	2	?	?	?	?	?	?	3	A^?^	?	?	4
***T. oztotlica*** (Ojeda & Palacios-Vargas, 1984)	Me	Cv	0	Pa5, Pm3^?^	?	?	?	?	14	4	A	35–40	35–40	4
***T. piracurucaensis*** Nunes & Bellini, 2018	Br	Lt	8	A0, A2-3, M2, S3, S5, Pa5, Pm3	6	0	A3, A5, B4-5	?	35–40	4	C	21–27	25–29	4
***T. sotoadamesi*** sp. nov	Br	Cv	0	A0, A2	5	0	B4-5	4	19–21	2	A	35	21–26	4
***T. tijucana*** (Arlé & Guimarães, 1979)	Br	Lt	2	?	?	?	?	?	?	2	A?	?	?	4
***T. trioculata*** Soto-Adames, 2015	Me	Lt	3	A0, A2, Pa5, Pm3	5	0	A3, A5, B5	4	14	3	A	12	15	4
***T. xtolokensis*** (Palacios-Vargas, Ojeda & Christiansen, 1985)	Me	Cv	0	A0, A2, Pa5^?^	?	?	?	?	20	4	A	41–69	41–69	4
***T. zampauloi*** sp. nov	Br	Cv	0–4	A0, A2	5	0	A3, A5, B4-5	3	27	4	A	30	23	4

Be, Belize; Br, Brazil; Ec, Ecuador; Ha, Haiti; Ho, Honduras; Me, Mexico. Cv, cave; Lt, Leaflitter, Tp, Termitophile, Ms, Mesovoid shallow substratum. ?, lacking or dubious information.

*Type material.* Holotype female in slide (15,482/CRFS-UEPB): Brazil, Minas Gerais State, Barão de Cocais municipality, cave MDIR-0028, next to “Mina de Brucutu”, 19°52′48.7″S, 43°26′13.6″W, 19–23.viii.2019, Carste team coll. Paratypes in slides (15,468, 15,483/CRFS-UEPB): 2 females, same data as holotype. Paratypes in slides (15,519, 15,576/CRFS-UEPB donated to MNJR): 2 females, same data as holotype. Additional records see S1.

*Description.* Total length (head + trunk) of specimens 1.53–1.75 mm (n = 5), holotype 1.70 mm.

Head. Ratio antennae: trunk = 1: 1.29–1.95 (n = 5), holotype = 1: 1.95; Ant III shorter than Ant II; Ant segments ratio as I: II, III, IV = 1: 1.80–2.24, 0.85–2.08, 0.85–2.08, holotype = 1: 1.80, 0.85, 1.34. Antennal chaetotaxy: Ant IV dorsally and ventrally with several short ciliate mic and mac, and finger-shaped sens, dorsally with a longitudinal row with about eight rod sens, ventrally with one subapical-organ and several wrinkly sens (Fig. 4A); Ant III dorsally and ventrally with several short ciliate mic and mac, and finger-shaped sens, dorsally without modified sens, ventrally with one apical **psp**, about three wrinkly sens on external longitudinal row, apical organ with two mic smooth chaetae externally, two coffee bean-like sens, and one rod sens (Fig. 4A); Ant II dorsally and ventrally with several short ciliate mic and mac, dorsally with four sub-apical finger-shaped sens, one wrinkly sens and two subapical rod sens, ventrally with one apical **psp**, about six wrinkly sens on longitudinal external row (Fig. 4A); and Ant I dorsally and ventrally with several short ciliate mic and mac, dorsally with three basal spine-like sens, ventrally with four basal spine-like sens, about five smooth mic and several finger-shaped sens (Fig. 4A). Eyes 0 + 0, rarely 2 + 2. Head dorsal chaetotaxy (Fig. 12A) with 12 **An** (**An1a**–**3**), six **A** (**A0**–**5**), five **M** (**M1**–**5**), five **S** (**S2**–**6**), two **Ps** (**Ps2**, **Ps5**), four **Pa** (**Pa1**–**5**), two **Pm** (**Pm1**, **Pm3**), seven **Pp** (**Pp1**–**7**), and two **Pe** (**Pe4**, **Pe6**) chaetae; **Pa5** and **Pm3** as mes, **An1a**–**3a** with 10 mac plus two mes, **A0** and **A2** as mac; interocular **p** mes present. Basomedian and basolateral labial fields with **a1**–**5** smooth, **M**, **Me**, **E** and **L1**–**2** ciliate, **r** reduced (Fig. 12B). Ventral chaetotaxy with 35–38 ciliate chaetae and one reduced lateral spine; postlabial **G1**–**4**; **X**, **X4**; **H1**–**4**; **J1**–**2**, chaetae **b.c.** present and a collar row of four to seven mes chaetae distally (Fig. 12B). Prelabral chaetae ciliate. Labral chaetae smooth, no modifications. Labial papilla **E** with **l.p.** finger-shaped and surpassing the base of apical appendage. Labial proximal chaetae smooth (**an1**–**3**, **p2**–**3**) and subequal in length (Fig. 12B). Maxillary palp with **t.a.** smooth and 1.23× larger than **b.c**.

Thorax dorsal chaetotaxy (Fig. 13A). Th II **a**, **m**, **p** series with two mic (**a1**–**2**), one mac (**a5**), three mic (**m1**–**2**, **m4**) and four mic (**p4**–**6e**), **p3** complex with three mac, respectively, **al** and **ms** present. Th III **a**, **m**, **p** series with three mic (**a1**–**3**), two mes (**a6**–**7**), three mic (**m4**, **m6**–**6p**), three mes (**m6e**, **m7**–**7e**), four mic (**p1**–**3**, **p6**) respectively. Ratio Th II: III = 1.04–1.36: 1 (n = 5), holotype = 1.05: 1.

Abdomen dorsal chaetotaxy (Fig. 13B, C). Abd I **a**, **m** series with one (**a5**) and six (**m2**–**6e**) mic respectively, **ms** present. Abd II **a, m**, **p** series with two mic (**a6**–**7**), two mac (**m3**, **m5**), three mic (**p5**–**7**) respectively, **el** mic and **as** present; **a5** and **m2** bothriotricha surrounded by five and four fan-shaped chaetae respectively. Abd III **a**, **m**, **p** series with one mic (**a7**), three fan-shaped chaetae (**a2**–**3**, **a6**), two mic (**m7i**–**7**), three mac (**m3**, **am6**, **pm6**), three mic (**p6e**, **p7i–7**), one mac (**p6**) chaetae respectively; **a5**, **m2** and **m5** bothriotricha with six, two and three fan-shaped chaetae respectively, **as** sens elongated, **ms** present. Abd IV **A**–**Fe** series with two mic (**A1**, **A6**), two mac (**A3**, **A5**), one mic (**B1**), one mes (**B6**), two mac (**B4–5**), four mic (**C1**–**4**), three mic (**T1**, **T5**–**6**), one mes (**T7**), five mic (**D1**–**3**, **De3**), one mes (**D3p**), one mic (**E4p2**), one mes (**E4p**), three mac (**E1**–**3**), one mic (**Ee12**), two mes (**Ee10**–**11**), one mac (**Ee9**), one mic (**F1**), two mes (**F3**, **F3p**), one mac (**F2**), one mic (**Fe2**), three mes (**Fe3**–**5**) chaetae, respectively; **T2**, **T4** and **E4** bothriotricha surrounded by five and two (**T3**) fan-shaped chaetae respectively; **ps** and **as** present, and at least six supernumerary sens with uncertain homology *‘s’* (Fig. 8A); Abd. IV posteriorly with four **psp**. Abd V **a**, **m**, **p** series with two mic (**a1**, **a3**), one mes (**a6**), one mac (**a5**), two mes (**m5a**, **m5e**), three mac (**m2**–**3**, **m5**), five mic (**p3a**–**6ae**), one mic (**p6e**) two mes (**ap6**–**pp6**), four mac (**p1**, **p3**–**5**) chaetae, respectively; **as**, **acc**.**p4**–**5** present. Ratio Abd III: IV = 1: 3.70–4.37 (n = 5), holotype = 1: 4.37.

Legs. Trochanteral organ diamond shape with about 20 spine-like chaetae, plus two **psp** one external and one on distal vertex of Omt (Fig. 14A). Unguis outer side with one paired tooth straight and not developed on proximal third; inner lamella wide with four teeth, basal pair subequal, **b.p.** not reaching the **m.t.** apex, **m.t.** just after the distal half, **a.t.** present. Unguiculus with lamellae smooth and lanceolate (**a.i.**, **a.e.**, **p.i.**), except **p.e.** slightly serrate (Fig. 14B); ratio unguis: unguiculus = 1.56–1.79: 1 (n = 5), holotype = 1.56: 1. Tibiotarsal smooth chaetae about 0.9 × smaller than unguiculus; tenent hair capitate and about 0.55 × smaller than unguis outer lamella.

Collophore (Fig. 14C). Anterior side with 12 ciliate, apically acuminate chaetae, five proximal, four subdistal (as mes) and three distal mac; lateral flap with 11 chaetae, five ciliate in the proximal row and six smooth in the distal row.

Furcula. Covered with ciliate chaetae, spine-like chaetae and scales. Manubrial plate with four ciliate chaetae (two inner mac) and three **psp** (Fig. 14D). Dens posterior face with two or more longitudinal rows of spine-like chaetae about 24 external and 25 internal, external spines larger and thinner than internal ones. Mucro with four teeth, ratio width: length = 0.29 (holotype).

*Etymology.* Species named after Dr. Bruno C. Bellini in recognition of his work on Brazilian Collembola.

*Remarks. Trogolaphysa bellinii* **sp. nov**. resembles *T. bessoni*, *T. epitychia* **sp. nov.***,* and *T. mariecurieae* **sp. nov.** by 0 + 0 eyes (*T. bellinii* **sp. nov.** rarely with 2 + 2 eyes), Th II with 3 + 3 mac, and Th III without mac, but can be distinguished by presenting Abd IV with 4 + 4 central mac (**A3**, **A5**, **B4**–**5**); *T. epitychia* **sp. nov.** with 3 + 3 central mac on Abd IV, *T. mariecuriea*e **sp. nov.** with 2 + 2 central mac on Abd IV.

*Trogolaphysa lacerta* **sp. nov.** Lima, Oliveira & Zeppelini

**Figure 15:**
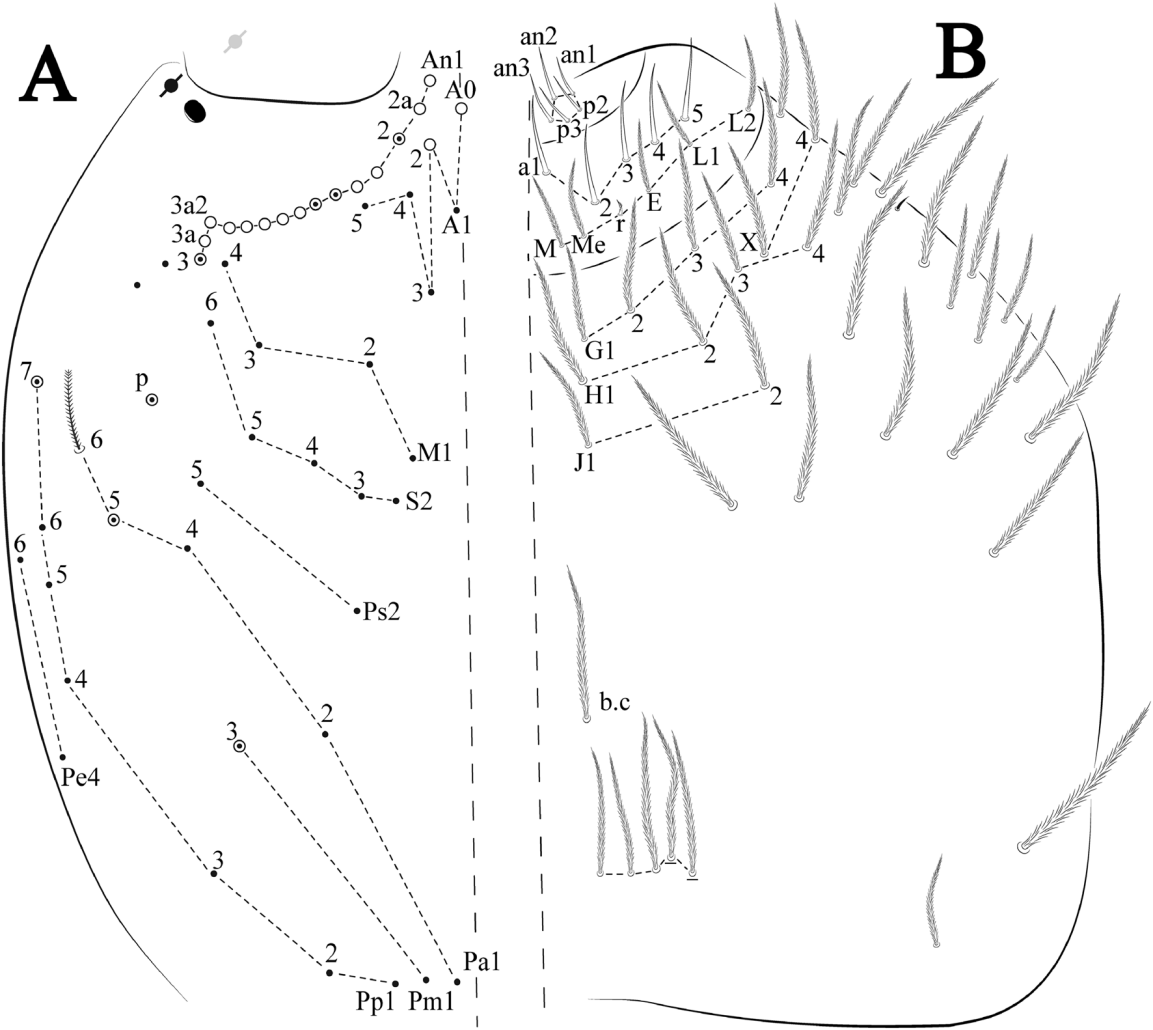
*Trogolaphysa lacerta* **sp. nov.**: (**A**) Head dorsal chaetotaxy, (**B**) labial proximal chaetae, basomedial and basolateral labial fields and postlabial chaetotaxy. Black cut circle, pseudopore; Gray cut circle pseudopore at the under surface.

**Figure 16:**
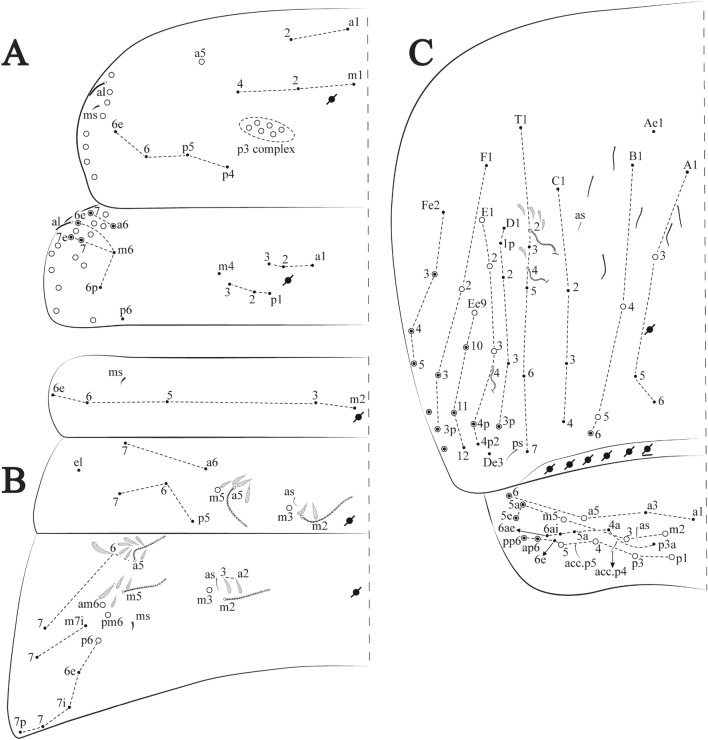
*Trogolaphysa lacerta* **sp. nov.**: Dorsal chaetotaxy. (**A**) Th II–III, (**B**) Abd I–III, (**C**) Abd IV–V.

**Figure 17:**
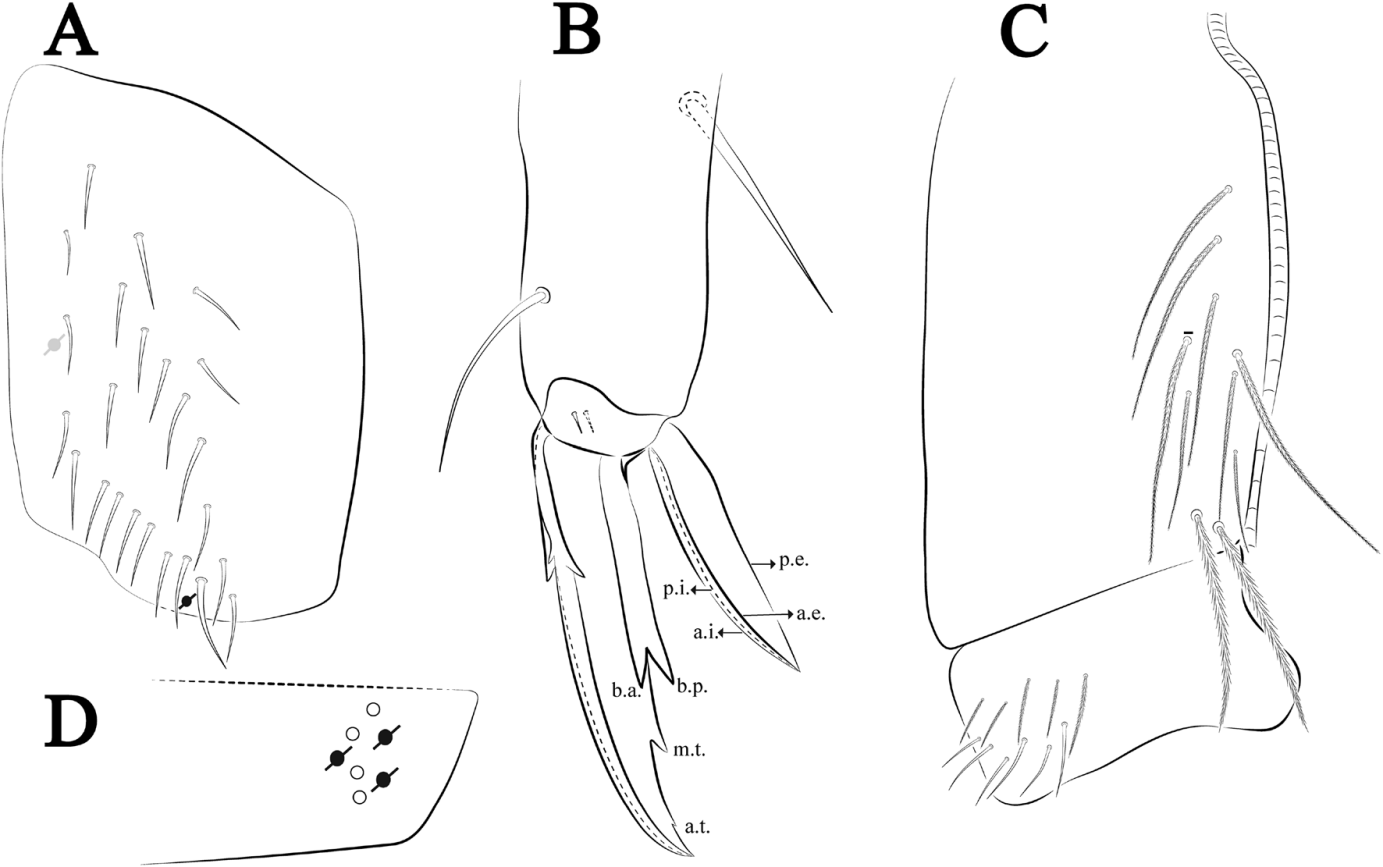
*Trogolaphysa lacerta* **sp. nov.**: (**A**) Trochanteral organ, (**B**) Distal tibiotarsus and empodial complex III (anterior view), (**C**) Manubrial plate, (**D**) Antero-lateral view of collophore chaetotaxy.

Figures 15, 16 and 17, Tables 1 and 2

*Type material.* Holotype male in slide (10,311/CRFS-UEPB): Brazil, Minas Gerais State, Conceição do Rio Acima municipality, cave GAND-115, next to “Lapa do Calango”, 20°04′08.4″S, 43°40′09.9″W, 10.ii–20.iii.2014, Carste team coll. Paratypes in slides (10,312, 10,309/CRFS-UEPB): 2 males, same data as holotype. Paratypes in slides (10,313, 10,314/CRFS-UEPB donated to MNJR): 2 females, same data as holotype. Additional records see S1.

*Description.* Total length (head + trunk) of specimens 1.31–2.43 mm (n = 5), holotype 1.86 mm.

Head. Ratio antennae: trunk = 1: 1.33–1.46 (n = 2), holotype = 1: 1.46; Ant III shorter than Ant II; Ant segments ratio, I: II, III, IV = 1: 1.78–2.05: 1.5–1.64: 2.64–2.83, holotype = 1: 1.80: 1.64: 2.64. Antennal chaetotaxy (no represented): Ant IV dorsally and ventrally with several short ciliate mic and mac, and finger-shaped sens, dorsally with a longitudinal row with about five rod sens, ventrally with one subapical-organ and several wrinkly sens (Fig. 4A); Ant III dorsally and ventrally with several short ciliate mic and mac, and finger-shaped sens, dorsally without modified sens, ventrally with one apical **psp**, one apical wrinkly sens on, apical organ with two coffee bean-like sens, and one rod sens (Fig. 4A); Ant II dorsally and ventrally with several short ciliate mic and mac, dorsally with three sub-apical finger-shaped sens, one wrinkly sens and two apical rod sens, ventrally with one apical **psp**, one longitudinal external row with two subapical wrinkly sens and two medial finger-shaped sens (Fig. 4A); and Ant I dorsally and ventrally with several short ciliate mic and mac, dorsally with three basal spine-like sens, ventrally with four basal spine-like sens, about five smooth mic and several finger-shaped sens (Fig. 4A). Eyes 0 + 0, rarely 3 + 3. Head dorsal chaetotaxy (Fig. 15A) with 15 **An** (**An1a**–**3**), six **A** (**A0**–**5**), four **M** (**M1**–**4**), five **S** (**S2**–**6**), two **Ps** (**Ps2**, **Ps5**), four **Pa** (**Pa1**–**2**, **Pa4**–**5**), two **Pm** (**Pm1**, **Pm3**), seven **Pp** (**Pp1**–**7**), and two **Pe** (**Pe4**, **Pe6**) chaetae; **Pm3**, **Pa5** and **Pp7** as mes, **An1a**–**3a** with 11 mac plus four meso, **A0** and **A2** as mac; interocular **p** mes present. Basomedian and basolateral labial fields with **a1**–**5** smooth, **M**, **Me**, **E** and **L1**–**2** ciliate, **r** reduced (Fig. 15B). Ventral chaetotaxy with 36–38 ciliate chaetae and 1 reduced lateral spine; postlabial **G1**–**4**; **X**, **X4**; **H1**–**4**; **J1**–**2**, chaetae **b.c.** present and a collar row of three to five mes chaetae distally (Fig. 15B). Prelabral chaetae ciliate. Labral chaetae smooth, no modifications. Labial papilla **E** with **l.p**. finger-shaped and surpassing the base of apical appendage. Labial proximal chaetae smooth (**an1**–**3**, **p2**–**3**) and subequal in length (Fig. 15B). Maxillary palp with **t.a.** smooth and 1.28× larger than **t.a**.

Thorax dorsal chaetotaxy (Fig. 16A). Th II **a**, **m**, **p** series with two mic (**a1**–**2**), one mac (**a5**), three mic (**m1**–**2**, **m4**) and four mic (**p4**–**6e**), **p3** complex with six mac, respectively, **al** and **ms** present. Th III **a**, **m**, **p** series with three mic (**a1**–**3**), two mes (**a6**–**7**), three mic (**m4**, **m6–6p**), three mes (**m6e**, **m7–7e**), four mic (**p1**–**3**, **p6**) respectively. Ratio Th II: III = 1.09–1.46: 1 (n = 5), holotype = 1.09: 1.

Abdomen dorsal chaetotaxy (Fig. 16B, C). Abd I **m** series with six (**m2**–**6e**) mic respectively, **ms** present. Abd II **a**, **m**, **p** series with two mic (**a6**–**7**), two mac (**m3**, **m5**), three mic (**p5**–**7**) respectively, **el** mic and **as** present; **a5** and **m2** bothriotricha surrounded by four and two fan-shaped chaetae respectively. Abd III **a**, **m**, **p** series with one mic (**a7**), three fan-shaped chaetae (**a2**–**3**, **a6**), two mic (**m7i**–**7**), three mac (**m3**, **am6**, **pm6**), four mic (**p6e**, **p7i–7p**), one mac (**p6**) chaetae respectively; **a5**, **m2** and **m5** bothriotricha with seven, two and four fan-shaped chaetae respectively, **as** sens elongated, **ms** present. Abd IV **A**–**Fe** series with four mic (**A1**, **A5**–**6**, **Ae1**), one mac (**A3**), one mic (**B1**), one mes (**B6**), two mac (**B4**–**5**), four mic (**C1**–**4**), five mic (**T1**, **T3**, **T5**–**7**), five mic (**D1**–**3**, **De3**), one mes (**D3p**), one mic (**E4p2**), one mes (**E4p**), three mac (**E1**–**3**), one mic (**Ee12**), two mes (**Ee10**–**11**), one mac (**Ee9**), one mic (**F1**), two mes (**F3–3p**), one mac (**F2**), one mic (**Fe2**), three mes (**Fe3**–**5**) chaetae, respectively; **T2**, **T4** and **E4** bothriotricha surrounded by four and one fan-shaped chaetae respectively; **ps** and **as** present, and at least six supernumerary sens with uncertain homology *‘s’*(Fig. 8A); Abd. IV posteriorly with five to six **psp**. Abd V **a**, **m**, **p** series with two mic (**a1**, **a3**), one mes (**a6**), one mac (**a5**), two mes (**m5a**, **m5e**), three mac (**m2**–**3**, **m5**), five mic (**p3a**–**6ae**), one mic (**p6e**) two mes (**ap6**–**pp6**), four mac (**p1**, **p3**–**5**) chaetae, respectively; **as**, **acc**.**p4**–**5** present. Ratio Abd III: IV = 1: 3.70–4.37 (n = 5), holotype = 1: 4.37.

Legs. Trochanteral organ diamond shape with about 24 spine-like chaetae, plus two **psp** one external and one on distal vertex of Omt (Fig. 17A). Unguis outer side with one paired tooth straight and not developed on proximal third; inner lamella wide with four teeth, basal pair subequal, **b.p.** not reaching the **m.t.** apex, **m.t.** just after the distal half, **a.t.** present. Unguiculus with all lamellae smooth and lanceolate (**a.i.**, **a.e.**, **p.i.**, **p.e.**) (Fig. 17B); ratio unguis: unguiculus = 1: 1.50–1.79 (n = 5), holotype = 1: 1.75. Tibiotarsal smooth chaetae about 0.7× smaller than unguiculus; tenent hair slightly acuminate and about 0.44× smaller than unguis outer lamella.

Collophore (Fig. 17C). Anterior side with 10 ciliate, apically acuminate chaetae, five proximal (thinner); three subdistal and two distal mac; lateral flap with 11 chaetae, five ciliate in the proximal row and six smooth in the distal row.

Furcula. Covered with ciliate chaetae, spine-like chaetae and scales. Manubrial plate with four ciliate chaetae (two inner mac) and three **psp** (Fig. 17D). Dens posterior face with two or more longitudinal rows of spine-like chaetae about 50 external and 37 internal, external spines larger and thinner than internal ones. Mucro with four teeth, ratio width: length = 0.31 (n = 5).

*Etymology. Lacerta* from Latin means lizard, in allusion to the name of the cave where this species was found, *Lapa do Calango* (cave of the *Calango*), which is a small lizard common in this region.

*Remarks. Trogolaphysa lacerta* **sp. nov.** The new species resembles *T. caripensis, T. ernesti, T. piracurucaensis, T. formosensis* and *T. dandarae* **sp. nov**. by the number of mac in Th II **p3** complex (6 + 6), but is easily distinguished by the head **m2** and **s5** mic (*T. caripensis*, *T. ernesti*, *T. formosensis*, *T. piracurucaensis* as mac) and Th III without mac (*T. dandarae ***sp. nov.** 3 + 3).

*Trogolaphysa chapelensis* **sp. nov.** Lima, Oliveira & Zeppelini

**Figure 18:**
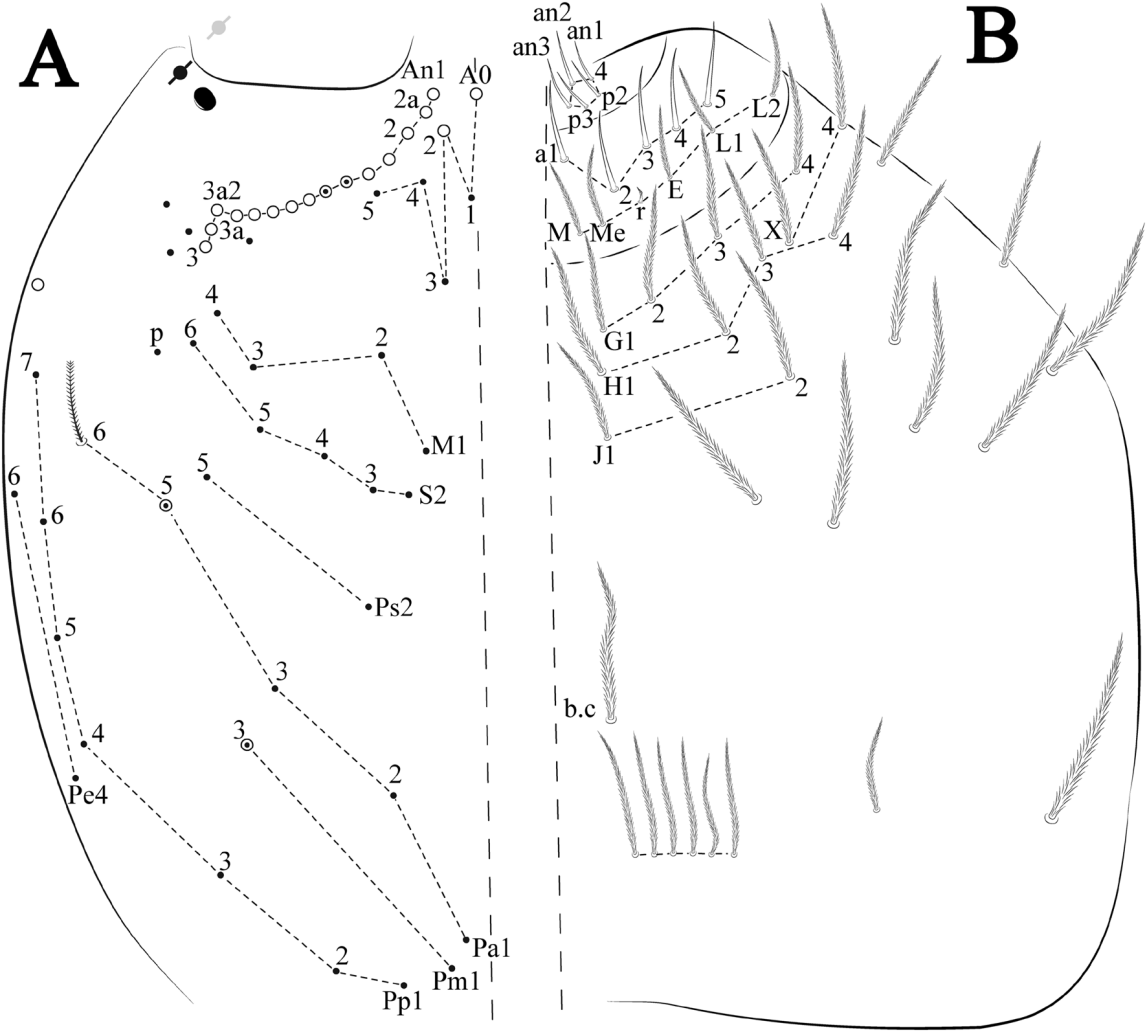
*Trogolaphysa chapelensis* **sp. nov.**: (**A**) Head dorsal chaetotaxy, (**B**) labial proximal chaetae, basomedial and basolateral labial fields and postlabial chaetotaxy. Black cut circle, pseudopore; Gray cut circle pseudopore at the under surface.

**Figure 19:**
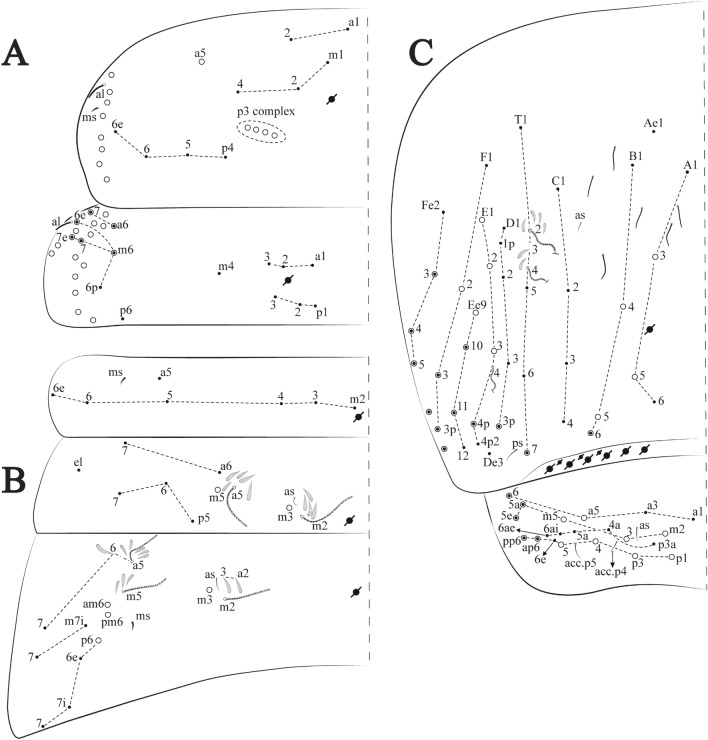
*Trogolaphysa chapelensis* **sp. nov.**: Dorsal chaetotaxy. (**A**) Th II–III, (**B**) Abd I–III, (**C**) Abd IV–V.

**Figure 20:**
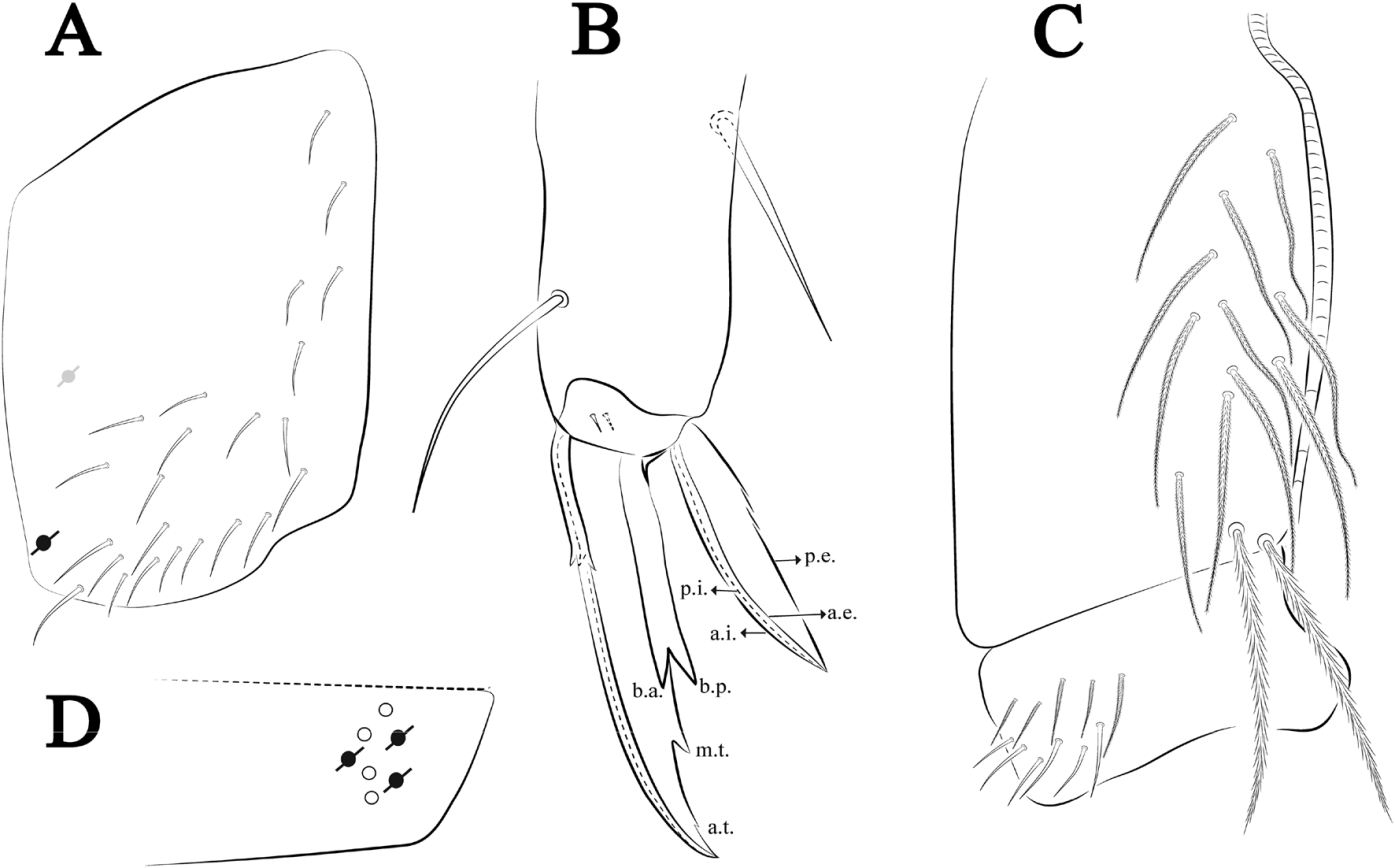
*Trogolaphysa chapelensis* **sp. nov.**: (**A**) Trochanteral organ, (**B**) Distal tibiotarsus and empodial complex III (anterior view), (**C**) Manubrial plate, (**D**) Antero-lateral view of collophore chaetotaxy.

Figures 18, 19 and 20, Tables 1 and 2

*Type material.* Holotype female in slide (4550/CRFS-UEPB): Brazil, Minas Gerais State, Rio Acima municipality, cave Gruta-2d7, next to “Morro do Chapéu” 20°07′42.1″S, 43°54′26.2″W, 02–10.viii.2011, Andrade et al. coll. Paratypes in slides (4551–4553/CRFS-UEPB): 3 females, Brazil, Minas Gerais State, Rio Acima municipality, cave Gruta-7d7, Qd7, 9d7 respectively, 20°07′42.1″S, 43°54′26.7″W, 29.iii–01.vi.2011, Andrade et al. coll. Paratype in slide (4603/CRFS-UEPB donated to MNJR): 1 female, Brazil, Minas Gerais State, Rio Acima municipality, cave Gruta Qd7, 20°09′46.1″S, 43°49′36.2″W, 925 m, 29.iii–01.vi.2011, Andrade et al. Coll. Additional records see S1.

*Description.* Total length (head + trunk) 1.21–2.22 mm (n = 5), holotype 2.22 mm.

Head. Ratio antennae: trunk = 1: 1.31–1.16 (n = 3), holotype = 1: 1.16; Ant III shorter than Ant II; Ant segments ratio, I: II, III, IV = 1: 1.66–1.85, 1.65–1.78, 2.95–3.76, holotype = 1: 1.66, 1.65, 2.95. Antennal chaetotaxy (no represented): Ant IV dorsally and ventrally with several short ciliate mic and mac, and finger-shaped sens, dorsally with about six rod sens on longitudinal row, ventrally with one subapical-organ and about three subapical wrinkly sens (Fig. 4A); Ant III dorsally and ventrally with several short ciliate mic and mac, and finger-shaped sens, dorsally without modified sens, ventrally with one apical **psp**, one apical wrinkly sens, apical organ with two coffee bean-like sens, and one rod sens (Fig. 4A); Ant II dorsally and ventrally with several short ciliate mic and mac, dorsally with about three sub-apical finger-shaped sens and about three apical rod sens, ventrally with one apical **psp**, one longitudinal external row with four wrinkly sens (Fig. 4A); and Ant I dorsally and ventrally with several short ciliate mic and mac, dorsally with three basal spine-like sens, ventrally with four basal spine-like sens, about three smooth mic and several finger-shaped sens (Fig. 4A). Eyes 0 + 0. Head dorsal chaetotaxy (Fig. 18A) with 15 **An** (**An1a**–**3**), six **A** (**A0**–**5**), four **M** (**M1**–**4**), five **S** (**S2**–**6**), two **Ps** (**Ps2**, **Ps5**), four **Pa** (**Pa1**–**5**), two **Pm** (**Pm1**, **Pm3**), seven **Pp** (**Pp1**–**7**), and two **Pe** (**Pe4**, **Pe6**) chaetae; **Pm3** and **Pa5** as mes, **An1a**–**3a** with 13 mac plus two mes, **A0** and **A2** as mac; interocular **p** mic present. Basomedian and basolateral labial fields with **a1**–**5** smooth, **M**, **Me**, **E** and **L1**–**2** ciliate, **r** reduced (Fig. 18B). Ventral chaetotaxy with 29 ciliate chaetae; postlabial **G1**–**4**; **X**, **X4**; **H1**–**4**; **J1**–**2**, chaetae **b.c.** present and a collar row of six mes chaetae distally (Fig. 18B). Prelabral chaetae ciliate. Labral chaetae smooth, no modifications. Labial papilla **E** with **l.p**. finger-shaped and surpassing the base of apical appendage. Labial proximal chaetae smooth (**an1**–**3**, **p2**–**3**) and subequal in length (Fig. 18B). Maxillary palp with **t.a.** smooth and 1.17× larger than **b.c**.

Thorax dorsal chaetotaxy (Fig. 19A). Th II **a**, **m**, **p** series with two mic (**a1**–**2**), one mac (**a5**), three mic (**m1**–**2**, **m4**) and four mic (**p4**–**6e**), **p3** complex with four mac, respectively, **al** and **ms** present. Th III **a**, **m**, **p** series with three mic (**a1**–**3**), two mes (**a6**–**7**), two mic (**m4**–**6p**), four mes (**m6**–**6e**, **m7**–**7e**), four mic (**p1**–**3**, **p6**) respectively. Ratio Th II: III = 1.10–1.31: 1 (n = 4), holotype = 1.10: 1.

Abdomen dorsal chaetotaxy (Fig. 19B, C). Abd I **a**, **m** series with one (**a5**) and six (**m2**–**6e**) mic respectively, **ms** present. Abd II **a, m**, **p** series with two mic (**a6**–**7**), two mac (**m3**, **m5**), three mic (**p5**–**7**) respectively, **el** mic and **as** present; **a5** and **m2** bothriotricha surrounded by five and four fan-shaped chaetae respectively. Abd III **a**, **m**, **p** series with one mic (**a7**), three fan-shaped chaetae (**a2**–**3**, **a6**), two mic (**m7i**–**7**), three mac (**m3**, **am6**, **pm6**), three mic (**p6e**, **p7i–7**), one mac (**p6**) chaetae respectively; **a5**, **m2** and **m5** bothriotricha with six, two and three fan-shaped chaetae respectively, **as** sens elongated, **ms** present. Abd IV **A**–**Fe** series with three mic (**A1**, **A6**, **Ae1**), two mac (**A3**, **A5**), one mic (**B1**), one mes (**B6**), two mac (**B4**–**5**), four mic (**C1**–**4**), three mic (**T1**, **T5**–**6**), one mes (**T7**), five mic (**D1**–**3**, **De3**), one mes (**D3p**), one mic (**E4p2**), one mes (**E4p**), three mac (**E1**–**3**), one mic (**Ee12**), two mes (**Ee10**–**11**), one mac (**Ee9**), one mic (**F1**), two mes (**F3–3p**), one mac (**F2**), one mic (**Fe2**), three mes (**Fe3**–**5**) chaetae, respectively; **T2**, **T4** and **E4** bothriotricha surrounded by four and two (**T3**) fan-shaped chaetae respectively; **ps** and **as** present, and at least six supernumerary sens with uncertain homology *‘s’* (Fig. 8A); Abd. IV posteriorly with nine **psp**. Abd V **a**, **m**, **p** series with two mic (**a1**, **a3**), one mes (**a6**), one mac (**a5**), two mes (**m5a**, **m5e**), three mac (**m2**–**3**, **m5**), five mic (**p3a**–**6ae**), one mic (**p6e**) two mes (**ap6**–**pp6**), four mac (**p1**, **p3**–**5**) chaetae, respectively; **as**, **acc**.**p4**–**5** present. Ratio Abd III: IV = 1: 3.46–5.80 (n = 5), holotype = 1: 5.80.

Legs. Trochanteral organ diamond shape with about 23 spine-like chaetae, plus two **psp** one external and one on distal vertex of Omt (Fig. 20A). Unguis outer side with one paired tooth straight and not developed on proximal third; inner lamella wide with four teeth, basal pair subequal, **b.p.** not reaching the **m.t.** apex, **m.t.** just after the distal half, **a.t.** present. Unguiculus with lamellae smooth and lanceolate (**a.i.**, **a.e.**, **p.i.**), except **p.e.** slightly serrate (Fig. 20B); ratio unguis: unguiculus = 1: 1.63–1.84 (n = 5), holotype = 1: 1.79. Tibiotarsal smooth chaetae about 0.8× smaller than unguiculus; tenent hair capitate and about 0.52× smaller unguis outer lamella.

Collophore (Fig. 20C). Anterior side with 13 ciliate, apically acuminate chaetae, seven proximal (thinner); four subdistal and two distal mac; lateral flap with 11 chaetae, five ciliate in the proximal row and six smooth in the distal row.

Furcula. Covered with ciliate chaetae, spine-like chaetae and scales. Manubrial plate with four ciliate chaetae (two inner mac) and three **psp** (Fig. 20D). Dens posterior face with two or more longitudinal rows of spine-like chaetae about 70 external and 30 internal, external spines larger and thinner than internal ones. Mucro with four teeth, ratio width: length = 0.33 (n = 5).

*Etymology.* Species named after Type locality *Morro do Chapeu*.

*Remarks. Trogolaphysa chapelensis* **sp. nov.** resembles *T. jacobyi*, *T. caripensis*, *T. bessoni*, and *T. belizeana* by te absence of eyes (0 + 0 eyes) but is easily distinguished by presenting 4 + 4 mac in Th II **p3** complex (2–3 + 2–3 *T. jacobyi*; 6 + 6 *T. caripensis*; 2 + 2 *T. bessoni*; 2–4 + 2–4 *T. belizeana*), and 9 + 9 **psp** posterior Abd IV (4 + 4T*. belizeana*).

*Trogolaphysa crystallensis* **sp. nov.** Oliveira, Lima & Zeppelini

**Figure 21:**
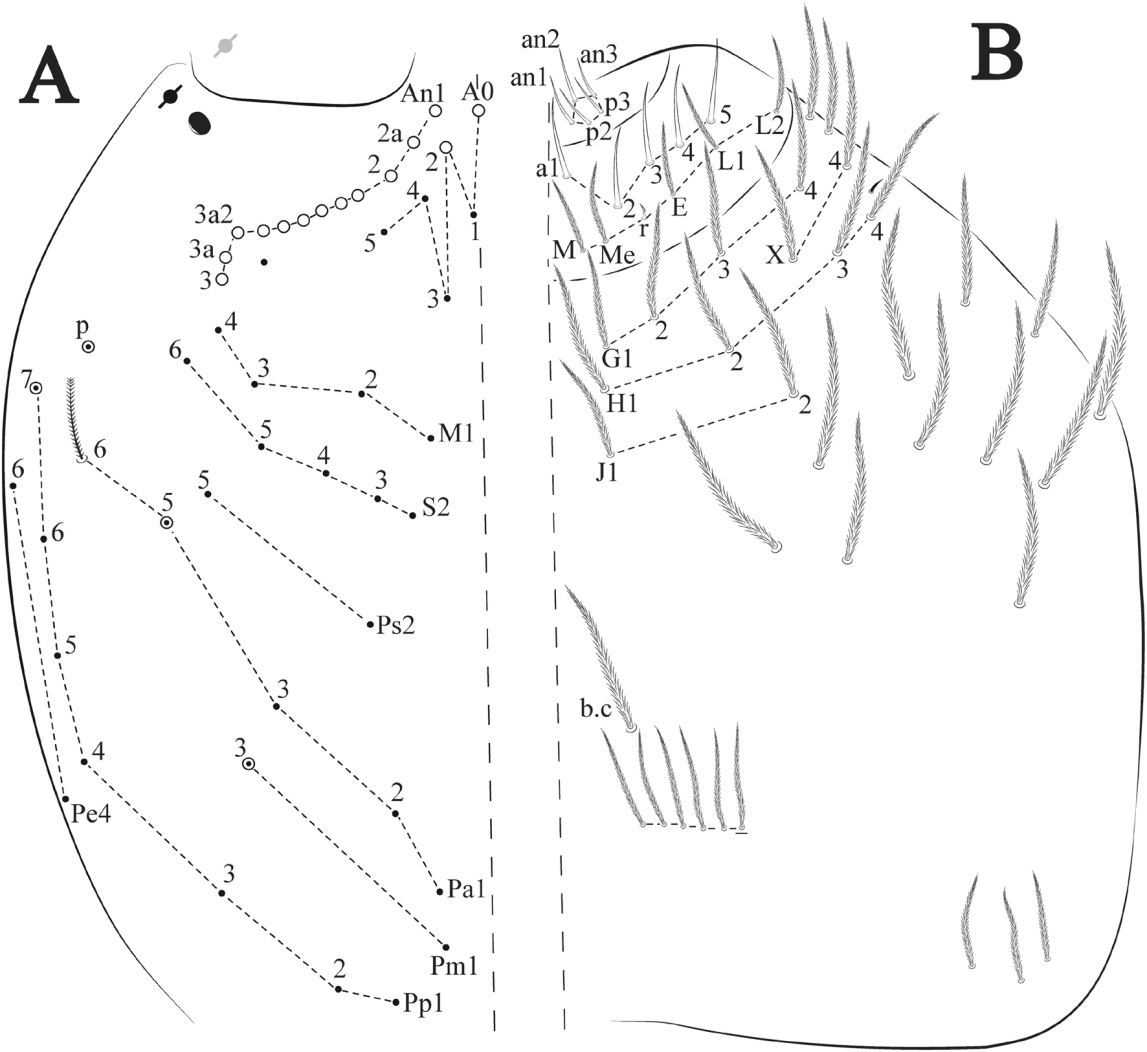
*Trogolaphysa crystallensis* **sp. nov.**: (**A**) Head dorsal chaetotaxy, (**B**) labial proximal chaetae, basomedial and basolateral labial fields and postlabial chaetotaxy. Black cut circle, pseudopore; Gray cut circle pseudopore at the under surface.

**Figure 22:**
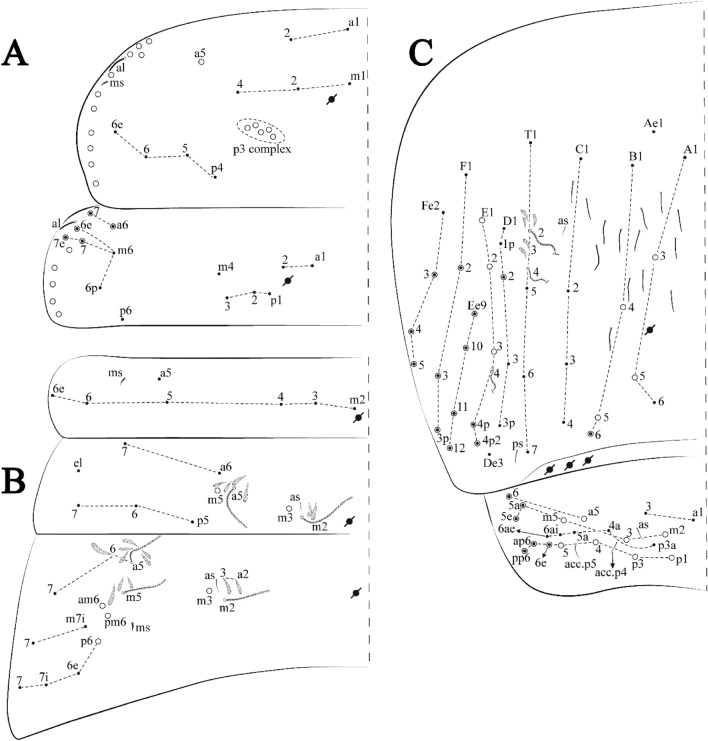
*Trogolaphysa crystallensis* **sp. nov.**: Dorsal chaetotaxy. (**A**) Th II–III, (**B**) Abd I–III; (**C**) Abd IV–V.

**Figure 23:**
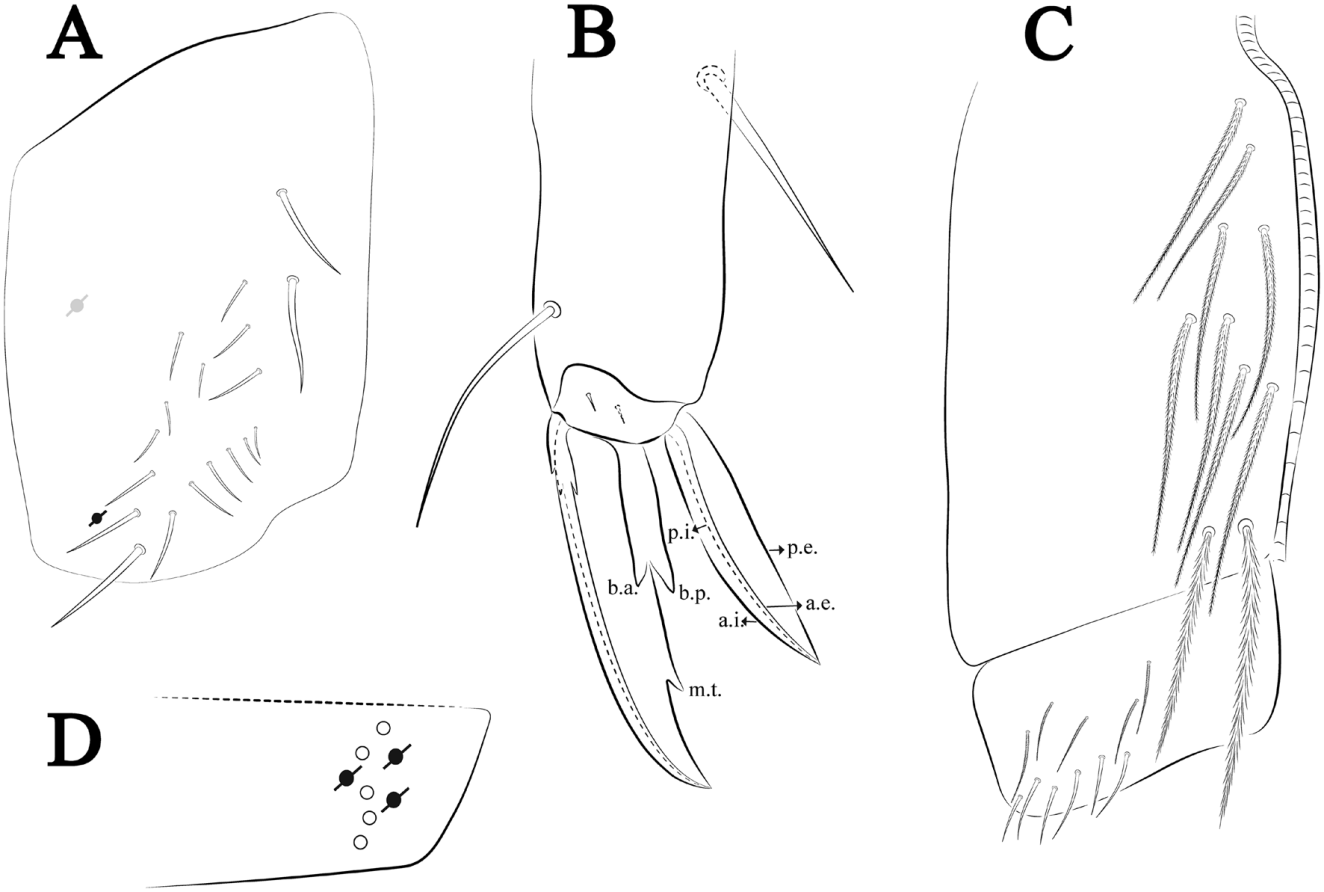
*Trogolaphysa crystallensis* **sp. nov.**: (**A**) Trochanteral organ, (**B**) Distal tibiotarsus and empodial complex III (anterior view), (**C**) Manubrial plate, (**D**) Antero-lateral view of collophore chaetotaxy.

Figures 21, 22 and 23, Tables 1 and 2

*Type material.* Holotype female in slide (16,252/CRFS-UEPB): Brazil, Minas Gerais State, Mariana municipality, cave LOC-0090, next to “Cachoeira Crystal”, 20°20′20.8″S, 43°23′44.3″W, 11–14.xi.2019, Carste team coll. Paratype in slide (16,251/CRFS-UEPB): female, same data as holotype. Paratype in slide (16,254/CRFS-UEPB donated to MNJR): female, same data as holotype. Additional records see S1.

*Description.* Total length (head + trunk) of specimens 1.40–1.68 mm (n = 3), holotype 1.68 mm.

Head. Ratio antennae: trunk = 1: 1.24–2.30 (n = 2), holotype = 1: 1.24; Ant III shorter than Ant II length; Ant segments ratio as I: II, III, IV = 1: 1.72–1.78, 1.58–1.64, 3.11–3.14, holotype = 1: 1.78, 1.64, 3.14. Antennal chaetotaxy (no represented): Ant IV dorsally and ventrally with several short ciliate mic and mac, and finger-shaped sens, dorsally with about three rod sens on longitudinal row, ventrally with one subapical-organ and several wrinkly sens (Fig. 4A); Ant III dorsally and ventrally with several short ciliate mic and mac, and finger-shaped sens, dorsally without modified sens, ventrally with one apical **psp**, about three wrinkly sens on external longitudinal row, apical organ with two rod sens, and one finger-shaped sens (Fig. 4A); Ant II dorsally and ventrally with several short ciliate mic and mac, dorsally with three sub-apical finger-shaped sens and one wrinkly sens, ventrally with one apical **psp** (Fig. 4A); and Ant I dorsally and ventrally with several short ciliate mic and mac, dorsally with three basal spine-like sens, ventrally with four basal spine-like sens, about three smooth mic and several finger-shaped sens (Fig. 4A). Eyes 0 + 0. Head dorsal chaetotaxy (Fig. 21A) with 12–13 **An** (**An1a**–**3**), six **A** (**A0**–**5**), four **M** (**M1**–**4**), five **S** (**S2**–**6**), two **Ps** (**Ps2**, **Ps5**), four **Pa** (**Pa1**–**5**), two **Pm** (**Pm1**, **Pm3**), seven **Pp** (**Pp1**–**7**), and two **Pe** (**Pe4**, **Pe6**) chaetae; **Pa5**, **Pm3** and **Pp7** as mes, **An1a**–**3a**, **A0** and **A2** as mac; interocular **p** mes present. Basomedian and basolateral labial fields with **a1**–**5** smooth, **M**, **Me**, **E** and **L1**–**2** ciliate, **r** reduced (Fig. 21B). Ventral chaetotaxy with 33–35 ciliate chaetae and one reduced lateral spine; postlabial **G1**–**4**; **X**, **X4**; **H1**–**4**; **J1**–**2**, chaetae **b.c.** present and a collar row of four to six mes chaetae distally (Fig. 21B). Prelabral chaetae ciliate. Labral chaetae smooth, no modifications. Labial papilla **E** with **l.p.** finger-shaped and surpassing the base of apical appendage. Labial proximal chaetae smooth (**an1**–**3**, **p2**–**3**) and subequal in length (Fig. 21B). Maxillary palp with **t.a.** smooth and 1.43 × larger than **b.c**.

Thorax dorsal chaetotaxy (Fig. 22A). Th II **a**, **m**, **p** series with two mic (**a1**–**2**), one mac (**a5**), three mic (**m1**–**2**, **m4**) and four mic (**p4**–**6e**), **p3** complex with five mac, respectively, **al** and **ms** present. Th III **a**, **m**, **p** series with two mic (**a1**–**2**), two mes (**a6**–**7**), theree mic (**m4**, **m6**–**6p**), three mes (**m6e**, **m7**–**7e**), four mic (**p1**–**3**, **p6**) respectively. Ratio Th II: III = 1.05–1.27: 1 (n = 3), holotype = 1.05: 1.

Abdomen dorsal chaetotaxy (Fig. 22B, C). Abd I **a**, **m** series with one (**a5**) and six (**m2**–**6e**) mic respectively, **ms** present. Abd II **a, m**, **p** series with two mic (**a6**–**7**), two mac (**m3**, **m5**), three mic (**p5**–**7**) respectively, **el** mic and **as** present; **a5** and **m2** bothriotricha surrounded by four and two fan-shaped chaetae respectively. Abd III **a**, **m**, **p** series with one mic (**a7**), three fan-shaped chaetae (**a2**–**3**, **a6**), two mic (**m7i**–**7**), three mac (**m3**, **am6**, **pm6**), three mic (**p6e**, **p7i–7**), one mac (**p6**) chaetae respectively, **a5**, **m2** and **m5** bothriotricha with six, two and three fan-shaped chaetae respectively, **as** sens elongated, **ms** present. Abd IV **A**–**Fe** series with three mic (**A1**, **A6, Ae1**), two mac (**A3**, **A5**), one mic (**B1**), one mes (**B6**), two mac (**B4**–**5**), four mic (**C1**–**4**), three mic (**T1**, **T5**–**7**), five mic (**D1**–**1p, D3**–**3p**, **De3**), one mes (**D2**), two mes (**E4p**–**4p2**), three mac (**E1**–**3**), four mes (**Ee9**–**12**), one mic (**F1**), three mes (**F2**–**3p**), one mic (**Fe2**), three mes (**Fe3**–**5**) chaetae, respectively; **T2**, **T4** and **E4** bothriotricha surrounded by three and two (**T3**) fan-shaped chaetae respectively; **ps** and **as** present, and at least 14 supernumerary sens with uncertain homology *‘s’*(Fig. 8A); Abd. IV posteriorly with three **psp**. Abd V **a**, **m**, **p** series with two mic (**a1**, **a3**), one mes (**a6**), one mac (**a5**), two mes (**m5a**, **m5e**), three mac (**m2**–**3**, **m5**), five mic (**p3a**–**P6ae**), three mes (**p6e**–**pp6**), four mac (**p1**, **p3**–**5**) chaetae, respectively; **as** and **acc**.**p4**–**5** present. Ratio Abd III: IV = 1: 4.06–4.51 (n = 3), holotype = 1: 4.51.

Legs. Trochanteral organ diamond shape with about 18 spine-like chaetae, plus two **psp** one external and one on distal vertex of Omt (Fig. 23A). Unguis outer side with one paired tooth straight and not developed on proximal third; inner lamella wide with three teeth, basal pair subequal, **b.p.** little larger, but not reaching the **m.t.** apex, **m.t.** just after the distal half, **a.t.** absent. Unguiculus with all lamellae smooth and lanceolate (**a.i.**, **a.e.**, **p.i.**, **p.e.**) (Fig. 23B); ratio unguis: unguiculus = 1.48–1.79: 1 (n = 3), holotype = 1.48: 1. Tibiotarsal smooth chaetae about 0.8× smaller than unguiculus; tenent hair acuminate and about 0.5× smaller than unguis outer lamella.

Collophore (Fig. 23C). Anterior side with 10 ciliate, apically acuminate chaetae, six proximal, two subdistal (as mes) and two distal mac; lateral flap with 11 chaetae, five ciliate in the proximal row and six smooth in the distal row.

Furcula. Covered with ciliate chaetae, spine-like chaetae and scales. Manubrial plate with four ciliate chaetae (two inner mac) and three **psp** (Fig. 23D). Dens posterior face with two or more longitudinal rows of spine-like chaetae about 60 external and 28 internal, external spines larger and thinner than internal ones. Mucro with four teeth, ratio width: length = 0.31 (holotype).

*Etymology.* Species named after Type locality *Cachoeira Crystal* (Portuguese for Crystal falls).

*Remarks. Trogolaphysa crystallensis* **sp. nov.** resembles *T. barroca* **sp. nov.**, *T. gisbertae* **sp. nov.**, *T. sotoadamesi* **sp. nov.**, *T. triocelata* and *T. zampauloi* **sp. nov.** by the absence of eyes (0 + 0 eyes) (*T. triocelata* 3 + 3 and *T. zampauloi* **sp. nov.** eventually 4 + 4), Th II with 5 + 5 mac, and Th III without mac. Can be distinguished from *T. barroca* **sp. nov.**, *T. gisbertae* **sp. nov.**, and *T. sotoadamesi* **sp. nov.** by the Abd IV with 4 + 4 central mac (A3, A5, B4–5); *T. barroca* **sp. nov.**, *T. gisbertae* **sp. nov.**, and *T. triocelata*, with 3 + 3 and *T. sotoadamesi* **sp. nov.** 2 + 2 central mac on Abd IV. Finally, the new species differentiates from *T. zampauloi* **sp. nov.** by unpaired lamella of unguis with one tooth, Omt with about 18 spine-like chaetae, dens external row with about 58 spines on *T. crystallensis* **sp. nov.** and unpaired lamella of unguis with two teeth, Omt with about 26 spine-like chaetae, dens external row with about 30 spines on *T. zampauloi* **sp. nov.**

*Trogolaphysa sotoadamesi* **sp. nov.** Ferreira, Oliveira & Zeppelini

**Figure 24:**
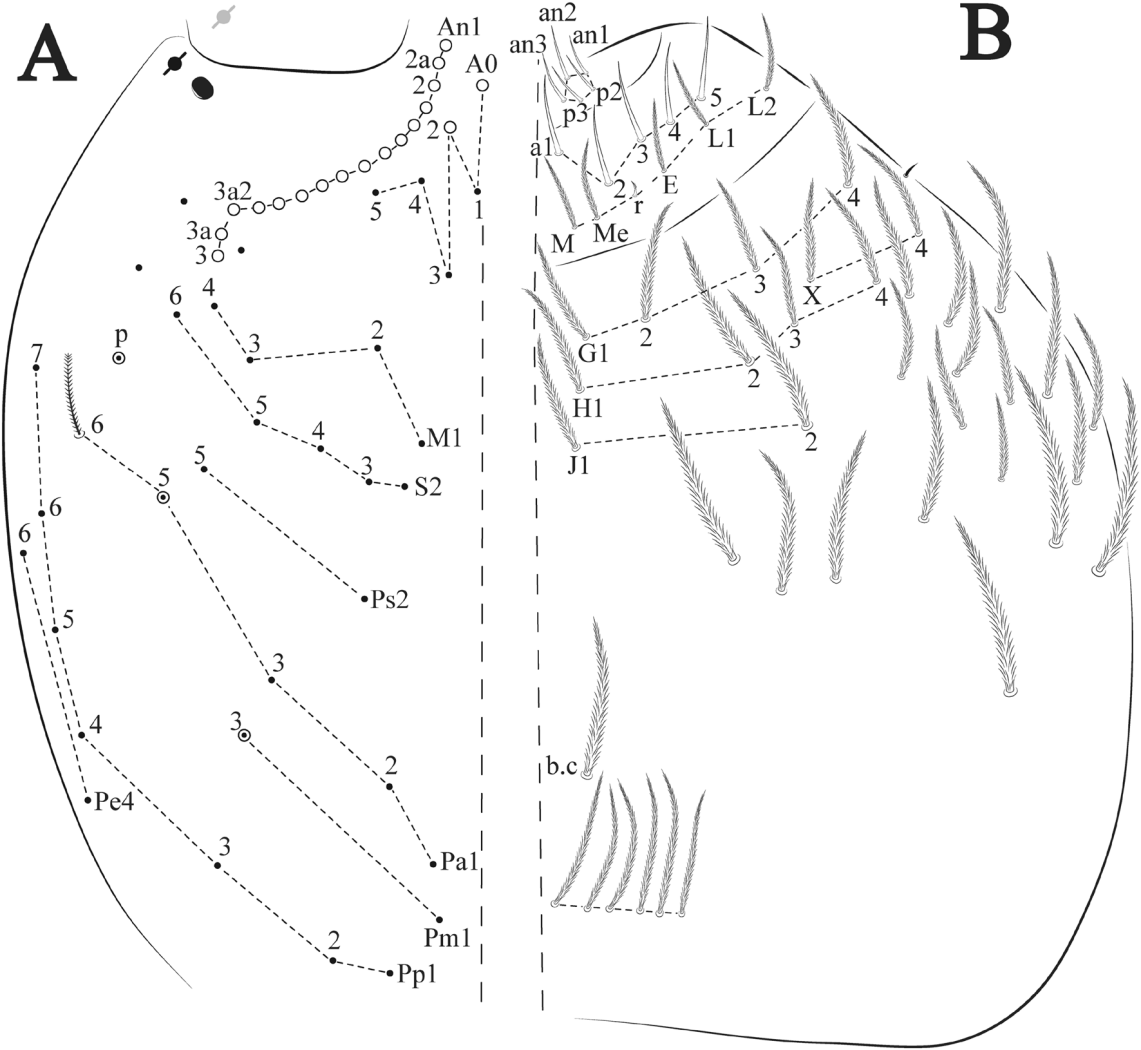
*Trogolaphysa sotoadamesi* **sp. nov.**: (**A**) Head dorsal chaetotaxy, (**B**) labial proximal chaetae, basomedial and basolateral labial fields and postlabial chaetotaxy. Black cut circle, pseudopore, Gray cut circle pseudopore at the under surface.

**Figure 25:**
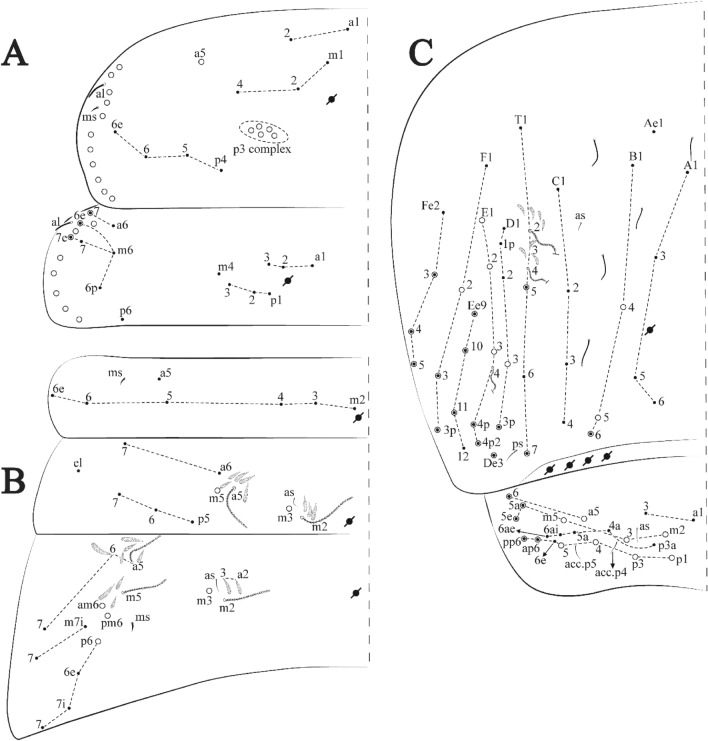
*Trogolaphysa sotoadamesi* **sp. nov.**: Dorsal chaetotaxy. (**A**) Th II–III, (**B**) Abd I–III, (**C**) Abd IV–V.

**Figure 26:**
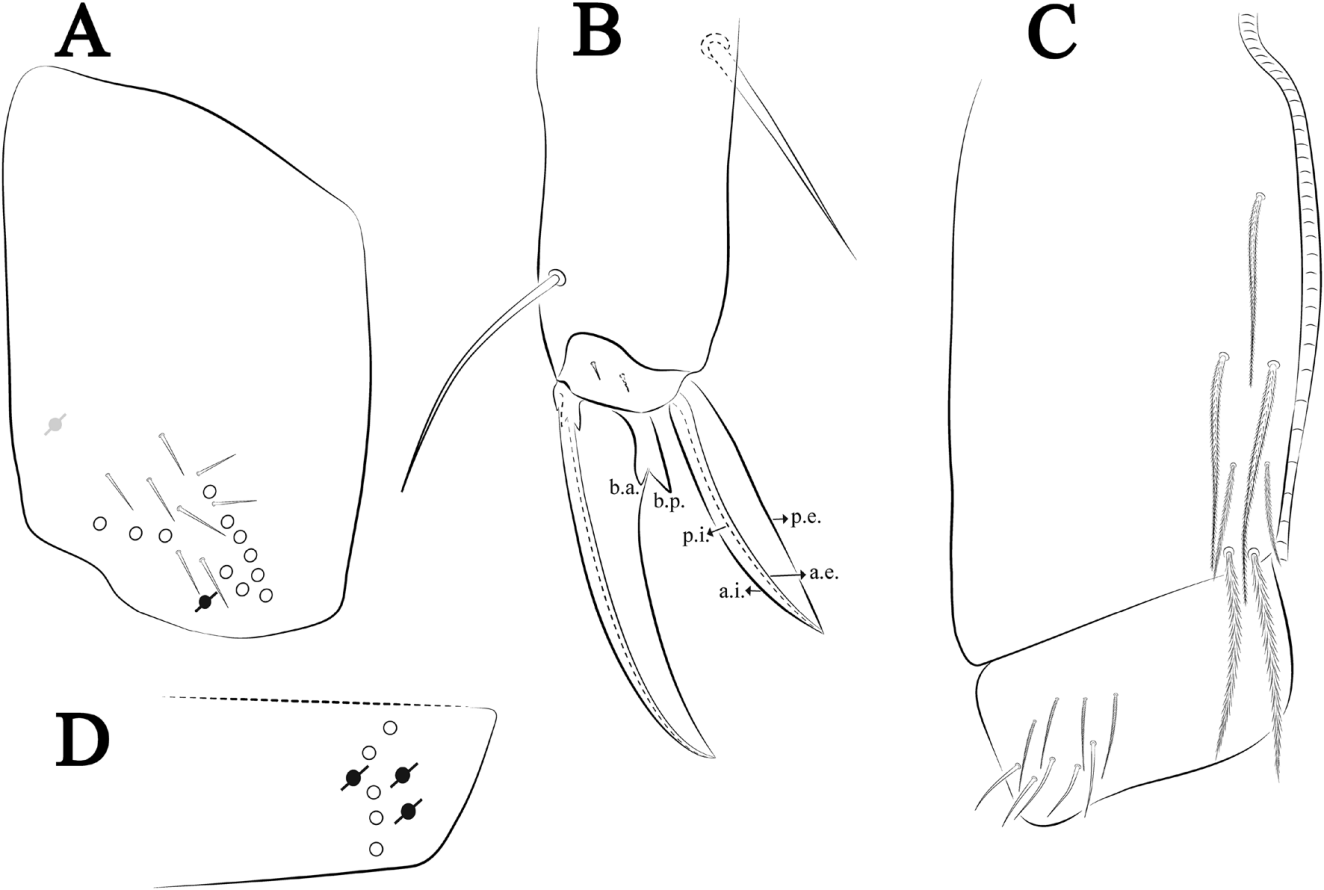
*Trogolaphysa sotoadamesi* **sp. nov.**: (**A**) Trochanteral organ, ((**B**) Distal tibiotarsus and empodial complex III (anterior view), (**C**) Manubrial plate, (**D**) Antero-lateral view of collophore chaetotaxy.

Figures 24, 25 and 26, Tables 1 and 2

*Type material.* Holotype male in slide (13,162/CRFS-UEPB): Brazil, Minas Gerais State, Mariana municipality, cave ALEA 0003, next to “Mina de Alegria”, 20°09′6.81″S, 43°29′13.6″W, 07.ii.2018, Bioespeloeo team coll. Paratypes in slides (13,146, 13,153/CRFS-UEPB): 2 females, same data as holotype, except 12.vi.2017. Paratype in slide (13,173, 13,186/CRFS-UEPB donated to MNJR): 2 females, same data as holotype, except 09.vi.2017. Additional records see S1.

*Description.* Total length (head + trunk) of specimens1.50–1.81 mm (n = 5), holotype 1.50 mm.

Head. Ratio antennae: trunk = 1: 1.26–1.45 (n = 3), holotype = 1: 1.38; Ant III shorter than Ant II; Ant segments ratio, I: II, III, IV = 1: 1.78–2.76, 1.52–2.22, 2.61–3.90, holotype = 1: 2.04, 1.68, 3.16. Antennal chaetotaxy (no represented): Ant IV dorsally and ventrally with several short ciliate mic and mac, and finger-shaped sens, dorsally with a longitudinal row with about three rod sens, ventrally with one subapical-organ and with several wrinkly sens (Fig. 4A); Ant III dorsally and ventrally with several short ciliate mic and mac, and finger-shaped sens, dorsally without modified sens, ventrally with one apical **psp**, several wrinkly sens, apical organ with two coffee bean-like sens, one rod sens and one finger-shaped sens (Fig. 4A); Ant II dorsally and ventrally with several short ciliate mic and mac, dorsally with two sub-apical rod sens and two finger-shaped sens, ventrally with one apical **psp** and several finger-shaped sens (Fig. 4A); and Ant I dorsally and ventrally with several short ciliate mic and mac, dorsally with three basal spine-like sens, ventrally with about seven basal spine-like sens, about three smooth mic and several finger-shaped sens (Fig. 3A). Eyes 0 + 0. Head dorsal chaetotaxy (Fig. 24A) with 16 **An** (**An1a**–**3**), six **A** (**A0**–**5**), four **M** (**M1**–**4**), five **S** (**S2**–**6**), two **Ps** (**Ps2**, **Ps5**), four **Pa** (**Pa1**–**5**), two **Pm** (**Pm1**, **Pm3**), seven **Pp** (**Pp1**–**7**), and two **Pe** (**Pe4**, **Pe6**) chaetae; **Pm3** as mes (rarely mic), **Pa5** as mes, **An1a**–**3a**, **A0** and **A2** as mac; interocular **p** mes present. Basomedian and basolateral labial fields with **a1**–**5** smooth, **M**, **Me**, **E** and **L1**–**2** ciliate, **r** reduced (Fig. 24B). Ventral chaetotaxy with 37 ciliate chaetae and one reduced lateral spine; postlabial **G1**–**4**; **X**, **X4**; **H1**–**4**; **J1**–**2**, chaetae **b.c.** present and a collar row of six mes chaetae distally (Fig. 24B). Prelabral chaetae ciliate. Labral chaetae smooth, no modifications. Labial papilla **E** with **l.p**. finger-shaped and surpassing the base of apical appendage. Labial proximal chaetae smooth (**an1**–**3**, **p2**–**p3**) and subequal in length (Fig. 24B). Maxillary palp with **t.a.** smooth and 1.28× larger than **b.c**.

Thorax dorsal chaetotaxy (Fig. 25A). Th II **a**, **m**, **p** series with two mic (**a1**–**2**), one mac (**a5**), three mic (**m1**–**2**, **m4**) and four mic (**p4**–**6e**), **p3** complex with five mac, respectively, **al** and **ms** present. Th III **a**, **m**, **p** series with three mic (**a1**–**3**, **a6**), one mes (**a7**), four mic (**m4**, **m6**–**7**, **m6p**), two mes (**m6e**, **m7e**), four mic (**p1**–**3**, **p6**) respectively. Ratio Th II: III = 1.17–1.52: 1 (n = 5), holotype = 1.03: 1.

Abdomen dorsal chaetotaxy (Fig. 25B, C). Abd I **a**, **m** series with one (**a5**) and six (**m2**–**6e**) mic respectively, **ms** present. Abd II **a**, **m**, **p** series with two mic (**a6**–**7**), two mac (**m3**, **m5**), three mic (**p5**–**7**) respectively, **el** mic and **as** present; **a5** and **m2** bothriotricha surrounded by five and three fan-shaped chaetae respectively. Abd III **a**, **m**, **p** series with one mic (**a7**), three fan-shaped chaetae (**a2**–**3**, **a6**), two mic (**m7i**–**7**), three mac (**m3**, **am6**, **pm6**), three mic (**p6e**, **p7i**–**7**), one mac (**p6**) chaetae respectively; **a5**, **m2** and **m5** bothriotricha with five, two and three fan-shaped chaetae respectively, **as** sens elongated, **ms** present. Abd IV **A**–**Fe** series with five mic (**A1, A3**, **A5**–**6**, **Ae1**), one mic (**B1**), one mes (**B6**), two mac (**B4**–**5**), four mic (**C1**–**4**), two mic (**T1**, **T6**), two mes (**T5, T7**), three mic (**D1**–**2**), two mes (**D3p, De3**), two mes (**E4p–p2**), three mac (**E1**–**3**), one mic (**Ee12**), three mes (**Ee9**–**11**), one mic (**F1**), two mes (**F3**–**3p**), one mac (**F2**), one mic (**Fe2**), three mes (**Fe3**–**5**) chaetae, respectively; **T2**, **T4** and **E4** bothriotricha surrounded by four and three (**T3**) fan-shaped chaetae respectively; **ps** and **as** present, and at least five supernumerary sens with uncertain homology *‘s’* (Fig. 8A); Abd. IV posteriorly with four **psp**. Abd V **a**, **m**, **p** series with two mic (**a1**, **a3**), one mes (**a6**), one mac (**a5**), two mes (**m5a**, **m5e**), three mac (**m2**–**3**, **m5**), five mic (**p3a**–**p6ae**), one mic (**P6e**) two mes (**ap6**–**pp6**), four mac (**p1**, **p3**–**5**) chaetae, respectively; **as**, **acc**.**p4**–**5** present. Ratio Abd III: IV = 1: 5.03–4.42 (n = 5), holotype = 1: 4.42.

Legs. Trochanteral organ triangular shape with about 19–21 spine-like chaetae, plus two **psp** one external and one on distal vertex of Omt (Fig. 26A). Unguis outer side with one paired tooth straight and not developed on proximal third; inner lamella wide with two teeth, basal pair unequal, **b.p.** larger than **b.a.**; **m.t.** and **a.t.** absent. Unguiculus with all lamellae smooth and lanceolate (**a.i.**, **a.e.**, **p.i.**, **p.e.**) (Fig. 26B); ratio unguis: unguiculus = 1: 1.46–1.91 (n = 5), holotype = 1: 1.91. Tibiotarsal smooth chaetae about 0.8 × smaller unguiculus; tenent hair acuminate and about 0.4 × smaller than unguis outer lamella.

Collophore (Fig. 26C). Anterior side with seven ciliate, apically acuminate chaetae, three proximal, two subdistal and two distal mac; lateral flap with nine chaetae, four ciliate in the proximal row and five smooth in the distal row.

Furcula. Covered with ciliate chaetae, spine-like chaetae and scales. Manubrial plate with five ciliate chaetae (two inner mac) and three **psp** (Fig. 26D). Dens posterior face with two or more longitudinal rows of spine-like chaetae about 35 external and 26 internal, external spines larger and thinner than internal ones. Mucro with four teeth, ratio width: length = 0.39 (n = 5).

*Etymology.* Species named after Dr. Felipe N. Soto-Adames for his contribution on Collembola taxonomy and systematics.

*Remarks. Trogolaphysa sotoadamesi* **sp. nov.** resembles *T. barroca* **sp. nov.**, *T. crystallensis* **sp. nov**., *T. gisbertae* **sp. nov.**, *T. zampauloi* **sp. nov.** by 0 + 0 eyes (*T. zampauloi* **sp. nov.** rarely with 4 + 4 eyes), Th II **p3** complex with five mac, Th III without mac, manubrial plate with five ciliate chaetae and mucro with four teeth. The new species *T. sotoadamesi* **sp. nov.** with 2 + 2 central mac on Abd IV differentiates from *T. barroca* **sp. nov.**, *T. gisbertae* **sp. nov.** with 3 + 3, and *T. crystallensis* **sp. nov.**, *T. zampauloi* **sp. nov**. with 4 + 4 central mac.

*Trogolaphysa mariecurieae* **sp. nov.** Ferreira, Oliveira & Zeppelini

**Figure 27:**
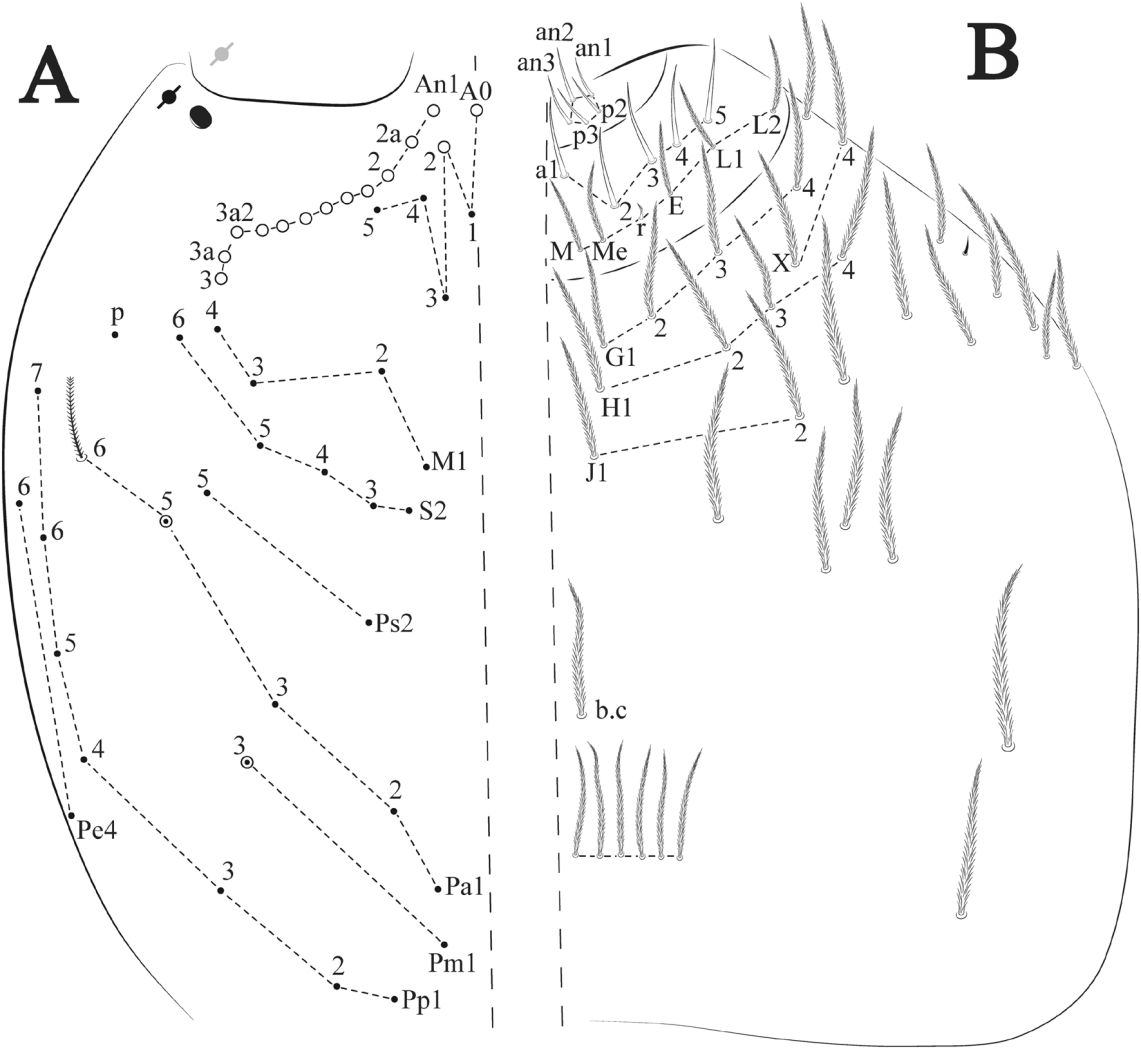
*Trogolaphysa mariecurieae* **sp. nov.**: (**A**) Head dorsal chaetotaxy, (**B**) labial proximal chaetae, basomedial and basolateral labial fields and postlabial chaetotaxy. Black cut circle, pseudopore, Gray cut circle pseudopore at the under surface.

**Figure 28:**
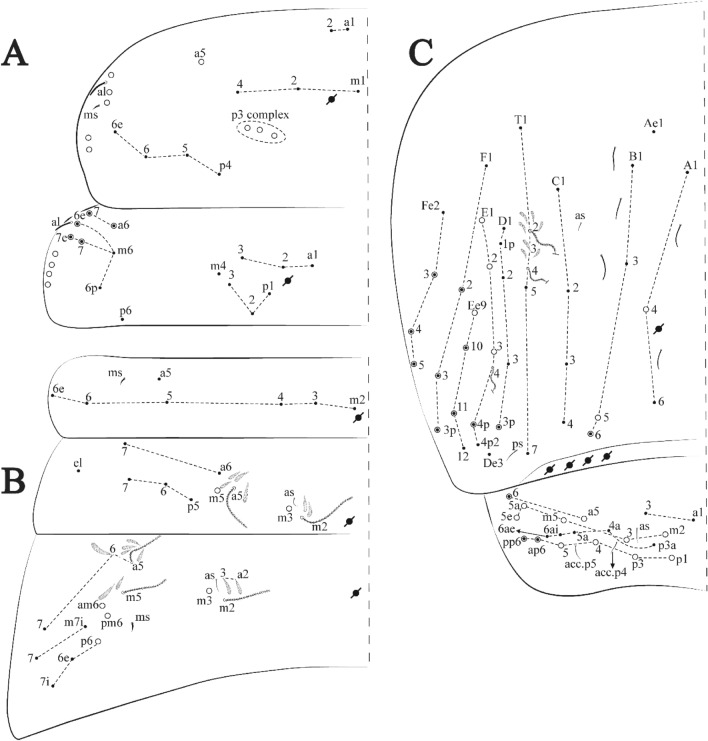
*Trogolaphysa mariecurieae* **sp. nov.**: Dorsal chaetotaxy. (**A**) Th II–III, (**B**) Abd I–III, (**C**) Abd IV–V.

**Figure 29:**
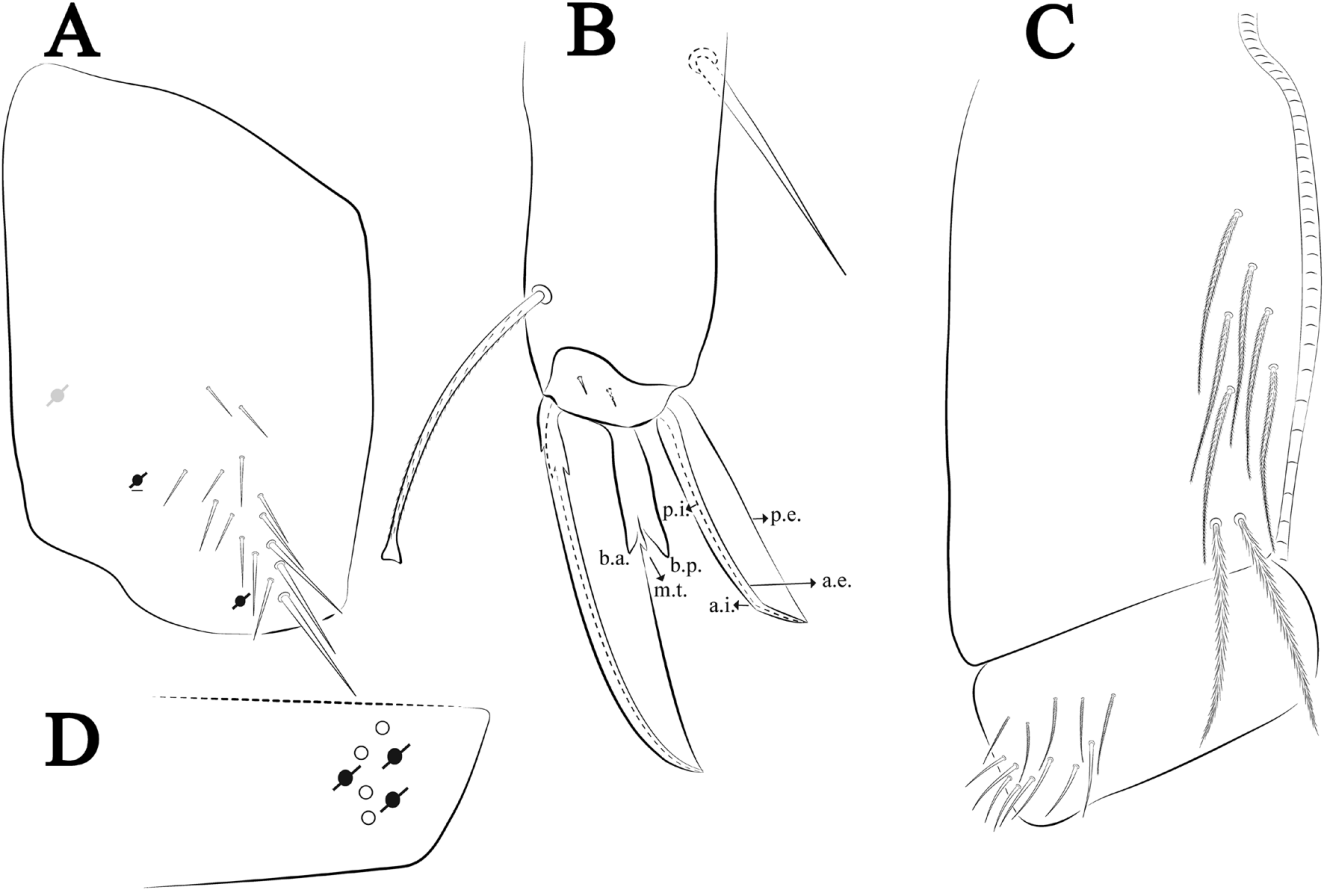
*Trogolaphysa mariecurieae* **sp. nov.**: (**A**) Trochanteral organ, (**B**) Distal tibiotarsus and empodial complex III (anterior view), (**C**) Manubrial plate, (**D**) Antero-lateral view of collophore chaetotaxy.

Figures 27, 28 and 29, Tables 1 and 2

*Type material.* Holotype female in slide (9109/CRFS-UEPB): Brazil, Minas Gerais State, Conceição do Mato Dentro municipality, MSS 10/11, next to “Pico do Soldado” 19°00′23.86″S, 43°23′41.266″W, 11–10.ix.2015, Carste team coll. Paratypes in slides (5888, 5857/CRFS-UEPB): 2 females, same data as holotype, except,19–23.v.2014, Soares et al*.* coll.

Paratype in slide (9222, 10,760/CRFS-UEPB donated to MNJR): 2 females, same data as holotype, except 19°00′18.72″S, 43°23′30.031″W, 14.x.2015 and 18–20.iv.2016. Additional records see S1.

*Description.* Total length (head + trunk) of specimens 1.07–1.49 mm (n = 5), holotype 1.49 mm.

Head. Ratio antennae: trunk = 1: 1.69–1.91 (n = 2), holotype = 1: 1.69; Ant III shorter than Ant II length; Ant segments ratio, I: II, III, IV = 1: 2.00–2.75, 1.69–2.55, 4.02–5.29, holotype = 1: 2.75, 1.69, 4.02. Antennal chaetotaxy (no represented): Ant IV dorsally and ventrally with several short less ciliate mic and mac, and finger-shaped sens, dorsally with one longitudinal row with about four rod sens, ventrally with one subapical-organ and several wrinkly sens (Fig. 4A); Ant III dorsally and ventrally with several short less ciliate mic and mac, and finger-shaped sens, dorsally without modified sens, ventrally with one apical **psp**, apical organ with two rod sens (Fig. 4A); Ant II dorsally and ventrally with several short less ciliate mic and mac, dorsally with five apical rod sens, ventrally with one apical **psp**, about five wrinkly sens on longitudinal external row (Fig. 4A); and Ant I dorsally and ventrally with several short less ciliate mic and mac, dorsally with three basal spine-like sens, ventrally with seven basal spine-like sens, about five smooth mic, and several finger-shaped sens (Fig. 4A). Eyes 0 + 0. Head dorsal chaetotaxy (Fig. 27A) with 12 **An** (**An1a**–**3**), six **A** (**A0**–**5**), four **M** (**M1**–**4**), five **S** (**S2**–**6**), two **Ps** (**Ps2**, **Ps5**), four **Pa** (**Pa1**–**3**, **Pa5**), two **Pm** (**Pm1**, **Pm3**), seven **Pp** (**Pp1**–**7**), and two **Pe** (**Pe4**, **Pe6**) chaetae; **Pm3** and **Pa5** as mes, **An1a**–**3a**, **A0** and **A2** as mac; interocular **p** mic present. Basomedian and basolateral labial fields with **a1**–**5** smooth, **M**, **Me**, **E** and **L1**–**2** ciliate, **r** reduced (Fig. 27B). Ventral chaetotaxy with 34 ciliate chaetae and one reduced lateral spine; postlabial **G1**–**4**; **X**, **X4**; **H1**–**4**; **J1**–**2**, chaetae **b.c.** present and a collar row of six mes chaetae distally (Fig. 27B). Prelabral chaetae ciliate. Labral chaetae smooth, no modifications. Labial papilla **E** with **l.p**. finger-shaped and surpassing the base of apical appendage. Labial proximal chaetae smooth (**an1**–**3**, **p2**–**3**) and subequal in length (Fig. 27B). Maxillary palp with **t.a.** smooth and 1.13× larger than **b.c**.

Thorax dorsal chaetotaxy (Fig. 28A). Th II **a**, **m**, **p** series with two mic (**a1**–**2**), one mac (**a5**), three mic (**m1**–**2**, **m4**) and four mic (**p4**–**6e**), **p3** complex with three mac, respectively, **al** and **ms** present. Th III **a**, **m**, **p** series with three mic (**a1**–**3**), two mes (**a6**–**7**), three mic (**m4**–**m6p**), three mes (**m6e**, **m7**–**7e**), four mic (**p1**–**3**, **p6**) respectively. Ratio Th II: III = 0.85–1.02: 1 (n = 4), holotype = 0.89: 1. Abdomen dorsal chaetotaxy (Fig. 28B, C). Abd I **a**, **m** series with one (**a5**) and six (**m2**–**6e**) mic respectively, **ms** present. Abd II **a, m**, **p** series with two mic (**a6**–**7**), two mac (**m3**, **m5**), three mic (**p5**–**7**) respectively, **el** mic and **as** present; **a5** and **m2** bothriotricha surrounded by four and two fan-shaped chaetae respectively. Abd III **a**, **m**, **p** series with one mic (**a7**), three fan-shaped chaetae (**a2**–**3**, **a6**), two mic (**m7i**–**7**), three mac (**m3**, **am6**, **pm6**), two mic (**p6e**, **p7i**), one mac (**p6**) chaetae respectively; **a5**, **m2** and **m5** bothriotricha with five, two and two fan-shaped chaetae respectively, **as** sens elongated, **ms** present. Abd IV **A**–**Fe** series with three mic (**A1**, **A6, Ae1**), one mac (**A4**), two mic (**B1**–**2**), one mes (**B6**), one mac (**B5**), four mic (**C1**–**4**), three mic (**T1**, **T5**, **T7**), five mic (**D1**–**3**, **De3**), one mes (**D3p**), one mic (**E4p2**), one mes (**E4p**), three mac (**E1**–**3**), one mic (**Ee12**), two mes (**Ee10**–**11**), one mac (**Ee9**), one mic (**F1**), three mes (**F2**–**3p**), one mic (**Fe2**), three mes (**Fe3**–**5**) chaetae, respectively; **T2**, **T4** and **E4** bothriotricha surrounded by four and three (**T3**) fan-shaped chaetae respectively; **ps** and **as** present, and at least five supernumerary sens with uncertain homology *‘s’* (Fig. 8A); Abd. IV posteriorly with four **psp**. Abd V **a**, **m**, **p** series with two mic (**a1**, **a3**), one mes (**a6**), one mac (**a5**), five mac (**m2**–**3**, **m5**–**5e**), five mic (**p3a**–**p6ae**), two mes (**ap6**–**pp6**), four mac (**p1**, **p3**–**5**) chaetae, respectively; **as**, **acc**.**p4**–**5** present. Ratio Abd III: IV = 1: 4.27–5.91 (n = 5), holotype = 1: 5.02.

Legs. Trochanteral organ diamond shape with about 15 spine-like chaetae, plus 2–3 **psp** one external, one on distal vertex and another (present or absent) on top of posterior spines row of Omt (Fig. 29A). Unguis outer side with one paired tooth straight and not developed on proximal third; inner lamella wide with two teeth, basal pair subequal, **b.p.** larger than **b.a.**, inner lamella with unpaired small **m.t.** between **b.a.** and **b.p.** and **a.t.** absent. Unguiculus with all lamellae smooth and truncate (**a.i.**, **a.e.**, **p.i.**, **p.e.**) (Fig. 29B); ratio unguis: unguiculus = 1.50–1.95: 1 (n = 5), holotype = 1.95: 1. Tibiotarsal smooth chaetae about 0.9× smaller than unguiculus; tenent hair slightly capitate and about 0.6× smaller than unguis outer lamella.

Collophore (Fig. 29C). Anterior side with eight ciliate, apically acuminate chaetae, six proximal and two distal mac; lateral flap with 13 chaetae, five ciliate in the proximal row and eight smooth in the distal row.

Furcula. Covered with ciliate chaetae, spine-like chaetae and scales. Manubrial plate with four ciliate chaetae (two inner mac) and three **psp** (Fig. 29D). Dens posterior face with two or more longitudinal rows of spine-like chaetae about 40 external and 22 internal, external spines larger and thinner than internal ones. Mucro with four teeth, ratio width: length = 0.23 (holotype).

*Etymology.* Species named after Dr. Marie Skłodowska-Curie for her enormous contribution to science.

*Remarks. Trogolaphysa mariecurieae* **sp. nov.** resembles *T. bellinii* **sp. nov.** *T. jacobyia* and *T. epitychia* **sp.** **nov.** by the absence of eyes (*T. bellinii* **sp. nov.** rarely with 2 + 2 eyes), Th II **p3** complex with three mac and with one unpaired tooth on inner lamella of unguis. The new species *T. mariecurieae* **sp. nov.** (Abd IV with 2 + 2 mac) differs from *T. jacobyia, T. epitychia* **sp.** **nov.** both with Abd IV 3 + 3, and *T. bellinii* **sp. nov**. with 4 + 4 central mac. *T. mariecurieae* **sp. nov.** and *T. bellinii* **sp. nov.** with capitate tenent hair, in contrast with *T. jacobyia* and *T. epitychia ***sp.** **nov*.*** with acuminated tenant hair.

*Trogolaphysa barroca* **sp. nov.** Brito & Zeppelini

**Figure 30:**
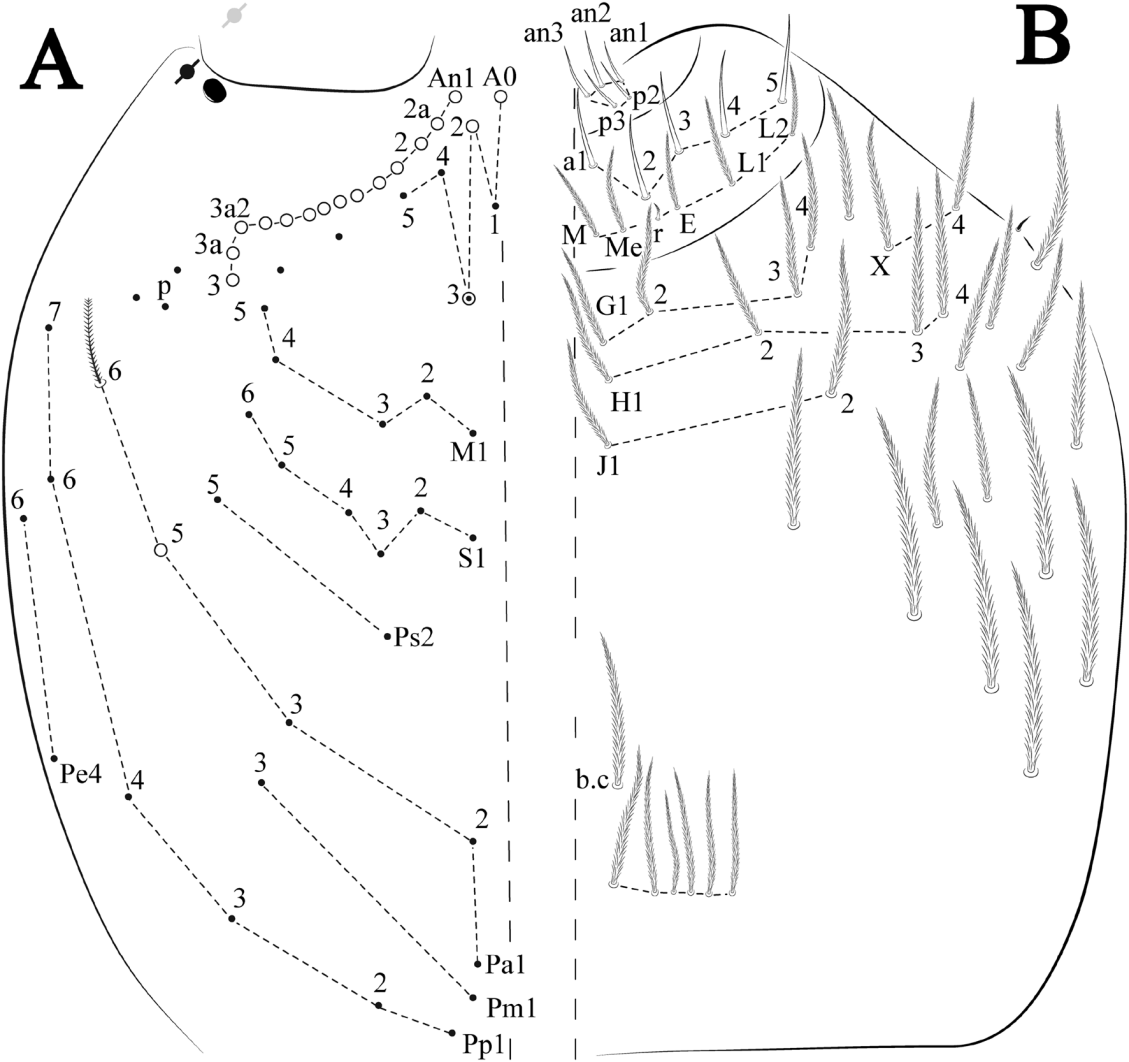
*Trogolaphysa barroca* **sp. nov.**: (**A**) Head dorsal chaetotaxy, (**B**) labial proximal chaetae, basomedial and basolateral labial fields and postlabial chaetotaxy. Black cut circle, pseudopore; Gray cut circle pseudopore at the under surface.

**Figure 31:**
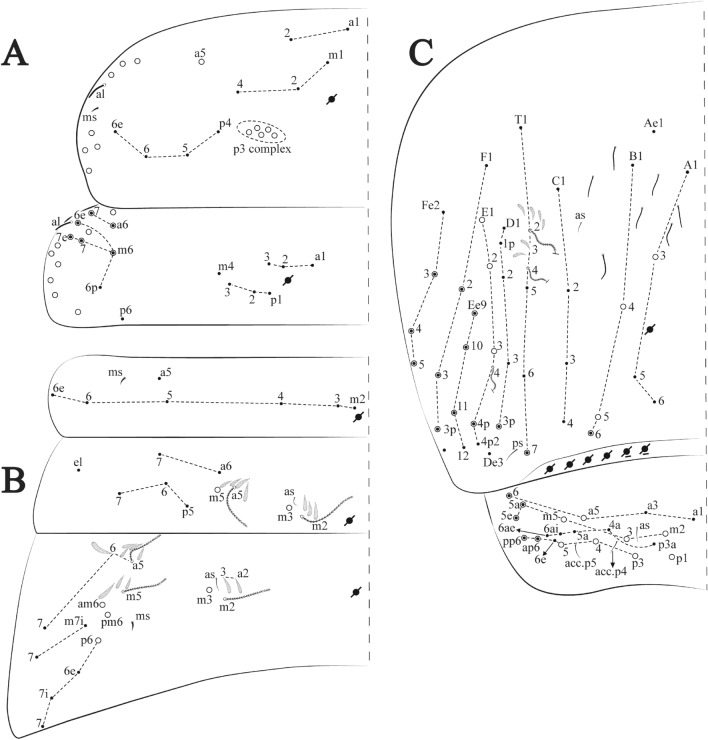
*Trogolaphysa barroca* **sp. nov.**: Dorsal chaetotaxy. (**A**) Th II–III, (**B**) Abd I–III, (**C**) Abd IV–V.

**Figure 32:**
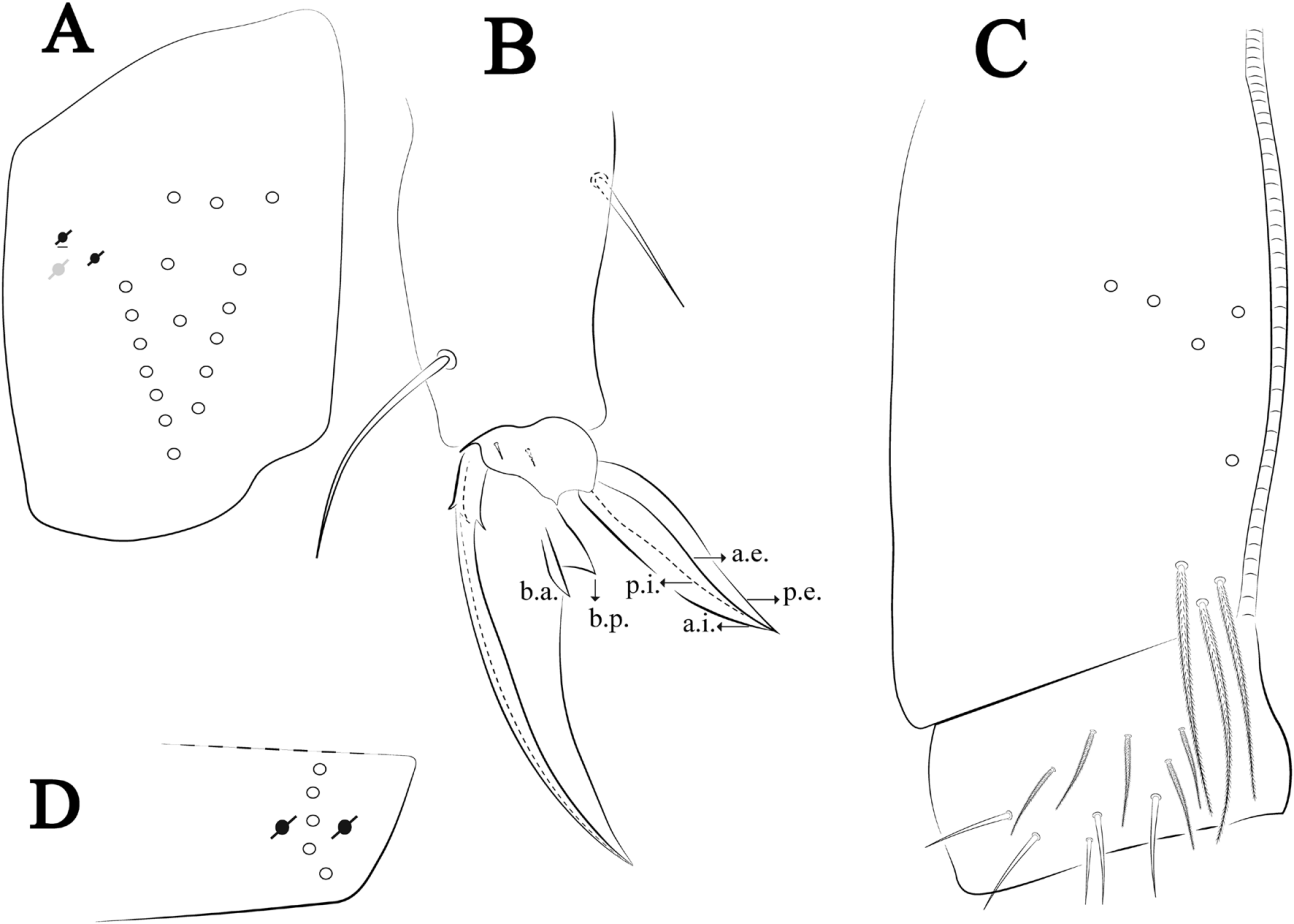
*Trogolaphysa barroca* **sp. nov.**: (**A**) Trochanteral organ, (**B**) Distal tibiotarsus and empodial complex III (anterior view), (**C**) Manubrial plate, (**D**) Antero-lateral view of collophore chaetotaxy.

Figures 30, 31 and 32, Tables 1 and 2

*Type material.* Holotype female in slide (13,167/CRFS-UEPB): Brazil, Minas Gerais State, Mariana municipality, ALFA-0003 cave, 20°09′06.8″S, 43°29′13.6″W, 07–27.ii.2018, Bioespeleo team coll. Paratype in slide (13,150/CRFS-UEPB): 1 female, same data as holotype, except 12.vi.2017. Paratype in slide (13,158/CRFS-UEPB donated to MNJR): 1 female, same data as holotype. Paratype in slide (13,197/CRFS-UEPB): 1 female, Brazil, Minas Gerais State, Mariana municipality, ALEA-0004 cave, 20°09′00.0″S, 43°29′11.8″W, 07.ii.2018, Bioespeleo team coll. Paratype in slide (13,203/CRFS-UEPB): 1 female, Brazil, Minas Gerais State, Mariana municipality, ALEA-0002 cave, 20°08′56.5″S, 43°29′09.8″W, 27.ii.2018, Bioespeleo team coll. Additional records see S1.

*Description.* Total length (head + trunk) of specimens 1.70–2.13 mm (n = 5), holotype 1.81 mm.

Head. Ratio antennae: trunk = 1: 1.27–1.60 (n = 3), holotype = 1: 1.27; Ant III shorter than Ant II; Ant segments ratio as, I: II, III, IV = 1: 1.90–2.41, 1.64–2.02, 2.69–3.64, holotype = 1: 1.90, 1.67, 2.69. Antennal chaetotaxy (no represented): Ant IV dorsally and ventrally with several short less ciliate mic and mac, and finger-shaped sens, dorsally with about four rod sens on longitudinal row, ventrally with one subapical-organ and several wrinkly sens (Fig. 4A); Ant III dorsally and ventrally with several short less ciliate mic and mac, and finger-shaped sens, dorsally without modified sens, ventrally with one apical **psp**, about nine wrinkly sens on external longitudinal row, apical organ with one finger-shaped sens, two coffee bean-like sens, and one rod sens (Fig. 4A); Ant II dorsally and ventrally with several short less ciliate mic and mac, dorsally with two sub-apical finger-shaped sens and two subapical rod sens, ventrally with one apical **psp**, and several wrinkly sens on longitudinal external row (Fig. 4A); and Ant I dorsally and ventrally with several short less ciliate mic and mac, dorsally with three basal spine-like sens, ventrally with about five basal spine-like sens, about five smooth mic and several finger-shaped sens (Fig. 4A). Eyes 0 + 0. Head dorsal chaetotaxy (Fig. 30A) with 14–15 **An** (**An1a**–**3**), six **A** (**A0**–**5**), five **M** (**M1**–**5**), six **S** (**S1**–**6**), two **Ps** (**Ps2**, **Ps5**), four **Pa** (**Pa1**–**3**, **Pa5**), two **Pm** (**Pm1**, **Pm3**), seven **Pp** (**Pp1**–**7**), and two **Pe** (**Pe4**, **Pe6**) chaetae; **Pm3** as mic, **A3** as mes, **An1a**–**3**, **A0**, **A2** and **Pa5** as mac; interocular **p** mic present. Basomedian and basolateral labial fields with **a1**–**5** smooth, **M**, **Me**, **E** and **L1**–**2** ciliate, **r** reduced (Fig. 30B). Ventral chaetotaxy with 33 ciliate chaetae and one reduced lateral spine; postlabial **G1**–**4**; **X**, **X4**; **H1**–**4**; **J1**–**2**, chaetae **b.c.** present and a collar row of five to six mes chaetae distally (Fig. 30B). Prelabral chaetae weakly ciliate. Labral chaetae smooth, no modifications. Labial papilla **E** with **l.p.** finger-shaped and subequal the base of apical appendage. Labial proximal chaetae smooth (**an1**–**3**, **p2**–**3**), and subequal in length (Fig. 30B). Maxillary palp with **t.a.** smooth and 1.14 × larger than **b.c.**

Thorax dorsal chaetotaxy (Fig. 31A). Th II **a**, **m**, **p** series with two mic (**a1**–**2**), one mac (**a5**), three mic (**m1**–**2**, **m4**) and four mic (**p4**–**6e**), **p3** complex with five mac, respectively, **al** and **ms** present. Th III **a**, **m**, **p** series with three mic (**a1**–**3**), two mes (**a6**–**7**), two mic (**m4**, **m6p**), four mes (**m6**–**6e**, **m7**–**7e**), and four mic (**p1**–**3**, **p6**), respectively. Ratio Th II: III = 1.11–1.35: 1 (n = 5), holotype = 1.29: 1.

Abdomen dorsal chaetotaxy (Fig. 31B, C). Abd I **a**, **m** series with one (**a5**) and six (**m2**–**6e**) mic, respectively, **ms** present. Abd II **a**, **m**, **p** series with two mic (**a6**–**7**), two mac (**m3**, **m5**), three mic (**p5**–**7**) respectively, **el** mic and **as** present; **a5** and **m2** bothriotricha surrounded by four and three fan-shaped chaetae, respectively. Abd III **a**, **m**, **p** series with one mic (**a7**), three fan-shaped chaetae (**a2**–**3**, **a6**), two mic (**m7i**–**7**), three mac (**m3**, **am6**, **pm6**), three mic (**p6e**, **p7i**–**7**), one mac (**p6**) chaetae, respectively; **a5**, **m2** and **m5** bothriotricha with six, two and three fan-shaped chaetae, respectively; **as** sens elongated, **ms** present. Abd IV **A**–**Fe** series with four mic (**A1**, **A5**–**6, Ae1**), one mac (**A3**), one mic (**B1**), one mes (**B6**), two mac (**B4**–**5**), four mic (**C1**–**4**), three mic (**T1**, **T5**–**6**), one mes (**T7**), five mic (**D1**–**3**, **De3**), one mes (**D3p**), one mic (**E4p2**), one mes (**E4p**), three mac (**E1**–**3**), one mic (**Ee12**), three mes (**Ee9**–**11**), one mic (**F1**), three mes (**F2**–**3p**), one mic (**Fe2**), three mes (**Fe3**–**5**) chaetae, respectively; **T2**, **T4** and **E4** bothriotricha surrounded by four and two (**T3**) fan-shaped chaetae, respectively; **ps** and **as** present, and at least seven supernumerary sens with uncertain homology *‘s’* (Fig. 8A); Abd. IV posteriorly with four to six **psp**. Abd V **a**, **m**, **p** series with two mic (**a1**, **a3**), one mes (**a6**), one mac (**a5**), two mes (**m5a**–**5e**), three mac (**m2**–**3**, **m5**), five mic (**p3a**–**6ae**), one mic (**p6e**), two mes (**ap6**, **pp6**), four mac (**p1**, **p3**–**5**) chaetae, respectively; **as** and **acc.p4**–**5** present. Ratio Abd III: IV = 1: 3.38–5.55 (n = 5), holotype = 1: 5.27.

Legs. Trochanteral organ diamond shape with about 16–21 spine-like chaetae, plus 2–3 **psp** one external, and two (one of them present or absent) on top of posterior spines row of Omt (Fig. 32A). Unguis outer side with one paired tooth straight and not developed on proximal third; inner lamella wide with two teeth, basal pair subequal; **b.p.** little larger than **b.a.**, **m.t.** and **a.t.** absent. Unguiculus with all lamellae smooth and lanceolate (**a.i.**, **a.e.**, **p.i.**, **p.e.**) (Fig. 32B); ratio unguis: unguiculus = 1.53–1.67: 1 (n = 5), holotype = 1.61: 1. Tibiotarsal smooth chaetae about 0.61 × smaller than unguiculus; tenent hair acuminate and about 0.4 × smaller than unguis outer lamella.

Collophore (Fig. 32C). Anterior side with eight ciliate, apically acuminate chaetae, four proximal (thinner), one subdistal and three distal mac; lateral flap with 10 chaetae, five ciliate in the proximal row and five smooth in the distal row.

Furcula. Covered with ciliate chaetae, spine-like chaetae and scales. Manubrial plate with five ciliate chaetae (three inner mac) and three **psp** (Fig. 32D). Dens posterior face with two or more longitudinal rows of spines-like chaetae about 22 external and 37–39 internal, external spines larger and thinner than internal ones. Mucro with four teeth, ratio width: length = 0.33 (holotype).

*Etymology.* Refers to the Baroque art (which is “barroco” noun, in Portuguese) of Mariana, Minas Gerais, type locality.

*Remarks. Trogolaphysa barroca* **sp. nov.** resembles *T. formosensis* by head **Pm3** mic (mac in *T. piracurucaensis*, *T. gisbertae* **sp. nov.** and *T. dandarae* **sp. nov.**; mes in *T. ernesti*, *T. sotoadamesi* **sp. nov.** and *T. mariecurieae* **sp. nov.**); 3 + 3 head dorsal mac like *T. ernesti*, although in the new species it is as **A0**, **A2** and **Pa5**, and in *T. ernesti* is **A0**, **A2**–**3**; unguis **m.t.** and **a.t.** teeth absent like *T. sotoadamesi* **sp. nov.** and *T. dandarae* **sp. nov.** (present in *T. bellini* **sp. nov.**, *T. lacerta* **sp. nov.** and *T. chapelensis* **sp. nov.**).

*Trogolaphysa epitychia* **sp. nov.** Oliveira, Lima & Zeppelini

**Figure 33:**
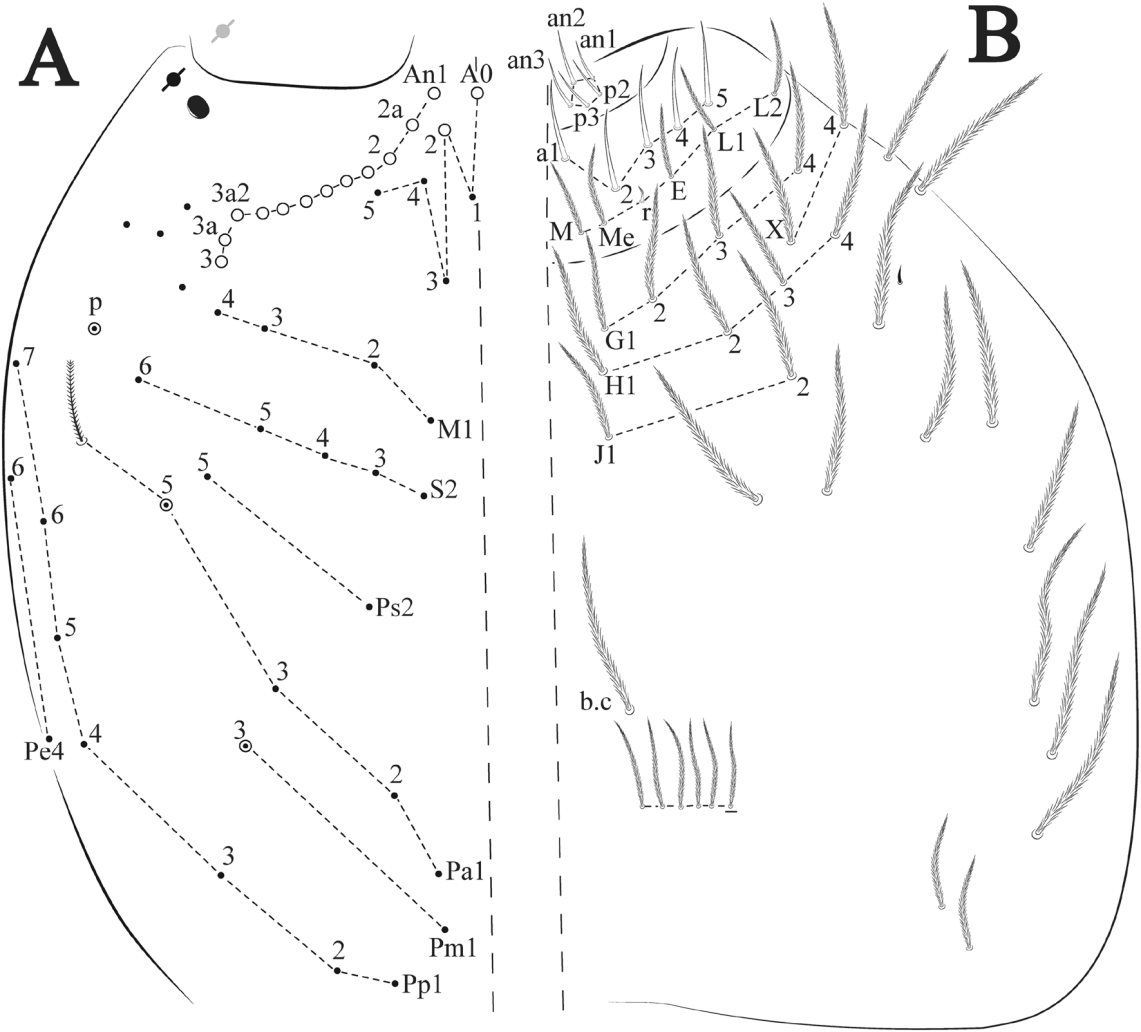
*Trogolaphysa epitychia ***sp*.* nov*.***: (**A**) Head dorsal chaetotaxy, (**B**) labial proximal chaetae, basomedial and basolateral labial fields and postlabial chaetotaxy. Black cut circle, pseudopore; Gray cut circle pseudopore at the under surface.

**Figure 34:**
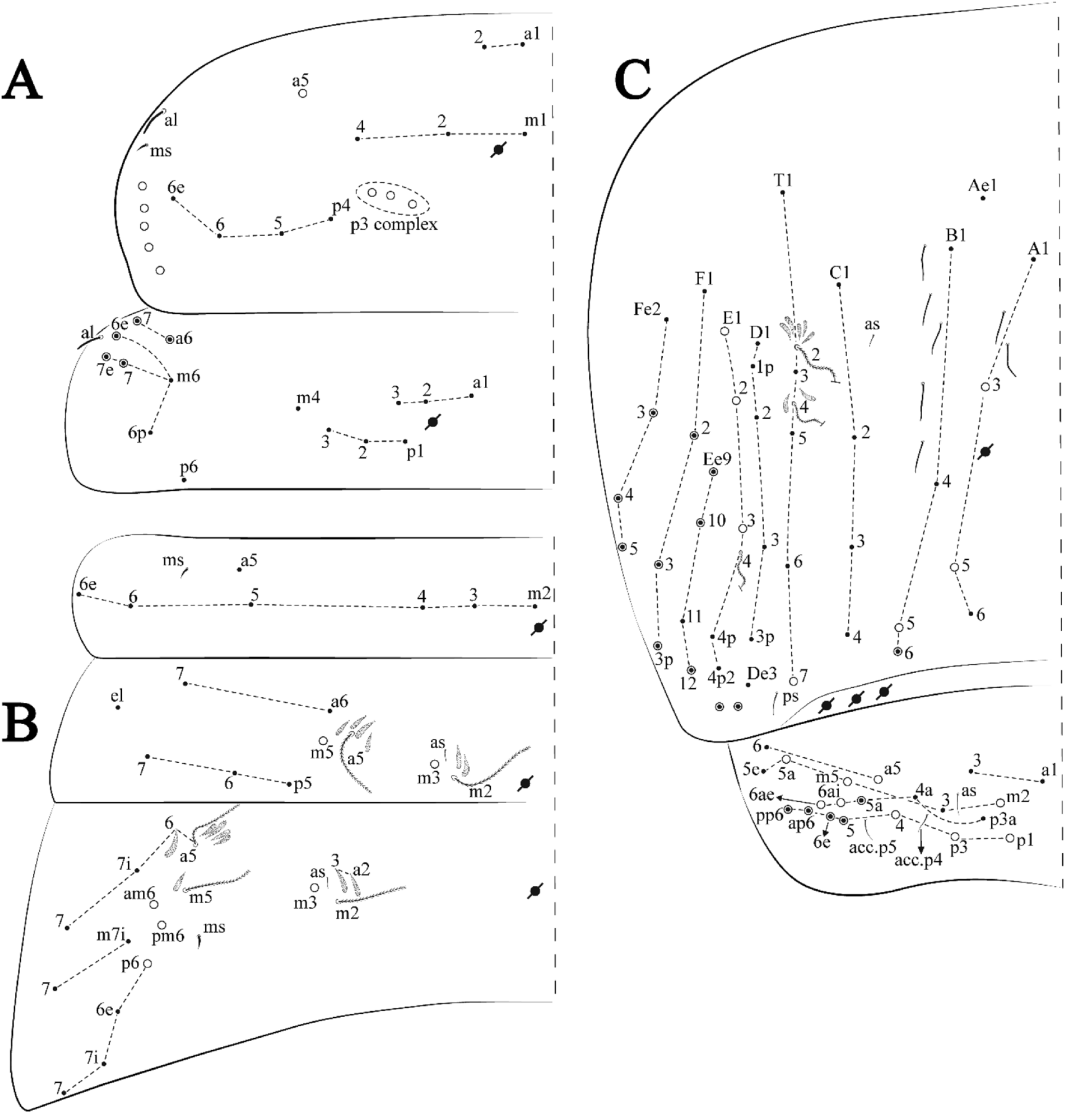
*Trogolaphysa epitychia ***sp*.* nov*.***: Dorsal chaetotaxy. (**A**) Th II–III, (**B**) Abd I–III, (**C**) Abd IV–V.

**Figure 35:**
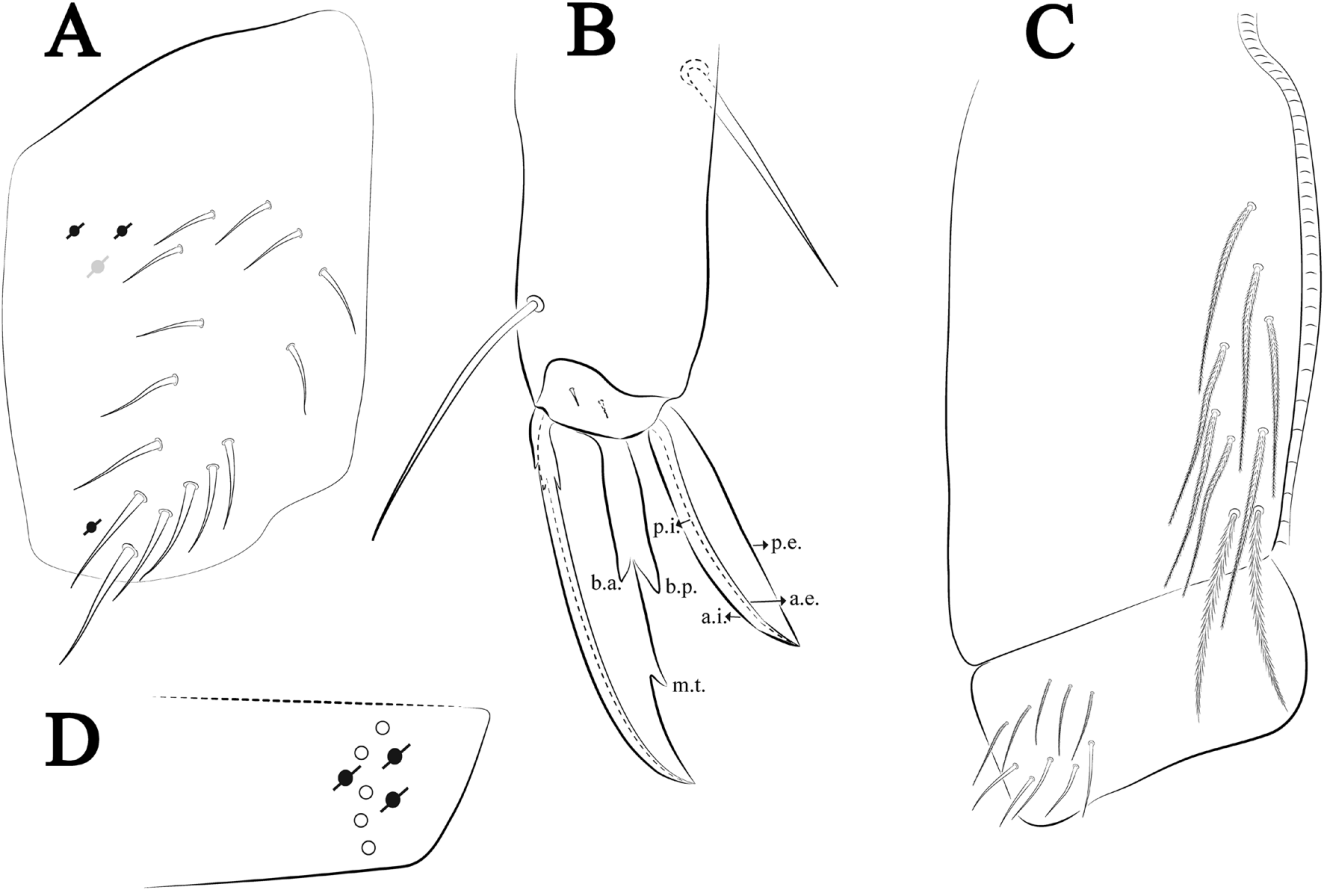
*Trogolaphysa epitychia ***sp*.* nov*.***: (**A**) Trochanteral organ, (**B**) Distal tibiotarsus and empodial complex III (anterior view), (**C**) Manubrial plate, (**D**) Antero-lateral view of collophore chaetotaxy.

Figures 33, 34 and 35, Tables 1 and 2

*Type material.* Holotype male in slide (10,578/CRFS-UEPB): Brazil, Minas Gerais State, Conceição do Mato Dentro municipality, cave CSS-0118, next to “São Sebastião do Bom Sucesso”, 18°56′14.1″S, 43°24′43.8″W, 21.xi–15.xii.2016, Carste team coll. Paratypes in slides (10,580, 10,585/CRFS-UEPB): 2 females, same data as holotype. Paratypes in slides (10,653, 10,692/CRFS-UEPB donated to MNJR): 1 female and 1 male, same data as holotype, except 22.xi–15.xii.2016 and 31.v–12.vi.2016, respectively. Additional records see S1.

*Description.* Total length (head + trunk) 1.13–1.35 mm (n = 5), holotype 1.13 mm.

Head. Ratio antennae: trunk = 1: 1.29–1.95 (n = 5), holotype = 1: 1.95; Ant III shorter than Ant II; Ant segments ratio as I: II, III, IV = 1: 1.69–2.20, 1.14–1.86, 2.37–3.52, holotype = 1: 1.71, 1.14, 2.37. Antennal chaetotaxy (no represented): Ant IV dorsally and ventrally with several short ciliate mic and mac, and finger-shaped sens, dorsally with one longitudinal row with about six rod sens, ventrally with one subapical-organ and one longitudinal row with about four wrinkly sens (Fig. 4A); Ant III dorsally and ventrally with several short ciliate mic and mac, and finger-shaped sens, dorsally without modified sens, ventrally with one apical **psp**, about three wrinkly sens on external longitudinal row, apical organ with two coffee bean-like sens, one rod sens and one smooth mic (Fig. 4A); Ant II dorsally and ventrally with several short ciliate mic and mac, dorsally with about six sub-apical finger-shaped sens and one wrinkly sens, ventrally with one apical **psp**, about three wrinkly sens on longitudinal external row (Fig. 4A); and Ant I dorsally and ventrally with several short ciliate mic and mac, three basal spine-like sens, ventrally with four basal spine-like sens, about three smooth mic, several finger-shaped sens, and two wrinkly sens (Fig. 4A). Eyes 0 + 0. Head dorsal chaetotaxy (Fig. 33A) with 12 **An** (**An1a**–**3**), six **A** (**A0**–**5**), four **M** (**M1**–**4**), five **S** (**S2**–**6**), two **Ps** (**Ps2**, **Ps5**), four **Pa** (**Pa1**–**5**), two **Pm** (**Pm1**, **Pm3**), seven **Pp** (**Pp1**–**7**), and two **Pe** (**Pe4**, **Pe6**) chaetae; **Pm3** and **Pa5** as mes, **An1a**–**3a**, **A0** and **A2** as mac; interocular **p** mes present. Basomedian and basolateral labial fields with **a1**–**5** smooth, **M**, **Me**, **E** and **L1**–**2** ciliate, **r** reduced (Fig. 33B). Ventral chaetotaxy with 31–32 ciliate chaetae and one reduced lateral spine; postlabial **G1**–**4**; **X**, **X4**; **H1**–**4**; **J1**–**2**, chaetae **b.c.** present and a collar row of five to six mes chaetae distally (Fig. 33B). Prelabral chaetae ciliate. Labral chaetae smooth, no modifications. Labial papilla **E** with **l.p**. finger-shaped and surpassing the base of apical appendage. Labial proximal chaetae smooth (**an1**–**3**, **p2**–**3**) and subequal in length (Fig. 33B). Maxillary palp with **t.a.** smooth and 1.26× larger than **b.c**.

Thorax dorsal chaetotaxy (Fig. 34A). Th II **a**, **m**, **p** series with two mic (**a1**–**2**), one mac (**a5**), three mic (**m1**–**2**, **m4**) and four mic (**p4**–**6e**), **p3** complex with three mac, respectively, **al** and **ms** present. Th III **a**, **m**, **p** series with three mic (**a1**–**3**), two mes (**a6–a7**), three mic (**m4, m6–6p**), three mes (**m6e**, **m7–7e**), four mic (**p1**–**3**, **p6**) respectively. Ratio Th II: III = 1.05–1.21: 1 (n = 5), holotype = 1.18: 1.

Abdomen dorsal chaetotaxy (Fig. 34B, C). Abd I **a**, **m** series with one (**a5**) and six (**m2**–**6e**) mic respectively, **ms** present. Abd II **a, m**, **p** series with two mic (**a6**–**7**), two mac (**m3**, **m5**), three mic (**p5**–**7**) respectively, **el** mic and **as** present; **a5** and **m2** bothriotricha surrounded by four and two fan-shaped chaetae respectively. Abd III **a**, **m**, **p** series with two mic (**a7i**–**7**), three fan-shaped chaetae (**a2**–**3**, **a6**), two mic (**m7i**–**7**), three mac (**m3**, **am6**, **pm6**), three mic (**p6e**, **p7i–7**), one mac (**p6**) chaetae, respectively; **a5**, **m2** and **m5** bothriotricha with five, two and one fan-shaped chaetae, respectively; **as** sens elongated, **ms** present. Abd IV **A**–**Fe** series with three mic (**A1**, **A6**, **Ae1**), two mac (**A3**, **A5**), two mic (**B1**, **B4**), one mes (**B6**), one mac (**B5**), four mic (**C1**–**4**), four mic (**T1**, **T3**, **T5**–**6**), one mac (**T7**), six mic (**D1**–**3p, De3**), two mic (**E4p**–**4p2**), three mac (**E1**–**3**), one mic (**Ee11**), three mes (**Ee9**–**10, Ee12**), one mic (**F1**), three mes (**F2**–**3p**), one mic (**Fe2**), three mes (**Fe3**–**5**) chaetae, respectively; **T2**, **T4** and **E4** bothriotricha surrounded by five and two fan-shaped chaetae, respectively; **ps** and **as** present, and at least seven supernumerary sens with uncertain homology *‘s’* (Fig. 8A); Abd. IV posteriorly with three **psp**. Abd V **a**, **m**, **p** series with three mic (**a1**, **a3**, **a6**), one mac (**a5**), two mic (**m3**, **me5**), three mac (**m2**, **m5**–**5a**), two mic (**p3a**–**4a**), one mes (**p5a**) two mac (**p6ai**–**6ae**), four mes (**p5**–**pp6**), three mac (**p1**, **p3**–**4**) chaetae, respectively; **as**, **acc**.**p4**–**5** present. Ratio Abd III: IV = 1: 4.69–5.55 (n = 5), holotype = 1: 4.88.

Legs. Trochanteral organ in V–shape with about 15 spine-like chaetae, plus 4 **psp** one external, one on distal vertex and another two on top of posterior spines row of Omt (Fig. 35A). Unguis outer side with one paired tooth straight and not developed on proximal third; inner lamella wide with four teeth, basal pair subequal, **b.p.** little larger, not reaching the **m.t.** apex, **m.t.** just after the distal half, **a.t.** absent. Unguiculus with all lamellae smooth and slightly truncate (**a.i.**, **a.e.**, **p.i.**, **p.e.**) (Fig. 35B); ratio unguis: unguiculus = 1.17–1.98: 1 (n = 5), holotype = 1.17: 1. Tibiotarsal smooth chaetae about 0.8× smaller than unguiculus; tenent hair acuminate and about 0.53× smaller than unguis outer lamella.

Collophore (Fig. 35C). Anterior side with nine ciliate, apically acuminate chaetae, five proximal, two subdistal and two distal mac; lateral flap with 10 chaetae, five ciliate in the proximal row and five smooth in the distal row.

Furcula. Covered with ciliate chaetae, spine-like chaetae and scales. Manubrial plate with five ciliate chaetae (two inner mac) and three **psp** (Fig. 35D). Dens posterior face with two or more longitudinal rows of spine-like chaetae about 60 external and 34 internal, external spines larger and thinner than internal ones. Mucro with four teeth, ratio width: length = 0.30 (holotype).

*Etymology. Epitychia* from Greek means success, in allusion to the collection site where the species was found *São Sebastião do Bom Sucesso*.

*Remarks. Trogolaphysa epitychia* **sp. nov.** resembles *T. bellinii* **sp. nov.**, *T. bessoni,* and *T. mariecurieae* **sp. nov.** by the absence of eyes (*T. bellinii* **sp. nov.** rarely with 2 + 2 eyes), Th II with 3 + 3 mac, and Th III without mac. Differentiates from *T. bellinii* **sp. nov.** and *T. mariecurieae* **sp. nov.** by Abd IV with 3 + 3 (**A3**, **A5**, **B5**), 4 + 4, and 2 + 2 mac on Abd IV respectively; on *T. bellinii* **sp. nov.** and can be distinguished from *T. bessoni* by the absence of unpaired tooth on inner lamella of unguis, external row of dens with 25 spines, inner row of dens with 20 spines (*T. epitychia ***sp. nov.** with one unpaired tooth **m.t.** on inner lamella of unguis, external row of dens with about 60 spines and inner row of dens with about 34 spines).

*Trogolaphysa zampauloi* **sp. nov.** Lima, Oliveira & Zeppelini

**Figure 36:**
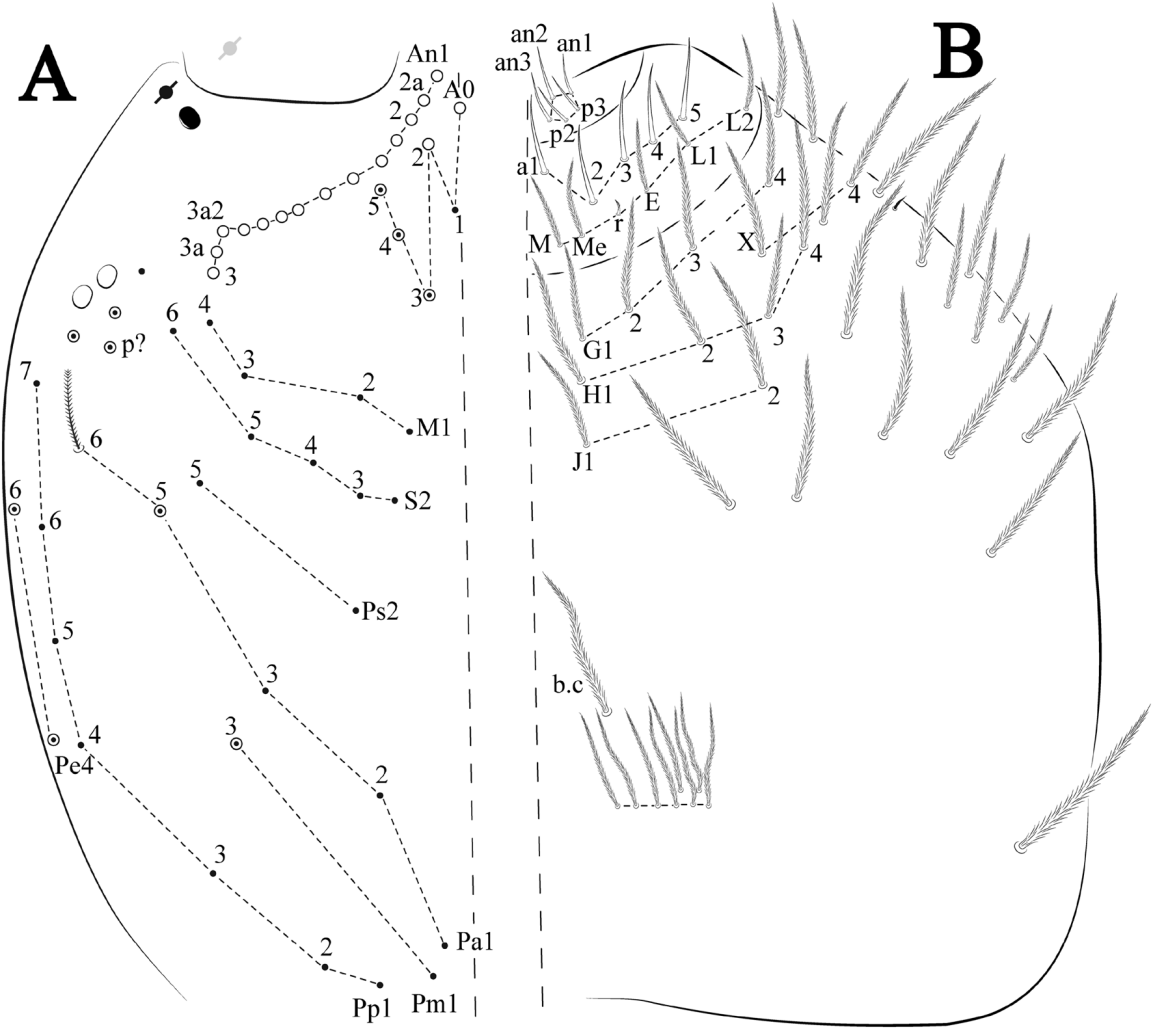
*Trogolaphysa zampauloi* **sp. nov.**: (**A**) Head dorsal chaetotaxy, (**B**) labial proximal chaetae, basomedial and basolateral labial fields and postlabial chaetotaxy. Black cut circle, pseudopore; Gray cut circle pseudopore at the under surface.

**Figure 37:**
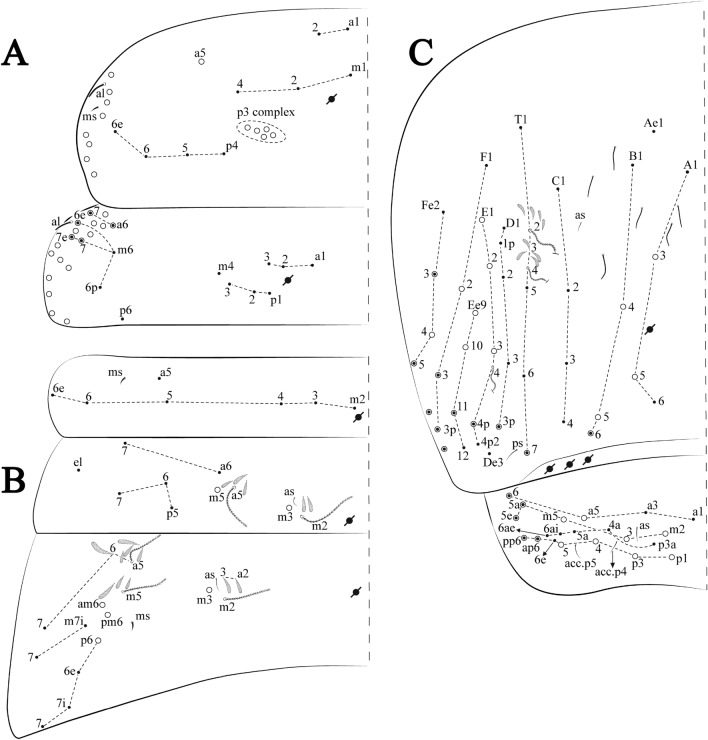
*Trogolaphysa zampauloi* **sp. nov.**: Dorsal chaetotaxy. (**A**) Th II–III, (**B**) Abd I–III, (**C**) Abd IV–V.

**Figure 38:**
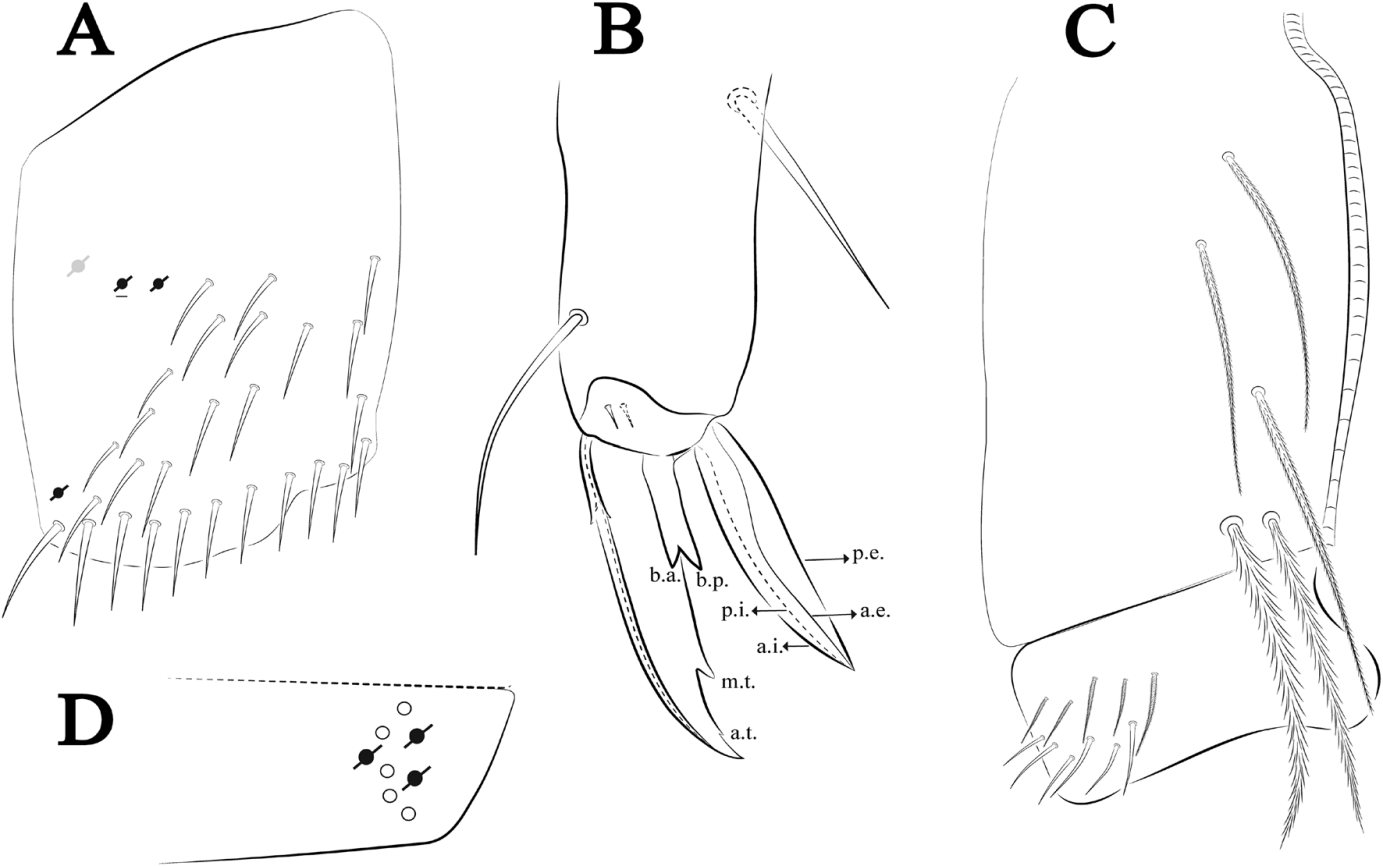
*Trogolaphysa zampauloi* **sp. nov.**: (**A**) Trochanteral organ, (**B**) Distal tibiotarsus and empodial complex III (anterior view), (**C**) Manubrial plate, (**D**) Antero-lateral view of collophore chaetotaxy.

Figures 36, 37 and 38, Tables 1 and 2

*Type material.* Holotype female in slide (11,851/CRFS-UEPB): Brazil, São Paulo State, Ribeira municipality, cave MTD-13, nexto to “Serra Pontalhão”, 24°38′47.4″S, 48°57′52.6″W, 08–20.iii.2016, Carste team coll. Paratypes in slides (11,875–11,878/CRFS-UEPB): 2 males and 2 females, Brazil, São Paulo State, Ribeira municipality, cave MTD-02, 24°37′27.3″S, 48°57′35.8″W, 08–20.iii.2016. Paratype in slide (11,876/CRFS-UEPB donated to MNJR). Additional records see S1.

*Description.* Total length (head + trunk) of specimens 1.35–1.91 mm (n = 5), holotype 1.35 mm.

Head. Ratio antennae: trunk = 1: 1.35–1.55 (n = 2), holotype = 1: 1.55; Ant III smaller than Ant II length; Ant segments ratio as I: II, III, IV = 1: 1.71–2.38, 1.60–1.88, 2.85–3.61, holotype = 1: 2.38, 1.88, 3.61. Antennal chaetotaxy (no represented): Ant IV dorsally and ventrally with several short ciliate mic and mac, and finger-shaped sens, dorsally with about three rod sens on longitudinal row, ventrally with one subapical-organ, and about three wrinkly sens (Fig. 4A); Ant III dorsally and ventrally with several short ciliate mic and mac, and finger-shaped sens, dorsally without modified sens, ventrally with one apical **psp**, one apical wrinkly sens, apical organ with two coffee bean-like sens, and one rod sens (Fig. 4A); Ant II dorsally and ventrally with several short ciliate mic and mac, dorsally with about three sub-apical finger-shaped sens and two apical rod sens, ventrally with one apical **psp**, one longitudinal external row with two subapical finger-shaped sens and two medial wrinkly sens (Fig. 4A); and Ant I dorsally and ventrally with several short ciliate mic and mac, dorsally with three basal spine-like sens, ventrally with four basal spine-like sens, about four smooth mic and several finger-shaped sens (Fig. 4A). Eyes 0 + 0 to 4 + 4. Head dorsal chaetotaxy (Fig. 36A) with 14 **An** (**An1a**–**3**), six **A** (**A0**–**5**), four **M** (**M1**–**4**), five **S** (**S2**–**6**), two **Ps** (**Ps2**, **Ps5**), four **Pa** (**Pa1**–**3**, **Pa5**), two **Pm** (**Pm1**, **Pm3**), seven **Pp** (**Pp1**–**7**), and two **Pe** (**Pe4**, **Pe6**) chaetae; **Pe4**, **Pe6**, **Pm3** and **Pa5** as mes, **An1a**–**3a** as mac, **A0** and **A2** as mac, **A3**–**5** as mes; interocular **p** mes present. Basomedian and basolateral labial fields with **a1**–**5** smooth, **M**, **Me**, **E** and **L1**–**2** ciliate, **r** reduced (Fig. 36B). Ventral chaetotaxy with about 37 ciliate chaetae, plus one reduced lateral spine; postlabial **G1**–**4**; **X**, **X4**; **H1**–**4**; **J1**–**2**, chaetae **b.c.** present and a collar row of eight mes chaetae distally (Fig. 36B). Prelabral chaetae ciliate. Labral chaetae smooth, no modifications. Labial papilla **E** with **l.p**. finger-shaped and surpassing the base of apical appendage. Labial proximal chaetae smooth (**an1**–**3**, **p2**–**3**) and subequal in length (Fig. 36B). Maxillary palp with **t.a.** smooth and 1.17 × smaller than **b.a**.

Thorax dorsal chaetotaxy (Fig. 37A). Th II **a**, **m**, **p** series with two mic (**a1**–**2**), one mac (**a5**), three mic (**m1**–**2**, **m4**) and four mic (**p4**–**6e**), **p3** complex with five mac, respectively, **al** and **ms** present. Th III **a**, **m**, **p** series with three mic (**a1**–**3**), two mes (**a6**–**a7**), three mic (**m4**, **m6**–**6p**), three mes (**m6e**, **m7**–**7e**), four mic (**p1**–**3**, **p6**), respectively. Ratio Th II: III = 1.02–1.48: 1 (n = 5), holotype = 1.21: 1

Abdomen dorsal chaetotaxy (Fig. 37B, C). Abd I **a**, **m** series with one (**a5**) and six (**m2**–**6e**) mic respectively, **ms** present. Abd II **a**, **m**, **p** series with two mic (**a6**–**7**), two mac (**m3**, **m5**), three mic (**p5**–**7**) respectively, **el** as mic and **as** present; **a5** and **m2** bothriotricha surrounded by three and two fan-shaped chaetae respectively. Abd III **a**, **m**, **p** series with one mic (**a7**), three fan-shaped chaetae (**a2**–**3**, **a6**), two mic (**m7i**–**7**), three mac (**m3**, **am6**, **pm6**), three mic (**p6e**, **p7i–7**), one mac (**p6**) chaetae respectively; **a5**, **m2** and **m5** bothriotricha with five, two and three fan-shaped chaetae respectively, **as** sens elongated, **ms** present. Abd IV **A**–**Fe** series with three mic (**A1**, **A6**, **Ae1**), two mac (**A3**, **A5**), one mic (**B1**), one mes (**B6**), two mac (**B4**–**5**), four mic (**C1**–**4**), three mic (**T1**, **T5**–**6**), one mes (**T7**), five mic (**D1**–**3**, **De3**), one mes (**D3p**), one mic (**E4p2**), one mes (**E4p**), three mac (**E1**–**3**), one mic (**Ee12**), one mes (**Ee11**), two mac (**Ee9**–**10**), one mic (**F1**), two mes (**F3–3p**), one mac (**F2**), one mic (**Fe2**), two mes (**Fe3**, **Fe5**), one mac (**Fe4**) chaetae, respectively; **T2**, **T4** and **E4** bothriotricha surrounded by four and four (**T3**) fan-shaped chaetae respectively; **ps** and **as** present, and at least six supernumerary sens with uncertain homology *‘s’* (Fig. 8A); Abd. IV posteriorly with three **psp**. Abd V **a**, **m**, **p** series with two mic (**a1**, **a3**), one mes (**a6**), one mac (**a5**), two mes (**m5a**, **m5e**), three mac (**m2**–**3**, **m5**), five mic (**p3a**–**6ae**), one mic (**p6e**) two mes (**ap6**–**pp6**), four mac (**p1**, **p3**–**5**) chaetae, respectively; **as**, **acc**.**p4**–**5** present. Ratio Abd III: IV = 1: 3.29–4.28 (n = 5), holotype = 1: 4.10.

Legs. Trochanteral organ diamond shape with about 27 spine-like chaetae, plus 3–4 **psp** one external, one on distal vertex and another two (one of them present or absent) on top of posterior spines row of Omt (Fig. 38A). Unguis outer side with one paired tooth straight and not developed on proximal third; inner lamella wide with four teeth, basal pair subequal, **b.p**. not reaching the **m.t.** apex, **m.t.** just after the distal half, **a.t.** present. Unguiculus with all lamellae smooth and lanceolate (**a.i.**, **a.e.**, **p.i.**, **p.e.**) (Fig. 38B); ratio unguis: unguiculus = 1.63–1.84 (n = 5), holotype = 1: 1.79. Tibiotarsal smooth chaetae about 0.8× smaller than unguiculus; tenent hair acuminate and about 0.39× smaller than unguis outer edge.

Collophore (Fig. 38C). Anterior side with five ciliate, apically acuminate chaetae, two proximal (thinner); one subdistal and two distal mac; lateral flap with 11 chaetae, five ciliate in the proximal row and six smooth in the distal row.

Furcula. Covered with ciliate chaetae, spine-like chaetae and scales. Manubrial plate with four ciliate chaetae (two inner mac) and three **psp** (Fig. 38D). Dens posterior face with two or more longitudinal rows of spine-like chaetae about 30 external and 23 internal, external spines larger and thinner than internal ones. Mucro with four teeth, ratio width: length = 0.29 (n = 5).

Etymology. Species named after the field biologist MSc. Robson de Almeida Zampaulo for his contribution to Brazilian biospeleology.

Remarks. *Trogolaphysa zampauloi* **sp. nov.** resembles *T. caripensis; T. ernesti; T. piracurucaensis* by Th III without mac, and 4 + 4 central mac (**A3**, **A5**, **B4**–**5**) in Abd IV, but is easily distinguished from these species by the presence of Th II with 4 + 4 mac in **p3** complex (6 + 6T*. caripensis, T. ernesti, T. piracurucaensis*). For more comparisons see remarks in *T. crystallensis* **sp. nov.**

*Trogolaphysa gisbertae* **sp. nov.** Brito & Zeppelini

**Figure 39:**
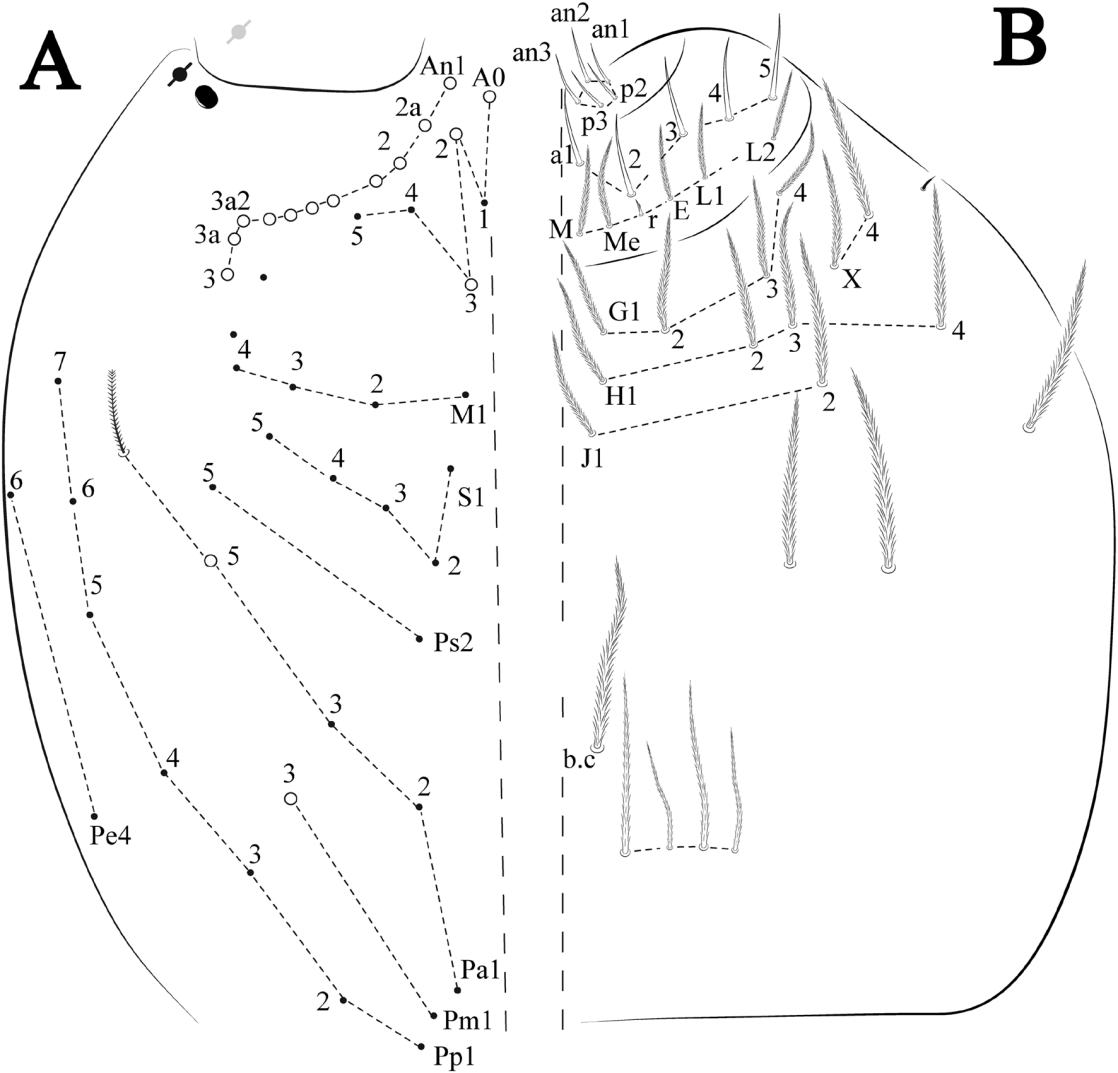
*Trogolaphysa gisbertae* **sp. nov.**: (**A**) Head dorsal chaetotaxy, (**B**) labial proximal chaetae, basomedial and basolateral labial fields and postlabial chaetotaxy. Black cut circle, pseudopore; Gray cut circle pseudopore at the under surface.

**Figure 40:**
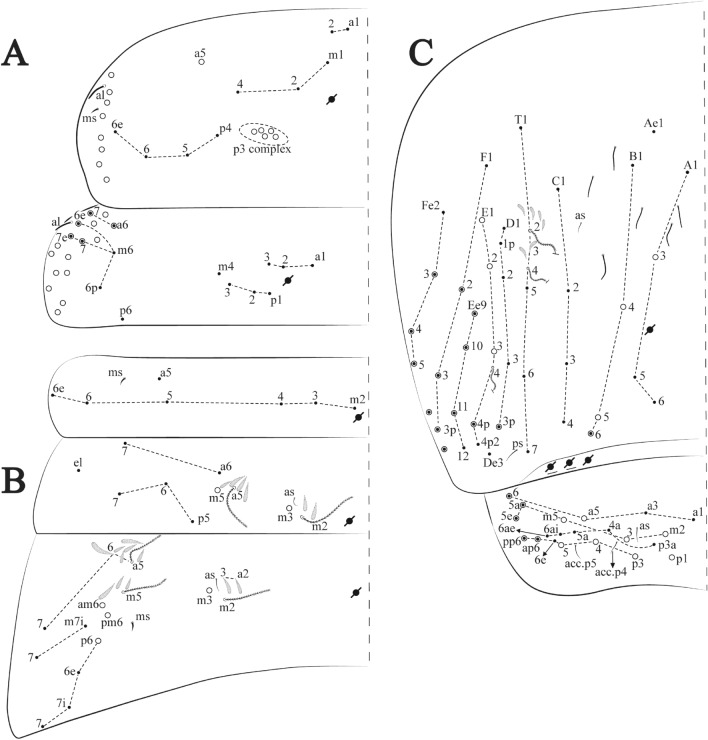
*Trogolaphysa gisbertae* **sp. nov.**: Dorsal chaetotaxy: (**A**) Th II–III, (**B**) Abd I–III, (**C**) Abd IV–V.

**Figure 41:**
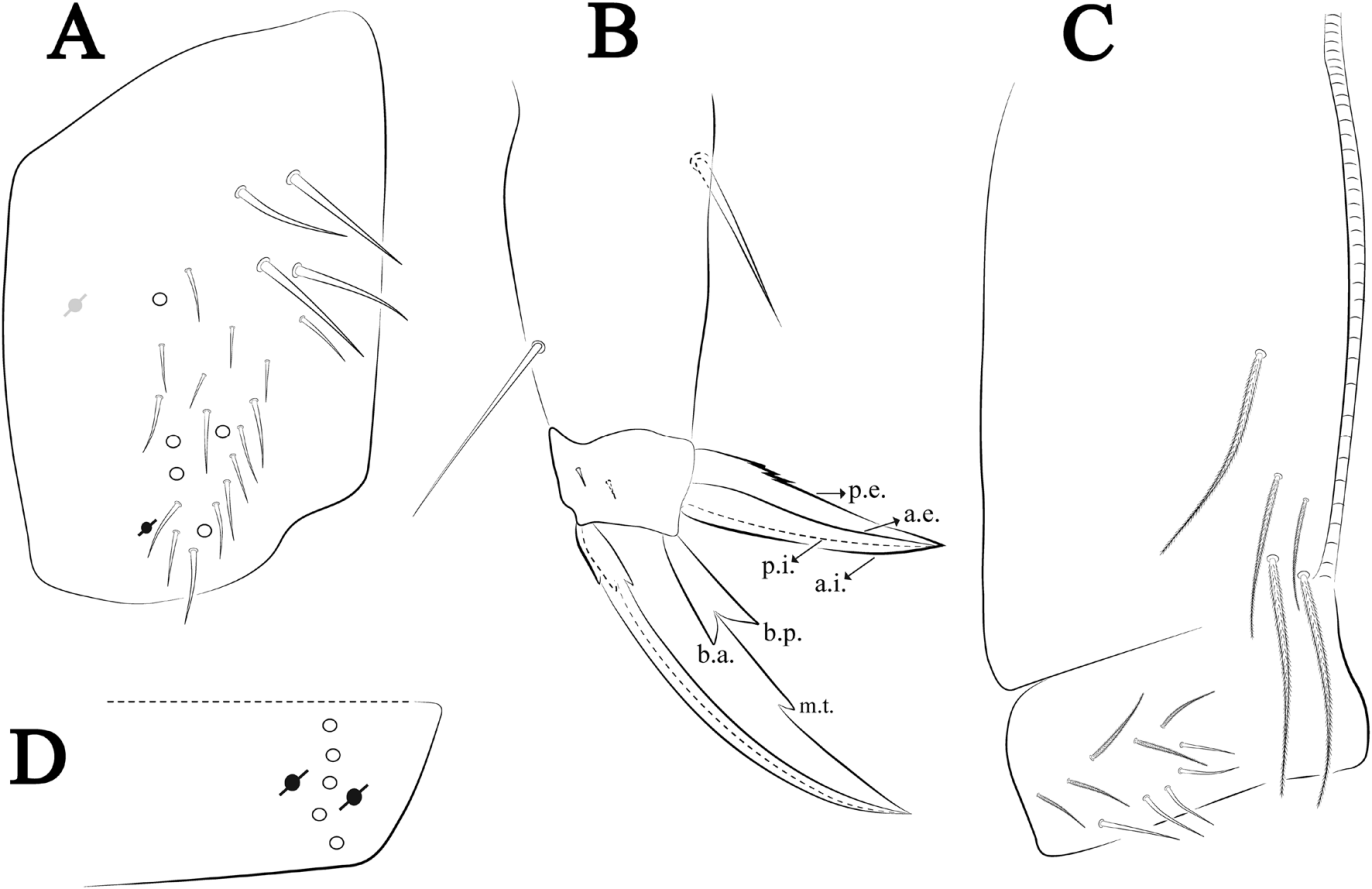
*Trogolaphysa gisbertae* **sp. nov.**: (**A**) Trochanteral organ, (**B**) Distal tibiotarsus and empodial complex III (anterior view), (**C**) Manubrial plate, (**D**) Antero-lateral view of collophore chaetotaxy.

Figures 39, 40 and 41, Tables 1 and 2

*Type material.* Holotype female in slide (6668/CRFS-UEPB): Brazil, Pará State, Parauapebas municipality, cave N1N8-N8-017, next to “Serra Norte”, 06°10′05.9″S, 50°09′25.6″W, 02–29.iv.2015, Carste team coll. Paratype in slide (6669/CRFS-UEPB donated to MNJR): 1 female, same data as holotype, except 04.ix–06.x.2014. Paratype in slide (6973/CRFS-UEPB): 1 female, same data as holotype, except 04.ix–06.x.2014. Paratypes in slides (6657, 7138/CRFS-UEPB): 2 females, Brazil, Pará State, Parauapebas municipality, N1N8-N8-020 cave, 06°10′07.8″S, 50°09′25.4″W, 17.vii–04.viii.2014, Carste team coll. Additional records see S1.

*Description.* Total length (head + trunk) of specimens 1.10–1.23 mm (n = 5), holotype 1.15 mm.

Head. Ratio antennae: trunk = 1: 1.44–1.55 (n = 3); Ant segments ratio as I: II, III, IV = 1: 1.67–2.43, 1.58–2.63, 2.91–5.46, holotype = 1: 2.03, – , 3.90. Antennal chaetotaxy (no represented): Ant IV dorsally and ventrally with several short ciliate mic and mac, and finger-shaped sens, dorsally with about five rod sens in row, ventrally with one subapical-organ and several wrinkly sens row (Fig. 4A); Ant III dorsally and ventrally with several short ciliate mic and mac, and finger-shaped sens, dorsally without modified sens, ventrally with one apical **psp**, about four wrinkly sens on external longitudinal row, apical organ with one finger-shaped sens, two coffee bean-like sens, and one rod sens (Fig. 4A); Ant II dorsally and ventrally with several short ciliate mic and mac, dorsally with four finger-shapedd sens in row and two subapical rod sens, ventrally with one apical **psp**, and about five wrinkly sens on longitudinal external row (Fig. 4A); and Ant I dorsally and ventrally with several short ciliate mic and mac, dorsally with three basal spine-like sens, ventrally with four basal spine-like sens, about five smooth mic and several fniger-shaped sens (Fig. 4A). Eyes 0 + 0. Head dorsal chaetotaxy (Fig. 39A) with 11 **An** (**An1a**–**3**), six **A** (**A0**–**5**), four **M** (**M1**–**4**), five **S** (**S1**–**5**), two **Ps** (**Ps2**, **Ps5**), four **Pa** (**Pa1**–**5**), two **Pm** (**Pm1**, **Pm3**), seven **Pp** (**Pp1**–**7**), and two **Pe** (**Pe4**, **Pe6**) chaetae; **An1a**–**3a**, **A0**, **A2**–**3**, **Pa5** and **Pm3** as mac; interocular **p** absent. Basomedian and basolateral labial fields with **a1**–**5** smooth, **M**, **Me**, **E** and **L1**–**2** ciliate, **r** reduced (Fig. 39B). Ventral chaetotaxy with 20 ciliate chaetae and one reduced lateral spine; postlabial **G1**–**4**; **X**, **X4**; **H1**–**4**; **J1**–**2**, chaetae **b.c.** present and a collar row of three to four mes chaetae distally (Fig. 39B). Prelabral chaetae ciliate. Labral chaetae smooth, no modifications. Labial papilla **E** with **l.p.** finger-shaped and surpassing the base of apical appendage. Labial proximal chaetae smooth (**an1**–**3**, **p2**–**3**) and subequal in length (Fig. 39B). Maxillary palp with **t.a.** smooth and 1.32 × larger than **b.c.**

Thorax dorsal chaetotaxy (Fig. 40A). Th II **a**, **m**, **p** series with two mic (**a1**–**2**), one mac (**a5**), three mic (**m1**–**2**, **m4**) and four mic (**p4**–**6e**), **p3** complex with five mac, respectively, **al** and **ms** presents. Th III **a**, **m**, **p** series with three mic (**a1**–**3**), two mes (**a6**–**7**), three mic (**m4**, **m6**–**6p**), three mes (**m6e**, **m7**–**7e**), and four mic (**p1**–**3**, **p6**), respectively. Ratio Th II: III = 1.00–2.60: 1 (n = 5), holotype = 1.28: 1.

Abdomen dorsal chaetotaxy (Fig. 40B, C). Abd I **a**, **m** series with one (**a5**) and six (**m2**–**6e**) mic, respectively, **ms** present. Abd II **a**, **m**, **p** series with two mic (**a6**–**7**), two mac (**m3**, **m5**), three mic (**p5**–**7**) respectively, **el** mic and **as** present; **a5** and **m2** bothriotricha surrounded by four and two fan-shaped chaetae, respectively. Abd III **a**, **m**, **p** series with one mic (**a7**), three fan-shaped chaetae (**a2**–**3**, **a6**), two mic (**m7i**–**7**), three mac (**m3**, **am6**, **pm6**), three mic (**p6e**, **p7i**–**7**), one mac (**p6**) chaetae, respectively; **a5**, **m2** and **m5** bothriotricha with six, two and three fan-shaped chaetae, respectively, **as** sens elongated, **ms** present. Abd IV **A**–**Fe** series with four mic (**A1**, **A5**–**6**, **Ae1**), one mac (**A3**), one mic (**B1**), one mes (**B6**), two mac (**B4**–**5**), four mic (**C1**–**4**), four mic (**T1**, **T5**–**7**), five mic (**D1**–**3**, **De3**), one mes (**D3p**), one mic (**E4p2**), one mes (**E4p**), three mac (**E1**–**3**), one mic (**Ee12**), three mes (**Ee9**–**11**), one mic (**F1**), three mes (**F2**–**3p**), one mic (**Fe2**), three mes (**Fe3**–**5**) chaetae, respectively; **T2**, **T4** and **E4** bothriotricha surrounded by four and two (**T3**) fan-shaped chaetae, respectively; **ps** and **as** present, and at least six supernumerary sens with uncertain homology *‘s’ *(Fig. 8A); Abd. IV posteriorly with one to three **psp**. Abd V **a**, **m**, **p** series with three mic (**a1**, **a3**), one mes (**a6**), one mac (**a5**), two mes (**m5a**–**5e**), three mac (**m2**–**3**, **m5**), five mic (**p3a**–**6ae**), one mic (**p6e**), two mes (**ap6**, **pp6**), four mac (**p1**, **p3**–**5**) chaetae, respectively; **as** and **acc.p4**–**5** present. Ratio Abd III: IV = 1: 3.29–4.90 (n = 5), holotype = 1: 3.29.

Legs. Trochanteral organ diamond shape with about 25 spine-like chaetae, plus two **psp** one external, and one on distal vertex of Omt (Fig. 41A). Unguis outer side with one paired tooth straight and not developed on proximal third; inner lamella wide with three teeth, basal pair subequal, **b.p.** not reaching the **m.t.** apex, **m.t.** just after the distal half, **a.t.** absent. Unguiculus with lamellae smooth and slightly truncate (**a.i.**, **a.e.**, **p.i.**), except **p.e.** slightly serrate (Fig. 41B); ratio unguis: unguiculus = 1.59–2.05: 1 (n = 5), holotype = 1.62: 1. Tibiotarsal smooth chaetae about 0.9× smaller than unguiculus; tenent hair acuminate and about 0.53× smaller than unguis outer lamella.

Collophore (Fig. 41C). Anterior side with five ciliate, apically acuminate chaetae, one proximal (thinner); two subdistal and two distal mac; lateral flap with 10 chaetae, five ciliate in the proximal row and five smooth in the distal row.

Furcula. Covered with ciliate chaetae, spine-like chaetae and scales. Manubrial plate with five ciliate chaetae (three inner mac) and three **psp** (Fig. 41D). Dens posterior face with two or more longitudinal rows of spine-like chaetae about 18 external and 24 internal, external spines larger and thinner than internal ones. Mucro with four teeth, ratio width: length = 0.26 (holotype).

*Etymology.* Honor to Gisberta Salce Júnior, Brazilian woman, murdered in 2006 (Porto, Portugal) in a transphobia crime.

*Remarks. Trogolaphysa gisbertae* **sp. nov.** differs from *T. ernesti* and *T. formosensis* (with 0 + 0 head dorsal mac), T*. piracurucaensis*, and *T. barroca* **sp. nov.** (1+1 head dorsal mac); and resembles *T. dandarae* **sp. nov.** (with 5+5 head dorsal mac), but it is easily distinguishable by Th II **p3** complex and Th III mac (5 + 5 and 0 + 0, 6 + 6 and 3 + 3, respectively); and unguis with **m.t.** present (absent in *T. sotoadamesi* **sp. nov.**, *T. barroca* **sp. nov.**).

*Trogolaphysa dandarae* **sp. nov.** Brito & Zeppelini

**Figure 42:**
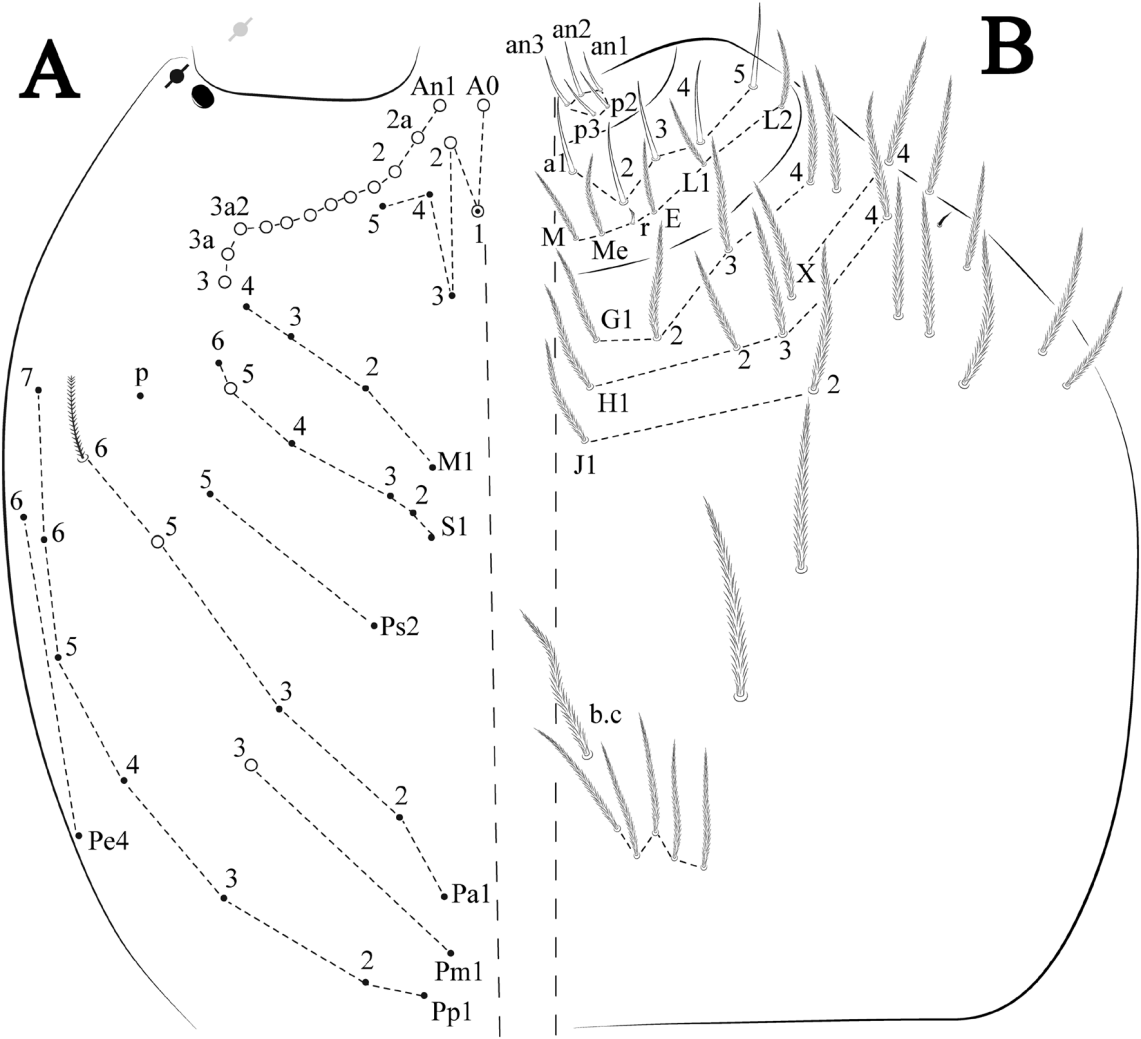
*Trogolaphysa dandarae* **sp. nov**.: (**A**) Head dorsal chaetotaxy, (**B**) labial proximal chaetae, basomedial and basolateral labial fields and postlabial chaetotaxy. Black cut circle, pseudopore; Gray cut circle pseudopore at the under surface.

**Figure 43:**
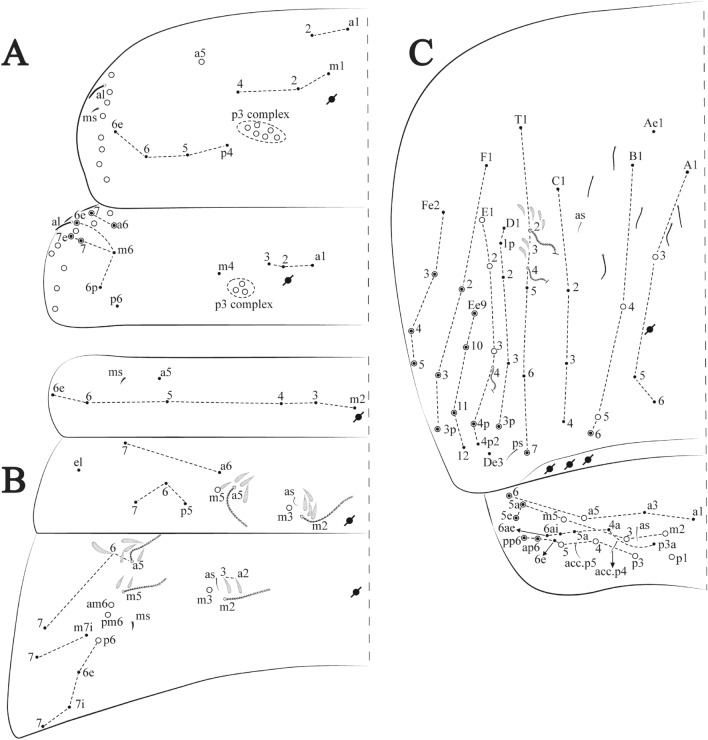
*Trogolaphysa dandarae* **sp. nov.**: Dorsal chaetotaxy. (**A**) Th II–III, (**B**) Abd I–III, (**C**) Abd IV–V.

**Figure 44:**
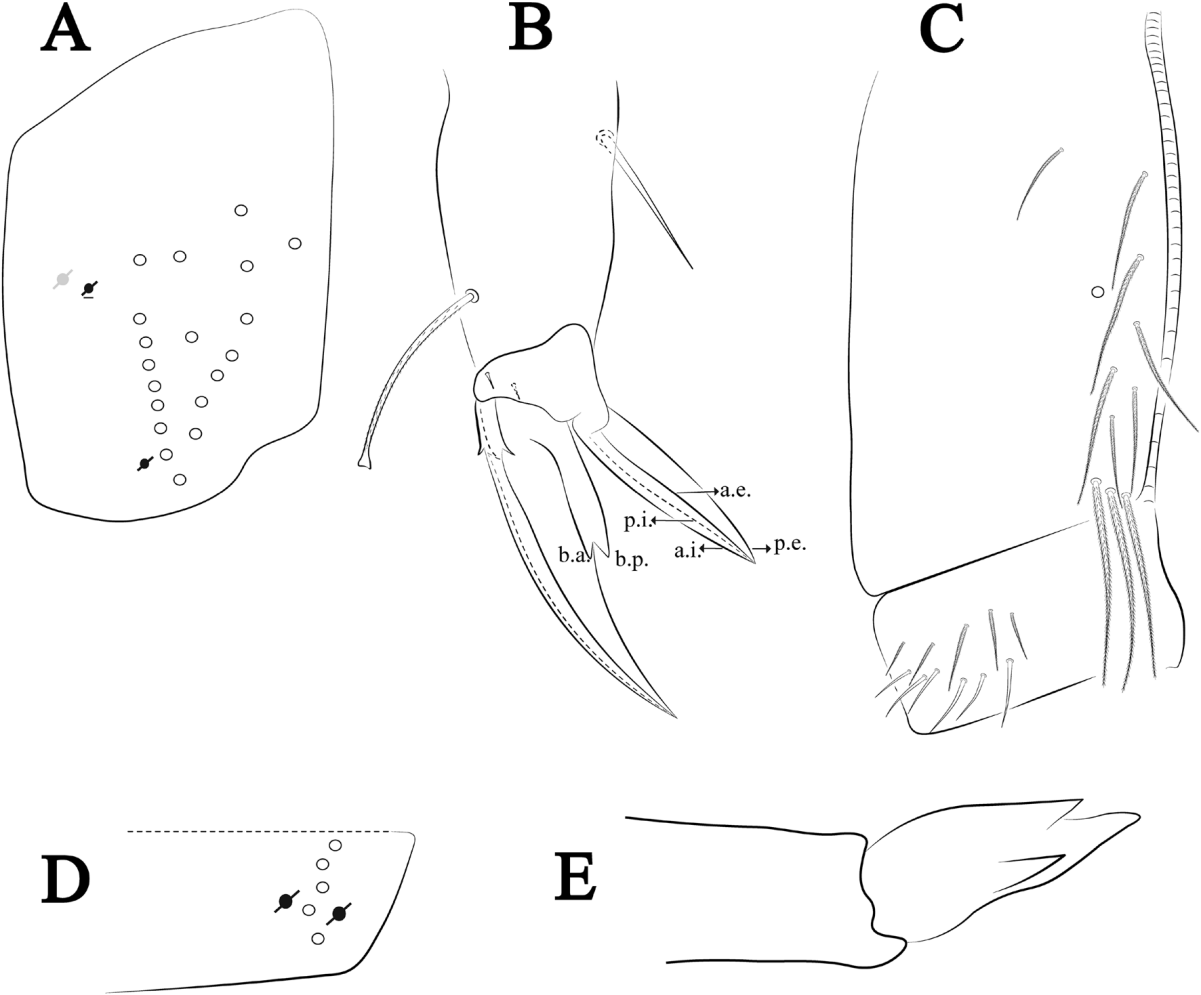
*Trogolaphysa dandarae* **sp. nov.**: (**A**) Trochanteral organ, (**B**) Distal tibiotarsus and empodial complex III (anterior view), (**C**) Manubrial plate, (**D**) Antero-lateral view of collophore chaetotaxy, (**E**) Mucro.

Figures 42, 43 and 44, Tables 1 and 2

*Type material.* Holotype female in slide (12,775/CRFS-UEPB): Brazil, Pará State, Parauapebas municipality, cave N4WS-0018/48, next to “Serra Norte”, 06°04′34.5″S, 50°11′37.7″W, 21–30.vii.2018, Brandt Meio Ambiente team coll. Paratype in slide (12,776/CRFS-UEPB donated to MNJR): 1 female, same data as holotype. Paratypes in slides (12,777, 12,778/CRFS-UEPB): 2 females, same data as holotype. Paratypes in slides (12,772, 12,773/CRFS-UEPB): 2 females, Brazil, Pará State, Parauapebas municipality, N4WS-0016 cave, 06°04′35.5″S, 50°11′37.1″W, 21–30.vii.2018, Brandt Meio Ambiente team coll. Additional records see S1.

*Description.* Total length (head + trunk) of specimens 1.43–1.75 mm (n = 5), holotype 1.58 mm.

Head. Ratio antennae: trunk = 1: 0.83–0.98 (n = 4), holotype = 1: 0.83; Ant III larger than Ant II; Ant segments ratio as I: II: III: IV = 1: 1.36–1.77: 1.65–2.03: 2.84–3.27, holotype = 1: 1.72: 1.99: 3.21. Antennal chaetotaxy (no represented): Ant IV dorsally and ventrally with several short ciliate mic and mac, and finger-shaped sens, dorsally with about two rod sens sub-apical on longitudinal row, ventrally with one subapical-organ and about three wrinkly sens on longitudinal row (Fig. 4A); Ant III dorsally and ventrally with several short ciliate mic and mac, and finger-shaped sens, dorsally without modified sens, ventrally with one apical **psp**, about three wrinkly sens and three smooth mic on external longitudinal row, apical organ with one finger-shaped sens, two coffee bean-like sens, and one rod sens (Fig. 4A); Ant II dorsally and ventrally with several short ciliate mic and mac, dorsally with about four sub-apical finger-shaped sens and two subapical rod sens, ventrally with one apical **psp**, and several wrinkly sens on longitudinal external row (Fig. 4A); and Ant I dorsally and ventrally with several short ciliate mic and mac, dorsally with three basal spine-like sens, ventrally with four basal spine-like sens, about five smooth mic and several finger-shaped sens (Fig. 4A). Eyes 0 + 0. Head dorsal chaetotaxy (Fig. 42A) with 12 **An** (**An1a**–**3**), six **A** (**A0**–**5**), four **M** (**M1**–**4**), six **S** (**S1**–**6**), two **Ps** (**Ps2**, **Ps5**), four **Pa** (**Pa1**–**5**), two **Pm** (**Pm1**, **Pm3**), seven **Pp** (**Pp1**–**7**), and two **Pe** (**Pe4**, **Pe6**) chaetae; **A1** as mes, **An1a**–**3**, **A0, A2**, **S5**, **Pa5** and **Pm3** as mac; interocular **p** mic present. Basomedian and basolateral labial fields with **a1**–**5** smooth, **M**, **Me**, **E** and **L1**–**2** ciliate, **r** reduced (Fig. 42B). Ventral chaetotaxy with 28 ciliate chaetae and one reduced lateral spine; postlabial **G1**–**4**; **X**, **X4**; **H1**–**4**; **J1**–**2**, chaetae **b.c.** present and a collar row of five chaetae distally (Fig. 42B). Prelabral chaetae ciliate. Labral chaetae smooth, no modifications. Labial papilla **E** with **l.p.** finger-shaped and subequal the base of apical appendage. Labial proximal chaetae smooth (**an1**–**3**, **p2**–**3**) and subequal in length (Fig. 42B). Maxillary palp with **t.a.** smooth and 1.58 × larger than **b.c**.

Thorax dorsal chaetotaxy (Fig. 43A). Th II **a**, **m**, **p** series with two mic (**a1**–**2**), one mac (**a5**), three mic (**m1**–**2**, **m4**) and four mic (**p4**–**6e**), **p3** complex with six mac, respectively, **al** and **ms** presents. Th III **a**, **m**, **p** series with three mic (**a1**–**3**), two mes (**a6**–**7**), two mic (**m6**–**6p**), three mes (**m6e**, **m7**–**7e**), and one mic (**p6**), respectively. Ratio Th II: III = 0.82–1.13: 1 (n = 6), holotype = 1.13: 1.

Abdomen dorsal chaetotaxy (Fig. 43B, C). Abd I **a**, **m** series with one (**a5**) and six (**m2**–**6e**) mic, respectively, **ms** present. Abd II **a**, **m**, **p** series with two mic (**a6**–**7**), two mac (**m3**, **m5**), three mic (**p5**–**7**) respectively, **el** mic and **as** present; **a5** and **m2** bothriotricha surrounded by four and four fan-shaped chaetae, respectively. Abd III **a**, **m**, **p** series with one mic (**a7**), three fan-shaped chaetae (**a2**–**3**, **a6**), two mic (**m7i**–**7**), three mac (**m3**, **am6**, **pm6**) and three mic (**p6e**–**7**), one mac (**p6**) chaetae respectively; **a5**, **m2** and **m5** bothriotricha with five, two and two fan-shaped chaetae, respectively, **as** sens elongated, **ms** present. Abd IV **A**–**Fe** series with four mic (**A1**, **A5**–**6**, **Ae1**), one mac (**A3**), one mic (**B1**), one mes (**B6**), two mac (**B4**–**5**), four mic (**C1**–**4**), three mic (**T1**, **T5**–**6**), one mes (**T7**), five mic (**D1**–**3**, **De3**), one mes (**D3p**), one mic (**E4p2**), one mes (**E4p**), three mac (**E1**–**3**), one mic (**Ee12**), three mes (**Ee9**–**11**), one mic (**F1**), three mes (**F2**–**3p**), one mic (**Fe2**), three mes (**Fe3**–**5**) chaetae, respectively; **T2**, **T4** and **E4** bothriotricha surrounded by four and two (**T3**) fan-shaped chaetae, respectively; **ps** and **as** present, and at least six supernumerary sens with uncertain homology *‘s’* (Fig. 8A); Abd. IV posteriorly with three **psp**. Abd V **a**, **m**, **p** series with three mic (**a1**, **a3**), one mes (**a6**), one mac (**a5**), two mic (**m5a**–**5e**), three mac (**m2**–**3**, **m5**), five mic (**p3a**–**6ae**), one mic (**p6e**), two mes (**ap6**, **pp6**), four mac (**p1**, **p3**–**5**) chaetae, respectively; **as** and **acc.p4**–**5** present. Ratio Abd III: IV = 1: 2.98–4.82 (n = 6), holotype = 1: 3.81.

Legs. Trochanteral organ diamond shape with about 19 spine-like chaetae, plus 2–3 **psp** one external, one on distal vertex and another (present or absent) on top of posterior spines row of Omt (Fig. 44A). Unguis outer side with one paired tooth straight and not developed on proximal third; inner lamella wide with two teeth, basal pair subequal, **m.t.** and **a.t.** absent. Unguiculus with all lamellae smooth and lanceolate (**a.i.**, **a.e.**, **p.i.**, **p.e.**) (Fig. 44B); ratio unguis: unguiculus = 1.49–1.80: 1 (n = 6), holotype = 1.80: 1. Tibiotarsal smooth chaetae about 1.25× smaller than unguiculus; tenent hair slightly capitate and about 0.54× smaller than unguis outer lamella.

Collophore (Fig. 44C). Anterior side with 11 ciliate, apically acuminate chaetae, six proximal (thinner); two subdistal and three distal mac; lateral flap with 11 chaetae, five ciliate in the proximal row and six smooth in the distal row.

Furcula. Covered with ciliate chaetae, spine-like chaetae and scales. Manubrial plate with four ciliate chaetae (two inner mac) and three **psp** (Fig. 44D). Dens posterior face with two or more longitudinal rows of spine-like chaetae about 31–39 external and 18–21 internal, external spines larger and thinner than internal ones. Mucro with three teeth (Fig. 44E), ratio width: length = 0,28 (holotype).

*Etymology.* Honor to Dandara Kettley, Brazilian man, transvestite, murdered in 2017 (Ceará, Brazil) in a homophobia crime.

*Remarks. Trogolaphysa dandarae* **sp. nov.** resembles *T. ernesti*, *T. formosensis* and *T. piracurucaensis* by chaetae head **S5** mac (all other Brazilian cave species with **S5** mic); head **Pm3** mac as in *T. gisbertae* **sp. nov.**, but they are different in terms of head ventral proximal collar mac, unguiculus, tenent hair and collophore anterior distal chaetae (5 + 5, smooth **pe**, capitate, 3 + 3 and 4 + 4, serrate **pe**, acuminate, 2 + 2, respectively); Th II P3 complex with 6 + 6 and Th III with 3 + 3 mac (6 + 6 and 0 + 0 in *T. lacerta* **sp. nov.**, *T. piracurucaensis*, *T. ernesti* and *T. caripensis*); *T. dandarae* **sp. nov.**, *T. belizeana* and *T. jacobyi* are the only cave species with 3 + 3 teeth in the mucro. See the comparison among them in remarks of the late species.

## Supplementary Information

Supplementary Information 1.

Supplementary Information 2.

## Data Availability

The datasets generated or analyzed during the current study are available from the corresponding author upon reasonable request.
