# Supplementary material for: Hotspot in ferruginous rock may have serious implications in Brazilian conservation policy
Source: Sci Rep. 2022 Sep 1;12:14871. doi: 10.1038/s41598-022-18798-1 (PMC9437091; doi:10.1038/s41598-022-18798-1)
Supplement: Supplementary file 1 — Supplementary Information 1. [file 41598_2022_18798_MOESM1_ESM.pdf]

Data entry order	Country	State	County	Locality	Coordinates		Date Collected	Collectors	*Species*
15466	Brasil	MG	Barão de Cocais	MDIR-0028	-19 52 48.690	-43 26 13.599	19-23.VIII.2019	Equipe Spelayon	*T. bellinii* **sp. nov.**
15467	Brasil	MG	Barão de Cocais	MDIR-0028	-19 52 48.690	-43 26 13.599	19-23.VIII.2019	Equipe Spelayon	*T. bellinii* **sp. nov.**
15468**	Brasil	MG	Barão de Cocais	MDIR-0028	-19 52 48.690	-43 26 13.599	19-23.VIII.2019	Equipe Spelayon	*T. bellinii* **sp. nov.**
15469	Brasil	MG	Barão de Cocais	MDIR-0028	-19 52 48.690	-43 26 13.599	19-23.VIII.2019	Equipe Spelayon	*T. bellinii* **sp. nov.**
15471	Brasil	MG	Barão de Cocais	MDIR-0028	-19 52 48.690	-43 26 13.599	19-23.VIII.2019	Equipe Spelayon	*T. bellinii* **sp. nov.**
15474	Brasil	MG	Barão de Cocais	MDIR-0028	-19 52 48.690	-43 26 13.599	19-23.VIII.2019	Equipe Spelayon	*T. bellinii* **sp. nov.**
15475	Brasil	MG	Barão de Cocais	MDIR-0028	-19 52 48.690	-43 26 13.599	19-23.VIII.2019	Equipe Spelayon	*T. bellinii* **sp. nov.**
15476	Brasil	MG	Barão de Cocais	MDIR-0028	-19 52 48.690	-43 26 13.599	19-23.VIII.2019	Equipe Spelayon	*T. bellinii* **sp. nov.**
15477	Brasil	MG	Barão de Cocais	MDIR-0028	-19 52 48.690	-43 26 13.599	19-23.VIII.2019	Equipe Spelayon	*T. bellinii* **sp. nov.**
15478	Brasil	MG	Barão de Cocais	MDIR-0028	-19 52 48.690	-43 26 13.599	19-23.VIII.2019	Equipe Spelayon	*T. bellinii* **sp. nov.**
15480	Brasil	MG	Barão de Cocais	MDIR-0028	-19 52 48.690	-43 26 13.599	19-23.VIII.2019	Equipe Spelayon	*T. bellinii* **sp. nov.**
15481	Brasil	MG	Barão de Cocais	MDIR-0028	-19 52 48.690	-43 26 13.599	19-23.VIII.2019	Equipe Spelayon	*T. bellinii* **sp. nov.**
15482*	Brasil	MG	Barão de Cocais	MDIR-0028	-19 52 48.690	-43 26 13.599	19-23.VIII.2019	Equipe Spelayon	*T. bellinii* **sp. nov.**
15483**	Brasil	MG	Barão de Cocais	MDIR-0028	-19 52 48.690	-43 26 13.599	19-23.VIII.2019	Equipe Spelayon	*T. bellinii* **sp. nov.**
15484	Brasil	MG	Barão de Cocais	MDIR-0028	-19 52 48.690	-43 26 13.599	19-23.VIII.2019	Equipe Spelayon	*T. bellinii* **sp. nov.**
15485	Brasil	MG	Barão de Cocais	MDIR-0028	-19 52 48.690	-43 26 13.599	19-23.VIII.2019	Equipe Spelayon	*T. bellinii* **sp. nov.**
15486	Brasil	MG	Barão de Cocais	MDIR-0028	-19 52 48.690	-43 26 13.599	19-23.VIII.2019	Equipe Spelayon	*T. bellinii* **sp. nov.**
15487	Brasil	MG	Barão de Cocais	MDIR-0028	-19 52 48.690	-43 26 13.599	19-23.VIII.2019	Equipe Spelayon	*T. bellinii* **sp. nov.**
15488	Brasil	MG	Barão de Cocais	MDIR-0028	-19 52 48.690	-43 26 13.599	19-23.VIII.2019	Equipe Spelayon	*T. bellinii* **sp. nov.**
15489	Brasil	MG	Barão de Cocais	MDIR-0028	-19 52 48.690	-43 26 13.599	19-23.VIII.2019	Equipe Spelayon	*T. bellinii* **sp. nov.**
15490	Brasil	MG	Barão de Cocais	MDIR-0028	-19 52 48.690	-43 26 13.599	19-23.VIII.2019	Equipe Spelayon	*T. bellinii* **sp. nov.**
15491	Brasil	MG	Barão de Cocais	MDIR-0028	-19 52 48.690	-43 26 13.599	19-23.VIII.2019	Equipe Spelayon	*T. bellinii* **sp. nov.**
15501	Brasil	MG	Barão de Cocais	MDIR-0028	-19 52 48.690	-43 26 13.599	19-23.VIII.2019	Equipe Spelayon	*T. bellinii* **sp. nov.**
15502	Brasil	MG	Barão de Cocais	MDIR-0028	-19 52 48.690	-43 26 13.599	19-23.VIII.2019	Equipe Spelayon	*T. bellinii* **sp. nov.**
15503	Brasil	MG	Barão de Cocais	MDIR-0028	-19 52 48.690	-43 26 13.599	19-23.VIII.2019	Equipe Spelayon	*T. bellinii* **sp. nov.**
15504	Brasil	MG	Barão de Cocais	MDIR-0028	-19 52 48.690	-43 26 13.599	19-23.VIII.2019	Equipe Spelayon	*T. bellinii* **sp. nov.**
15505	Brasil	MG	Barão de Cocais	MDIR-0028	-19 52 48.690	-43 26 13.599	19-23.VIII.2019	Equipe Spelayon	*T. bellinii* **sp. nov.**
15508	Brasil	MG	Barão de Cocais	MDIR-0028	-19 52 48.690	-43 26 13.599	19-23.VIII.2019	Equipe Spelayon	*T. bellinii* **sp. nov.**
15509	Brasil	MG	Barão de Cocais	MDIR-0028	-19 52 48.690	-43 26 13.599	19-23.VIII.2019	Equipe Spelayon	*T. bellinii* **sp. nov.**
15510	Brasil	MG	Barão de Cocais	MDIR-0028	-19 52 48.690	-43 26 13.599	19-23.VIII.2019	Equipe Spelayon	*T. bellinii* **sp. nov.**
15511	Brasil	MG	Barão de Cocais	MDIR-0028	-19 52 48.690	-43 26 13.599	19-23.VIII.2019	Equipe Spelayon	*T. bellinii* **sp. nov.**
15513	Brasil	MG	Barão de Cocais	MDIR-0028	-19 52 48.690	-43 26 13.599	19-23.VIII.2019	Equipe Spelayon	*T. bellinii* **sp. nov.**
15514	Brasil	MG	Barão de Cocais	MDIR-0028	-19 52 48.690	-43 26 13.599	19-23.VIII.2019	Equipe Spelayon	*T. bellinii* **sp. nov.**
15515	Brasil	MG	Barão de Cocais	MDIR-0028	-19 52 48.690	-43 26 13.599	19-23.VIII.2019	Equipe Spelayon	*T. bellinii* **sp. nov.**
15516	Brasil	MG	Barão de Cocais	MDIR-0028	-19 52 48.690	-43 26 13.599	19-23.VIII.2019	Equipe Spelayon	*T. bellinii* **sp. nov.**
15517	Brasil	MG	Barão de Cocais	MDIR-0028	-19 52 48.690	-43 26 13.599	19-23.VIII.2019	Equipe Spelayon	*T. bellinii* **sp. nov.**
15518	Brasil	MG	Barão de Cocais	MDIR-0028	-19 52 48.690	-43 26 13.599	19-23.VIII.2019	Equipe Spelayon	*T. bellinii* **sp. nov.**
15519**	Brasil	MG	Barão de Cocais	MDIR-0028	-19 52 48.690	-43 26 13.599	19-23.VIII.2019	Equipe Spelayon	*T. bellinii* **sp. nov.**
15520	Brasil	MG	Barão de Cocais	MDIR-0028	-19 52 48.690	-43 26 13.599	19-23.VIII.2019	Equipe Spelayon	*T. bellinii* **sp. nov.**
15521	Brasil	MG	Barão de Cocais	MDIR-0028	-19 52 48.690	-43 26 13.599	19-23.VIII.2019	Equipe Spelayon	*T. bellinii* **sp. nov.**
15522	Brasil	MG	Barão de Cocais	MDIR-0028	-19 52 48.690	-43 26 13.599	19-23.VIII.2019	Equipe Spelayon	*T. bellinii* **sp. nov.**
15523	Brasil	MG	Barão de Cocais	MDIR-0028	-19 52 48.690	-43 26 13.599	19-23.VIII.2019	Equipe Spelayon	*T. bellinii* **sp. nov.**
15524	Brasil	MG	Barão de Cocais	MDIR-0028	-19 52 48.690	-43 26 13.599	19-23.VIII.2019	Equipe Spelayon	*T. bellinii* **sp. nov.**
15525	Brasil	MG	Barão de Cocais	MDIR-0028	-19 52 48.690	-43 26 13.599	19-23.VIII.2019	Equipe Spelayon	*T. bellinii* **sp. nov.**
15526	Brasil	MG	Barão de Cocais	MDIR-0028	-19 52 48.690	-43 26 13.599	19-23.VIII.2019	Equipe Spelayon	*T. bellinii* **sp. nov.**
15527	Brasil	MG	Barão de Cocais	MDIR-0028	-19 52 48.690	-43 26 13.599	19-23.VIII.2019	Equipe Spelayon	*T. bellinii* **sp. nov.**
15528	Brasil	MG	Barão de Cocais	MDIR-0028	-19 52 48.690	-43 26 13.599	19-23.VIII.2019	Equipe Spelayon	*T. bellinii* **sp. nov.**
15530	Brasil	MG	Barão de Cocais	MDIR-0028	-19 52 48.690	-43 26 13.599	19-23.VIII.2019	Equipe Spelayon	*T. bellinii* **sp. nov.**
15531	Brasil	MG	Barão de Cocais	MDIR-0028	-19 52 48.690	-43 26 13.599	19-23.VIII.2019	Equipe Spelayon	*T. bellinii* **sp. nov.**
15532	Brasil	MG	Barão de Cocais	MDIR-0028	-19 52 48.690	-43 26 13.599	19-23.VIII.2019	Equipe Spelayon	*T. bellinii* **sp. nov.**
15533	Brasil	MG	Barão de Cocais	MDIR-0028	-19 52 48.690	-43 26 13.599	19-23.VIII.2019	Equipe Spelayon	*T. bellinii* **sp. nov.**
15534	Brasil	MG	Barão de Cocais	MDIR-0028	-19 52 48.690	-43 26 13.599	19-23.VIII.2019	Equipe Spelayon	*T. bellinii* **sp. nov.**
15536	Brasil	MG	Barão de Cocais	MDIR-0028	-19 52 48.690	-43 26 13.599	19-23.VIII.2019	Equipe Spelayon	*T. bellinii* **sp. nov.**
15537	Brasil	MG	Barão de Cocais	MDIR-0028	-19 52 48.690	-43 26 13.599	19-23.VIII.2019	Equipe Spelayon	*T. bellinii* **sp. nov.**
15538	Brasil	MG	Barão de Cocais	MDIR-0028	-19 52 48.690	-43 26 13.599	19-23.VIII.2019	Equipe Spelayon	*T. bellinii* **sp. nov.**

15539	Brasil	MG	Barão de Cocais	MDIR-0028	-19 52 48.690	-43 26 13.599	19-23.VIII.2019	Equipe Spelayon	*T. bellinii* **sp. nov.**
15540	Brasil	MG	Barão de Cocais	MDIR-0028	-19 52 48.690	-43 26 13.599	19-23.VIII.2019	Equipe Spelayon	*T. bellinii* **sp. nov.**
15541	Brasil	MG	Barão de Cocais	MDIR-0028	-19 52 48.690	-43 26 13.599	19-23.VIII.2019	Equipe Spelayon	*T. bellinii* **sp. nov.**
15542	Brasil	MG	Barão de Cocais	MDIR-0028	-19 52 48.690	-43 26 13.599	19-23.VIII.2019	Equipe Spelayon	*T. bellinii* **sp. nov.**
15562	Brasil	MG	Barão de Cocais	MDIR-0028	-19 52 48.690	-43 26 13.599	19-23.VIII.2019	Equipe Spelayon	*T. bellinii* **sp. nov.**
15563	Brasil	MG	Barão de Cocais	MDIR-0028	-19 52 48.690	-43 26 13.599	19-23.VIII.2019	Equipe Spelayon	*T. bellinii* **sp. nov.**
15564	Brasil	MG	Barão de Cocais	MDIR-0028	-19 52 48.690	-43 26 13.599	19-23.VIII.2019	Equipe Spelayon	*T. bellinii* **sp. nov.**
15565	Brasil	MG	Barão de Cocais	MDIR-0028	-19 52 48.690	-43 26 13.599	19-23.VIII.2019	Equipe Spelayon	*T. bellinii* **sp. nov.**
15566	Brasil	MG	Barão de Cocais	MDIR-0028	-19 52 48.690	-43 26 13.599	19-23.VIII.2019	Equipe Spelayon	*T. bellinii* **sp. nov.**
15569	Brasil	MG	Barão de Cocais	MDIR-0028	-19 52 48.690	-43 26 13.599	19-23.VIII.2019	Equipe Spelayon	*T. bellinii* **sp. nov.**
15570	Brasil	MG	Barão de Cocais	MDIR-0028	-19 52 48.690	-43 26 13.599	19-23.VIII.2019	Equipe Spelayon	*T. bellinii* **sp. nov.**
15571	Brasil	MG	Barão de Cocais	MDIR-0028	-19 52 48.690	-43 26 13.599	19-23.VIII.2019	Equipe Spelayon	*T. bellinii* **sp. nov.**
15573	Brasil	MG	Barão de Cocais	MDIR-0028	-19 52 48.690	-43 26 13.599	19-23.VIII.2019	Equipe Spelayon	*T. bellinii* **sp. nov.**
15574	Brasil	MG	Barão de Cocais	MDIR-0028	-19 52 48.690	-43 26 13.599	19-23.VIII.2019	Equipe Spelayon	*T. bellinii* **sp. nov.**
15575	Brasil	MG	Barão de Cocais	MDIR-0028	-19 52 48.690	-43 26 13.599	19-23.VIII.2019	Equipe Spelayon	*T. bellinii* **sp. nov.**
15576**	Brasil	MG	Barão de Cocais	MDIR-0028	-19 52 48.690	-43 26 13.599	19-23.VIII.2019	Equipe Spelayon	*T. bellinii* **sp. nov.**
15577	Brasil	MG	Barão de Cocais	MDIR-0028	-19 52 48.690	-43 26 13.599	19-23.VIII.2019	Equipe Spelayon	*T. bellinii* **sp. nov.**
15579	Brasil	MG	Barão de Cocais	MDIR-0028	-19 52 48.690	-43 26 13.599	19-23.VIII.2019	Equipe Spelayon	*T. bellinii* **sp. nov.**
15580	Brasil	MG	Barão de Cocais	MDIR-0028	-19 52 48.690	-43 26 13.599	19-23.VIII.2019	Equipe Spelayon	*T. bellinii* **sp. nov.**
15581	Brasil	MG	Barão de Cocais	MDIR-0028	-19 52 48.690	-43 26 13.599	19-23.VIII.2019	Equipe Spelayon	*T. bellinii* **sp. nov.**
15582	Brasil	MG	Barão de Cocais	MDIR-0028	-19 52 48.690	-43 26 13.599	19-23.VIII.2019	Equipe Spelayon	*T. bellinii* **sp. nov.**
15583	Brasil	MG	Barão de Cocais	MDIR-0028	-19 52 48.690	-43 26 13.599	19-23.VIII.2019	Equipe Spelayon	*T. bellinii* **sp. nov.**
15584	Brasil	MG	Barão de Cocais	MDIR-0028	-19 52 48.690	-43 26 13.599	19-23.VIII.2019	Equipe Spelayon	*T. bellinii* **sp. nov.**
15586	Brasil	MG	Barão de Cocais	MDIR-0028	-19 52 48.690	-43 26 13.599	19-23.VIII.2019	Equipe Spelayon	*T. bellinii* **sp. nov.**
15587	Brasil	MG	Barão de Cocais	MDIR-0028	-19 52 48.690	-43 26 13.599	19-23.VIII.2019	Equipe Spelayon	*T. bellinii* **sp. nov.**
15589	Brasil	MG	Barão de Cocais	MDIR-0028	-19 52 48.690	-43 26 13.599	19-23.VIII.2019	Equipe Spelayon	*T. bellinii* **sp. nov.**
15590	Brasil	MG	Barão de Cocais	MDIR-0028	-19 52 48.690	-43 26 13.599	19-23.VIII.2019	Equipe Spelayon	*T. bellinii* **sp. nov.**
15591	Brasil	MG	Barão de Cocais	MDIR-0028	-19 52 48.690	-43 26 13.599	19-23.VIII.2019	Equipe Spelayon	*T. bellinii* **sp. nov.**
15592	Brasil	MG	Barão de Cocais	MDIR-0028	-19 52 48.690	-43 26 13.599	19-23.VIII.2019	Equipe Spelayon	*T. bellinii* **sp. nov.**
15593	Brasil	MG	Barão de Cocais	MDIR-0028	-19 52 48.690	-43 26 13.599	19-23.VIII.2019	Equipe Spelayon	*T. bellinii* **sp. nov.**
15594	Brasil	MG	Barão de Cocais	MDIR-0028	-19 52 48.690	-43 26 13.599	19-23.VIII.2019	Equipe Spelayon	*T. bellinii* **sp. nov.**
15596	Brasil	MG	Barão de Cocais	MDIR-0028	-19 52 48.690	-43 26 13.599	19-23.VIII.2019	Equipe Spelayon	*T. bellinii* **sp. nov.**
15597	Brasil	MG	Barão de Cocais	MDIR-0028	-19 52 48.690	-43 26 13.599	19-23.VIII.2019	Equipe Spelayon	*T. bellinii* **sp. nov.**
15598	Brasil	MG	Barão de Cocais	MDIR-0028	-19 52 48.690	-43 26 13.599	19-23.VIII.2019	Equipe Spelayon	*T. bellinii* **sp. nov.**
15599	Brasil	MG	Barão de Cocais	MDIR-0028	-19 52 48.690	-43 26 13.599	19-23.VIII.2019	Equipe Spelayon	*T. bellinii* **sp. nov.**
15600	Brasil	MG	Barão de Cocais	MDIR-0028	-19 52 48.690	-43 26 13.599	19-23.VIII.2019	Equipe Spelayon	*T. bellinii* **sp. nov.**
15601	Brasil	MG	Barão de Cocais	MDIR-0028	-19 52 48.690	-43 26 13.599	19-23.VIII.2019	Equipe Spelayon	*T. bellinii* **sp. nov.**
15602	Brasil	MG	Barão de Cocais	MDIR-0028	-19 52 48.690	-43 26 13.599	19-23.VIII.2019	Equipe Spelayon	*T. bellinii* **sp. nov.**
15604	Brasil	MG	Barão de Cocais	MDIR-0028	-19 52 48.690	-43 26 13.599	19-23.VIII.2019	Equipe Spelayon	*T. bellinii* **sp. nov.**
15605	Brasil	MG	Barão de Cocais	MDIR-0028	-19 52 48.690	-43 26 13.599	19-23.VIII.2019	Equipe Spelayon	*T. bellinii* **sp. nov.**
15606	Brasil	MG	Barão de Cocais	MDIR-0028	-19 52 48.690	-43 26 13.599	19-23.VIII.2019	Equipe Spelayon	*T. bellinii* **sp. nov.**
15607	Brasil	MG	Barão de Cocais	MDIR-0028	-19 52 48.690	-43 26 13.599	19-23.VIII.2019	Equipe Spelayon	*T. bellinii* **sp. nov.**
15608	Brasil	MG	Barão de Cocais	MDIR-0028	-19 52 48.690	-43 26 13.599	19-23.VIII.2019	Equipe Spelayon	*T. bellinii* **sp. nov.**
15609	Brasil	MG	Barão de Cocais	MDIR-0028	-19 52 48.690	-43 26 13.599	19-23.VIII.2019	Equipe Spelayon	*T. bellinii* **sp. nov.**
15610	Brasil	MG	Barão de Cocais	MDIR-0028	-19 52 48.690	-43 26 13.599	19-23.VIII.2019	Equipe Spelayon	*T. bellinii* **sp. nov.**
15611	Brasil	MG	Barão de Cocais	MDIR-0028	-19 52 48.690	-43 26 13.599	19-23.VIII.2019	Equipe Spelayon	*T. bellinii* **sp. nov.**
15612	Brasil	MG	Barão de Cocais	MDIR-0028	-19 52 48.690	-43 26 13.599	19-23.VIII.2019	Equipe Spelayon	*T. bellinii* **sp. nov.**
15613	Brasil	MG	Barão de Cocais	MDIR-0028	-19 52 48.690	-43 26 13.599	19-23.VIII.2019	Equipe Spelayon	*T. bellinii* **sp. nov.**
15614	Brasil	MG	Barão de Cocais	MDIR-0028	-19 52 48.690	-43 26 13.599	19-23.VIII.2019	Equipe Spelayon	*T. bellinii* **sp. nov.**
15615	Brasil	MG	Barão de Cocais	MDIR-0028	-19 52 48.690	-43 26 13.599	19-23.VIII.2019	Equipe Spelayon	*T. bellinii* **sp. nov.**
15616	Brasil	MG	Barão de Cocais	MDIR-0028	-19 52 48.690	-43 26 13.599	19-23.VIII.2019	Equipe Spelayon	*T. bellinii* **sp. nov.**
15617	Brasil	MG	Barão de Cocais	MDIR-0028	-19 52 48.690	-43 26 13.599	19-23.VIII.2019	Equipe Spelayon	*T. bellinii* **sp. nov.**
15618	Brasil	MG	Barão de Cocais	MDIR-0028	-19 52 48.690	-43 26 13.599	19-23.VIII.2019	Equipe Spelayon	*T. bellinii* **sp. nov.**
15619	Brasil	MG	Barão de Cocais	MDIR-0028	-19 52 48.690	-43 26 13.599	19-23.VIII.2019	Equipe Spelayon	*T. bellinii* **sp. nov.**
15621	Brasil	MG	Barão de Cocais	MDIR-0028	-19 52 48.690	-43 26 13.599	19-23.VIII.2019	Equipe Spelayon	*T. bellinii* **sp. nov.**
15622	Brasil	MG	Barão de Cocais	MDIR-0028	-19 52 48.690	-43 26 13.599	19-23.VIII.2019	Equipe Spelayon	*T. bellinii* **sp. nov.**
15624	Brasil	MG	Barão de Cocais	MDIR-0028	-19 52 48.690	-43 26 13.599	19-23.VIII.2019	Equipe Spelayon	*T. bellinii* **sp. nov.**

15625	Brasil	MG	Barão de Cocais	MDIR-0028	-19 52 48.690	-43 26 13.599	19-23.VIII.2019	Equipe Spelayon	*T. bellinii* **sp. nov.**
15626	Brasil	MG	Barão de Cocais	MDIR-0028	-19 52 48.690	-43 26 13.599	19-23.VIII.2019	Equipe Spelayon	*T. bellinii* **sp. nov.**
15627	Brasil	MG	Barão de Cocais	MDIR-0028	-19 52 48.690	-43 26 13.599	19-23.VIII.2019	Equipe Spelayon	*T. bellinii* **sp. nov.**
15628	Brasil	MG	Barão de Cocais	MDIR-0028	-19 52 48.690	-43 26 13.599	19-23.VIII.2019	Equipe Spelayon	*T. bellinii* **sp. nov.**
15629	Brasil	MG	Barão de Cocais	MDIR-0028	-19 52 48.690	-43 26 13.599	19-23.VIII.2019	Equipe Spelayon	*T. bellinii* **sp. nov.**
15630	Brasil	MG	Barão de Cocais	MDIR-0028	-19 52 48.690	-43 26 13.599	19-23.VIII.2019	Equipe Spelayon	*T. bellinii* **sp. nov.**
15631	Brasil	MG	Barão de Cocais	MDIR-0028	-19 52 48.690	-43 26 13.599	19-23.VIII.2019	Equipe Spelayon	*T. bellinii* **sp. nov.**
15632	Brasil	MG	Barão de Cocais	MDIR-0028	-19 52 48.690	-43 26 13.599	19-23.VIII.2019	Equipe Spelayon	*T. bellinii* **sp. nov.**
15633	Brasil	MG	Barão de Cocais	MDIR-0028	-19 52 48.690	-43 26 13.599	19-23.VIII.2019	Equipe Spelayon	*T. bellinii* **sp. nov.**
15634	Brasil	MG	Barão de Cocais	MDIR-0028	-19 52 48.690	-43 26 13.599	19-23.VIII.2019	Equipe Spelayon	*T. bellinii* **sp. nov.**
15635	Brasil	MG	Barão de Cocais	BRU-0005	-19 52 13.033	-43 24 14.902	19-23.VIII.2019	Equipe Spelayon	*T. bellinii* **sp. nov.**
15646	Brasil	MG	Barão de Cocais	BRU-0005	-19 52 13.033	-43 24 14.902	19-23.VIII.2019	Equipe Spelayon	*T. bellinii* **sp. nov.**
15647	Brasil	MG	Barão de Cocais	BRU-0005	-19 52 13.033	-43 24 14.902	19-23.VIII.2019	Equipe Spelayon	*T. bellinii* **sp. nov.**
15648	Brasil	MG	Barão de Cocais	BRU-0005	-19 52 13.033	-43 24 14.902	19-23.VIII.2019	Equipe Spelayon	*T. bellinii* **sp. nov.**
15649	Brasil	MG	Barão de Cocais	BRU-0005	-19 52 13.033	-43 24 14.902	19-23.VIII.2019	Equipe Spelayon	*T. bellinii* **sp. nov.**
15650	Brasil	MG	Barão de Cocais	BRU-0005	-19 52 13.033	-43 24 14.902	19-23.VIII.2019	Equipe Spelayon	*T. bellinii* **sp. nov.**
15651	Brasil	MG	Barão de Cocais	BRU-0005	-19 52 13.033	-43 24 14.902	19-23.VIII.2019	Equipe Spelayon	*T. bellinii* **sp. nov.**
15654	Brasil	MG	Barão de Cocais	BRU-0005	-19 52 13.033	-43 24 14.902	19-23.VIII.2019	Equipe Spelayon	*T. bellinii* **sp. nov.**
15657	Brasil	MG	Barão de Cocais	BRU-0005	-19 52 13.033	-43 24 14.902	19-23.VIII.2019	Equipe Spelayon	*T. bellinii* **sp. nov.**
15658	Brasil	MG	Barão de Cocais	BRU-0005	-19 52 13.033	-43 24 14.902	19-23.VIII.2019	Equipe Spelayon	*T. bellinii* **sp. nov.**
15666	Brasil	MG	Barão de Cocais	BRU-0005	-19 52 13.033	-43 24 14.902	19-23.VIII.2019	Equipe Spelayon	*T. bellinii* **sp. nov.**
15667	Brasil	MG	Barão de Cocais	BRU-0005	-19 52 13.033	-43 24 14.902	19-23.VIII.2019	Equipe Spelayon	*T. bellinii* **sp. nov.**
15668	Brasil	MG	Barão de Cocais	BRU-0005	-19 52 13.033	-43 24 14.902	19-23.VIII.2019	Equipe Spelayon	*T. bellinii* **sp. nov.**
15670	Brasil	MG	Barão de Cocais	BRU-0034	-19 51 1.965	-43 22 40.574	19-23.VIII.2019	Equipe Spelayon	*T. bellinii* **sp. nov.**
15672	Brasil	MG	Barão de Cocais	BRU-0008	-19 52 32.054	-43 25 1.809	19-23.VIII.2019	Equipe Spelayon	*T. bellinii* **sp. nov.**
15673	Brasil	MG	Barão de Cocais	BRU-0008	-19 52 32.054	-43 25 1.809	19-23.VIII.2019	Equipe Spelayon	*T. bellinii* **sp. nov.**
15674	Brasil	MG	Barão de Cocais	BRU-0008	-19 52 32.054	-43 25 1.809	19-23.VIII.2019	Equipe Spelayon	*T. bellinii* **sp. nov.**
15675	Brasil	MG	Barão de Cocais	BRU-0008	-19 52 32.054	-43 25 1.809	19-23.VIII.2019	Equipe Spelayon	*T. bellinii* **sp. nov.**
15679	Brasil	MG	Barão de Cocais	BRU-0008	-19 52 32.054	-43 25 1.809	19-23.VIII.2019	Equipe Spelayon	*T. bellinii* **sp. nov.**
15680	Brasil	MG	Barão de Cocais	BRU-0008	-19 52 32.054	-43 25 1.809	19-23.VIII.2019	Equipe Spelayon	*T. bellinii* **sp. nov.**
15681	Brasil	MG	Barão de Cocais	BRU-0008	-19 52 32.054	-43 25 1.809	19-23.VIII.2019	Equipe Spelayon	*T. bellinii* **sp. nov.**
15682	Brasil	MG	Barão de Cocais	BRU-0008	-19 52 32.054	-43 25 1.809	19-23.VIII.2019	Equipe Spelayon	*T. bellinii* **sp. nov.**
15683	Brasil	MG	Barão de Cocais	BRU-0008	-19 52 32.054	-43 25 1.809	19-23.VIII.2019	Equipe Spelayon	*T. bellinii* **sp. nov.**
16493	Brasil	MG	Barão de Cocais	BRU-0035	-19 53 19.770	-43 25 2.332	18.V.2020	Equipe Spelayon	*T. bellinii* **sp. nov.**
16494	Brasil	MG	Barão de Cocais	BRU-0035	-19 53 19.770	-43 25 2.332	18.V.2020	Equipe Spelayon	*T. bellinii* **sp. nov.**
16495	Brasil	MG	Barão de Cocais	BRU-0035	-19 53 19.770	-43 25 2.332	18.V.2020	Equipe Spelayon	*T. bellinii* **sp. nov.**
16520	Brasil	MG	Barão de Cocais	BRU-0002	-19 53 19.824	-43 26 14.772	16.V.2020	Equipe Spelayon	*T. bellinii* **sp. nov.**
16521	Brasil	MG	Barão de Cocais	BRU-0002	-19 53 19.824	-43 26 14.772	16.V.2020	Equipe Spelayon	*T. bellinii* **sp. nov.**
16522	Brasil	MG	Barão de Cocais	BRU-0002	-19 53 19.824	-43 26 14.772	16.V.2020	Equipe Spelayon	*T. bellinii* **sp. nov.**
16523	Brasil	MG	Barão de Cocais	BRU-0002	-19 53 19.824	-43 26 14.772	16.V.2020	Equipe Spelayon	*T. bellinii* **sp. nov.**
16524	Brasil	MG	Barão de Cocais	BRU-0002	-19 53 19.824	-43 26 14.772	16.V.2020	Equipe Spelayon	*T. bellinii* **sp. nov.**
14066	Brasil	MG	Brumadinho	PBR-0007	-20 09 51.116	-43 58 33.979	26.III-05.IV.2019	Equipe Carste	*T. bellinii* **sp. nov.**
14068	Brasil	MG	Brumadinho	PBR-0007	-20 09 51.116	-43 58 33.979	26.III-05.IV.2019	Equipe Carste	*T. bellinii* **sp. nov.**
14069	Brasil	MG	Brumadinho	PBR-0007	-20 09 51.116	-43 58 33.979	26.III-05.IV.2019	Equipe Carste	*T. bellinii* **sp. nov.**
14070	Brasil	MG	Brumadinho	PBR-0007	-20 09 51.116	-43 58 33.979	26.III-05.IV.2019	Equipe Carste	*T. bellinii* **sp. nov.**
14071	Brasil	MG	Brumadinho	PBR-0007	-20 09 51.116	-43 58 33.979	26.III-05.IV.2019	Equipe Carste	*T. bellinii* **sp. nov.**
14072	Brasil	MG	Brumadinho	PBR-0007	-20 09 51.116	-43 58 33.979	26.III-05.IV.2019	Equipe Carste	*T. bellinii* **sp. nov.**
15731	Brasil	MG	Brumadinho	PBR - 0021	-20 09 50.481	-43 58 36.773	9-20.IV.2018	Equipe Carste	*T. bellinii* **sp. nov.**
15732	Brasil	MG	Brumadinho	PBR - 0021	-20 09 50.481	-43 58 36.773	9-20.IV.2018	Equipe Carste	*T. bellinii* **sp. nov.**
15741	Brasil	MG	Brumadinho	PBR - 0007	-20 09 51.116	-43 58 33.979	12-14.III.2018	Equipe Carste	*T. bellinii* **sp. nov.**
15747	Brasil	MG	Brumadinho	PBR - 0025	-20 09 49.935	-43 58 37.914	09-20.IV.2018	Equipe Carste	*T. bellinii* **sp. nov.**
15749	Brasil	MG	Brumadinho	PBR - 0025	-20 09 49.935	-43 58 37.914	09-20.IV.2018	Equipe Carste	*T. bellinii* **sp. nov.**
15750	Brasil	MG	Brumadinho	PBR - 0025	-20 09 49.935	-43 58 37.914	09-20.IV.2018	Equipe Carste	*T. bellinii* **sp. nov.**
15754	Brasil	MG	Brumadinho	PBR - 0032	-20 09 31.050	-43 59 25.227	09-20.IV.2018	Equipe Carste	*T. bellinii* **sp. nov.**
15988	Brasil	MG	Brumadinho	PBR-0007	-20 09 51.116	-43 58 33.979	26.IX-04.X.2019	Equipe Carste	*T. bellinii* **sp. nov.**
12517	Brasil	MG	Itambé do Mato Dentro	MLog	-19 19 54.192	-43 18 27.324	26.VI.06.VII.2018	Equipe Carste	*T. bellinii* **sp. nov.**
11584	Brasil	MG	Matozinhos	?	-19 30 30.421	-44 06 53.239	25.III-11.IV.2013	Equipe Bueno	*T. bellinii* **sp. nov.**

6515	Brasil	MG	Pedro Leopoldo	?	-19 34 44.187	-44 01 0.373	04-20.III.2015	Equipe Carste	*T. bellinii* **sp. nov.**
6516	Brasil	MG	Pedro Leopoldo	?	-19 34 44.187	-44 01 0.373	04-20.III.2015	Equipe Carste	*T. bellinii* **sp. nov.**
6535	Brasil	MG	Pedro Leopoldo	?	-19 34 20.732	-44 00 42.292	04-20.III.2015	Equipe Carste	*T. bellinii* **sp. nov.**
6536	Brasil	MG	Pedro Leopoldo	?	-19 34 40.868	-44 00 54.250	04-20.III.2015	Equipe Carste	*T. bellinii* **sp. nov.**
6539	Brasil	MG	Pedro Leopoldo	?	-19 34 44.187	-44 01 0.373	04-20.III.2015	Equipe Carste	*T. bellinii* **sp. nov.**
6540	Brasil	MG	Pedro Leopoldo	?	-19 34 12.409	-44 00 43.269	04-20.III.2015	Equipe Carste	*T. bellinii* **sp. nov.**
7282	Brasil	MG	Pedro Leopoldo	?	-19 34 44.187	-44 01 0.373	22.VII-18.VIII.2015	Equipe Carste	*T. bellinii* **sp. nov.**
7283	Brasil	MG	Pedro Leopoldo	?	-19 34 44.188	-44 01 0.374	22.VII-18.VIII.2015	Equipe Carste	*T. bellinii* **sp. nov.**
9139	Brasil	MG	Pedro Leopoldo	?	-19 34 12.701	-44 0 37.124	22.VII-18.VIII.2015	Equipe Carste	*T. bellinii* **sp. nov.**
9141	Brasil	MG	Pedro Leopoldo	?	-19 34 12.701	-44 0 37.124	22.VII-18.VIII.2015	Equipe Carste	*T. bellinii* **sp. nov.**
9142	Brasil	MG	Pedro Leopoldo	?	-19 34 12.701	-44 0 37.124	22.VII-18.VIII.2015	Equipe Carste	*T. bellinii* **sp. nov.**
9152	Brasil	MG	Pedro Leopoldo	?	-19 34 11.352	-44 00 40.358	22.VII-18.VIII.2015	Equipe Carste	*T. bellinii* **sp. nov.**
11419	Brasil	MG	Pedro Leopoldo	HOLC - 042	-19 34 39.435	-44 0 47.978	27.VI-01VII.2016	Equipe Carste	*T. bellinii* **sp. nov.**
11420	Brasil	MG	Pedro Leopoldo	HOLC - 042	-19 34 39.435	-44 0 47.978	27.VI-01VII.2016	Equipe Carste	*T. bellinii* **sp. nov.**
11421	Brasil	MG	Pedro Leopoldo	HOLC - 042	-19 34 39.859	-44 0 48.215	15-18.II.2016	Equipe Carste	*T. bellinii* **sp. nov.**
11422	Brasil	MG	Pedro Leopoldo	HOLC - 042	-19 34 39.859	-44 0 48.215	15-18.II.2016	Equipe Carste	*T. bellinii* **sp. nov.**
11455	Brasil	MG	Pedro Leopoldo	HOLC - 107	-19 35 3.557	-43 59 57.996	02.XII.2016	Equipe Carste	*T. bellinii* **sp. nov.**
11456	Brasil	MG	Pedro Leopoldo	HOLC - 107	-19 35 3.557	-43 59 57.996	27.VI-01VII.2016	Equipe Carste	*T. bellinii* **sp. nov.**
11460	Brasil	MG	Pedro Leopoldo	HOLC - 106	-19 35 3.427	-43 59 58.031	02-14.VI.2016	Equipe Carste	*T. bellinii* **sp. nov.**
11474	Brasil	MG	Pedro Leopoldo	CAMP-13	-19 34 4.227	-44 00 28.322	3-21.XI.2014	Equipe Spelayon	*T. bellinii* **sp. nov.**
15328	Brasil	MG	Prudente de Morais	Gruta 01L - Zona Afótica	-19 26 32.479	-44 06 24.251	04.III.2019	Equipe Zampaulo & Reis	*T. bellinii* **sp. nov.**
15330	Brasil	MG	Prudente de Morais	Gruta 01L - Zona Afótica	-19 26 32.479	-44 06 24.251	04.III.2019	Equipe Zampaulo & Reis	*T. bellinii* **sp. nov.**
15332	Brasil	MG	Prudente de Morais	Gruta 01L - Zona Afótica	-19 26 32.479	-44 06 24.251	04.III.2019	Equipe Zampaulo & Reis	*T. bellinii* **sp. nov.**
15333	Brasil	MG	Prudente de Morais	Gruta 01L - Zona Afótica	-19 26 32.479	-44 06 24.251	04.III.2019	Equipe Zampaulo & Reis	*T. bellinii* **sp. nov.**
15360	Brasil	MG	Prudente de Morais	Gruta 06L - Zona afótica	-19 26 28.946	-44 06 26.945	17.III.2019	Equipe Zampaulo & Reis	*T. bellinii* **sp. nov.**
15362	Brasil	MG	Prudente de Morais	Gruta 34L - Zona Afótica	-19 26 24.863	-44 06 23.469	04.III.2019	Equipe Zampaulo & Reis	*T. bellinii* **sp. nov.**
15363	Brasil	MG	Prudente de Morais	Gruta 34L - Zona Afótica	-19 26 24.863	-44 06 23.469	04.III.2019	Equipe Zampaulo & Reis	*T. bellinii* **sp. nov.**
15365	Brasil	MG	Prudente de Morais	Gruta 34L - Zona Afótica	-19 26 24.863	-44 06 23.469	04.III.2019	Equipe Zampaulo & Reis	*T. bellinii* **sp. nov.**
15366	Brasil	MG	Prudente de Morais	Gruta 34L - Zona Afótica	-19 26 24.863	-44 06 23.469	04.III.2019	Equipe Zampaulo & Reis	*T. bellinii* **sp. nov.**
15373	Brasil	MG	Prudente de Morais	Gruta 40L - Zona Afótica	-19 26 24.945	-44 06 20.383	16.III.2019	Equipe Zampaulo & Reis	*T. bellinii* **sp. nov.**
15374	Brasil	MG	Prudente de Morais	Gruta 40L - Zona Afótica	-19 26 24.945	-44 06 20.383	16.III.2019	Equipe Zampaulo & Reis	*T. bellinii* **sp. nov.**
15375	Brasil	MG	Prudente de Morais	Gruta 40L - Zona Afótica	-19 26 24.945	-44 06 20.383	16.III.2019	Equipe Zampaulo & Reis	*T. bellinii* **sp. nov.**
15378	Brasil	MG	Prudente de Morais	Gruta 40L - Zona Afótica	-19 26 24.945	-44 06 20.383	16.III.2019	Equipe Zampaulo & Reis	*T. bellinii* **sp. nov.**
15380	Brasil	MG	Prudente de Morais	Gruta 40L - Zona Afótica	-19 26 24.945	-44 06 20.383	16.III.2019	Equipe Zampaulo & Reis	*T. bellinii* **sp. nov.**
15381	Brasil	MG	Prudente de Morais	Gruta 40L - Zona Afótica	-19 26 24.945	-44 06 20.383	16.III.2019	Equipe Zampaulo & Reis	*T. bellinii* **sp. nov.**
15382	Brasil	MG	Prudente de Morais	Gruta 40L - Zona Afótica	-19 26 24.945	-44 06 20.383	16.III.2019	Equipe Zampaulo & Reis	*T. bellinii* **sp. nov.**
15383	Brasil	MG	Prudente de Morais	Gruta 40L - Zona Afótica	-19 26 24.945	-44 06 20.383	16.III.2019	Equipe Zampaulo & Reis	*T. bellinii* **sp. nov.**
15384	Brasil	MG	Prudente de Morais	Gruta 40L - Zona Afótica	-19 26 24.945	-44 06 20.383	16.III.2019	Equipe Zampaulo & Reis	*T. bellinii* **sp. nov.**
15385	Brasil	MG	Prudente de Morais	Gruta 40L - Zona Afótica	-19 26 24.945	-44 06 20.383	16.III.2019	Equipe Zampaulo & Reis	*T. bellinii* **sp. nov.**
15386	Brasil	MG	Prudente de Morais	Gruta 40L - Zona Afótica	-19 26 24.945	-44 06 20.383	16.III.2019	Equipe Zampaulo & Reis	*T. bellinii* **sp. nov.**
15389	Brasil	MG	Prudente de Morais	Gruta 51L - Zona Entrada	-19 26 23.510	-44 06 13.052	09.III.2019	Equipe Zampaulo & Reis	*T. bellinii* **sp. nov.**
15390	Brasil	MG	Prudente de Morais	Gruta 101L - Zona afótica	-19 26 28.946	-44 06 26.945	09.III.2019	Equipe Zampaulo & Reis	*T. bellinii* **sp. nov.**
15391	Brasil	MG	Prudente de Morais	Gruta 101L - Zona afótica	-19 26 28.946	-44 06 26.945	09.III.2019	Equipe Zampaulo & Reis	*T. bellinii* **sp. nov.**
15392	Brasil	MG	Prudente de Morais	Gruta 101L - Zona afótica	-19 26 28.946	-44 06 26.945	09.III.2019	Equipe Zampaulo & Reis	*T. bellinii* **sp. nov.**
15396	Brasil	MG	Prudente de Morais	Gruta 58L - Zona Afótica	-19 26 26.004	-44 06 4.157	09.III.2019	Equipe Zampaulo & Reis	*T. bellinii* **sp. nov.**
15400	Brasil	MG	Prudente de Morais	Gruta ES17 - Zona Afótica	-19 26 45.097	-44 06 10.190	08.III.2019	Equipe Zampaulo & Reis	*T. bellinii* **sp. nov.**
15401	Brasil	MG	Prudente de Morais	Gruta ES17 - Zona Afótica	-19 26 45.097	-44 06 10.190	08.III.2019	Equipe Zampaulo & Reis	*T. bellinii* **sp. nov.**
15402	Brasil	MG	Prudente de Morais	Gruta ES17 - Zona Afótica	-19 26 45.097	-44 06 10.190	08.III.2019	Equipe Zampaulo & Reis	*T. bellinii* **sp. nov.**
15403	Brasil	MG	Prudente de Morais	Gruta ES17 - Zona Afótica	-19 26 45.097	-44 06 10.190	08.III.2019	Equipe Zampaulo & Reis	*T. bellinii* **sp. nov.**
15404	Brasil	MG	Prudente de Morais	Gruta ES17 - Zona Afótica	-19 26 45.097	-44 06 10.190	08.III.2019	Equipe Zampaulo & Reis	*T. bellinii* **sp. nov.**
15405	Brasil	MG	Prudente de Morais	Gruta ES17 - Zona Afótica	-19 26 45.097	-44 06 10.190	08.III.2019	Equipe Zampaulo & Reis	*T. bellinii* **sp. nov.**
15406	Brasil	MG	Prudente de Morais	Gruta ES17 - Zona Afótica	-19 26 45.097	-44 06 10.190	08.III.2019	Equipe Zampaulo & Reis	*T. bellinii* **sp. nov.**
15407	Brasil	MG	Prudente de Morais	Gruta ES17 - Zona Entrada	-19 26 45.097	-44 06 10.190	08.III.2019	Equipe Zampaulo & Reis	*T. bellinii* **sp. nov.**
15408	Brasil	MG	Prudente de Morais	Gruta ES17 - Zona Entrada	-19 26 45.097	-44 06 10.190	08.III.2019	Equipe Zampaulo & Reis	*T. bellinii* **sp. nov.**
15409	Brasil	MG	Prudente de Morais	Gruta ES17 - Zona Entrada	-19 26 45.097	-44 06 10.190	08.III.2019	Equipe Zampaulo & Reis	*T. bellinii* **sp. nov.**
15410	Brasil	MG	Prudente de Morais	Gruta ES17 - Zona Entrada	-19 26 45.097	-44 06 10.190	08.III.2019	Equipe Zampaulo & Reis	*T. bellinii* **sp. nov.**
15411	Brasil	MG	Prudente de Morais	Gruta ES17 - Zona Entrada	-19 26 45.097	-44 06 10.190	08.III.2019	Equipe Zampaulo & Reis	*T. bellinii* **sp. nov.**

15412	Brasil	MG	Prudente de Morais	Gruta ES17 - Zona Entrada	-19 26 45.097	-44 06 10.190	08.III.2019	Equipe Zampaulo & Reis	*T. bellinii* **sp. nov.**
15413	Brasil	MG	Prudente de Morais	Gruta ES17 - Zona Entrada	-19 26 45.097	-44 06 10.190	08.III.2019	Equipe Zampaulo & Reis	*T. bellinii* **sp. nov.**
15421	Brasil	MG	Prudente de Morais	Gruta ES01 - Zona Entrada	-19 26 45.168	-44 06 11.390	01.III.2019	Equipe Zampaulo & Reis	*T. bellinii* **sp. nov.**
15436	Brasil	MG	Prudente de Morais	Gruta ES13 - Zona Afótica	-19 26 45.766	-44 06 13.959	02.III.2019	Equipe Zampaulo & Reis	*T. bellinii* **sp. nov.**
15437	Brasil	MG	Prudente de Morais	Gruta ES13 - Zona Afótica	-19 26 45.766	-44 06 13.959	02.III.2019	Equipe Zampaulo & Reis	*T. bellinii* **sp. nov.**
15450	Brasil	MG	Prudente de Morais	Gruta ES03 - Zona penumbra	-19 26 45.557	-44 06 11.216	01.III.2019	Equipe Zampaulo & Reis	*T. bellinii* **sp. nov.**
16145	Brasil	MG	Prudente de Morais	Gruta 06L	-19 26 32.72	-44 06 24.24	17.VIII.2019	Equipe Zampaulo & Reis	*T. bellinii* **sp. nov.**
16146	Brasil	MG	Prudente de Morais	Gruta 06L	-19 26 32.72	-44 06 24.24	17.VIII.2019	Equipe Zampaulo & Reis	*T. bellinii* **sp. nov.**
16147	Brasil	MG	Prudente de Morais	Gruta 06L	-19 26 32.72	-44 06 24.24	17.VIII.2019	Equipe Zampaulo & Reis	*T. bellinii* **sp. nov.**
16148	Brasil	MG	Prudente de Morais	Gruta 06L	-19 26 32.72	-44 06 24.24	17.VIII.2019	Equipe Zampaulo & Reis	*T. bellinii* **sp. nov.**
16149	Brasil	MG	Prudente de Morais	Gruta 06L	-19 26 32.72	-44 06 24.24	17.VIII.2019	Equipe Zampaulo & Reis	*T. bellinii* **sp. nov.**
16150	Brasil	MG	Prudente de Morais	Gruta 06L	-19 26 32.72	-44 06 24.24	17.VIII.2019	Equipe Zampaulo & Reis	*T. bellinii* **sp. nov.**
16151	Brasil	MG	Prudente de Morais	Gruta 06L	-19 26 32.72	-44 06 24.24	17.VIII.2019	Equipe Zampaulo & Reis	*T. bellinii* **sp. nov.**
16152	Brasil	MG	Prudente de Morais	Gruta 06L	-19 26 32.72	-44 06 24.24	17.VIII.2019	Equipe Zampaulo & Reis	*T. bellinii* **sp. nov.**
16162	Brasil	MG	Prudente de Morais	Gruta 06L	-19 26 32.72	-44 06 24.24	17.VIII.2019	Equipe Zampaulo & Reis	*T. bellinii* **sp. nov.**
16169	Brasil	MG	Prudente de Morais	ES-13	-19 26 46.01	-44 06 13.95	16.VIII.2019	Equipe Zampaulo & Reis	*T. bellinii* **sp. nov.**
16195	Brasil	MG	Prudente de Morais	ES-17	-19 26 45.34	-44 06 10.18	10.IX.2019	Equipe Zampaulo & Reis	*T. bellinii* **sp. nov.**
16196	Brasil	MG	Prudente de Morais	ES-17	-19 26 45.34	-44 06 10.18	10.IX.2019	Equipe Zampaulo & Reis	*T. bellinii* **sp. nov.**
16205	Brasil	MG	Prudente de Morais	ES-17	-19 26 45.34	-44 06 10.18	10.IX.2019	Equipe Zampaulo & Reis	*T. bellinii* **sp. nov.**
16236	Brasil	MG	Prudente de Morais	Gruta 01L	-19 26 32.72	-44 06 24.24	17.VIII.2019	Equipe Zampaulo & Reis	*T. bellinii* **sp. nov.**
16352	Brasil	MG	Prudente de Morais	ICMAT-0053	-19 30 35.584	-44 07 11.291	13.IX.2018	Equipe Ativo Ambiental	*T. bellinii* **sp. nov.**
16353	Brasil	MG	Prudente de Morais	ABISMO-0018	-19 30 35.557	-44 07 5.767	01.X.2018	Equipe Ativo Ambiental	*T. bellinii* **sp. nov.**
12530	Brasil	MG	Santa Maria de Itabira	MLog	-19 19 54.192	-43 18 27.324	06-10.VIII.2018	Equipe Carste	*T. bellinii* **sp. nov.**
12533	Brasil	MG	Santa Maria de Itabira	MLog	-19 24 43.321	-43 08 53.838	06-10.VIII.2018	Equipe Carste	*T. bellinii* **sp. nov.**
13377	Brasil	MG	Santa Maria de Itabira	MLog. MOES-0022A	-19 24 43.738	-43 08 53.250	03-06.XII.2018	Equipe Carste	*T. bellinii* **sp. nov.**
13378	Brasil	MG	Santa Maria de Itabira	MLog. MOES-0022A	-19 24 43.738	-43 08 53.250	03-06.XII.2018	Equipe Carste	*T. bellinii* **sp. nov.**
13380	Brasil	MG	Santa Maria de Itabira	MLog. MOES-0022A	-19 24 43.738	-43 08 53.250	03-06.XII.2018	Equipe Carste	*T. bellinii* **sp. nov.**
13381	Brasil	MG	Santa Maria de Itabira	MLog. MOES-0022A	-19 24 43.738	-43 08 53.250	03-06.XII.2018	Equipe Carste	*T. bellinii* **sp. nov.**
13383	Brasil	MG	Santa Maria de Itabira	MLog. MOES-0022A	-19 24 43.738	-43 08 53.250	03-06.XII.2018	Equipe Carste	*T. bellinii* **sp. nov.**
13384	Brasil	MG	Santa Maria de Itabira	MLog. MOES-0022A	-19 24 43.738	-43 08 53.250	03-06.XII.2018	Equipe Carste	*T. bellinii* **sp. nov.**
13386	Brasil	MG	Santa Maria de Itabira	MLog. MOES-0023	-19 24 43.620	-43 08 54.417	03-06.XII.2018	Equipe Carste	*T. bellinii* **sp. nov.**
13329	Brasil	MG	Santo Sebastião do Rio Preto	MLog. MCFC-10	-19 19 52.837	-43 18 46.010	13-29.XI.2018	Equipe Carste	*T. bellinii* **sp. nov.**
13330	Brasil	MG	Santo Sebastião do Rio Preto	MLog. MCFC-10	-19 19 52.837	-43 18 46.010	13-29.XI.2018	Equipe Carste	*T. bellinii* **sp. nov.**
16397	Brasil	MG	São Gonçalo do Rio Abaixo	MDIR-0028	-19 52 48.690	-43 26 13.599	10-14.II.2020	Equipe Spelayon	*T. bellinii* **sp. nov.**
16398	Brasil	MG	São Gonçalo do Rio Abaixo	MDIR-0028	-19 52 48.690	-43 26 13.599	10-14.II.2020	Equipe Spelayon	*T. bellinii* **sp. nov.**
16401	Brasil	MG	São Gonçalo do Rio Abaixo	MDIR-0028	-19 52 48.690	-43 26 13.599	10-14.II.2020	Equipe Spelayon	*T. bellinii* **sp. nov.**
16402	Brasil	MG	São Gonçalo do Rio Abaixo	MDIR-0028	-19 52 48.690	-43 26 13.599	10-14.II.2020	Equipe Spelayon	*T. bellinii* **sp. nov.**
16403	Brasil	MG	São Gonçalo do Rio Abaixo	MDIR-0028	-19 52 48.690	-43 26 13.599	10-14.II.2020	Equipe Spelayon	*T. bellinii* **sp. nov.**
16404	Brasil	MG	São Gonçalo do Rio Abaixo	MDIR-0028	-19 52 48.690	-43 26 13.599	10-14.II.2020	Equipe Spelayon	*T. bellinii* **sp. nov.**
16405	Brasil	MG	São Gonçalo do Rio Abaixo	MDIR-0028	-19 52 48.690	-43 26 13.599	10-14.II.2020	Equipe Spelayon	*T. bellinii* **sp. nov.**
16406	Brasil	MG	São Gonçalo do Rio Abaixo	MDIR-0028	-19 52 48.690	-43 26 13.599	10-14.II.2020	Equipe Spelayon	*T. bellinii* **sp. nov.**
16407	Brasil	MG	São Gonçalo do Rio Abaixo	MDIR-0028	-19 52 48.690	-43 26 13.599	10-14.II.2020	Equipe Spelayon	*T. bellinii* **sp. nov.**
16410	Brasil	MG	São Gonçalo do Rio Abaixo	MDIR-0028	-19 52 48.690	-43 26 13.599	10-14.II.2020	Equipe Spelayon	*T. bellinii* **sp. nov.**
16411	Brasil	MG	São Gonçalo do Rio Abaixo	MDIR-0028	-19 52 48.690	-43 26 13.599	10-14.II.2020	Equipe Spelayon	*T. bellinii* **sp. nov.**
16413	Brasil	MG	São Gonçalo do Rio Abaixo	MDIR-0028	-19 52 48.690	-43 26 13.599	10-14.II.2020	Equipe Spelayon	*T. bellinii* **sp. nov.**
16414	Brasil	MG	São Gonçalo do Rio Abaixo	MDIR-0028	-19 52 48.690	-43 26 13.599	10-14.II.2020	Equipe Spelayon	*T. bellinii* **sp. nov.**
16415	Brasil	MG	São Gonçalo do Rio Abaixo	MDIR-0028	-19 52 48.690	-43 26 13.599	10-14.II.2020	Equipe Spelayon	*T. bellinii* **sp. nov.**
16416	Brasil	MG	São Gonçalo do Rio Abaixo	MDIR-0028	-19 52 48.690	-43 26 13.599	10-14.II.2020	Equipe Spelayon	*T. bellinii* **sp. nov.**
16417	Brasil	MG	São Gonçalo do Rio Abaixo	MDIR-0028	-19 52 48.690	-43 26 13.599	10-14.II.2020	Equipe Spelayon	*T. bellinii* **sp. nov.**
16418	Brasil	MG	São Gonçalo do Rio Abaixo	MDIR-0028	-19 52 48.690	-43 26 13.599	10-14.II.2020	Equipe Spelayon	*T. bellinii* **sp. nov.**
16419	Brasil	MG	São Gonçalo do Rio Abaixo	MDIR-0028	-19 52 48.690	-43 26 13.599	10-14.II.2020	Equipe Spelayon	*T. bellinii* **sp. nov.**
16420	Brasil	MG	São Gonçalo do Rio Abaixo	MDIR-0028	-19 52 48.690	-43 26 13.599	10-14.II.2020	Equipe Spelayon	*T. bellinii* **sp. nov.**
16421	Brasil	MG	São Gonçalo do Rio Abaixo	MDIR-0028	-19 52 48.690	-43 26 13.599	10-14.II.2020	Equipe Spelayon	*T. bellinii* **sp. nov.**
16422	Brasil	MG	São Gonçalo do Rio Abaixo	MDIR-0028	-19 52 48.690	-43 26 13.599	10-14.II.2020	Equipe Spelayon	*T. bellinii* **sp. nov.**
16423	Brasil	MG	São Gonçalo do Rio Abaixo	MDIR-0028	-19 52 48.690	-43 26 13.599	10-14.II.2020	Equipe Spelayon	*T. bellinii* **sp. nov.**
16425	Brasil	MG	São Gonçalo do Rio Abaixo	MDIR-0028	-19 52 48.690	-43 26 13.599	10-14.II.2020	Equipe Spelayon	*T. bellinii* **sp. nov.**
16427	Brasil	MG	São Gonçalo do Rio Abaixo	MDIR-0028	-19 52 48.690	-43 26 13.599	10-14.II.2020	Equipe Spelayon	*T. bellinii* **sp. nov.**

16430	Brasil	MG	São Gonçalo do Rio Abaixo	MDIR-0028	-19 52 48.690	-43 26 13.599	10-14.II.2020	Equipe Spelayon	*T. bellinii* **sp. nov.**
16434	Brasil	MG	São Gonçalo do Rio Abaixo	MDIR-0028	-19 52 48.690	-43 26 13.599	10-14.II.2020	Equipe Spelayon	*T. bellinii* **sp. nov.**
16437	Brasil	MG	São Gonçalo do Rio Abaixo	MDIR-0028	-19 52 48.690	-43 26 13.599	10-14.II.2020	Equipe Spelayon	*T. bellinii* **sp. nov.**
16442	Brasil	MG	São Gonçalo do Rio Abaixo	MDIR-0028	-19 52 48.690	-43 26 13.599	10-14.II.2020	Equipe Spelayon	*T. bellinii* **sp. nov.**
16445	Brasil	MG	São Gonçalo do Rio Abaixo	MDIR-0028	-19 52 48.690	-43 26 13.599	10-14.II.2020	Equipe Spelayon	*T. bellinii* **sp. nov.**
16446	Brasil	MG	São Gonçalo do Rio Abaixo	BRU-0005	-19 52 48.690	-43 26 13.599	10-14.II.2020	Equipe Spelayon	*T. bellinii* **sp. nov.**
16447	Brasil	MG	São Gonçalo do Rio Abaixo	BRU-0005	-19 52 48.690	-43 26 13.599	10-14.II.2020	Equipe Spelayon	*T. bellinii* **sp. nov.**
16448	Brasil	MG	São Gonçalo do Rio Abaixo	BRU-0005	-19 52 48.690	-43 26 13.599	10-14.II.2020	Equipe Spelayon	*T. bellinii* **sp. nov.**
16455	Brasil	MG	São Gonçalo do Rio Abaixo	BRU-0005	-19 52 48.690	-43 26 13.599	10-14.II.2020	Equipe Spelayon	*T. bellinii* **sp. nov.**
16456	Brasil	MG	São Gonçalo do Rio Abaixo	BRU-0005	-19 52 48.690	-43 26 13.599	10-14.II.2020	Equipe Spelayon	*T. bellinii* **sp. nov.**
16457	Brasil	MG	São Gonçalo do Rio Abaixo	BRU-0005	-19 52 48.690	-43 26 13.599	10-14.II.2020	Equipe Spelayon	*T. bellinii* **sp. nov.**
16458	Brasil	MG	São Gonçalo do Rio Abaixo	BRU-0005	-19 52 48.690	-43 26 13.599	10-14.II.2020	Equipe Spelayon	*T. bellinii* **sp. nov.**
16459	Brasil	MG	São Gonçalo do Rio Abaixo	BRU-0005	-19 52 48.690	-43 26 13.599	10-14.II.2020	Equipe Spelayon	*T. bellinii* **sp. nov.**
16460	Brasil	MG	São Gonçalo do Rio Abaixo	BRU-0005	-19 52 48.690	-43 26 13.599	10-14.II.2020	Equipe Spelayon	*T. bellinii* **sp. nov.**
16472	Brasil	MG	São Gonçalo do Rio Abaixo	BRU-0008	-19 52 32.054	-43 25 1.809	10-14.II.2020	Equipe Spelayon	*T. bellinii* **sp. nov.**
16473	Brasil	MG	São Gonçalo do Rio Abaixo	BRU-0008	-19 52 32.054	-43 25 1.809	10-14.II.2020	Equipe Spelayon	*T. bellinii* **sp. nov.**
16474	Brasil	MG	São Gonçalo do Rio Abaixo	BRU-0008	-19 52 32.054	-43 25 1.809	10-14.II.2020	Equipe Spelayon	*T. bellinii* **sp. nov.**
16475	Brasil	MG	São Gonçalo do Rio Abaixo	BRU-0008	-19 52 32.054	-43 25 1.809	10-14.II.2020	Equipe Spelayon	*T. bellinii* **sp. nov.**
16476	Brasil	MG	São Gonçalo do Rio Abaixo	BRU-0008	-19 52 32.054	-43 25 1.809	10-14.II.2020	Equipe Spelayon	*T. bellinii* **sp. nov.**
8863	Brasil	MG	Vazerlândia	?	-19 41 48.589	-43 53 53.040	19.I.2015	Equipe Rabelo, L. M.	*T. bellinii* **sp. nov.**
6567	Brasil	MG	Vespasiano	?	-19 41 48.589	-43 53 53.040	06-16.I.2015	Equipe Carste	*T. bellinii* **sp. nov.**
6568	Brasil	MG	Vespasiano	?	-19 41 48.589	-43 53 53.040	15-25.VI.2015	Equipe Carste	*T. bellinii* **sp. nov.**
6569	Brasil	MG	Vespasiano	?	-19 41 48.589	-43 53 53.040	15-25.VI.2015	Equipe Carste	*T. bellinii* **sp. nov.**
9926	Brasil	MG	Rio Acima	GAND-116	-20 04 9.502	-43 40 9.421	10.II-20.III.2014	Equipe Carste	*T. lacerta* **sp. nov.**
9929	Brasil	MG	Rio Acima	GAND-116	-20 04 9.502	-43 40 9.421	10.II-20.III.2014	Equipe Carste	*T. lacerta* **sp. nov.**
9932	Brasil	MG	Rio Acima	GAND-116	-20 04 9.502	-43 40 9.421	10.II-20.III.2014	Equipe Carste	*T. lacerta* **sp. nov.**
9935	Brasil	MG	Rio Acima	GAND-115	-20 04 9.640	-43 43 1.983	15-31.III.2016	Equipe Carste	*T. lacerta* **sp. nov.**
9936	Brasil	MG	Rio Acima	GAND-115	-20 04 9.640	-43 43 1.983	15-31.III.2016	Equipe Carste	*T. lacerta* **sp. nov.**
9940	Brasil	MG	Rio Acima	GAND-115	-20 04 9.640	-43 43 1.983	10.II-20.III.2014	Equipe Carste	*T. lacerta* **sp. nov.**
9941	Brasil	MG	Rio Acima	GAND-115	-20 04 9.640	-43 43 1.983	10.II-20.III.2014	Equipe Carste	*T. lacerta* **sp. nov.**
9942	Brasil	MG	Rio Acima	GAND-111	-20 04 6.316	-43 40 5.248	15-31.III.2016	Equipe Carste	*T. lacerta* **sp. nov.**
9943	Brasil	MG	Rio Acima	GAND-109	-20 04 2.135	-43 40 7.211	15-31.III.2016	Equipe Carste	*T. lacerta* **sp. nov.**
9945	Brasil	MG	Rio Acima	GAND-109	-20 04 2.135	-43 40 7.211	15-31.III.2016	Equipe Carste	*T. lacerta* **sp. nov.**
9949	Brasil	MG	Rio Acima	GAND-38	-20 02 28.505	-43 39 8.288	15-31.III.2016	Equipe Carste	*T. lacerta* **sp. nov.**
9952	Brasil	MG	Rio Acima	GAND-122	-20 02 51.440	-43 39 51.872	15-31.III.2016	Equipe Carste	*T. lacerta* **sp. nov.**
9953	Brasil	MG	Rio Acima	GAND-122	-20 02 51.440	-43 39 51.872	15-31.III.2016	Equipe Carste	*T. lacerta* **sp. nov.**
9954	Brasil	MG	Rio Acima	GAND-114	-20 04 4.544	-43 40 11.872	10.II-20.III.2014	Equipe Carste	*T. lacerta* **sp. nov.**
9976	Brasil	MG	Rio Acima	GAND-116	-20 04 9.502	-43 40 9.421	10.II-20.III.2014	Equipe Carste	*T. lacerta* **sp. nov.**
9978	Brasil	MG	Rio Acima	GAND-116	-20 04 9.502	-43 40 9.421	10.II-20.III.2014	Equipe Carste	*T. lacerta* **sp. nov.**
9979	Brasil	MG	Rio Acima	GAND-116	-20 04 9.502	-43 40 9.421	10.II-20.III.2014	Equipe Carste	*T. lacerta* **sp. nov.**
9980	Brasil	MG	Rio Acima	GAND-114	-20 04 4.544	-43 40 11.872	10.II-20.III.2014	Equipe Carste	*T. lacerta* **sp. nov.**
9981	Brasil	MG	Rio Acima	GAND-114	-20 04 4.544	-43 40 11.872	10.II-20.III.2014	Equipe Carste	*T. lacerta* **sp. nov.**
9982	Brasil	MG	Rio Acima	GAND-114	-20 04 4.544	-43 40 11.872	10.II-20.III.2014	Equipe Carste	*T. lacerta* **sp. nov.**
9983	Brasil	MG	Rio Acima	GAND-114	-20 04 4.544	-43 40 11.872	10.II-20.III.2014	Equipe Carste	*T. lacerta* **sp. nov.**
9984	Brasil	MG	Rio Acima	GAND-114	-20 04 4.544	-43 40 11.872	15-31.III.2016	Equipe Carste	*T. lacerta* **sp. nov.**
9985	Brasil	MG	Rio Acima	GAND-122	-20 02 51.440	-43 39 51.872	15-31.III.2016	Equipe Carste	*T. lacerta* **sp. nov.**
9986	Brasil	MG	Rio Acima	GAND-122	-20 02 51.440	-43 39 51.872	15-31.III.2016	Equipe Carste	*T. lacerta* **sp. nov.**
9987	Brasil	MG	Rio Acima	GAND-115	-20 04 9.640	-43 43 1.983	15-31.III.2016	Equipe Carste	*T. lacerta* **sp. nov.**
9988	Brasil	MG	Rio Acima	GAND-115	-20 04 9.640	-43 43 1.983	15-31.III.2016	Equipe Carste	*T. lacerta* **sp. nov.**
9989	Brasil	MG	Rio Acima	GAND-38	-20 02 28.505	-43 39 8.288	15-31.III.2016	Equipe Carste	*T. lacerta* **sp. nov.**
9990	Brasil	MG	Rio Acima	GAND-38	-20 02 28.505	-43 39 8.288	15-31.III.2016	Equipe Carste	*T. lacerta* **sp. nov.**
9992	Brasil	MG	Rio Acima	GAND-65	-20 04 21.233	-43 39 29.253	15-31.III.2016	Equipe Carste	*T. lacerta* **sp. nov.**
9995	Brasil	MG	Rio Acima	GAND-125	-20 02 49.389	-43 39 34.439	15-31.III.2016	Equipe Carste	*T. lacerta* **sp. nov.**
9996	Brasil	MG	Rio Acima	GAND-127	-20 03 9.350	-43 39 37.505	15-31.III.2016	Equipe Carste	*T. lacerta* **sp. nov.**
10012	Brasil	MG	Rio Acima	GAND-114	-20 04 4.544	-43 40 11.872	15-31.III.2016	Equipe Carste	*T. lacerta* **sp. nov.**
10013	Brasil	MG	Rio Acima	GAND-114	-20 04 4.544	-43 40 11.872	10.II-20.III.2014	Equipe Carste	*T. lacerta* **sp. nov.**
10014	Brasil	MG	Rio Acima	GAND-116	-20 04 9.502	-43 40 9.421	10.II-20.III.2014	Equipe Carste	*T. lacerta* **sp. nov.**

10025	Brasil	MG	Rio Acima	GAND-111	-20 04 6.316	-43 40 5.248	14.VII-18.IX.2016	Equipe Carste	*T. lacerta* **sp. nov.**
10030	Brasil	MG	Rio Acima	GAND-116	-20 04 9.502	-43 40 9.421	14.VII-18.IX.2016	Equipe Carste	*T. lacerta* **sp. nov.**
10032	Brasil	MG	Rio Acima	GAND-042	-20 02 59.459	-43 39 11.328	14.VII-18.IX.2016	Equipe Carste	*T. lacerta* **sp. nov.**
10033	Brasil	MG	Rio Acima	GAND-064	-20 04 24.481	-43 35 59.139	14.VII-18.IX.2016	Equipe Carste	*T. lacerta* **sp. nov.**
10034	Brasil	MG	Rio Acima	GAND-116	-20 04 9.502	-43 40 9.421	14.VII-18.IX.2016	Equipe Carste	*T. lacerta* **sp. nov.**
10035	Brasil	MG	Rio Acima	GAND-111	-20 04 6.316	-43 40 5.248	14.VII-18.IX.2016	Equipe Carste	*T. lacerta* **sp. nov.**
10036	Brasil	MG	Rio Acima	GAND-111	-20 04 6.316	-43 40 5.248	14.VII-18.IX.2016	Equipe Carste	*T. lacerta* **sp. nov.**
10037	Brasil	MG	Rio Acima	GAND-111	-20 04 6.316	-43 40 5.248	14.VII-18.IX.2016	Equipe Carste	*T. lacerta* **sp. nov.**
10038	Brasil	MG	Rio Acima	GAND-067	-20 02 22.321	-43 39 20.558	14.VII-18.IX.2016	Equipe Carste	*T. lacerta* **sp. nov.**
10056	Brasil	MG	Rio Acima	GAND-033	-20 05 23.163	-43 39 8.345	14.VII-18.IX.2016	Equipe Carste	*T. lacerta* **sp. nov.**
10057	Brasil	MG	Rio Acima	GAND-033	-20 05 23.163	-43 39 8.345	14.VII-18.IX.2016	Equipe Carste	*T. lacerta* **sp. nov.**
10058	Brasil	MG	Rio Acima	GAND-033	-20 05 23.163	-43 39 8.345	14.VII-18.IX.2016	Equipe Carste	*T. lacerta* **sp. nov.**
10061	Brasil	MG	Rio Acima	GAND-066	-20 04 54.945	-43 39 5.832	14.VII-18.IX.2016	Equipe Carste	*T. lacerta* **sp. nov.**
10062	Brasil	MG	Rio Acima	GAND-122	-20 02 51.440	-43 39 51.872	14.VII-18.IX.2016	Equipe Carste	*T. lacerta* **sp. nov.**
10063	Brasil	MG	Rio Acima	GAND-122	-20 02 51.440	-43 39 51.872	14.VII-18.IX.2016	Equipe Carste	*T. lacerta* **sp. nov.**
10064	Brasil	MG	Rio Acima	GAND-033	-20 05 23.163	-43 39 8.345	14.VII-18.IX.2016	Equipe Carste	*T. lacerta* **sp. nov.**
10086	Brasil	MG	Rio Acima	GAND-094	-20 03 54.624	-43 40 11.887	14.VII-18.IX.2016	Equipe Carste	*T. lacerta* **sp. nov.**
10088	Brasil	MG	Rio Acima	GAND-094	-20 03 54.624	-43 40 11.887	14.VII-18.IX.2016	Equipe Carste	*T. lacerta* **sp. nov.**
10089	Brasil	MG	Rio Acima	GAND-116	-20 04 9.502	-43 40 9.421	14.VII-18.IX.2016	Equipe Carste	*T. lacerta* **sp. nov.**
10090	Brasil	MG	Rio Acima	GAND-114	-20 04 4.544	-43 40 11.872	14.VII-18.IX.2016	Equipe Carste	*T. lacerta* **sp. nov.**
10091	Brasil	MG	Rio Acima	GAND-095	-20 03 54.816	-43 40 11.438	14.VII-18.IX.2016	Equipe Carste	*T. lacerta* **sp. nov.**
10098	Brasil	MG	Rio Acima	GAND-033	-20 05 23.163	-43 39 8.345	14.VII-18.IX.2016	Equipe Carste	*T. lacerta* **sp. nov.**
10107	Brasil	MG	Rio Acima	GAND-65	-20 04 21.233	-43 39 29.253	14.VII-18.IX.2016	Equipe Carste	*T. lacerta* **sp. nov.**
10109	Brasil	MG	Rio Acima	GAND-65	-20 04 21.233	-43 39 29.253	14.VII-18.IX.2016	Equipe Carste	*T. lacerta* **sp. nov.**
10123	Brasil	MG	Rio Acima	GAND-094	-20 03 54.624	-43 40 11.887	14.VII-18.IX.2016	Equipe Carste	*T. lacerta* **sp. nov.**
10124	Brasil	MG	Rio Acima	GAND-094	-20 03 54.624	-43 40 11.887	14.VII-18.IX.2016	Equipe Carste	*T. lacerta* **sp. nov.**
10125	Brasil	MG	Rio Acima	GAND-094	-20 03 54.624	-43 40 11.887	14.VII-18.IX.2016	Equipe Carste	*T. lacerta* **sp. nov.**
10126	Brasil	MG	Rio Acima	GAND-094	-20 03 54.624	-43 40 11.887	14.VII-18.IX.2016	Equipe Carste	*T. lacerta* **sp. nov.**
10127	Brasil	MG	Rio Acima	GAND-094	-20 03 54.624	-43 40 11.887	14.VII-18.IX.2016	Equipe Carste	*T. lacerta* **sp. nov.**
10128	Brasil	MG	Rio Acima	GAND-094	-20 03 54.624	-43 40 11.887	14.VII-18.IX.2016	Equipe Carste	*T. lacerta* **sp. nov.**
10129	Brasil	MG	Rio Acima	GAND-094	-20 03 54.624	-43 40 11.887	14.VII-18.IX.2016	Equipe Carste	*T. lacerta* **sp. nov.**
10130	Brasil	MG	Rio Acima	GAND-094	-20 03 54.624	-43 40 11.887	14.VII-18.IX.2016	Equipe Carste	*T. lacerta* **sp. nov.**
10131	Brasil	MG	Rio Acima	GAND-110	-20 03 59.349	-43 40 8.680	14.VII-18.IX.2016	Equipe Carste	*T. lacerta* **sp. nov.**
10133	Brasil	MG	Rio Acima	GAND-110	-20 03 59.349	-43 40 8.680	14.VII-18.IX.2016	Equipe Carste	*T. lacerta* **sp. nov.**
10134	Brasil	MG	Rio Acima	GAND-115	-20 04 9.640	-43 43 1.983	14.VII-18.IX.2016	Equipe Carste	*T. lacerta* **sp. nov.**
10135	Brasil	MG	Rio Acima	GAND-115	-20 04 9.640	-43 43 1.983	14.VII-18.IX.2016	Equipe Carste	*T. lacerta* **sp. nov.**
10137	Brasil	MG	Rio Acima	GAND-095	-20 03 54.816	-43 40 11.438	14.VII-18.IX.2016	Equipe Carste	*T. lacerta* **sp. nov.**
10138	Brasil	MG	Rio Acima	GAND-095	-20 03 54.816	-43 40 11.438	14.VII-18.IX.2016	Equipe Carste	*T. lacerta* **sp. nov.**
10139	Brasil	MG	Rio Acima	GAND-122	-20 02 51.440	-43 39 51.872	14.VII-18.IX.2016	Equipe Carste	*T. lacerta* **sp. nov.**
10140	Brasil	MG	Rio Acima	GAND-122	-20 02 51.440	-43 39 51.872	14.VII-18.IX.2016	Equipe Carste	*T. lacerta* **sp. nov.**
10141	Brasil	MG	Rio Acima	GAND-073	-20 02 22.354	-43 39 29.265	14.VII-18.IX.2016	Equipe Carste	*T. lacerta* **sp. nov.**
10142	Brasil	MG	Rio Acima	GAND-125	-20 02 49.389	-43 39 34.439	14.VII-18.IX.2016	Equipe Carste	*T. lacerta* **sp. nov.**
10145	Brasil	MG	Rio Acima	GAND-118	-20 04 13.289	-43 40 2.642	14.VII-18.IX.2016	Equipe Carste	*T. lacerta* **sp. nov.**
10146	Brasil	MG	Rio Acima	GAND-118	-20 04 13.289	-43 40 2.642	14.VII-18.IX.2016	Equipe Carste	*T. lacerta* **sp. nov.**
10148	Brasil	MG	Rio Acima	GAND-125	-20 02 49.389	-43 39 34.439	14.VII-18.IX.2016	Equipe Carste	*T. lacerta* **sp. nov.**
10171	Brasil	MG	Rio Acima	GAND-110	-20 03 59.349	-43 40 8.680	14.VII-18.IX.2016	Equipe Carste	*T. lacerta* **sp. nov.**
10173	Brasil	MG	Rio Acima	GAND-65	-20 04 21.233	-43 39 29.253	15-31.III.2016	Equipe Carste	*T. lacerta* **sp. nov.**
10174	Brasil	MG	Rio Acima	GAND-65	-20 04 21.233	-43 39 29.253	15-31.III.2016	Equipe Carste	*T. lacerta* **sp. nov.**
10175	Brasil	MG	Rio Acima	GAND-65	-20 04 21.233	-43 39 29.253	15-31.III.2016	Equipe Carste	*T. lacerta* **sp. nov.**
10176	Brasil	MG	Rio Acima	GAND-65	-20 04 21.233	-43 39 29.253	15-31.III.2016	Equipe Carste	*T. lacerta* **sp. nov.**
10177	Brasil	MG	Rio Acima	GAND-65	-20 04 21.233	-43 39 29.253	15-31.III.2016	Equipe Carste	*T. lacerta* **sp. nov.**
10178	Brasil	MG	Rio Acima	GAND-65	-20 04 21.233	-43 39 29.253	15-31.III.2016	Equipe Carste	*T. lacerta* **sp. nov.**
10179	Brasil	MG	Rio Acima	GAND-65	-20 04 21.233	-43 39 29.253	15-31.III.2016	Equipe Carste	*T. lacerta* **sp. nov.**
10180	Brasil	MG	Rio Acima	GAND-65	-20 04 21.233	-43 39 29.253	15-31.III.2016	Equipe Carste	*T. lacerta* **sp. nov.**
10182	Brasil	MG	Rio Acima	GAND-114	-20 04 4.544	-43 40 11.872	10.II-20.III.2014	Equipe Carste	*T. lacerta* **sp. nov.**
10183	Brasil	MG	Rio Acima	GAND-114	-20 04 4.544	-43 40 11.872	10.II-20.III.2014	Equipe Carste	*T. lacerta* **sp. nov.**
10184	Brasil	MG	Rio Acima	GAND-114	-20 04 4.544	-43 40 11.872	10.II-20.III.2014	Equipe Carste	*T. lacerta* **sp. nov.**

10185	Brasil	MG	Rio Acima	GAND-114	-20 04 4.544	-43 40 11.872	10.II-20.III.2014	Equipe Carste	*T. lacerta* **sp. nov.**
10186	Brasil	MG	Rio Acima	GAND-114	-20 04 4.544	-43 40 11.872	10.II-20.III.2014	Equipe Carste	*T. lacerta* **sp. nov.**
10187	Brasil	MG	Rio Acima	GAND-114	-20 04 4.544	-43 40 11.872	10.II-20.III.2014	Equipe Carste	*T. lacerta* **sp. nov.**
10188	Brasil	MG	Rio Acima	GAND-114	-20 04 4.544	-43 40 11.872	10.II-20.III.2014	Equipe Carste	*T. lacerta* **sp. nov.**
10190	Brasil	MG	Rio Acima	GAND-114	-20 04 4.544	-43 40 11.872	10.II-20.III.2014	Equipe Carste	*T. lacerta* **sp. nov.**
10191	Brasil	MG	Rio Acima	GAND-114	-20 04 4.544	-43 40 11.872	10.II-20.III.2014	Equipe Carste	*T. lacerta* **sp. nov.**
10192	Brasil	MG	Rio Acima	GAND-114	-20 04 4.544	-43 40 11.872	14.VII-18.IX.2016	Equipe Carste	*T. lacerta* **sp. nov.**
10193	Brasil	MG	Rio Acima	GAND-111	-20 04 6.316	-43 40 5.248	15-31.III.2016	Equipe Carste	*T. lacerta* **sp. nov.**
10195	Brasil	MG	Rio Acima	GAND-111	-20 04 6.316	-43 40 5.248	15-31.III.2016	Equipe Carste	*T. lacerta* **sp. nov.**
10196	Brasil	MG	Rio Acima	GAND-111	-20 04 6.316	-43 40 5.248	15-31.III.2016	Equipe Carste	*T. lacerta* **sp. nov.**
10197	Brasil	MG	Rio Acima	GAND-111	-20 04 6.316	-43 40 5.248	14.VII-18.IX.2016	Equipe Carste	*T. lacerta* **sp. nov.**
10201	Brasil	MG	Rio Acima	GAND-116	-20 04 9.502	-43 40 9.421	10.II-20.III.2014	Equipe Carste	*T. lacerta* **sp. nov.**
10202	Brasil	MG	Rio Acima	GAND-109	-20 04 2.135	-43 40 7.211	15-31.III.2016	Equipe Carste	*T. lacerta* **sp. nov.**
10203	Brasil	MG	Rio Acima	GAND-109	-20 04 2.135	-43 40 7.211	14.VII-18.IX.2016	Equipe Carste	*T. lacerta* **sp. nov.**
10205	Brasil	MG	Rio Acima	GAND-122	-20 02 51.440	-43 39 51.872	14.VII-18.IX.2016	Equipe Carste	*T. lacerta* **sp. nov.**
10206	Brasil	MG	Rio Acima	GAND-122	-20 02 51.440	-43 39 51.872	14.VII-18.IX.2016	Equipe Carste	*T. lacerta* **sp. nov.**
10231	Brasil	MG	Rio Acima	GAND-110	-20 03 59.349	-43 40 8.680	14.VII-18.IX.2016	Equipe Carste	*T. lacerta* **sp. nov.**
10232	Brasil	MG	Rio Acima	GAND-110	-20 03 59.349	-43 40 8.680	14.VII-18.IX.2016	Equipe Carste	*T. lacerta* **sp. nov.**
10233	Brasil	MG	Rio Acima	GAND-110	-20 03 59.349	-43 40 8.680	14.VII-18.IX.2016	Equipe Carste	*T. lacerta* **sp. nov.**
10234	Brasil	MG	Rio Acima	GAND-110	-20 03 59.349	-43 40 8.680	14.VII-18.IX.2016	Equipe Carste	*T. lacerta* **sp. nov.**
10235	Brasil	MG	Rio Acima	GAND-110	-20 03 59.349	-43 40 8.680	14.VII-18.IX.2016	Equipe Carste	*T. lacerta* **sp. nov.**
10236	Brasil	MG	Rio Acima	GAND-110	-20 03 59.349	-43 40 8.680	14.VII-18.IX.2016	Equipe Carste	*T. lacerta* **sp. nov.**
10238	Brasil	MG	Rio Acima	GAND-109	-20 04 2.135	-43 40 7.211	14.VII-18.IX.2016	Equipe Carste	*T. lacerta* **sp. nov.**
10240	Brasil	MG	Rio Acima	GAND-040	-20 02 33.800	-43 39 11.684	14.VII-18.IX.2016	Equipe Carste	*T. lacerta* **sp. nov.**
10241	Brasil	MG	Rio Acima	GAND-040	-20 02 33.800	-43 39 11.684	14.VII-18.IX.2016	Equipe Carste	*T. lacerta* **sp. nov.**
10242	Brasil	MG	Rio Acima	GAND-041	-20 02 35.013	-43 39 12.982	14.VII-18.IX.2016	Equipe Carste	*T. lacerta* **sp. nov.**
10261	Brasil	MG	Rio Acima	GAND-116	-20 04 9.502	-43 40 9.421	14.VII-18.IX.2016	Equipe Carste	*T. lacerta* **sp. nov.**
10262	Brasil	MG	Rio Acima	GAND-116	-20 04 9.502	-43 40 9.421	14.VII-18.IX.2016	Equipe Carste	*T. lacerta* **sp. nov.**
10266	Brasil	MG	Rio Acima	GAND-115	-20 04 9.640	-43 43 1.983	14.VII-18.IX.2016	Equipe Carste	*T. lacerta* **sp. nov.**
10268	Brasil	MG	Rio Acima	GAND-065	-20 04 21.233	-43 39 29.253	14.VII-18.IX.2016	Equipe Carste	*T. lacerta* **sp. nov.**
10270	Brasil	MG	Rio Acima	GAND-095	-20 03 54.816	-43 40 11.438	14.VII-18.IX.2016	Equipe Carste	*T. lacerta* **sp. nov.**
10271	Brasil	MG	Rio Acima	GAND-095	-20 03 54.816	-43 40 11.438	14.VII-18.IX.2016	Equipe Carste	*T. lacerta* **sp. nov.**
10273	Brasil	MG	Rio Acima	GAND-125	-20 02 49.389	-43 39 34.439	14.VII-18.IX.2016	Equipe Carste	*T. lacerta* **sp. nov.**
10275	Brasil	MG	Rio Acima	GAND-114	-20 04 4.544	-43 40 11.872	10.II-20.III.2014	Equipe Carste	*T. lacerta* **sp. nov.**
10276	Brasil	MG	Rio Acima	GAND-119	-20 04 26.249	-43 40 34.960	10.II-20.III.2014	Equipe Carste	*T. lacerta* **sp. nov.**
10277	Brasil	MG	Rio Acima	GAND-114	-20 04 4.544	-43 40 11.872	10.II-20.III.2014	Equipe Carste	*T. lacerta* **sp. nov.**
10279	Brasil	MG	Rio Acima	GAND-119	-20 04 26.249	-43 40 34.960	10.II-20.III.2014	Equipe Carste	*T. lacerta* **sp. nov.**
10281	Brasil	MG	Rio Acima	GAND-119	-20 04 26.249	-43 40 34.960	10.II-20.III.2014	Equipe Carste	*T. lacerta* **sp. nov.**
10282	Brasil	MG	Rio Acima	GAND-119	-20 04 26.249	-43 40 34.960	10.II-20.III.2014	Equipe Carste	*T. lacerta* **sp. nov.**
10283	Brasil	MG	Rio Acima	GAND-119	-20 04 26.249	-43 40 34.960	10.II-20.III.2014	Equipe Carste	*T. lacerta* **sp. nov.**
10284	Brasil	MG	Rio Acima	GAND-125	-20 02 49.389	-43 39 34.439	15-31.III.2016	Equipe Carste	*T. lacerta* **sp. nov.**
10285	Brasil	MG	Rio Acima	GAND-125	-20 02 49.389	-43 39 34.439	15-31.III.2016	Equipe Carste	*T. lacerta* **sp. nov.**
10286	Brasil	MG	Rio Acima	GAND-125	-20 02 49.389	-43 39 34.439	15-31.III.2016	Equipe Carste	*T. lacerta* **sp. nov.**
10309*	Brasil	MG	Rio Acima	GAND-115	-20 04 9.640	-43 43 1.983	10.II-20.III.2014	Equipe Carste	*T. lacerta* **sp. nov.**
10311**	Brasil	MG	Rio Acima	GAND-115	-20 04 9.640	-43 43 1.983	10.II-20.III.2014	Equipe Carste	*T. lacerta* **sp. nov.**
10312**	Brasil	MG	Rio Acima	GAND-115	-20 04 9.640	-43 43 1.983	10.II-20.III.2014	Equipe Carste	*T. lacerta* **sp. nov.**
10313**	Brasil	MG	Rio Acima	GAND-115	-20 04 9.640	-43 43 1.983	10.II-20.III.2014	Equipe Carste	*T. lacerta* **sp. nov.**
10314**	Brasil	MG	Rio Acima	GAND-115	-20 04 9.640	-43 43 1.983	10.II-20.III.2014	Equipe Carste	*T. lacerta* **sp. nov.**
10315	Brasil	MG	Rio Acima	GAND-038	-20 02 28.505	-43 39 8.288	15-31.III.2016	Equipe Carste	*T. lacerta* **sp. nov.**
10316	Brasil	MG	Rio Acima	GAND-038	-20 02 28.505	-43 39 8.288	15-31.III.2016	Equipe Carste	*T. lacerta* **sp. nov.**
10317	Brasil	MG	Rio Acima	GAND-114	-20 04 4.544	-43 40 11.872	10.II-20.III.2014	Equipe Carste	*T. lacerta* **sp. nov.**
10318	Brasil	MG	Rio Acima	GAND-122	-20 02 51.440	-43 39 51.872	15-31.III.2016	Equipe Carste	*T. lacerta* **sp. nov.**
10319	Brasil	MG	Rio Acima	GAND-038	-20 02 28.505	-43 39 8.288	15-31.III.2016	Equipe Carste	*T. lacerta* **sp. nov.**
10320	Brasil	MG	Rio Acima	GAND-115	-20 04 9.640	-43 43 1.983	15-31.III.2016	Equipe Carste	*T. lacerta* **sp. nov.**
10335	Brasil	MG	Rio Acima	GAND-095	-20 03 54.816	-43 40 11.438	14.VII-18.IX.2016	Equipe Carste	*T. lacerta* **sp. nov.**
10338	Brasil	MG	Rio Acima	GAND-114	-20 04 4.544	-43 40 11.872	10.II-20.III.2014	Equipe Carste	*T. lacerta* **sp. nov.**
10339	Brasil	MG	Rio Acima	GAND-114	-20 04 4.544	-43 40 11.872	10.II-20.III.2014	Equipe Carste	*T. lacerta* **sp. nov.**

10340	Brasil	MG	Rio Acima	GAND-065	-20 04 21.233	-43 39 29.253	15-31.III.2016	Equipe Carste	*T. lacerta* **sp. nov.**
10341	Brasil	MG	Rio Acima	GAND-065	-20 04 21.233	-43 39 29.253	15-31.III.2016	Equipe Carste	*T. lacerta* **sp. nov.**
10342	Brasil	MG	Rio Acima	GAND-073	-20 02 22.354	-43 39 29.265	10.II-20.III.2014	Equipe Carste	*T. lacerta* **sp. nov.**
10343	Brasil	MG	Rio Acima	GAND-073	-20 02 22.354	-43 39 29.265	10.II-20.III.2014	Equipe Carste	*T. lacerta* **sp. nov.**
10344	Brasil	MG	Rio Acima	GAND-073	-20 02 22.354	-43 39 29.265	10.II-20.III.2014	Equipe Carste	*T. lacerta* **sp. nov.**
10345	Brasil	MG	Rio Acima	GAND-067	-20 02 22.321	-43 39 20.558	10.II-20.III.2014	Equipe Carste	*T. lacerta* **sp. nov.**
10346	Brasil	MG	Rio Acima	GAND-042	-20 02 59.459	-43 39 11.328	10.II-20.III.2014	Equipe Carste	*T. lacerta* **sp. nov.**
10348	Brasil	MG	Rio Acima	GAND-123	-20 04 57.871	-43 39 14.207	10.II-20.III.2014	Equipe Carste	*T. lacerta* **sp. nov.**
10350	Brasil	MG	Rio Acima	GAND-021	-20 04 16.382	-43 39 58.726	10.II-20.III.2014	Equipe Carste	*T. lacerta* **sp. nov.**
10351	Brasil	MG	Rio Acima	GAND-094	-20 03 54.624	-43 40 11.887	10.II-20.III.2014	Equipe Carste	*T. lacerta* **sp. nov.**
10352	Brasil	MG	Rio Acima	GAND-094	-20 03 54.624	-43 40 11.887	10.II-20.III.2014	Equipe Carste	*T. lacerta* **sp. nov.**
10353	Brasil	MG	Rio Acima	GAND-094	-20 03 54.624	-43 40 11.887	10.II-20.III.2014	Equipe Carste	*T. lacerta* **sp. nov.**
10354	Brasil	MG	Rio Acima	GAND-094	-20 03 54.624	-43 40 11.887	10.II-20.III.2014	Equipe Carste	*T. lacerta* **sp. nov.**
10355	Brasil	MG	Rio Acima	GAND-066	-20 04 54.945	-43 39 5.832	10.II-20.III.2014	Equipe Carste	*T. lacerta* **sp. nov.**
10356	Brasil	MG	Rio Acima	GAND-066	-20 04 54.945	-43 39 5.832	10.II-20.III.2014	Equipe Carste	*T. lacerta* **sp. nov.**
10357	Brasil	MG	Rio Acima	GAND-066	-20 04 54.945	-43 39 5.832	10.II-20.III.2014	Equipe Carste	*T. lacerta* **sp. nov.**
10359	Brasil	MG	Rio Acima	GAND-040	-20 02 33.800	-43 39 11.684	10.II-20.III.2014	Equipe Carste	*T. lacerta* **sp. nov.**
10360	Brasil	MG	Rio Acima	GAND-040	-20 02 33.800	-43 39 11.684	10.II-20.III.2014	Equipe Carste	*T. lacerta* **sp. nov.**
10362	Brasil	MG	Rio Acima	GAND-040	-20 02 33.800	-43 39 11.684	10.II-20.III.2014	Equipe Carste	*T. lacerta* **sp. nov.**
10366	Brasil	MG	Rio Acima	GAND-033	-20 05 23.163	-43 39 8.345	10.II-20.III.2014	Equipe Carste	*T. lacerta* **sp. nov.**
10371	Brasil	MG	Rio Acima	GAND-073	-20 02 22.354	-43 39 29.265	10.II-20.III.2014	Equipe Carste	*T. lacerta* **sp. nov.**
10374	Brasil	MG	Rio Acima	GAND-073	-20 02 22.354	-43 39 29.265	10.II-20.III.2014	Equipe Carste	*T. lacerta* **sp. nov.**
10385	Brasil	MG	Rio Acima	GAND-024	-20 05 6.829	-43 39 50.278	10.II-20.III.2014	Equipe Carste	*T. lacerta* **sp. nov.**
10386	Brasil	MG	Rio Acima	GAND-024	-20 05 6.829	-43 39 50.278	10.II-20.III.2014	Equipe Carste	*T. lacerta* **sp. nov.**
10387	Brasil	MG	Rio Acima	GAND-023	-20 05 12.701	-43 43 50.701	10.II-20.III.2014	Equipe Carste	*T. lacerta* **sp. nov.**
10516	Brasil	MG	Rio Acima	SG 015	-20 05 39.960	-43 41 2.194	14.VII-18.IX.2016	Equipe Carste	*T. lacerta* **sp. nov.**
10518	Brasil	MG	Rio Acima	SG 044	-20 05 45.774	-43 40 26.373	10.II-20.III.2014	Equipe Carste	*T. lacerta* **sp. nov.**
10538	Brasil	MG	Rio Acima	SG 037	-20 06 15.633	-43 40 0.747	10.II-20.III.2014	Equipe Carste	*T. lacerta* **sp. nov.**
10665	Brasil	MG	Rio Acima	GAND-030A	-20 02 52.111	-43 39 50.249	15-31.III.2016	Equipe Carste	*T. lacerta* **sp. nov.**
10666	Brasil	MG	Rio Acima	GAND-030A	-20 02 52.111	-43 39 50.249	15-31.III.2016	Equipe Carste	*T. lacerta* **sp. nov.**
10778	Brasil	MG	Rio Acima	GAND-125	-20 02 49.389	-43 39 34.439	15-31.III.2016	Equipe Carste	*T. lacerta* **sp. nov.**
10780	Brasil	MG	Rio Acima	GAND-122	-20 02 51.440	-43 39 51.872	15-31.III.2016	Equipe Carste	*T. lacerta* **sp. nov.**
10791	Brasil	MG	Rio Acima	GAND-116	-20 04 9.502	-43 40 9.421	15-31.III.2016	Equipe Carste	*T. lacerta* **sp. nov.**
4550*	Brasil	MG	Rio Acima	GRUTA 2D7	-20 07 42.1	-42 54 26.2	02-10.VI.2021	R. Andrade	*T. chapelensis* **sp. nov.**
4551**	Brasil	MG	Rio Acima	GRUTA 7D7	-20 07 42.091	-43 54 26.760	29.III-1.IV.2011	R. Andrade	*T. chapelensis* **sp. nov.**
4552**	Brasil	MG	Rio Acima	GRUTA QD7	-20 07 42.091	-43 54 26.760	29.III-1.IV.2011	R. Andrade	*T. chapelensis* **sp. nov.**
4553**	Brasil	MG	Rio Acima	GRUTA 9D7	-20 07 42.091	-43 54 26.760	29.III-1.IV.2011	R. Andrade	*T. chapelensis* **sp. nov.**
4603**	Brasil	MG	Rio Acima	GRUTA QD7	-20 09 46.165	-43 49 36.211	29.III-1.IV.2011	R. Andrade	*T. chapelensis* **sp. nov.**
11987	Brasil	MG	Matozinhos	Belocal BM 034	-19 31 52.897	-44 05 29.997	07-20.XII.2016	Equipe Spelayon	*T. chapelensis* **sp. nov.**
16251**	Brasil	MG	Mariana	LOC-0090	-20 20 20.768	-43 23 44.320	11-14.XI.2019	Equipe Carste	*T. crystallensis* **sp. nov.**
16252*	Brasil	MG	Mariana	LOC-0090	-20 20 20.768	-43 23 44.320	11-14.XI.2019	Equipe Carste	*T. crystallensis* **sp. nov.**
16254**	Brasil	MG	Mariana	LOC-0090	-20 20 20.768	-43 23 44.320	11-14.XI.2019	Equipe Carste	*T. crystallensis* **sp. nov.**
14601	Brasil	MG	Catas Altas	Cav. BR – 0025	-20 07 26.689	-43 24 37.501	02.VII.2019	Equipe Simões & Prous	*T. sotoadamesi* **sp. nov.**
6190	Brasil	MG	Conceição do Mato Dentro	ESF-63	-18 59 51.866	-43 23 57.541	15-27.IX.2014	Equipe Carste	*T. sotoadamesi* **sp. nov.**
9225	Brasil	MG	Conceição do Mato Dentro	?	-19 00 23.860	-43 23 41.266	14.X.2015	Equipe Carste	*T. sotoadamesi* **sp. nov.**
10766	Brasil	MG	Conceição do Mato Dentro	?	-19 00 18.723	-43 23 30.236	13-14.VI.2016	Equipe Carste	*T. sotoadamesi* **sp. nov.**
11200	Brasil	MG	Conceição do Mato Dentro	SPT 0150	-19 05 29.679	-43 21 52.232	24-27.IV.2017	Equipe Carste	*T. sotoadamesi* **sp. nov.**
11323	Brasil	MG	Conceição do Mato Dentro	CSF-75	-18 59 52.032	-43 23 46.734	17.III.2015	Equipe Carste	*T. sotoadamesi* **sp. nov.**
11536	Brasil	MG	Mariana	?	-20 10 53.229	-43 31 7.912	16.I-11.II.2011	Equipe Bessi	*T. sotoadamesi* **sp. nov.**
13145	Brasil	MG	Mariana	ALEA-0003 - Alegria - Capanema	-20 09 6.817	-43 29 13.614	12.06.2017	Equipe Bioespeloeo	*T. sotoadamesi* **sp. nov.**
13146**	Brasil	MG	Mariana	ALEA-0003 - Alegria - Capanema	-20 09 6.817	-43 29 13.614	12.06.2017	Equipe Bioespeloeo	*T. sotoadamesi* **sp. nov.**
13147	Brasil	MG	Mariana	ALEA-0003 - Alegria - Capanema	-20 09 6.817	-43 29 13.614	12.06.2017	Equipe Bioespeloeo	*T. sotoadamesi* **sp. nov.**
13148	Brasil	MG	Mariana	ALEA-0003 - Alegria - Capanema	-20 09 6.817	-43 29 13.614	12.06.2017	Equipe Bioespeloeo	*T. sotoadamesi* **sp. nov.**
13149	Brasil	MG	Mariana	ALEA-0003 - Alegria - Capanema	-20 09 6.817	-43 29 13.614	12.06.2017	Equipe Bioespeloeo	*T. sotoadamesi* **sp. nov.**
13151	Brasil	MG	Mariana	ALEA-0003 - Alegria - Capanema	-20 09 6.817	-43 29 13.614	12.06.2017	Equipe Bioespeloeo	*T. sotoadamesi* **sp. nov.**
13152	Brasil	MG	Mariana	ALEA-0003 - Alegria - Capanema	-20 09 6.817	-43 29 13.614	12.06.2017	Equipe Bioespeloeo	*T. sotoadamesi* **sp. nov.**
13153**	Brasil	MG	Mariana	ALEA-0003 - Alegria - Capanema	-20 09 6.817	-43 29 13.614	12.06.2017	Equipe Bioespeloeo	*T. sotoadamesi* **sp. nov.**

13154	Brasil	MG	Mariana	ALEA-0003 - Alegria - Capanema	-20 09 6.817	-43 29 13.614	12.06.2017	Equipe Bioespeloeo	*T. sotoadamesi* **sp. nov.**
13155	Brasil	MG	Mariana	ALEA-0003 - Alegria - Capanema	-20 09 6.817	-43 29 13.614	12.06.2017	Equipe Bioespeloeo	*T. sotoadamesi* **sp. nov.**
13156	Brasil	MG	Mariana	ALEA-0003 - Alegria - Capanema	-20 09 6.817	-43 29 13.614	07.02.2018	Equipe Bioespeloeo	*T. sotoadamesi* **sp. nov.**
13157	Brasil	MG	Mariana	ALEA-0003 - Alegria - Capanema	-20 09 6.817	-43 29 13.614	07.02.2018	Equipe Bioespeloeo	*T. sotoadamesi* **sp. nov.**
13159	Brasil	MG	Mariana	ALEA-0003 - Alegria - Capanema	-20 09 6.817	-43 29 13.614	07.02.2018	Equipe Bioespeloeo	*T. sotoadamesi* **sp. nov.**
13160	Brasil	MG	Mariana	ALEA-0003 - Alegria - Capanema	-20 09 6.817	-43 29 13.614	07.02.2018	Equipe Bioespeloeo	*T. sotoadamesi* **sp. nov.**
13161	Brasil	MG	Mariana	ALEA-0003 - Alegria - Capanema	-20 09 6.817	-43 29 13.614	07.02.2018	Equipe Bioespeloeo	*T. sotoadamesi* **sp. nov.**
13162*	Brasil	MG	Mariana	ALEA-0003 - Alegria - Capanema	-20 09 6.817	-43 29 13.614	07.02.2018	Equipe Bioespeloeo	*T. sotoadamesi* **sp. nov.**
13163	Brasil	MG	Mariana	ALEA-0003 - Alegria - Capanema	-20 09 6.817	-43 29 13.614	07.02.2018	Equipe Bioespeloeo	*T. sotoadamesi* **sp. nov.**
13164	Brasil	MG	Mariana	ALEA-0003 - Alegria - Capanema	-20 09 6.817	-43 29 13.614	07.02.2018	Equipe Bioespeloeo	*T. sotoadamesi* **sp. nov.**
13165	Brasil	MG	Mariana	ALEA-0003 - Alegria - Capanema	-20 09 6.817	-43 29 13.614	07.02.2018	Equipe Bioespeloeo	*T. sotoadamesi* **sp. nov.**
13166	Brasil	MG	Mariana	ALEA-0003 - Alegria - Capanema	-20 09 6.817	-43 29 13.614	07.02.2018	Equipe Bioespeloeo	*T. sotoadamesi* **sp. nov.**
13168	Brasil	MG	Mariana	ALEA-0003 - Alegria - Capanema	-20 09 6.817	-43 29 13.614	07.02.2018	Equipe Bioespeloeo	*T. sotoadamesi* **sp. nov.**
13169	Brasil	MG	Mariana	ALEA-0001 - Alegria - Capanema	-20 09 0.560	-43 29 4.583	09.06.2017	Equipe Bioespeloeo	*T. sotoadamesi* **sp. nov.**
13170	Brasil	MG	Mariana	ALEA-0001 - Alegria - Capanema	-20 09 0.560	-43 29 4.583	09.06.2017	Equipe Bioespeloeo	*T. sotoadamesi* **sp. nov.**
13171	Brasil	MG	Mariana	ALEA-0001 - Alegria - Capanema	-20 09 0.560	-43 29 4.583	09.06.2017	Equipe Bioespeloeo	*T. sotoadamesi* **sp. nov.**
13172	Brasil	MG	Mariana	ALEA-0001 - Alegria - Capanema	-20 09 0.560	-43 29 4.583	09.06.2017	Equipe Bioespeloeo	*T. sotoadamesi* **sp. nov.**
13173**	Brasil	MG	Mariana	ALEA-0001 - Alegria - Capanema	-20 09 0.560	-43 29 4.583	09.06.2017	Equipe Bioespeloeo	*T. sotoadamesi* **sp. nov.**
13174	Brasil	MG	Mariana	ALEA-0001 - Alegria - Capanema	-20 09 0.560	-43 29 4.583	09.06.2017	Equipe Bioespeloeo	*T. sotoadamesi* **sp. nov.**
13175	Brasil	MG	Mariana	ALEA-0001 - Alegria - Capanema	-20 09 0.560	-43 29 4.583	09.06.2017	Equipe Bioespeloeo	*T. sotoadamesi* **sp. nov.**
13176	Brasil	MG	Mariana	ALEA-0001 - Alegria - Capanema	-20 09 0.560	-43 29 4.583	09.06.2017	Equipe Bioespeloeo	*T. sotoadamesi* **sp. nov.**
13177	Brasil	MG	Mariana	ALEA-0005 - Alegria - Capanema	-20 08 56.507	-43 29 9.788	27.03.2018	Equipe Bioespeloeo	*T. sotoadamesi* **sp. nov.**
13178	Brasil	MG	Mariana	ALEA-0005 - Alegria - Capanema	-20 08 56.507	-43 29 9.788	27.03.2018	Equipe Bioespeloeo	*T. sotoadamesi* **sp. nov.**
13179	Brasil	MG	Mariana	ALEA-0001 - Alegria - Capanema	-20 09 0.560	-43 29 4.583	08.02.2018	Equipe Bioespeloeo	*T. sotoadamesi* **sp. nov.**
13180	Brasil	MG	Mariana	ALEA-0001 - Alegria - Capanema	-20 09 0.560	-43 29 4.583	08.02.2018	Equipe Bioespeloeo	*T. sotoadamesi* **sp. nov.**
13181	Brasil	MG	Mariana	ALEA-0001 - Alegria - Capanema	-20 09 0.560	-43 29 4.583	08.02.2018	Equipe Bioespeloeo	*T. sotoadamesi* **sp. nov.**
13182	Brasil	MG	Mariana	ALEA-0001 - Alegria - Capanema	-20 09 0.560	-43 29 4.583	08.02.2018	Equipe Bioespeloeo	*T. sotoadamesi* **sp. nov.**
13183	Brasil	MG	Mariana	ALEA-0001 - Alegria - Capanema	-20 09 0.560	-43 29 4.583	08.02.2018	Equipe Bioespeloeo	*T. sotoadamesi* **sp. nov.**
13184	Brasil	MG	Mariana	ALEA-0001 - Alegria - Capanema	-20 09 0.560	-43 29 4.583	08.02.2018	Equipe Bioespeloeo	*T. sotoadamesi* **sp. nov.**
13185	Brasil	MG	Mariana	ALEA-0001 - Alegria - Capanema	-20 09 0.560	-43 29 4.583	08.02.2018	Equipe Bioespeloeo	*T. sotoadamesi* **sp. nov.**
13186**	Brasil	MG	Mariana	ALEA-0004 - Alegria - Capanema	-20 09 0.004	-43 29 11.786	09.06.2017	Equipe Bioespeloeo	*T. sotoadamesi* **sp. nov.**
13187	Brasil	MG	Mariana	ALEA-0004 - Alegria - Capanema	-20 09 0.004	-43 29 11.786	09.06.2017	Equipe Bioespeloeo	*T. sotoadamesi* **sp. nov.**
13188	Brasil	MG	Mariana	ALEA-0004 - Alegria - Capanema	-20 09 0.004	-43 29 11.786	09.06.2017	Equipe Bioespeloeo	*T. sotoadamesi* **sp. nov.**
13189	Brasil	MG	Mariana	ALEA-0004 - Alegria - Capanema	-20 09 0.004	-43 29 11.786	09.06.2017	Equipe Bioespeloeo	*T. sotoadamesi* **sp. nov.**
13190	Brasil	MG	Mariana	ALEA-0004 - Alegria - Capanema	-20 09 0.004	-43 29 11.786	09.06.2017	Equipe Bioespeloeo	*T. sotoadamesi* **sp. nov.**
13191	Brasil	MG	Mariana	ALEA-0004 - Alegria - Capanema	-20 09 0.004	-43 29 11.786	09.06.2017	Equipe Bioespeloeo	*T. sotoadamesi* **sp. nov.**
13192	Brasil	MG	Mariana	ALEA-0004 - Alegria - Capanema	-20 09 0.004	-43 29 11.786	09.06.2017	Equipe Bioespeloeo	*T. sotoadamesi* **sp. nov.**
13193	Brasil	MG	Mariana	ALEA-0004 - Alegria - Capanema	-20 09 0.004	-43 29 11.786	07.02.2018	Equipe Bioespeloeo	*T. sotoadamesi* **sp. nov.**
13194	Brasil	MG	Mariana	ALEA-0004 - Alegria - Capanema	-20 09 0.004	-43 29 11.786	07.02.2018	Equipe Bioespeloeo	*T. sotoadamesi* **sp. nov.**
13195	Brasil	MG	Mariana	ALEA-0004 - Alegria - Capanema	-20 09 0.004	-43 29 11.786	07.02.2018	Equipe Bioespeloeo	*T. sotoadamesi* **sp. nov.**
13196	Brasil	MG	Mariana	ALEA-0004 - Alegria - Capanema	-20 09 0.004	-43 29 11.786	07.02.2018	Equipe Bioespeloeo	*T. sotoadamesi* **sp. nov.**
13198	Brasil	MG	Mariana	ALEA-0004 - Alegria - Capanema	-20 09 0.004	-43 29 11.786	07.02.2018	Equipe Bioespeloeo	*T. sotoadamesi* **sp. nov.**
13199	Brasil	MG	Mariana	ALEA-0004 - Alegria - Capanema	-20 09 0.004	-43 29 11.786	07.02.2018	Equipe Bioespeloeo	*T. sotoadamesi* **sp. nov.**
13200	Brasil	MG	Mariana	ALEA-0004 - Alegria - Capanema	-20 09 0.004	-43 29 11.786	07.02.2018	Equipe Bioespeloeo	*T. sotoadamesi* **sp. nov.**
13201	Brasil	MG	Mariana	ALEA-0004 - Alegria - Capanema	-20 09 0.004	-43 29 11.786	07.02.2018	Equipe Bioespeloeo	*T. sotoadamesi* **sp. nov.**
13202	Brasil	MG	Mariana	ALEA-0004 - Alegria - Capanema	-20 09 0.004	-43 29 11.786	07.02.2018	Equipe Bioespeloeo	*T. sotoadamesi* **sp. nov.**
13204	Brasil	MG	Mariana	ALEA-0002 - Alegria - Capanema	-20 08 56.507	-43 29 9.788	27.III.2018	Equipe Bioespeloeo	*T. sotoadamesi* **sp. nov.**
13205	Brasil	MG	Mariana	ALEA-0002 - Alegria - Capanema	-20 08 56.507	-43 29 9.788	27.III.2018	Equipe Bioespeloeo	*T. sotoadamesi* **sp. nov.**
13206	Brasil	MG	Mariana	ALEA-0002 - Alegria - Capanema	-20 09 5.681	-43 29 13.797	27.03.2018	Equipe Bioespeloeo	*T. sotoadamesi* **sp. nov.**
13207	Brasil	MG	Mariana	ALEA-0030 - Alegria - Capanema	-20 09 0.269	-43 29 4.758	08.02.2018	Equipe Bioespeloeo	*T. sotoadamesi* **sp. nov.**
13208	Brasil	MG	Mariana	AL-01 - Alegria - Capanema	-20 09 51.021	-43 28 21.491	08.06.2017	Equipe Bioespeloeo	*T. sotoadamesi* **sp. nov.**
13209	Brasil	MG	Mariana	AL-01 - Alegria - Capanema	-20 09 51.021	-43 28 21.491	08.06.2017	Equipe Bioespeloeo	*T. sotoadamesi* **sp. nov.**
13210	Brasil	MG	Mariana	AL-02 - Alegria - Capanema	-20 09 49.888	-43 28 22.088	08.06.2017	Equipe Bioespeloeo	*T. sotoadamesi* **sp. nov.**
13211	Brasil	MG	Mariana	AL-02 - Alegria - Capanema	-20 09 49.888	-43 28 22.088	08.06.2017	Equipe Bioespeloeo	*T. sotoadamesi* **sp. nov.**
13212	Brasil	MG	Mariana	AL-02 - Alegria - Capanema	-20 09 49.888	-43 28 22.088	08.06.2017	Equipe Bioespeloeo	*T. sotoadamesi* **sp. nov.**
13213	Brasil	MG	Mariana	AL-02 - Alegria - Capanema	-20 09 49.888	-43 28 22.088	08.06.2017	Equipe Bioespeloeo	*T. sotoadamesi* **sp. nov.**
13214	Brasil	MG	Mariana	AL-02 - Alegria - Capanema	-20 09 49.888	-43 28 22.088	08.06.2017	Equipe Bioespeloeo	*T. sotoadamesi* **sp. nov.**

13215	Brasil	MG	Mariana	AL-02 - Alegria - Capanema	-20 09 49.888	-43 28 22.088	05.II.2018	Equipe Bioespeloeo	*T. sotoadamesi* **sp. nov.**
13216	Brasil	MG	Mariana	AL-02 - Alegria - Capanema	-20 09 49.888	-43 28 22.088	05.II.2018	Equipe Bioespeloeo	*T. sotoadamesi* **sp. nov.**
13217	Brasil	MG	Mariana	AL-02 - Alegria - Capanema	-20 09 49.888	-43 28 22.088	05.II.2018	Equipe Bioespeloeo	*T. sotoadamesi* **sp. nov.**
13218	Brasil	MG	Mariana	AL-02 - Alegria - Capanema	-20 09 49.888	-43 28 22.088	05.II.2018	Equipe Bioespeloeo	*T. sotoadamesi* **sp. nov.**
5857**	Brasil	MG	Conceição do Mato Dentro	SERP-79	-19 00 14.588	-43 23 56.092	15-27.IX.2014	Soares et. al.	*T. mariecurieae* **sp. nov.**
5888**	Brasil	MG	Conceição do Mato Dentro	SERP-78	-18 59 52.382	-43 23 53.262	15-27.IX.2014	Soares et. al.	*T. mariecurieae* **sp. nov.**
6189	Brasil	MG	Conceição do Mato Dentro	ESF-63	-18 59 51.866	-43 23 57.541	15-27.IX.2014	Equipe Carste	*T. mariecurieae* **sp. nov.**
6191	Brasil	MG	Conceição do Mato Dentro	CMN-41	-19 00 14.556	-43 23 56.093	15-27.IX.2014	Equipe Carste	*T. mariecurieae* **sp. nov.**
9107	Brasil	MG	Conceição do Mato Dentro	?	-19 00 23.860	-43 23 41.266	10.IX.2015	Equipe Carste	*T. mariecurieae* **sp. nov.**
9108	Brasil	MG	Conceição do Mato Dentro	?	-19 00 23.860	-43 23 41.266	10.IX.2015	Equipe Carste	*T. mariecurieae* **sp. nov.**
9109*	Brasil	MG	Conceição do Mato Dentro	?	-19 00 23.860	-43 23 41.266	10.IX.2015	Equipe Carste	*T. mariecurieae* **sp. nov.**
9110	Brasil	MG	Conceição do Mato Dentro	?	-19 00 18.458	-43 23 37.180	10.IX.2015	Equipe Carste	*T. mariecurieae* **sp. nov.**
9112	Brasil	MG	Conceição do Mato Dentro	?	-19 00 23.860	-43 23 41.266	10.IX.2015	Equipe Carste	*T. mariecurieae* **sp. nov.**
9113	Brasil	MG	Conceição do Mato Dentro	?	-19 00 18.721	-43 23 30.031	10.IX.2015	Equipe Carste	*T. mariecurieae* **sp. nov.**
9171	Brasil	MG	Conceição do Mato Dentro	?	-19 00 18.721	-43 23 30.031	11-14.I.2016	Equipe Carste	*T. mariecurieae* **sp. nov.**
9220	Brasil	MG	Conceição do Mato Dentro	?	-19 00 18.721	-43 23 30.031	14.X.2015	Equipe Carste	*T. mariecurieae* **sp. nov.**
9221	Brasil	MG	Conceição do Mato Dentro	?	-19 00 15.037	-43 23 47.779	14.X.2015	Equipe Carste	*T. mariecurieae* **sp. nov.**
9222**	Brasil	MG	Conceição do Mato Dentro	?	-19 00 18.721	-43 23 30.031	14.X.2015	Equipe Carste	*T. mariecurieae* **sp. nov.**
9223	Brasil	MG	Conceição do Mato Dentro	?	-19 00 18.721	-43 23 30.031	14.X.2015	Equipe Carste	*T. mariecurieae* **sp. nov.**
9224	Brasil	MG	Conceição do Mato Dentro	?	-19 00 23.860	-43 23 41.266	14.X.2015	Equipe Carste	*T. mariecurieae* **sp. nov.**
9229	Brasil	MG	Conceição do Mato Dentro	?	-19 00 18.721	-43 23 30.031	14.X.2015	Equipe Carste	*T. mariecurieae* **sp. nov.**
9250	Brasil	MG	Conceição do Mato Dentro	?	-19 00 15.037	-43 23 47.779	11.XI.2015	Equipe Carste	*T. mariecurieae* **sp. nov.**
9251	Brasil	MG	Conceição do Mato Dentro	?	-19 00 20.416	-43 23 30.459	11.XI.2015	Equipe Carste	*T. mariecurieae* **sp. nov.**
10638	Brasil	MG	Conceição do Mato Dentro	CSS-0113	-18 56 14.323	-43 24 41.626	31.V-12.VII.2016	Equipe Carste	*T. mariecurieae* **sp. nov.**
10760**	Brasil	MG	Conceição do Mato Dentro	?	-19 00 17.795	-43 23 31.989	18-20.IV.2016	Equipe Carste	*T. mariecurieae* **sp. nov.**
10761	Brasil	MG	Conceição do Mato Dentro	?	-19 00 17.795	-43 23 31.989	18-20.IV.2016	Equipe Carste	*T. mariecurieae* **sp. nov.**
10767	Brasil	MG	Conceição do Mato Dentro	?	-19 00 18.723	-43 23 30.236	13-14.VI.2016	Equipe Carste	*T. mariecurieae* **sp. nov.**
11333	Brasil	MG	Conceição do Mato Dentro	FSS-0026	-18 59 52.032	-43 23 46.734	31.V-12.VII.2016	Equipe Carste	*T. mariecurieae* **sp. nov.**
11337	Brasil	MG	Conceição do Mato Dentro	CMN - 28	-19 00 5.641	-43 23 44.450	25-31.III.2016	Equipe Carste	*T. mariecurieae* **sp. nov.**
11707	Brasil	MG	Conceição do Mato Dentro	?	-19 05 29.679	-43 21 52.232	06-10.V.2017	Equipe Carste	*T. mariecurieae* **sp. nov.**
12343	Brasil	MG	Conceição do Mato Dentro	?	-19 00 6.549	-43 23 40.440	18-20.VII.2017	Equipe Carste	*T. mariecurieae* **sp. nov.**
13484	Brasil	MG	Conceição do Mato Dentro	CSS-0024	-18 55 1.461	-43 25 46.157	30.IV-05.V.2018	Equipe Carste	*T. mariecurieae* **sp. nov.**
13492	Brasil	MG	Conceição do Mato Dentro	CSS-0077	-18 55 16.170	-43 25 20.078	30.IV-05.V.2018	Equipe Carste	*T. mariecurieae* **sp. nov.**
13513	Brasil	MG	Conceição do Mato Dentro	CSS-0070	-18 56 45.373	-43 24 28.753	18 -27.IV.2018	Equipe Carste	*T. mariecurieae* **sp. nov.**
13529	Brasil	MG	Conceição do Mato Dentro	FSS-0115	-18 56 25.363	-43 24 24.225	18 -27.IV.2018	Equipe Carste	*T. mariecurieae* **sp. nov.**
14047	Brasil	MG	Conceição do Mato Dentro	CSS-0068	-18 56 35.920	-43 24 45.421	22-24.IV.2019	Equipe Carste	*T. mariecurieae* **sp. nov.**
13220	Brasil	MG	Mariana	ALEA-0001 - Alegria - Capanema	-20 09 0.560	-43 29 4.583	08.II.2018	Equipe Bioespeloeo	*T. mariecurieae* **sp. nov.**
13150**	Brasil	MG	Mariana	ALEA-0003 - Alegria - Capanema	-20 09 6.817	-43 29 13.614	12.06.2017	Equipe Bioespeloeo	*T. barroca* **sp. nov.**
13158**	Brasil	MG	Mariana	ALEA-0003 - Alegria - Capanema	-20 09 6.817	-43 29 13.614	07.02.2018	Equipe Bioespeloeo	*T. barroca* **sp. nov.**
13167*	Brasil	MG	Mariana	ALEA-0003 - Alegria - Capanema	-20 09 6.817	-43 29 13.614	07.02.2018	Equipe Bioespeloeo	*T. barroca* **sp. nov.**
13197**	Brasil	MG	Mariana	ALEA-0004 - Alegria - Capanema	-20 09 0.004	-43 29 11.786	07.02.2018	Equipe Bioespeloeo	*T. barroca* **sp. nov.**
13203**	Brasil	MG	Mariana	ALEA-0002 - Alegria - Capanema	-20 08 56.507	-43 29 9.788	27.III.2018	Equipe Bioespeloeo	*T. barroca* **sp. nov.**
11698	Brasil	PA	Canaã dos Carajás	SB - 264	-6 18 42.953	-49 51 45.900	25.IV-16.V.2017	Equipe Carste	*T. gisbertae* **sp. nov.**
15462	Brasil	PA	Canaã dos Carajás	S11B-0080	-6 21 39.612	-50 23 55.707	25.I.2019	Equipe Ativo Ambiental	*T. gisbertae* **sp. nov.**
7996	Brasil	PA	Curionópolis	SL2&3	-6 00 6.506	-49 36 58.875	01-19.III.2016	Equipe Spelayon	*T. gisbertae* **sp. nov.**
8016	Brasil	PA	Curionópolis	SL2&3	-6 01 14.530	-49 37 29.436	02-15.III.2016	Equipe Spelayon	*T. gisbertae* **sp. nov.**
8017	Brasil	PA	Curionópolis	SL2&3	-5 59 59.817	-49 36 52.941	01-19.III.2016	Equipe Spelayon	*T. gisbertae* **sp. nov.**
8083	Brasil	PA	Curionópolis	SL2&3	-5 59 50.199	-49 36 47.567	01-19.III.2016	Equipe Spelayon	*T. gisbertae* **sp. nov.**
8084	Brasil	PA	Curionópolis	SL2&3	-5 59 50.200	-49 36 47.568	01-19.III.2016	Equipe Spelayon	*T. gisbertae* **sp. nov.**
8085	Brasil	PA	Curionópolis	SL2&3	-6 00 3.701	-49 36 56.769	01-19.III.2016	Equipe Spelayon	*T. gisbertae* **sp. nov.**
8086	Brasil	PA	Curionópolis	SL2&3	-5 59 56.950	-49 36 52.265	01-19.III.2016	Equipe Spelayon	*T. gisbertae* **sp. nov.**
8143	Brasil	PA	Curionópolis	SL2&3	-6 00 37.279	-49 38 19.319	02-15.III.2016	Equipe Spelayon	*T. gisbertae* **sp. nov.**
8164	Brasil	PA	Curionópolis	SL2&3	-6 00 3.701	-49 36 56.769	01-19.III.2016	Equipe Spelayon	*T. gisbertae* **sp. nov.**
8165	Brasil	PA	Curionópolis	SL2&3	-6 00 3.701	-49 36 56.769	01-19.III.2016	Equipe Spelayon	*T. gisbertae* **sp. nov.**
8166	Brasil	PA	Curionópolis	SL2&3	-6 00 3.701	-49 36 56.769	01-19.III.2016	Equipe Spelayon	*T. gisbertae* **sp. nov.**
8167	Brasil	PA	Curionópolis	SL2&3	-6 01 14.989	-49 37 30.638	02-15.III.2016	Equipe Spelayon	*T. gisbertae* **sp. nov.**
8228	Brasil	PA	Curionópolis	SL2&3	-6 00 3.701	-49 36 56.769	01-19.III.2016	Equipe Spelayon	*T. gisbertae* **sp. nov.**

8229	Brasil	PA	Curionópolis	SL2&3	-6 00 5.434	-49 36 59.919	01-19.III.2016	Equipe Spelayon	*T. gisbertae* **sp. nov.**
8231	Brasil	PA	Curionópolis	SL2&3	-5 59 38.852	-49 36 53.970	01-19.III.2016	Equipe Spelayon	*T. gisbertae* **sp. nov.**
8232	Brasil	PA	Curionópolis	SL2&3	-5 59 38.853	-49 36 53.971	01-19.III.2016	Equipe Spelayon	*T. gisbertae* **sp. nov.**
11498	Brasil	PA	Curionópolis	?	-06 00 10.733	-55 36 56.686	04.03.2014	Equipe Spelayon	*T. gisbertae* **sp. nov.**
6657**	Brasil	PA	Parauapebas	N1N8-N8 – 020	-06 10 7.803	-50 09 25.359	17.VII-04.VIII.2014	Equipe Carste	*T. gisbertae* **sp. nov.**
6669**	Brasil	PA	Parauapebas	N1N8-N8 – 017	-06 10 5.980	-50 09 25.622	17.VII-04.VIII.2014	Equipe Carste	*T. gisbertae* **sp. nov.**
6668*	Brasil	PA	Parauapebas	N1N8-N8 – 017	-06 10 5.980	-50 09 25.623	17.VII-04.VIII.2014	Equipe Carste	*T. gisbertae* **sp. nov.**
6716	Brasil	PA	Parauapebas	N1N8-N8 – 023	-06 10 6.052	-44 09 28.933	02-29.IV.2015	Equipe Carste	*T. gisbertae* **sp. nov.**
6717	Brasil	PA	Parauapebas	N1N8-N8 – 013	-06 10 4.457	-50 09 29.424	02-29.IV.2015	Equipe Carste	*T. gisbertae* **sp. nov.**
6722	Brasil	PA	Parauapebas	N1N8-N8 – 023	-06 10 6.052	-44 09 28.933	02-29.IV.2015	Equipe Carste	*T. gisbertae* **sp. nov.**
6723	Brasil	PA	Parauapebas	N1N8-N8 – 023	-06 10 6.052	-44 09 28.933	02-29.IV.2015	Equipe Carste	*T. gisbertae* **sp. nov.**
6724	Brasil	PA	Parauapebas	N1N8-N8 – 009	-06 10 6.222	-50 09 34.731	02-29.IV.2015	Equipe Carste	*T. gisbertae* **sp. nov.**
6725	Brasil	PA	Parauapebas	N1N8-N8 – 017	-06 10 5.949	-50 09 25.615	02-29.IV.2015	Equipe Carste	*T. gisbertae* **sp. nov.**
6726	Brasil	PA	Parauapebas	N1N8-N8 – 009	-06 10 6.222	-50 09 34.731	02-29.IV.2015	Equipe Carste	*T. gisbertae* **sp. nov.**
6900	Brasil	PA	Parauapebas	N1N8-N8 – 002	-06 10 3.973	-50 09 33.206	02-29.IV.2015	Equipe Carste	*T. gisbertae* **sp. nov.**
6901	Brasil	PA	Parauapebas	N1N8-N8 – 007	-06 10 4.723	-50 09 34.083	02-29.IV.2015	Equipe Carste	*T. gisbertae* **sp. nov.**
6902	Brasil	PA	Parauapebas	N1N8-N8 – 009	-06 10 6.222	-50 09 34.731	02-29.IV.2015	Equipe Carste	*T. gisbertae* **sp. nov.**
6903	Brasil	PA	Parauapebas	N1N8-N8 – 010	-06 10 8.564	-50 09 33.231	02-29.IV.2015	Equipe Carste	*T. gisbertae* **sp. nov.**
6904	Brasil	PA	Parauapebas	N1N8-N8 – 010	-06 10 8.564	-50 09 33.231	02-29.IV.2015	Equipe Carste	*T. gisbertae* **sp. nov.**
6905	Brasil	PA	Parauapebas	N1N8-N8 – 022	-06 10 4.910	-50 09 28.454	02-29.IV.2015	Equipe Carste	*T. gisbertae* **sp. nov.**
6906	Brasil	PA	Parauapebas	N1N8-N8 – 023	-06 10 6.052	-44 09 28.933	02-29.IV.2015	Equipe Carste	*T. gisbertae* **sp. nov.**
6973**	Brasil	PA	Parauapebas	N1N8-N8 – 017	-06 10 5.980	-50 09 25.623	17.VII-04.VIII.2014	Equipe Carste	*T. gisbertae* **sp. nov.**
7043	Brasil	PA	Parauapebas	N1N8-N8 – 010	-06 10 8.564	-50 09 33.231	02-29.IV.2015	Equipe Carste	*T. gisbertae* **sp. nov.**
7044	Brasil	PA	Parauapebas	N1N8-N8 – 010	-06 10 8.564	-50 09 33.231	02-29.IV.2015	Equipe Carste	*T. gisbertae* **sp. nov.**
7045	Brasil	PA	Parauapebas	N1N8-N8 – 005	-06 10 4.555	-50 09 30.569	02-29.IV.2015	Equipe Carste	*T. gisbertae* **sp. nov.**
7047	Brasil	PA	Parauapebas	N1N8-N1 – 147	-06 02 32.137	-50 16 26.016	02-29.IV.2015	Equipe Carste	*T. gisbertae* **sp. nov.**
7138**	Brasil	PA	Parauapebas	N1N8-N8 – 020	-06 10 7.803	-50 09 25.359	17.VII-04.VIII.2014	Equipe Carste	*T. gisbertae* **sp. nov.**
7172	Brasil	PA	Parauapebas	N1N8-N8 – 023	-06 10 6.052	-44 09 28.933	02-29.IV.2015	Equipe Carste	*T. gisbertae* **sp. nov.**
7173	Brasil	PA	Parauapebas	N1N8-N8 – 023	-06 10 6.052	-44 09 28.933	02-29.IV.2015	Equipe Carste	*T. gisbertae* **sp. nov.**
7175	Brasil	PA	Parauapebas	N1N8-N8 – 010	-06 10 8.564	-50 09 33.231	02-29.IV.2015	Equipe Carste	*T. gisbertae* **sp. nov.**
7176	Brasil	PA	Parauapebas	N1N8-N1 –240	-06 01 18.499	-50 16 26.080	02-29.IV.2015	Equipe Carste	*T. gisbertae* **sp. nov.**
7301	Brasil	PA	Parauapebas	N1N8-N8 – 0004	-06 10 5.787	-50 09 27.021	24.II-13.III.2015	Equipe Carste	*T. gisbertae* **sp. nov.**
7320	Brasil	PA	Parauapebas	N1N8-N1 – 0205	-06 02 42.179	-50 16 35.827	24.II-13.III.2015	Equipe Carste	*T. gisbertae* **sp. nov.**
7321	Brasil	PA	Parauapebas	N1N8-N8 – 0004	-06 10 5.787	-50 09 27.021	24.II-13.III.2015	Equipe Carste	*T. gisbertae* **sp. nov.**
7343	Brasil	PA	Parauapebas	N3-039	-06 02 23.708	-50 13 21.362	05-17.III.2013	Equipe Carste	*T. gisbertae* **sp. nov.**
11726	Brasil	PA	Parauapebas	Cav N4E-098	-06 02 1.497	-50 09 19.548	27.VIII.2017	Equipe Ativo Ambiental	*T. gisbertae* **sp. nov.**
13637	Brasil	PA	São Félix do Xingú	SFX_0073	-6 25 22.849	-51 50 36.085	22.II.2018	Equipe Ativo Ambiental	*T. gisbertae* **sp. nov.**
13638	Brasil	PA	São Félix do Xingú	SFX_0073	-6 25 22.849	-51 50 36.085	22.II.2018	Equipe Ativo Ambiental	*T. gisbertae* **sp. nov.**
13639	Brasil	PA	São Félix do Xingú	SFX_0075	-6 25 25.098	-51 50 34.721	05.III.2018	Equipe Ativo Ambiental	*T. gisbertae* **sp. nov.**
13640	Brasil	PA	São Félix do Xingú	SFX_0037	-6 23 47.743	-51 53 23.756	27.II.2018	Equipe Ativo Ambiental	*T. gisbertae* **sp. nov.**
13641	Brasil	PA	São Félix do Xingú	SFX_0037	-6 23 47.743	-51 53 23.756	27.II.2018	Equipe Ativo Ambiental	*T. gisbertae* **sp. nov.**
13645	Brasil	PA	São Félix do Xingú	SFX_0009	-6 25 59.253	-51 50 55.904	23.II.2018	Equipe Ativo Ambiental	*T. gisbertae* **sp. nov.**
13647	Brasil	PA	São Félix do Xingú	SFX_0001	-6 25 11.818	-51 50 50.747	03.II.2018	Equipe Ativo Ambiental	*T. gisbertae* **sp. nov.**
13649	Brasil	PA	São Félix do Xingú	SFX_0032	-6 23 25.287	-51 52 39.319	30.I.2018	Equipe Ativo Ambiental	*T. gisbertae* **sp. nov.**
12281	Brasil	PA	Parauapebas	N4WS-23	-06 03 46.744	-50 11 29.665	15.I.2018	Equipe R. Andrade	*T. dandarae* **sp. nov.**
12282	Brasil	PA	Parauapebas	N4WS-23	-06 03 46.744	-50 11 29.665	15.I.2018	Equipe R. Andrade	*T. dandarae* **sp. nov.**
12283	Brasil	PA	Parauapebas	N4WS-38	-06 04 6.154	-50 11 30.645	22.XI.2017	Equipe R. Andrade	*T. dandarae* **sp. nov.**
12772**	Brasil	PA	Parauapebas	N4WS-0016	-06 04 35.568	-50 11 37.140	21-30.VII.2018	Equipe Brandt Meio Ambiente	*T. dandarae* **sp. nov.**
12773**	Brasil	PA	Parauapebas	N4WS-0016	-06 04 35.568	-50 11 37.140	21-30.VII.2018	Equipe Brandt Meio Ambiente	*T. dandarae* **sp. nov.**
12774	Brasil	PA	Parauapebas	N4WS-0018/48	-06 04 34.527	-50 11 37.759	21-30.VII.2018	Equipe Brandt Meio Ambiente	*T. dandarae* **sp. nov.**
12775*	Brasil	PA	Parauapebas	N4WS-0018/48	-06 04 34.527	-50 11 37.759	21-30.VII.2018	Equipe Brandt Meio Ambiente	*T. dandarae* **sp. nov.**
12776**	Brasil	PA	Parauapebas	N4WS-0018/48	-06 04 34.527	-50 11 37.759	21-30.VII.2018	Equipe Brandt Meio Ambiente	*T. dandarae* **sp. nov.**
12777**	Brasil	PA	Parauapebas	N4WS-0018/48	-06 04 34.527	-50 11 37.759	21-30.VII.2018	Equipe Brandt Meio Ambiente	*T. dandarae* **sp. nov.**
12778**	Brasil	PA	Parauapebas	N4WS-0018/48	-06 04 34.527	-50 11 37.759	21-30.VII.2018	Equipe Brandt Meio Ambiente	*T. dandarae* **sp. nov.**
13277	Brasil	PA	Parauapebas	N4WS-0006	-06 04 36.056	-50 11 36.456	21-30.VII.2018	Equipe Brandt Meio Ambiente	*T. dandarae* **sp. nov.**
13280	Brasil	PA	Parauapebas	N4WS-0050/51	-06 04 42.956	-50 11 34.364	21-30.VII.2018	Equipe Brandt Meio Ambiente	*T. dandarae* **sp. nov.**
13281	Brasil	PA	Parauapebas	N4WS-0050/51	-06 04 42.956	-50 11 34.364	21-30.VII.2018	Equipe Brandt Meio Ambiente	*T. dandarae* **sp. nov.**

13648	Brasil	PA	São Félix do Xingú	SFX_0035	-6 23 39.379	-51 53 21.593	19.I.2018	Equipe Ativo Ambiental	*T. dandarae* **sp. nov.**
1023	Brasil	MG	Conceição do Mato Dentro	CAI-03	-18 53 0.891	-43 25 50.460	03-13.V.2011	Equipe Bessi	*T. epitychia* **sp. nov.**
1025	Brasil	MG	Conceição do Mato Dentro	CSS-01	-18 54 59.428	-43 25 40.331	03-13.V.2011	Equipe Bessi	*T. epitychia* **sp. nov.**
1026	Brasil	MG	Conceição do Mato Dentro	CSS-04	-18 55 0.697	-43 25 40.422	15.XII.10 - 14.I.11	Equipe Bessi	*T. epitychia* **sp. nov.**
1027	Brasil	MG	Conceição do Mato Dentro	CSS-15	-18 56 16.623	-43 24 29.162	15.XII.10 - 14.I.11	Equipe Bessi	*T. epitychia* **sp. nov.**
1028	Brasil	MG	Conceição do Mato Dentro	CSS-09	-18 56 9.369	-43 24 44.236	03-13.V.2011	Equipe Bessi	*T. epitychia* **sp. nov.**
1029	Brasil	MG	Conceição do Mato Dentro	CSS-05	-18 55 0.631	-43 25 40.320	15.XII.10 - 14.I.11	Equipe Bessi	*T. epitychia* **sp. nov.**
1031	Brasil	MG	Conceição do Mato Dentro	CSS-06	-18 55 1.583	-43 25 41.337	15.XII.10 - 14.I.11	Equipe Bessi	*T. epitychia* **sp. nov.**
1032	Brasil	MG	Conceição do Mato Dentro	CSS-06	-18 55 1.583	-43 25 41.337	03-13.V.2011	Equipe Bessi	*T. epitychia* **sp. nov.**
1034	Brasil	MG	Conceição do Mato Dentro	CSS-09	-18 56 9.369	-43 24 44.236	15.XII.10 - 14.I.11	Equipe Bessi	*T. epitychia* **sp. nov.**
1643	Brasil	MG	Conceição do Mato Dentro	CSS-09	-18 56 9.369	-43 24 44.236	03-13.V.2011	Equipe Bessi	*T. epitychia* **sp. nov.**
1646	Brasil	MG	Conceição do Mato Dentro	CSS-06	-18 55 1.583	-43 25 41.337	03-13.V.2011	Equipe Bessi	*T. epitychia* **sp. nov.**
1658	Brasil	MG	Conceição do Mato Dentro	CSS-05	-18 55 0.631	-43 25 40.320	03-13.V.2011	Equipe Bessi	*T. epitychia* **sp. nov.**
1661	Brasil	MG	Conceição do Mato Dentro	CSS-06	-18 55 1.583	-43 25 41.337	15.XII.10-14.I.2011	Equipe Bessi	*T. epitychia* **sp. nov.**
3436	Brasil	MG	Conceição do Mato Dentro	Gruta CAI-03	-18 53 0.891	-43 25 50.460	27-29.II-01-09.III.2012	Equipe Bessi	*T. epitychia* **sp. nov.**
3440	Brasil	MG	Conceição do Mato Dentro	Gruta CSS-02	-18 54 59.882	-43 25 40.122	27-29.II-01-09.III.2012	Equipe Bessi	*T. epitychia* **sp. nov.**
3441	Brasil	MG	Conceição do Mato Dentro	Gruta CSS-05	-18 55 0.631	-43 25 40.320	27-29.II-01-09.III.2012	Equipe Bessi	*T. epitychia* **sp. nov.**
3442	Brasil	MG	Conceição do Mato Dentro	Gruta CSS-05	-18 55 0.631	-43 25 40.320	27-29.II-01-09.III.2012	Equipe Bessi	*T. epitychia* **sp. nov.**
3444	Brasil	MG	Conceição do Mato Dentro	Gruta CSS-06	-18 55 1.583	-43 25 41.337	27-29.II-01-09.III.2012	Equipe Bessi	*T. epitychia* **sp. nov.**
3446	Brasil	MG	Conceição do Mato Dentro	Gruta CSS-06	-18 55 1.583	-43 25 41.337	27-29.II-01-09.III.2012	Equipe Bessi	*T. epitychia* **sp. nov.**
3448	Brasil	MG	Conceição do Mato Dentro	Gruta CSS-06	-18 55 1.583	-43 25 41.337	27-29.II-01-09.III.2012	Equipe Bessi	*T. epitychia* **sp. nov.**
3449	Brasil	MG	Conceição do Mato Dentro	Gruta CSS-06	-18 55 1.583	-43 25 41.337	27-29.II-01-09.III.2012	Equipe Bessi	*T. epitychia* **sp. nov.**
3452	Brasil	MG	Conceição do Mato Dentro	Gruta CSS-09	-18 56 9.369	-43 24 44.236	27-29.II-01-09.III.2012	Equipe Bessi	*T. epitychia* **sp. nov.**
3463	Brasil	MG	Conceição do Mato Dentro	Gruta CSS-04	-18 55 0.697	-43 25 40.422	25.VI-06.VII.2012	Equipe Bessi	*T. epitychia* **sp. nov.**
3468	Brasil	MG	Conceição do Mato Dentro	Gruta CSS-06	-18 55 1.583	-43 25 41.337	25.VI-06.VII.2012	Equipe Bessi	*T. epitychia* **sp. nov.**
3481	Brasil	MG	Conceição do Mato Dentro	Gruta CSS-05	-18 55 0.631	-43 25 40.320	25.VI-06.VII.2012	Equipe Bessi	*T. epitychia* **sp. nov.**
3486	Brasil	MG	Conceição do Mato Dentro	Gruta CSS-09	-18 56 9.369	-43 24 44.236	25.VI-06.VII.2012	Equipe Bessi	*T. epitychia* **sp. nov.**
3492	Brasil	MG	Conceição do Mato Dentro	Gruta CSS-05	-18 55 0.631	-43 25 40.320	12-23.XI.2012	Equipe Bessi	*T. epitychia* **sp. nov.**
3493	Brasil	MG	Conceição do Mato Dentro	Gruta CSS-05	-18 55 0.631	-43 25 40.320	12-23.XI.2012	Equipe Bessi	*T. epitychia* **sp. nov.**
3508	Brasil	MG	Conceição do Mato Dentro	Gruta CSS-09	-18 56 9.369	-43 24 44.236	12-23.XI.2012	Equipe Bessi	*T. epitychia* **sp. nov.**
4900	Brasil	MG	Conceição do Mato Dentro	?	-18 56 9.369	-43 24 44.236	12-26.VIII.2013	Equipe Soares	*T. epitychia* **sp. nov.**
4901	Brasil	MG	Conceição do Mato Dentro	?	-18 56 9.369	-43 24 44.236	12-26.VIII.2013	Equipe Soares	*T. epitychia* **sp. nov.**
4902	Brasil	MG	Conceição do Mato Dentro	?	-18 56 9.369	-43 24 44.236	12-26.VIII.2013	Equipe Soares	*T. epitychia* **sp. nov.**
4904	Brasil	MG	Conceição do Mato Dentro	?	-18 56 9.369	-43 24 44.236	12-26.VIII.2013	Equipe Soares	*T. epitychia* **sp. nov.**
4905	Brasil	MG	Conceição do Mato Dentro	?	-18 54 59.428	-43 25 40.331	12-26.VIII.2013	Equipe Soares	*T. epitychia* **sp. nov.**
4906	Brasil	MG	Conceição do Mato Dentro	?	-18 55 0.697	-43 25 40.422	12-26.VIII.2013	Equipe Soares	*T. epitychia* **sp. nov.**
4907	Brasil	MG	Conceição do Mato Dentro	?	-18 55 0.631	-43 25 40.320	12-26.VIII.2013	Equipe Soares	*T. epitychia* **sp. nov.**
4908	Brasil	MG	Conceição do Mato Dentro	?	-18 55 0.631	-43 25 40.320	12-26.VIII.2013	Equipe Soares	*T. epitychia* **sp. nov.**
4909	Brasil	MG	Conceição do Mato Dentro	?	-18 55 0.631	-43 25 40.320	12-26.VIII.2013	Equipe Soares	*T. epitychia* **sp. nov.**
4910	Brasil	MG	Conceição do Mato Dentro	?	-18 55 1.583	-43 25 41.337	12-26.VIII.2013	Equipe Soares	*T. epitychia* **sp. nov.**
4911	Brasil	MG	Conceição do Mato Dentro	?	-18 55 1.583	-43 25 41.337	12-26.VIII.2013	Equipe Soares	*T. epitychia* **sp. nov.**
4912	Brasil	MG	Conceição do Mato Dentro	?	-18 55 1.583	-43 25 41.337	12-26.VIII.2013	Equipe Soares	*T. epitychia* **sp. nov.**
4913	Brasil	MG	Conceição do Mato Dentro	?	-18 55 1.583	-43 25 41.337	12-26.VIII.2013	Equipe Soares	*T. epitychia* **sp. nov.**
4914	Brasil	MG	Conceição do Mato Dentro	?	-18 55 1.583	-43 25 41.337	12-26.VIII.2013	Equipe Soares	*T. epitychia* **sp. nov.**
4915	Brasil	MG	Conceição do Mato Dentro	?	-18 55 1.583	-43 25 41.337	12-26.VIII.2013	Equipe Soares	*T. epitychia* **sp. nov.**
8978	Brasil	MG	Conceição do Mato Dentro	?	-18 53 0.891	-43 25 50.460	20.VII-06.VIII.2015	Equipe Carste	*T. epitychia* **sp. nov.**
8980	Brasil	MG	Conceição do Mato Dentro	?	-18 53 0.891	-43 25 50.460	20.VII-06.VIII.2015	Equipe Carste	*T. epitychia* **sp. nov.**
8981	Brasil	MG	Conceição do Mato Dentro	?	-18 53 0.891	-43 25 50.460	20.VII-06.VIII.2015	Equipe Carste	*T. epitychia* **sp. nov.**
8982	Brasil	MG	Conceição do Mato Dentro	?	-18 53 0.891	-43 25 50.460	20.VII-06.VIII.2015	Equipe Carste	*T. epitychia* **sp. nov.**
8983	Brasil	MG	Conceição do Mato Dentro	?	-18 53 0.891	-43 25 50.460	20.VII-06.VIII.2015	Equipe Carste	*T. epitychia* **sp. nov.**
8984	Brasil	MG	Conceição do Mato Dentro	?	-18 55 1.583	-43 25 41.337	20.VII-06.VIII.2015	Equipe Carste	*T. epitychia* **sp. nov.**
8985	Brasil	MG	Conceição do Mato Dentro	?	-18 55 1.583	-43 25 41.337	20.VII-06.VIII.2015	Equipe Carste	*T. epitychia* **sp. nov.**
8986	Brasil	MG	Conceição do Mato Dentro	?	-18 55 1.583	-43 25 41.337	20.VII-06.VIII.2015	Equipe Carste	*T. epitychia* **sp. nov.**
8987	Brasil	MG	Conceição do Mato Dentro	?	-18 54 59.428	-43 25 40.331	20.VII-06.VIII.2015	Equipe Carste	*T. epitychia* **sp. nov.**
8988	Brasil	MG	Conceição do Mato Dentro	?	-18 55 0.864	-43 25 40.865	20.VII-06.VIII.2015	Equipe Carste	*T. epitychia* **sp. nov.**
8989	Brasil	MG	Conceição do Mato Dentro	?	-18 55 0.865	-43 25 40.866	20.VII-06.VIII.2015	Equipe Carste	*T. epitychia* **sp. nov.**
8990	Brasil	MG	Conceição do Mato Dentro	?	-18 54 59.882	-43 25 40.122	20.VII-06.VIII.2015	Equipe Carste	*T. epitychia* **sp. nov.**

8991	Brasil	MG	Conceição do Mato Dentro	?	-18 54 59.882	-43 25 40.122	20.VII-06.VIII.2015	Equipe Carste	*T. epitychia* **sp. nov.**
8992	Brasil	MG	Conceição do Mato Dentro	?	-18 56 56.142	-43 24 10.294	20.VII-06.VIII.2015	Equipe Carste	*T. epitychia* **sp. nov.**
9033	Brasil	MG	Conceição do Mato Dentro	?	-18 56 9.369	-43 24 44.236	20.VII-06.VIII.2015	Equipe Carste	*T. epitychia* **sp. nov.**
9034	Brasil	MG	Conceição do Mato Dentro	?	-18 56 9.369	-43 24 44.236	20.VII-06.VIII.2015	Equipe Carste	*T. epitychia* **sp. nov.**
9035	Brasil	MG	Conceição do Mato Dentro	?	-18 56 9.369	-43 24 44.236	20.VII-06.VIII.2015	Equipe Carste	*T. epitychia* **sp. nov.**
9036	Brasil	MG	Conceição do Mato Dentro	?	-18 55 1.583	-43 25 41.337	20.VII-06.VIII.2015	Equipe Carste	*T. epitychia* **sp. nov.**
9037	Brasil	MG	Conceição do Mato Dentro	?	-18 55 1.583	-43 25 41.337	20.VII-06.VIII.2015	Equipe Carste	*T. epitychia* **sp. nov.**
9038	Brasil	MG	Conceição do Mato Dentro	?	-18 55 1.583	-43 25 41.337	20.VII-06.VIII.2015	Equipe Carste	*T. epitychia* **sp. nov.**
9039	Brasil	MG	Conceição do Mato Dentro	?	-18 55 0.631	-43 25 40.320	20.VII-06.VIII.2015	Equipe Carste	*T. epitychia* **sp. nov.**
9040	Brasil	MG	Conceição do Mato Dentro	?	-18 55 0.631	-43 25 40.320	20.VII-06.VIII.2015	Equipe Carste	*T. epitychia* **sp. nov.**
9042	Brasil	MG	Conceição do Mato Dentro	?	-18 55 0.697	-43 25 40.422	20.VII-06.VIII.2015	Equipe Carste	*T. epitychia* **sp. nov.**
9104	Brasil	MG	Conceição do Mato Dentro	?	-18 55 0.631	-43 25 40.320	11-29.I.2016	Equipe Carste	*T. epitychia* **sp. nov.**
9306	Brasil	MG	Conceição do Mato Dentro	?	-18 53 0.891	-43 25 50.460	20.VII-06.VIII.2015	Equipe Carste	*T. epitychia* **sp. nov.**
9307	Brasil	MG	Conceição do Mato Dentro	?	-18 53 0.891	-43 25 50.460	20.VII-06.VIII.2015	Equipe Carste	*T. epitychia* **sp. nov.**
9318	Brasil	MG	Conceição do Mato Dentro	?	-18 55 0.697	-43 25 40.422	20.VII-06.VIII.2015	Equipe Carste	*T. epitychia* **sp. nov.**
9322	Brasil	MG	Conceição do Mato Dentro	?	-18 55 0.697	-43 25 40.422	20.VII-06.VIII.2015	Equipe Carste	*T. epitychia* **sp. nov.**
9323	Brasil	MG	Conceição do Mato Dentro	?	-18 55 0.697	-43 25 40.422	20.VII-06.VIII.2015	Equipe Carste	*T. epitychia* **sp. nov.**
9324	Brasil	MG	Conceição do Mato Dentro	?	-18 55 0.631	-43 25 40.320	20.VII-06.VIII.2015	Equipe Carste	*T. epitychia* **sp. nov.**
9325	Brasil	MG	Conceição do Mato Dentro	?	-18 55 0.631	-43 25 40.320	20.VII-06.VIII.2015	Equipe Carste	*T. epitychia* **sp. nov.**
9326	Brasil	MG	Conceição do Mato Dentro	?	-18 56 56.142	-43 24 10.294	20.VII-06.VIII.2015	Equipe Carste	*T. epitychia* **sp. nov.**
9327	Brasil	MG	Conceição do Mato Dentro	?	-18 56 56.142	-43 24 10.294	20.VII-06.VIII.2015	Equipe Carste	*T. epitychia* **sp. nov.**
9333	Brasil	MG	Conceição do Mato Dentro	?	-18 56 16.623	-43 24 29.162	20.VII-06.VIII.2015	Equipe Carste	*T. epitychia* **sp. nov.**
9692	Brasil	MG	Conceição do Mato Dentro	?	-18 56 14.049	-43 24 43.816	31.V-12.VI.2016	Equipe Carste	*T. epitychia* **sp. nov.**
9693	Brasil	MG	Conceição do Mato Dentro	?	-18 56 14.049	-43 24 43.816	31.V-12.VI.2016	Equipe Carste	*T. epitychia* **sp. nov.**
9695	Brasil	MG	Conceição do Mato Dentro	?	-18 56 14.049	-43 24 43.816	31.V-12.VI.2016	Equipe Carste	*T. epitychia* **sp. nov.**
9696	Brasil	MG	Conceição do Mato Dentro	?	-18 56 14.049	-43 24 43.816	31.V-12.VI.2016	Equipe Carste	*T. epitychia* **sp. nov.**
9699	Brasil	MG	Conceição do Mato Dentro	?	-18 56 14.049	-43 24 43.816	31.V-12.VI.2016	Equipe Carste	*T. epitychia* **sp. nov.**
9700	Brasil	MG	Conceição do Mato Dentro	?	-18 56 14.049	-43 24 43.816	31.V-12.VI.2016	Equipe Carste	*T. epitychia* **sp. nov.**
9701	Brasil	MG	Conceição do Mato Dentro	?	-18 56 14.049	-43 24 43.816	31.V-12.VI.2016	Equipe Carste	*T. epitychia* **sp. nov.**
9702	Brasil	MG	Conceição do Mato Dentro	?	-18 56 24.762	-43 24 22.453	31.V-12.VI.2016	Equipe Carste	*T. epitychia* **sp. nov.**
9703	Brasil	MG	Conceição do Mato Dentro	?	-18 56 24.762	-43 24 22.453	31.V-12.VI.2016	Equipe Carste	*T. epitychia* **sp. nov.**
9705	Brasil	MG	Conceição do Mato Dentro	?	-18 56 24.762	-43 24 22.453	31.V-12.VI.2016	Equipe Carste	*T. epitychia* **sp. nov.**
9706	Brasil	MG	Conceição do Mato Dentro	?	-18 56 24.762	-43 24 22.453	31.V-12.VI.2016	Equipe Carste	*T. epitychia* **sp. nov.**
9707	Brasil	MG	Conceição do Mato Dentro	?	-18 56 24.762	-43 24 22.453	31.V-12.VI.2016	Equipe Carste	*T. epitychia* **sp. nov.**
9708	Brasil	MG	Conceição do Mato Dentro	?	-18 56 24.762	-43 24 22.453	31.V-12.VI.2016	Equipe Carste	*T. epitychia* **sp. nov.**
9709	Brasil	MG	Conceição do Mato Dentro	?	-18 56 24.762	-43 24 22.453	31.V-12.VI.2016	Equipe Carste	*T. epitychia* **sp. nov.**
9710	Brasil	MG	Conceição do Mato Dentro	?	-18 56 24.762	-43 24 22.453	31.V-12.VI.2016	Equipe Carste	*T. epitychia* **sp. nov.**
9711	Brasil	MG	Conceição do Mato Dentro	?	-18 56 24.762	-43 24 22.453	31.V-12.VI.2016	Equipe Carste	*T. epitychia* **sp. nov.**
9712	Brasil	MG	Conceição do Mato Dentro	?	-18 56 24.762	-43 24 22.453	31.V-12.VI.2016	Equipe Carste	*T. epitychia* **sp. nov.**
9713	Brasil	MG	Conceição do Mato Dentro	?	-18 56 14.049	-43 24 43.816	31.V-12.VI.2016	Equipe Carste	*T. epitychia* **sp. nov.**
9714	Brasil	MG	Conceição do Mato Dentro	?	-18 56 14.049	-43 24 43.816	31.V-12.VI.2016	Equipe Carste	*T. epitychia* **sp. nov.**
9715	Brasil	MG	Conceição do Mato Dentro	?	-18 56 14.049	-43 24 43.816	31.V-12.VI.2016	Equipe Carste	*T. epitychia* **sp. nov.**
9716	Brasil	MG	Conceição do Mato Dentro	?	-18 56 14.323	-43 24 41.626	31.V-12.VI.2016	Equipe Carste	*T. epitychia* **sp. nov.**
9717	Brasil	MG	Conceição do Mato Dentro	?	-18 56 14.323	-43 24 41.626	31.V-12.VI.2016	Equipe Carste	*T. epitychia* **sp. nov.**
9719	Brasil	MG	Conceição do Mato Dentro	?	-18 56 14.323	-43 24 41.626	31.V-12.VI.2016	Equipe Carste	*T. epitychia* **sp. nov.**
9720	Brasil	MG	Conceição do Mato Dentro	?	-18 56 14.323	-43 24 41.626	31.V-12.VI.2016	Equipe Carste	*T. epitychia* **sp. nov.**
9721	Brasil	MG	Conceição do Mato Dentro	?	-18 56 14.323	-43 24 41.626	31.V-12.VI.2016	Equipe Carste	*T. epitychia* **sp. nov.**
9722	Brasil	MG	Conceição do Mato Dentro	?	-18 56 14.323	-43 24 41.626	31.V-12.VI.2016	Equipe Carste	*T. epitychia* **sp. nov.**
9723	Brasil	MG	Conceição do Mato Dentro	?	-18 56 14.323	-43 24 41.626	31.V-12.VI.2016	Equipe Carste	*T. epitychia* **sp. nov.**
9724	Brasil	MG	Conceição do Mato Dentro	?	-18 56 13.945	-43 24 24.128	31.V-12.VI.2016	Equipe Carste	*T. epitychia* **sp. nov.**
10555	Brasil	MG	Conceição do Mato Dentro	Anglo Monitoramento	-18 56 14.049	-43 24 43.816	22.XI-15.XII.2016	Equipe Carste	*T. epitychia* **sp. nov.**
10557	Brasil	MG	Conceição do Mato Dentro	Anglo Monitoramento	-18 56 14.049	-43 24 43.816	22.XI-15.XII.2016	Equipe Carste	*T. epitychia* **sp. nov.**
10558	Brasil	MG	Conceição do Mato Dentro	Anglo Monitoramento	-18 56 14.049	-43 24 43.816	22.XI-15.XII.2016	Equipe Carste	*T. epitychia* **sp. nov.**
10560	Brasil	MG	Conceição do Mato Dentro	Anglo Monitoramento	-18 56 14.049	-43 24 43.816	22.XI-15.XII.2016	Equipe Carste	*T. epitychia* **sp. nov.**
10561	Brasil	MG	Conceição do Mato Dentro	Anglo Monitoramento	-18 56 14.049	-43 24 43.816	22.XI-15.XII.2016	Equipe Carste	*T. epitychia* **sp. nov.**
10564	Brasil	MG	Conceição do Mato Dentro	Anglo Monitoramento	-18 56 14.049	-43 24 43.816	22.XI-15.XII.2016	Equipe Carste	*T. epitychia* **sp. nov.**
10565	Brasil	MG	Conceição do Mato Dentro	Anglo Monitoramento	-18 56 14.049	-43 24 43.816	22.XI-15.XII.2016	Equipe Carste	*T. epitychia* **sp. nov.**

10566	Brasil	MG	Conceição do Mato Dentro	Anglo Monitoramento	-18 56 14.049	-43 24 43.816	22.XI-15.XII.2016	Equipe Carste	*T. epitychia* **sp. nov.**
10567	Brasil	MG	Conceição do Mato Dentro	Anglo Monitoramento	-18 56 14.049	-43 24 43.816	22.XI-15.XII.2016	Equipe Carste	*T. epitychia* **sp. nov.**
10568	Brasil	MG	Conceição do Mato Dentro	Anglo Monitoramento	-18 56 14.049	-43 24 43.816	22.XI-15.XII.2016	Equipe Carste	*T. epitychia* **sp. nov.**
10569	Brasil	MG	Conceição do Mato Dentro	Anglo Monitoramento	-18 56 14.049	-43 24 43.816	22.XI-15.XII.2016	Equipe Carste	*T. epitychia* **sp. nov.**
10570	Brasil	MG	Conceição do Mato Dentro	Anglo Monitoramento	-18 56 14.049	-43 24 43.816	22.XI-15.XII.2016	Equipe Carste	*T. epitychia* **sp. nov.**
10571	Brasil	MG	Conceição do Mato Dentro	Anglo Monitoramento	-18 56 14.049	-43 24 43.816	22.XI-15.XII.2016	Equipe Carste	*T. epitychia* **sp. nov.**
10572	Brasil	MG	Conceição do Mato Dentro	Anglo Monitoramento	-18 56 14.049	-43 24 43.816	22.XI-15.XII.2016	Equipe Carste	*T. epitychia* **sp. nov.**
10575	Brasil	MG	Conceição do Mato Dentro	Anglo Monitoramento	-18 56 14.049	-43 24 43.816	22.XI-15.XII.2016	Equipe Carste	*T. epitychia* **sp. nov.**
10576	Brasil	MG	Conceição do Mato Dentro	Anglo Monitoramento	-18 56 14.049	-43 24 43.816	21.XI-15.XII.2016	Equipe Carste	*T. epitychia* **sp. nov.**
10577	Brasil	MG	Conceição do Mato Dentro	Anglo Monitoramento	-18 56 14.049	-43 24 43.816	21.XI-15.XII.2016	Equipe Carste	*T. epitychia* **sp. nov.**
10578*	Brasil	MG	Conceição do Mato Dentro	Anglo Monitoramento	-18 56 14.049	-43 24 43.816	21.XI-15.XII.2016	Equipe Carste	*T. epitychia* **sp. nov.**
10580**	Brasil	MG	Conceição do Mato Dentro	Anglo Monitoramento	-18 56 14.049	-43 24 43.816	21.XI-15.XII.2016	Equipe Carste	*T. epitychia* **sp. nov.**
10581	Brasil	MG	Conceição do Mato Dentro	Anglo Monitoramento	-18 56 14.049	-43 24 43.816	21.XI-15.XII.2016	Equipe Carste	*T. epitychia* **sp. nov.**
10583	Brasil	MG	Conceição do Mato Dentro	Anglo Monitoramento	-18 56 14.049	-43 24 43.816	21.XI-15.XII.2016	Equipe Carste	*T. epitychia* **sp. nov.**
10584	Brasil	MG	Conceição do Mato Dentro	Anglo Monitoramento	-18 56 14.049	-43 24 43.816	21.XI-15.XII.2016	Equipe Carste	*T. epitychia* **sp. nov.**
10585**	Brasil	MG	Conceição do Mato Dentro	Anglo Monitoramento	-18 56 14.049	-43 24 43.816	21.XI-15.XII.2016	Equipe Carste	*T. epitychia* **sp. nov.**
10586	Brasil	MG	Conceição do Mato Dentro	Anglo Monitoramento	-18 56 14.049	-43 24 43.816	21.XI-15.XII.2016	Equipe Carste	*T. epitychia* **sp. nov.**
10587	Brasil	MG	Conceição do Mato Dentro	CSS-0113	-18 56 14.420	-43 24 41.522	22.XI-15.XII.2016	Equipe Carste	*T. epitychia* **sp. nov.**
10589	Brasil	MG	Conceição do Mato Dentro	CSS-0113	-18 56 14.420	-43 24 41.522	22.XI-15.XII.2016	Equipe Carste	*T. epitychia* **sp. nov.**
10590	Brasil	MG	Conceição do Mato Dentro	CSS-0113	-18 56 14.420	-43 24 41.522	22.XI-15.XII.2016	Equipe Carste	*T. epitychia* **sp. nov.**
10591	Brasil	MG	Conceição do Mato Dentro	CSS-0113	-18 56 14.420	-43 24 41.522	22.XI-15.XII.2016	Equipe Carste	*T. epitychia* **sp. nov.**
10592	Brasil	MG	Conceição do Mato Dentro	CSS-0113	-18 56 14.420	-43 24 41.522	22.XI-15.XII.2016	Equipe Carste	*T. epitychia* **sp. nov.**
10593	Brasil	MG	Conceição do Mato Dentro	CSS-0113	-18 56 14.420	-43 24 41.522	22.XI-15.XII.2016	Equipe Carste	*T. epitychia* **sp. nov.**
10594	Brasil	MG	Conceição do Mato Dentro	CSS-0113	-18 56 14.420	-43 24 41.522	22.XI-15.XII.2016	Equipe Carste	*T. epitychia* **sp. nov.**
10595	Brasil	MG	Conceição do Mato Dentro	CSS-0113	-18 56 14.420	-43 24 41.522	22.XI-15.XII.2016	Equipe Carste	*T. epitychia* **sp. nov.**
10601	Brasil	MG	Conceição do Mato Dentro	CSS-0115	-18 57 18.687	-43 24 41.051	22.XI-15.XII.2016	Equipe Carste	*T. epitychia* **sp. nov.**
10602	Brasil	MG	Conceição do Mato Dentro	CSS-0116	-18 56 13.978	-43 24 24.128	22.XI-15.XII.2016	Equipe Carste	*T. epitychia* **sp. nov.**
10611	Brasil	MG	Conceição do Mato Dentro	CSS-001	-18 54 58.878	-43 25 40.644	04-15.VII.2016	Equipe Carste	*T. epitychia* **sp. nov.**
10626	Brasil	MG	Conceição do Mato Dentro	Anglo Monitoramento	-18 56 14.049	-43 24 43.816	22.XI-15.XII.2016	Equipe Carste	*T. epitychia* **sp. nov.**
10627	Brasil	MG	Conceição do Mato Dentro	Anglo Monitoramento	-18 56 14.049	-43 24 43.816	22.XI-15.XII.2016	Equipe Carste	*T. epitychia* **sp. nov.**
10628	Brasil	MG	Conceição do Mato Dentro	Anglo Monitoramento	-18 56 14.049	-43 24 43.816	22.XI-15.XII.2016	Equipe Carste	*T. epitychia* **sp. nov.**
10629	Brasil	MG	Conceição do Mato Dentro	Anglo Monitoramento	-18 56 14.049	-43 24 43.816	22.XI-15.XII.2016	Equipe Carste	*T. epitychia* **sp. nov.**
10630	Brasil	MG	Conceição do Mato Dentro	Anglo Monitoramento	-18 56 14.049	-43 24 43.816	22.XI-15.XII.2016	Equipe Carste	*T. epitychia* **sp. nov.**
10632	Brasil	MG	Conceição do Mato Dentro	CSS-0116	-18 56 13.979	-43 24 24.129	22.XI-15.XII.2016	Equipe Carste	*T. epitychia* **sp. nov.**
10634	Brasil	MG	Conceição do Mato Dentro	CSS-0113	-18 56 14.420	-43 24 41.522	31.V-12.VII.2016	Equipe Carste	*T. epitychia* **sp. nov.**
10635	Brasil	MG	Conceição do Mato Dentro	CSS-0113	-18 56 14.420	-43 24 41.522	31.V-12.VII.2016	Equipe Carste	*T. epitychia* **sp. nov.**
10636	Brasil	MG	Conceição do Mato Dentro	CSS-0113	-18 56 14.323	-43 24 41.626	31.V-12.VII.2016	Equipe Carste	*T. epitychia* **sp. nov.**
10637	Brasil	MG	Conceição do Mato Dentro	CSS-0113	-18 56 14.323	-43 24 41.626	31.V-12.VII.2016	Equipe Carste	*T. epitychia* **sp. nov.**
10639	Brasil	MG	Conceição do Mato Dentro	CSS-0113	-18 56 14.323	-43 24 41.626	31.V-12.VII.2016	Equipe Carste	*T. epitychia* **sp. nov.**
10640	Brasil	MG	Conceição do Mato Dentro	CSS-0113	-18 56 14.323	-43 24 41.626	31.V-12.VII.2016	Equipe Carste	*T. epitychia* **sp. nov.**
10641	Brasil	MG	Conceição do Mato Dentro	CSS-0113	-18 56 14.323	-43 24 41.626	31.V-12.VII.2016	Equipe Carste	*T. epitychia* **sp. nov.**
10643	Brasil	MG	Conceição do Mato Dentro	CSS-0101	-18 56 40.835	-43 24 19.225	31.V-12.VII.2016	Equipe Carste	*T. epitychia* **sp. nov.**
10649	Brasil	MG	Conceição do Mato Dentro	CSS-0113	-18 56 14.420	-43 24 41.522	31.V-12.VII.2016	Equipe Carste	*T. epitychia* **sp. nov.**
10650	Brasil	MG	Conceição do Mato Dentro	?	-18 56 14.049	-43 24 43.816	31.V-12.VII.2016	Equipe Carste	*T. epitychia* **sp. nov.**
10651	Brasil	MG	Conceição do Mato Dentro	?	-18 56 14.049	-43 24 43.816	31.V-12.VII.2016	Equipe Carste	*T. epitychia* **sp. nov.**
10652	Brasil	MG	Conceição do Mato Dentro	?	-18 56 14.049	-43 24 43.816	31.V-12.VII.2016	Equipe Carste	*T. epitychia* **sp. nov.**
10653**	Brasil	MG	Conceição do Mato Dentro	?	-18 56 14.049	-43 24 43.816	31.V-12.VII.2016	Equipe Carste	*T. epitychia* **sp. nov.**
10654	Brasil	MG	Conceição do Mato Dentro	Anglo Monitoramento	-18 56 14.049	-43 24 43.816	31.V-12.VII.2016	Equipe Carste	*T. epitychia* **sp. nov.**
10655	Brasil	MG	Conceição do Mato Dentro	Anglo Monitoramento	-18 56 14.049	-43 24 43.816	31.V-12.VII.2016	Equipe Carste	*T. epitychia* **sp. nov.**
10656	Brasil	MG	Conceição do Mato Dentro	CSS-0115	-18 56 13.577	-43 24 41.803	31.V-12.VII.2016	Equipe Carste	*T. epitychia* **sp. nov.**
10670	Brasil	MG	Conceição do Mato Dentro	CMN - 03	-18 59 1.421	-43 24 35.564	12.III-02.IV.2014	Equipe Carste	*T. epitychia* **sp. nov.**
10671	Brasil	MG	Conceição do Mato Dentro	CMN - 03	-18 59 1.421	-43 24 35.564	12.III-02.IV.2014	Equipe Carste	*T. epitychia* **sp. nov.**
10673	Brasil	MG	Conceição do Mato Dentro	?	-18 56 24.762	-43 24 22.453	31.V-12.VII.2016	Equipe Carste	*T. epitychia* **sp. nov.**
10677	Brasil	MG	Conceição do Mato Dentro	FSS-0081	-18 56 46.457	-43 24 26.281	31.V-12.VII.2016	Equipe Carste	*T. epitychia* **sp. nov.**
10682	Brasil	MG	Conceição do Mato Dentro	Anglo Monitoramento	-18 56 14.049	-43 24 43.816	22.XI-15.XII.2016	Equipe Carste	*T. epitychia* **sp. nov.**
10683	Brasil	MG	Conceição do Mato Dentro	Anglo Monitoramento	-18 56 14.049	-43 24 43.816	22.XI-15.XII.2016	Equipe Carste	*T. epitychia* **sp. nov.**
10684	Brasil	MG	Conceição do Mato Dentro	Anglo Monitoramento	-18 56 14.049	-43 24 43.816	22.XI-15.XII.2016	Equipe Carste	*T. epitychia* **sp. nov.**

10685	Brasil	MG	Conceição do Mato Dentro	Anglo Monitoramento	-18 56 14.049	-43 24 43.816	22.XI-15.XII.2016	Equipe Carste	*T. epitychia* **sp. nov.**
10686	Brasil	MG	Conceição do Mato Dentro	Anglo Monitoramento	-18 56 14.049	-43 24 43.816	22.XI-15.XII.2016	Equipe Carste	*T. epitychia* **sp. nov.**
10687	Brasil	MG	Conceição do Mato Dentro	Anglo Monitoramento	-18 56 14.049	-43 24 43.816	22.XI-15.XII.2016	Equipe Carste	*T. epitychia* **sp. nov.**
10688	Brasil	MG	Conceição do Mato Dentro	CSS-0118	-18 56 14.049	-43 24 43.816	22.XI-15.XII.2016	Equipe Carste	*T. epitychia* **sp. nov.**
10690	Brasil	MG	Conceição do Mato Dentro	CSS-0115	-18 57 18.687	-43 24 41.051	22.XI-15.XII.2016	Equipe Carste	*T. epitychia* **sp. nov.**
10691	Brasil	MG	Conceição do Mato Dentro	CSS-0113	-18 56 14.420	-43 24 41.522	22.XI-15.XII.2016	Equipe Carste	*T. epitychia* **sp. nov.**
10692**	Brasil	MG	Conceição do Mato Dentro	?	-18 56 14.049	-43 24 43.816	22.XI-15.XII.2016	Equipe Carste	*T. epitychia* **sp. nov.**
10693	Brasil	MG	Conceição do Mato Dentro	FSS-0081	-18 56 46.457	-43 24 26.281	22.XI-15.XII.2016	Equipe Carste	*T. epitychia* **sp. nov.**
10694	Brasil	MG	Conceição do Mato Dentro	FSS-0081	-18 56 46.390	-43 24 26.077	22.XI-15.XII.2016	Equipe Carste	*T. epitychia* **sp. nov.**
10695	Brasil	MG	Conceição do Mato Dentro	SPT - 0045	-19 10 12.648	-43 16 22.504	20-22.XII.2016	Equipe Carste	*T. epitychia* **sp. nov.**
10696	Brasil	MG	Conceição do Mato Dentro	SPT - 0045	-19 10 12.648	-43 16 22.504	20-22.XII.2016	Equipe Carste	*T. epitychia* **sp. nov.**
10697	Brasil	MG	Conceição do Mato Dentro	SPT - 0045	-19 10 12.648	-43 16 22.504	20-22.XII.2016	Equipe Carste	*T. epitychia* **sp. nov.**
10698	Brasil	MG	Conceição do Mato Dentro	SPT - 0045	-19 10 12.648	-43 16 22.504	20-22.XII.2016	Equipe Carste	*T. epitychia* **sp. nov.**
10699	Brasil	MG	Conceição do Mato Dentro	SPT - 0045	-19 10 12.648	-43 16 22.504	20-22.XII.2016	Equipe Carste	*T. epitychia* **sp. nov.**
10700	Brasil	MG	Conceição do Mato Dentro	SPT - 0045	-19 10 12.648	-43 16 22.504	20-22.XII.2016	Equipe Carste	*T. epitychia* **sp. nov.**
10701	Brasil	MG	Conceição do Mato Dentro	SPT - 0045	-19 10 12.648	-43 16 22.504	20-22.XII.2016	Equipe Carste	*T. epitychia* **sp. nov.**
10702	Brasil	MG	Conceição do Mato Dentro	SPT - 0102	-19 09 57.795	-43 16 30.462	14-21.III.2017	Equipe Carste	*T. epitychia* **sp. nov.**
10728	Brasil	MG	Conceição do Mato Dentro	CAS-01	-18 52 39.558	-43 31 20.566	04-15.VII.2016	Equipe Carste	*T. epitychia* **sp. nov.**
10833	Brasil	MG	Conceição do Mato Dentro	SPT 0316	-19 13 15.392	-43 23 23.301	06-10.V.2017	Equipe Carste	*T. epitychia* **sp. nov.**
10834	Brasil	MG	Conceição do Mato Dentro	SPT 0316	-19 13 15.392	-43 23 23.301	06-07.IV.2017	Equipe Carste	*T. epitychia* **sp. nov.**
10838	Brasil	MG	Conceição do Mato Dentro	SPT 0045	-19 10 12.648	-43 16 22.504	06-10.V.2017	Equipe Carste	*T. epitychia* **sp. nov.**
10839	Brasil	MG	Conceição do Mato Dentro	SPT 0045	-19 10 12.648	-43 16 22.504	06-10.V.2017	Equipe Carste	*T. epitychia* **sp. nov.**
10845	Brasil	MG	Conceição do Mato Dentro	SPT 0100	-19 09 59.699	-43 16 32.393	29-30.III.2017	Equipe Carste	*T. epitychia* **sp. nov.**
10854	Brasil	MG	Conceição do Mato Dentro	SPT 0160	-19 09 51.760	-43 16 42.436	26.VII-13..X.2016	Equipe Carste	*T. epitychia* **sp. nov.**
10855	Brasil	MG	Conceição do Mato Dentro	SPT 0160	-19 09 51.761	-43 16 42.573	16-26.I.2017	Equipe Carste	*T. epitychia* **sp. nov.**
10856	Brasil	MG	Conceição do Mato Dentro	SPT 0160	-19 09 51.761	-43 16 42.573	16-26.I.2017	Equipe Carste	*T. epitychia* **sp. nov.**
10857	Brasil	MG	Conceição do Mato Dentro	SPT 0160	-19 09 51.761	-43 16 42.573	16-26.I.2017	Equipe Carste	*T. epitychia* **sp. nov.**
10858	Brasil	MG	Conceição do Mato Dentro	SPT 0160	-19 09 51.761	-43 16 42.573	16-26.I.2017	Equipe Carste	*T. epitychia* **sp. nov.**
10859	Brasil	MG	Conceição do Mato Dentro	SPT 0160	-19 09 51.761	-43 16 42.573	16-26.I.2017	Equipe Carste	*T. epitychia* **sp. nov.**
10861	Brasil	MG	Conceição do Mato Dentro	SPT 0160	-19 09 51.761	-43 16 42.573	16-26.I.2017	Equipe Carste	*T. epitychia* **sp. nov.**
10871	Brasil	MG	Conceição do Mato Dentro	SPT 0465	-19 13 14.148	-43 23 22.423	26.VII-13..X.2016	Equipe Carste	*T. epitychia* **sp. nov.**
10872	Brasil	MG	Conceição do Mato Dentro	SPT 0465	-19 13 14.148	-43 23 22.423	26.VII-13..X.2016	Equipe Carste	*T. epitychia* **sp. nov.**
10878	Brasil	MG	Conceição do Mato Dentro	SPT 0440	-19 12 54.438	-43 28 38.122	16-26.I.2017	Equipe Carste	*T. epitychia* **sp. nov.**
10879	Brasil	MG	Conceição do Mato Dentro	SPT 0438	-19 12 53.486	-43 22 55.577	16-26.I.2017	Equipe Carste	*T. epitychia* **sp. nov.**
10880	Brasil	MG	Conceição do Mato Dentro	SPT 0438	-19 12 53.486	-43 22 55.577	16-26.I.2017	Equipe Carste	*T. epitychia* **sp. nov.**
10882	Brasil	MG	Conceição do Mato Dentro	SPT 0030	-19 10 4.216	-43 16 25.090	26.VII-13..X.2016	Equipe Carste	*T. epitychia* **sp. nov.**
10883	Brasil	MG	Conceição do Mato Dentro	SPT 0030	-19 10 4.216	-43 16 25.090	26.VII-13..X.2016	Equipe Carste	*T. epitychia* **sp. nov.**
10884	Brasil	MG	Conceição do Mato Dentro	SPT 0030	-19 10 4.216	-43 16 25.090	16-26.I.2017	Equipe Carste	*T. epitychia* **sp. nov.**
10885	Brasil	MG	Conceição do Mato Dentro	SPT 0030	-19 10 4.216	-43 16 25.090	16-26.I.2017	Equipe Carste	*T. epitychia* **sp. nov.**
10886	Brasil	MG	Conceição do Mato Dentro	SPT 0030	-19 10 4.216	-43 16 25.090	16-26.I.2017	Equipe Carste	*T. epitychia* **sp. nov.**
10887	Brasil	MG	Conceição do Mato Dentro	SPT 0036	-19 10 2.596	-43 16 25.758	26.VII-13..X.2016	Equipe Carste	*T. epitychia* **sp. nov.**
10888	Brasil	MG	Conceição do Mato Dentro	SPT 0036	-19 10 2.596	-43 16 25.758	26.VII-13..X.2016	Equipe Carste	*T. epitychia* **sp. nov.**
10889	Brasil	MG	Conceição do Mato Dentro	SPT 0036	-19 10 2.596	-43 16 25.758	16-26.I.2017	Equipe Carste	*T. epitychia* **sp. nov.**
10892	Brasil	MG	Conceição do Mato Dentro	SPT 0351	-19 13 17.248	-43 23 42.081	16-26.I.2017	Equipe Carste	*T. epitychia* **sp. nov.**
10893	Brasil	MG	Conceição do Mato Dentro	SPT 0351	-19 13 17.248	-43 23 42.081	16-26.I.2017	Equipe Carste	*T. epitychia* **sp. nov.**
10899	Brasil	MG	Conceição do Mato Dentro	SPT 0285	-19 09 56.488	-43 16 12.576	26.VII-13..X.2016	Equipe Carste	*T. epitychia* **sp. nov.**
10900	Brasil	MG	Conceição do Mato Dentro	SPT 0156	-19 09 50.958	-43 16 40.151	26.VII-13..X.2016	Equipe Carste	*T. epitychia* **sp. nov.**
10901	Brasil	MG	Conceição do Mato Dentro	SPT 0156	-19 09 50.958	-43 16 40.151	26.VII-13..X.2016	Equipe Carste	*T. epitychia* **sp. nov.**
10902	Brasil	MG	Conceição do Mato Dentro	SPT 0156	-19 09 50.958	-43 16 40.151	26.VII-13..X.2016	Equipe Carste	*T. epitychia* **sp. nov.**
10906	Brasil	MG	Conceição do Mato Dentro	SPT 0104	-19 09 59.525	-43 16 24.284	26.VII-13..X.2016	Equipe Carste	*T. epitychia* **sp. nov.**
10912	Brasil	MG	Conceição do Mato Dentro	SPT 0438	-19 12 51.535	-43 22 55.596	16-26.I.2017	Equipe Carste	*T. epitychia* **sp. nov.**
10914	Brasil	MG	Conceição do Mato Dentro	SPT 0438	-19 12 51.600	-43 22 55.595	26.VII-13..X.2016	Equipe Carste	*T. epitychia* **sp. nov.**
10916	Brasil	MG	Conceição do Mato Dentro	SPT 0434	-19 12 45.149	-43 22 54.392	16-26.I.2017	Equipe Carste	*T. epitychia* **sp. nov.**
10919	Brasil	MG	Conceição do Mato Dentro	SPT 0585	-19 10 26.215	-43 15 51.934	16-26.I.2017	Equipe Carste	*T. epitychia* **sp. nov.**
10920	Brasil	MG	Conceição do Mato Dentro	SPT 0585	-19 10 26.215	-43 15 51.934	16-26.I.2017	Equipe Carste	*T. epitychia* **sp. nov.**
10921	Brasil	MG	Conceição do Mato Dentro	SPT 0585	-19 10 26.215	-43 15 51.934	16-26.I.2017	Equipe Carste	*T. epitychia* **sp. nov.**
10922	Brasil	MG	Conceição do Mato Dentro	SPT 0585	-19 10 26.215	-43 15 51.934	16-26.I.2017	Equipe Carste	*T. epitychia* **sp. nov.**

10923	Brasil	MG	Conceição do Mato Dentro	SPT 0585	-19 10 26.215	-43 15 51.934	16-26.I.2017	Equipe Carste	*T. epitychia* **sp. nov.**
10929	Brasil	MG	Conceição do Mato Dentro	SPT 0053	-19 10 2.025	-43 16 23.744	26.VII-13..X.2016	Equipe Carste	*T. epitychia* **sp. nov.**
10930	Brasil	MG	Conceição do Mato Dentro	SPT 0053	-19 10 2.025	-43 16 23.744	26.VII-13..X.2016	Equipe Carste	*T. epitychia* **sp. nov.**
10935	Brasil	MG	Conceição do Mato Dentro	SPT 0028	-19 09 56.488	-43 16 12.576	26.VII-13..X.2016	Equipe Carste	*T. epitychia* **sp. nov.**
10936	Brasil	MG	Conceição do Mato Dentro	SPT 0028	-19 09 56.488	-43 16 12.576	26.VII-13..X.2016	Equipe Carste	*T. epitychia* **sp. nov.**
10937	Brasil	MG	Conceição do Mato Dentro	SPT 0055	-19 10 0.056	-43 16 25.373	26.VII-13..X.2016	Equipe Carste	*T. epitychia* **sp. nov.**
10939	Brasil	MG	Conceição do Mato Dentro	SPT 0694	-19 09 36.608	-43 16 35.919	26.VII-13..X.2016	Equipe Carste	*T. epitychia* **sp. nov.**
10940	Brasil	MG	Conceição do Mato Dentro	SPT 0055	-19 10 0.056	-43 16 25.373	26.VII-13..X.2016	Equipe Carste	*T. epitychia* **sp. nov.**
10947	Brasil	MG	Conceição do Mato Dentro	SPT 0028	-19 09 56.422	-43 16 12.508	02-05.V.2017	Equipe Carste	*T. epitychia* **sp. nov.**
10948	Brasil	MG	Conceição do Mato Dentro	SPT 0028	-19 09 56.422	-43 16 12.508	02-05.V.2017	Equipe Carste	*T. epitychia* **sp. nov.**
10950	Brasil	MG	Conceição do Mato Dentro	SPT 0045	-19 10 12.648	-43 16 22.504	06-10.V.2017	Equipe Carste	*T. epitychia* **sp. nov.**
10952	Brasil	MG	Conceição do Mato Dentro	SPT 0045	-19 10 12.648	-43 16 22.504	06-10.V.2017	Equipe Carste	*T. epitychia* **sp. nov.**
10953	Brasil	MG	Conceição do Mato Dentro	SPT 0045	-19 10 12.648	-43 16 22.504	06-10.V.2017	Equipe Carste	*T. epitychia* **sp. nov.**
10954	Brasil	MG	Conceição do Mato Dentro	SPT 0160	-19 09 51.760	-43 16 42.436	02-05.V.2017	Equipe Carste	*T. epitychia* **sp. nov.**
10955	Brasil	MG	Conceição do Mato Dentro	SPT 0160	-19 09 51.760	-43 16 42.436	02-05.V.2017	Equipe Carste	*T. epitychia* **sp. nov.**
10956	Brasil	MG	Conceição do Mato Dentro	SPT 0160	-19 09 51.760	-43 16 42.436	02-05.V.2017	Equipe Carste	*T. epitychia* **sp. nov.**
10957	Brasil	MG	Conceição do Mato Dentro	SPT 0160	-19 09 51.760	-43 16 42.436	02-05.V.2017	Equipe Carste	*T. epitychia* **sp. nov.**
10958	Brasil	MG	Conceição do Mato Dentro	SPT 0160	-19 09 51.760	-43 16 42.436	02-05.V.2017	Equipe Carste	*T. epitychia* **sp. nov.**
10959	Brasil	MG	Conceição do Mato Dentro	SPT 0160	-19 09 51.760	-43 16 42.436	02-05.V.2017	Equipe Carste	*T. epitychia* **sp. nov.**
10960	Brasil	MG	Conceição do Mato Dentro	SPT 0160	-19 09 51.760	-43 16 42.436	02-05.V.2017	Equipe Carste	*T. epitychia* **sp. nov.**
10961	Brasil	MG	Conceição do Mato Dentro	SPT 0160	-19 09 51.760	-43 16 42.436	02-05.V.2017	Equipe Carste	*T. epitychia* **sp. nov.**
10962	Brasil	MG	Conceição do Mato Dentro	SPT 0160	-19 09 51.760	-43 16 42.436	02-05.V.2017	Equipe Carste	*T. epitychia* **sp. nov.**
10963	Brasil	MG	Conceição do Mato Dentro	SPT 0104	-19 09 59.525	-43 16 24.284	02-05.V.2017	Equipe Carste	*T. epitychia* **sp. nov.**
10964	Brasil	MG	Conceição do Mato Dentro	SPT 0104	-19 09 59.525	-43 16 24.284	02-05.V.2017	Equipe Carste	*T. epitychia* **sp. nov.**
10965	Brasil	MG	Conceição do Mato Dentro	SPT 0104	-19 09 59.525	-43 16 24.284	02-05.V.2017	Equipe Carste	*T. epitychia* **sp. nov.**
10966	Brasil	MG	Conceição do Mato Dentro	SPT 0104	-19 09 59.525	-43 16 24.284	02-05.V.2017	Equipe Carste	*T. epitychia* **sp. nov.**
10967	Brasil	MG	Conceição do Mato Dentro	SPT 0104	-19 09 59.525	-43 16 24.284	02-05.V.2017	Equipe Carste	*T. epitychia* **sp. nov.**
10968	Brasil	MG	Conceição do Mato Dentro	SPT 0104	-19 09 59.525	-43 16 24.284	02-05.V.2017	Equipe Carste	*T. epitychia* **sp. nov.**
10969	Brasil	MG	Conceição do Mato Dentro	SPT 0104	-19 09 59.525	-43 16 24.284	02-05.V.2017	Equipe Carste	*T. epitychia* **sp. nov.**
10970	Brasil	MG	Conceição do Mato Dentro	SPT 0104	-19 09 59.525	-43 16 24.284	02-05.V.2017	Equipe Carste	*T. epitychia* **sp. nov.**
10973	Brasil	MG	Conceição do Mato Dentro	SPT 0053	-19 10 1.862	-43 16 23.780	02-05.V.2017	Equipe Carste	*T. epitychia* **sp. nov.**
10974	Brasil	MG	Conceição do Mato Dentro	SPT 0053	-19 10 1.862	-43 16 23.780	02-05.V.2017	Equipe Carste	*T. epitychia* **sp. nov.**
10975	Brasil	MG	Conceição do Mato Dentro	SPT 0053	-19 10 1.862	-43 16 23.780	02-05.V.2017	Equipe Carste	*T. epitychia* **sp. nov.**
10976	Brasil	MG	Conceição do Mato Dentro	SPT 0053	-19 10 1.862	-43 16 23.780	02-05.V.2017	Equipe Carste	*T. epitychia* **sp. nov.**
10977	Brasil	MG	Conceição do Mato Dentro	SPT 0053	-19 10 1.862	-43 16 23.780	02-05.V.2017	Equipe Carste	*T. epitychia* **sp. nov.**
10978	Brasil	MG	Conceição do Mato Dentro	SPT 0053	-19 10 1.862	-43 16 23.780	02-05.V.2017	Equipe Carste	*T. epitychia* **sp. nov.**
10979	Brasil	MG	Conceição do Mato Dentro	SPT 0053	-19 10 1.862	-43 16 23.780	02-05.V.2017	Equipe Carste	*T. epitychia* **sp. nov.**
10980	Brasil	MG	Conceição do Mato Dentro	SPT 0053	-19 10 1.862	-43 16 23.780	02-05.V.2017	Equipe Carste	*T. epitychia* **sp. nov.**
10985	Brasil	MG	Conceição do Mato Dentro	SPT 0053	-19 10 1.862	-43 16 23.780	02-05.V.2017	Equipe Carste	*T. epitychia* **sp. nov.**
10990	Brasil	MG	Conceição do Mato Dentro	SPT 0297	-19 05 30.795	-43 21 49.689	06-10.V.2017	Equipe Carste	*T. epitychia* **sp. nov.**
10991	Brasil	MG	Conceição do Mato Dentro	SPT 0104	-19 09 59.525	-43 16 24.284	02-05.V.2017	Equipe Carste	*T. epitychia* **sp. nov.**
10992	Brasil	MG	Conceição do Mato Dentro	SPT 0036	-19 10 2.596	-43 16 25.758	02-05.V.2017	Equipe Carste	*T. epitychia* **sp. nov.**
10993	Brasil	MG	Conceição do Mato Dentro	SPT 0151	-19 05 31.388	-43 21 50.539	02-05.V.2017	Equipe Carste	*T. epitychia* **sp. nov.**
10995	Brasil	MG	Conceição do Mato Dentro	SPT 0045	-19 10 12.648	-43 16 22.504	20.XII-22.XII.2016	Equipe Carste	*T. epitychia* **sp. nov.**
10996	Brasil	MG	Conceição do Mato Dentro	SPT 0045	-19 10 12.648	-43 16 22.504	20.XII-22.XII.2016	Equipe Carste	*T. epitychia* **sp. nov.**
10997	Brasil	MG	Conceição do Mato Dentro	SPT 0045	-19 10 12.648	-43 16 22.504	20.XII-22.XII.2016	Equipe Carste	*T. epitychia* **sp. nov.**
10998	Brasil	MG	Conceição do Mato Dentro	SPT 0045	-19 10 12.648	-43 16 22.504	20.XII-22.XII.2016	Equipe Carste	*T. epitychia* **sp. nov.**
10999	Brasil	MG	Conceição do Mato Dentro	SPT 0045	-19 10 12.648	-43 16 22.504	20.XII-22.XII.2016	Equipe Carste	*T. epitychia* **sp. nov.**
11000	Brasil	MG	Conceição do Mato Dentro	SPT 0045	-19 10 12.648	-43 16 22.504	20.XII-22.XII.2016	Equipe Carste	*T. epitychia* **sp. nov.**
11001	Brasil	MG	Conceição do Mato Dentro	SPT 0045	-19 10 12.648	-43 16 22.504	20.XII-22.XII.2016	Equipe Carste	*T. epitychia* **sp. nov.**
11002	Brasil	MG	Conceição do Mato Dentro	SPT 0232	-19 05 33.996	-43 21 40.318	29.XI-16.XII.2016	Equipe Carste	*T. epitychia* **sp. nov.**
11004	Brasil	MG	Conceição do Mato Dentro	SPT 0465	-19 13 14.245	-43 23 22.388	10-15.I.2017	Equipe Carste	*T. epitychia* **sp. nov.**
11005	Brasil	MG	Conceição do Mato Dentro	SPT 0465	-19 13 14.245	-43 23 22.388	10-15.I.2017	Equipe Carste	*T. epitychia* **sp. nov.**
11006	Brasil	MG	Conceição do Mato Dentro	SPT 0465	-19 13 14.245	-43 23 22.388	10-15.I.2017	Equipe Carste	*T. epitychia* **sp. nov.**
11007	Brasil	MG	Conceição do Mato Dentro	SPT 0465	-19 13 14.245	-43 23 22.388	10-15.I.2017	Equipe Carste	*T. epitychia* **sp. nov.**
11010	Brasil	MG	Conceição do Mato Dentro	SPT 0482	-19 12 59.403	-43 23 10.104	10-15.I.2017	Equipe Carste	*T. epitychia* **sp. nov.**
11011	Brasil	MG	Conceição do Mato Dentro	SPT 0482	-19 12 59.403	-43 23 10.104	10-15.I.2017	Equipe Carste	*T. epitychia* **sp. nov.**

11013	Brasil	MG	Conceição do Mato Dentro	SPT 0195	-19 13 4.523	-43 23 7.897	10-15.I.2017	Equipe Carste	*T. epitychia* **sp. nov.**
11014	Brasil	MG	Conceição do Mato Dentro	SPT 0195	-19 13 4.523	-43 23 7.897	10-15.I.2017	Equipe Carste	*T. epitychia* **sp. nov.**
11015	Brasil	MG	Conceição do Mato Dentro	SPT 0195	-19 13 4.523	-43 23 7.897	10-15.I.2017	Equipe Carste	*T. epitychia* **sp. nov.**
11017	Brasil	MG	Conceição do Mato Dentro	SPT 0053	-19 10 2.025	-43 16 23.744	26.VII-13.X.2016	Equipe Carste	*T. epitychia* **sp. nov.**
11019	Brasil	MG	Conceição do Mato Dentro	SPT 0316	-19 13 15.490	-43 23 23.369	26.VII-13.X.2016	Equipe Carste	*T. epitychia* **sp. nov.**
11024	Brasil	MG	Conceição do Mato Dentro	SPT 0156	-19 09 50.958	-43 16 40.151	26.VII-13.X.2016	Equipe Carste	*T. epitychia* **sp. nov.**
11025	Brasil	MG	Conceição do Mato Dentro	SPT 0156	-19 09 50.958	-43 16 40.151	26.VII-13.X.2016	Equipe Carste	*T. epitychia* **sp. nov.**
11026	Brasil	MG	Conceição do Mato Dentro	SPT 0156	-19 09 50.958	-43 16 40.151	26.VII-13.X.2016	Equipe Carste	*T. epitychia* **sp. nov.**
11027	Brasil	MG	Conceição do Mato Dentro	SPT 0156	-19 09 50.958	-43 16 40.151	26.VII-13.X.2016	Equipe Carste	*T. epitychia* **sp. nov.**
11028	Brasil	MG	Conceição do Mato Dentro	SPT 0156	-19 09 50.958	-43 16 40.151	26.VII-13.X.2016	Equipe Carste	*T. epitychia* **sp. nov.**
11029	Brasil	MG	Conceição do Mato Dentro	SPT 0156	-19 09 50.958	-43 16 40.151	26.VII-13.X.2016	Equipe Carste	*T. epitychia* **sp. nov.**
11030	Brasil	MG	Conceição do Mato Dentro	SPT 0156	-19 09 50.958	-43 16 40.151	26.VII-13.X.2016	Equipe Carste	*T. epitychia* **sp. nov.**
11031	Brasil	MG	Conceição do Mato Dentro	SPT 0045	-19 10 12.648	-43 16 22.504	26.VII-13.X.2016	Equipe Carste	*T. epitychia* **sp. nov.**
11033	Brasil	MG	Conceição do Mato Dentro	SPT 0045	-19 10 12.648	-43 16 22.504	26.VII-13.X.2016	Equipe Carste	*T. epitychia* **sp. nov.**
11036	Brasil	MG	Conceição do Mato Dentro	SPT 0161	-19 09 36.921	-43 16 38.072	26.VII-13.X.2016	Equipe Carste	*T. epitychia* **sp. nov.**
11037	Brasil	MG	Conceição do Mato Dentro	SPT 0161	-19 09 36.921	-43 16 38.072	26.VII-13.X.2016	Equipe Carste	*T. epitychia* **sp. nov.**
11038	Brasil	MG	Conceição do Mato Dentro	SPT 0351	-19 13 17.313	-43 23 42.149	26.VII-13.X.2016	Equipe Carste	*T. epitychia* **sp. nov.**
11040	Brasil	MG	Conceição do Mato Dentro	SPT 0482	-19 13 5.452	-43 23 10.011	26.VII-13.X.2016	Equipe Carste	*T. epitychia* **sp. nov.**
11042	Brasil	MG	Conceição do Mato Dentro	SPT 0594	-19 09 36.608	-43 16 35.919	26.VII-13.X.2016	Equipe Carste	*T. epitychia* **sp. nov.**
11043	Brasil	MG	Conceição do Mato Dentro	SPT 0522	-19 13 4.806	-43 23 10.531	26.VII-13.X.2016	Equipe Carste	*T. epitychia* **sp. nov.**
11044	Brasil	MG	Conceição do Mato Dentro	SPT 0500	-19 13 5.446	-43 23 9.361	10-15.I.2017	Equipe Carste	*T. epitychia* **sp. nov.**
11045	Brasil	MG	Conceição do Mato Dentro	SPT 0104	-19 09 59.525	-43 16 24.284	26.VII-13.X.2016	Equipe Carste	*T. epitychia* **sp. nov.**
11048	Brasil	MG	Conceição do Mato Dentro	?	-19 05 29.679	-43 21 52.232	29.XI-16.XII.2016	Equipe Carste	*T. epitychia* **sp. nov.**
11049	Brasil	MG	Conceição do Mato Dentro	SPT 0053	-19 10 1.862	-43 16 23.780	16-26.I.2017	Equipe Carste	*T. epitychia* **sp. nov.**
11050	Brasil	MG	Conceição do Mato Dentro	SPT 0053	-19 10 1.862	-43 16 23.780	16-26.I.2017	Equipe Carste	*T. epitychia* **sp. nov.**
11051	Brasil	MG	Conceição do Mato Dentro	SPT 0053	-19 10 1.862	-43 16 23.780	16-26.I.2017	Equipe Carste	*T. epitychia* **sp. nov.**
11052	Brasil	MG	Conceição do Mato Dentro	SPT 0053	-19 10 1.862	-43 16 23.780	16-26.I.2017	Equipe Carste	*T. epitychia* **sp. nov.**
11053	Brasil	MG	Conceição do Mato Dentro	SPT 0053	-19 10 1.862	-43 16 23.780	16-26.I.2017	Equipe Carste	*T. epitychia* **sp. nov.**
11054	Brasil	MG	Conceição do Mato Dentro	SPT 0053	-19 10 1.862	-43 16 23.780	16-26.I.2017	Equipe Carste	*T. epitychia* **sp. nov.**
11056	Brasil	MG	Conceição do Mato Dentro	SPT 0053	-19 10 1.862	-43 16 23.780	16-26.I.2017	Equipe Carste	*T. epitychia* **sp. nov.**
11058	Brasil	MG	Conceição do Mato Dentro	SPT 0585	-19 10 26.215	-43 15 51.934	16-26.I.2017	Equipe Carste	*T. epitychia* **sp. nov.**
11059	Brasil	MG	Conceição do Mato Dentro	SPT 0585	-19 10 26.215	-43 15 51.934	16-26.I.2017	Equipe Carste	*T. epitychia* **sp. nov.**
11062	Brasil	MG	Conceição do Mato Dentro	SPT 0583	-19 10 21.426	-43 15 51.129	16-26.I.2017	Equipe Carste	*T. epitychia* **sp. nov.**
11072	Brasil	MG	Conceição do Mato Dentro	SPT 0497	-19 13 4.654	-43 23 7.999	10-15.I.2017	Equipe Carste	*T. epitychia* **sp. nov.**
11081	Brasil	MG	Conceição do Mato Dentro	SPT 0081	-19 09 57.239	-43 15 52.169	26.VII-13.X.2016	Equipe Carste	*T. epitychia* **sp. nov.**
11082	Brasil	MG	Conceição do Mato Dentro	SPT 0587	-19 10 24.263	-43 15 51.886	26.VII-13.X.2016	Equipe Carste	*T. epitychia* **sp. nov.**
11086	Brasil	MG	Conceição do Mato Dentro	SPT 0238	-19 05 39.066	-43 21 36.162	26.VII-13.X.2016	Equipe Carste	*T. epitychia* **sp. nov.**
11094	Brasil	MG	Conceição do Mato Dentro	SPT 0131	-19 05 23.666	-43 21 52.633	16-26.I.2017	Equipe Carste	*T. epitychia* **sp. nov.**
11095	Brasil	MG	Conceição do Mato Dentro	SPT 0131	-19 05 23.666	-43 21 52.633	16-26.I.2017	Equipe Carste	*T. epitychia* **sp. nov.**
11096	Brasil	MG	Conceição do Mato Dentro	SPT 0104	-19 09 59.525	-43 16 24.284	16-26.I.2017	Equipe Carste	*T. epitychia* **sp. nov.**
11097	Brasil	MG	Conceição do Mato Dentro	SPT 0161	-19 09 36.921	-43 16 38.072	16-26.I.2017	Equipe Carste	*T. epitychia* **sp. nov.**
11098	Brasil	MG	Conceição do Mato Dentro	SPT 0158	-19 09 51.121	-43 16 40.218	16-26.I.2017	Equipe Carste	*T. epitychia* **sp. nov.**
11106	Brasil	MG	Conceição do Mato Dentro	SPT 0045	-19 10 12.648	-43 16 22.504	20.XII-22.XII.2016	Equipe Carste	*T. epitychia* **sp. nov.**
11107	Brasil	MG	Conceição do Mato Dentro	SPT 0045	-19 10 12.648	-43 16 22.504	20.XII-22.XII.2016	Equipe Carste	*T. epitychia* **sp. nov.**
11108	Brasil	MG	Conceição do Mato Dentro	SPT 0045	-19 10 12.648	-43 16 22.504	20.XII-22.XII.2016	Equipe Carste	*T. epitychia* **sp. nov.**
11109	Brasil	MG	Conceição do Mato Dentro	SPT 0045	-19 10 12.648	-43 16 22.504	20.XII-22.XII.2016	Equipe Carste	*T. epitychia* **sp. nov.**
11111	Brasil	MG	Conceição do Mato Dentro	SPT 0045	-19 10 12.648	-43 16 22.504	20.XII-22.XII.2016	Equipe Carste	*T. epitychia* **sp. nov.**
11112	Brasil	MG	Conceição do Mato Dentro	SPT 0045	-19 10 12.648	-43 16 22.504	20.XII-22.XII.2016	Equipe Carste	*T. epitychia* **sp. nov.**
11113	Brasil	MG	Conceição do Mato Dentro	SPT 0045	-19 10 12.648	-43 16 22.504	20.XII-22.XII.2016	Equipe Carste	*T. epitychia* **sp. nov.**
11114	Brasil	MG	Conceição do Mato Dentro	SPT 0045	-19 10 12.648	-43 16 22.504	20.XII-22.XII.2016	Equipe Carste	*T. epitychia* **sp. nov.**
11115	Brasil	MG	Conceição do Mato Dentro	SPT 0045	-19 10 12.648	-43 16 22.504	20.XII-22.XII.2016	Equipe Carste	*T. epitychia* **sp. nov.**
11116	Brasil	MG	Conceição do Mato Dentro	SPT 0045	-19 10 12.648	-43 16 22.504	20.XII-22.XII.2016	Equipe Carste	*T. epitychia* **sp. nov.**
11117	Brasil	MG	Conceição do Mato Dentro	SPT 0045	-19 10 12.648	-43 16 22.504	20.XII-22.XII.2016	Equipe Carste	*T. epitychia* **sp. nov.**
11118	Brasil	MG	Conceição do Mato Dentro	SPT 0045	-19 10 12.648	-43 16 22.504	20.XII-22.XII.2016	Equipe Carste	*T. epitychia* **sp. nov.**
11119	Brasil	MG	Conceição do Mato Dentro	SPT 0045	-19 10 12.648	-43 16 22.504	20.XII-22.XII.2016	Equipe Carste	*T. epitychia* **sp. nov.**
11120	Brasil	MG	Conceição do Mato Dentro	SPT 0045	-19 10 12.648	-43 16 22.504	20.XII-22.XII.2016	Equipe Carste	*T. epitychia* **sp. nov.**
11127	Brasil	MG	Conceição do Mato Dentro	?	-19 05 29.484	-43 21 48.573	29.XI-16.XII.2016	Equipe Carste	*T. epitychia* **sp. nov.**

11129	Brasil	MG	Conceição do Mato Dentro	SPT 0538	-19 13 16.512	-43 23 17.504	10-15.I.2017	Equipe Carste	*T. epitychia* **sp. nov.**
11130	Brasil	MG	Conceição do Mato Dentro	SPT 0055	-19 10 0.056	-43 16 25.373	26.VII-13.X.2016	Equipe Carste	*T. epitychia* **sp. nov.**
11131	Brasil	MG	Conceição do Mato Dentro	SPT 0030	-19 10 4.216	-43 16 25.090	26.VII-13.X.2016	Equipe Carste	*T. epitychia* **sp. nov.**
11132	Brasil	MG	Conceição do Mato Dentro	SPT 0031	-19 10 3.496	-43 16 24.619	16-26.I.2017	Equipe Carste	*T. epitychia* **sp. nov.**
11133	Brasil	MG	Conceição do Mato Dentro	SPT 0033	-19 13 8.812	-43 23 14.806	10-15.I.2017	Equipe Carste	*T. epitychia* **sp. nov.**
11134	Brasil	MG	Conceição do Mato Dentro	SPT 0512	-19 02 9.944	-43 23 9.876	10-15.I.2017	Equipe Carste	*T. epitychia* **sp. nov.**
11135	Brasil	MG	Conceição do Mato Dentro	SPT 0538	-9 13 16.512	-43 23 17.504	10-15.I.2017	Equipe Carste	*T. epitychia* **sp. nov.**
11137	Brasil	MG	Conceição do Mato Dentro	SPT 0458	-19 13 16.524	-43 23 18.942	10-15.I.2017	Equipe Carste	*T. epitychia* **sp. nov.**
11138	Brasil	MG	Conceição do Mato Dentro	SPT 0465	-19 13 14.245	-43 23 22.388	10-15.I.2017	Equipe Carste	*T. epitychia* **sp. nov.**
11140	Brasil	MG	Conceição do Mato Dentro	SPT 0351	-19 13 17.248	-43 23 42.081	16-26.I.2017	Equipe Carste	*T. epitychia* **sp. nov.**
11141	Brasil	MG	Conceição do Mato Dentro	SPT 0344	-19 13 17.199	-43 23 40.266	16-26.I.2017	Equipe Carste	*T. epitychia* **sp. nov.**
11142	Brasil	MG	Conceição do Mato Dentro	SPT 0344	-19 13 17.394	-43 23 40.265	26.VII-13.X.2016	Equipe Carste	*T. epitychia* **sp. nov.**
11147	Brasil	MG	Conceição do Mato Dentro	?	-19 13 8.812	-43 23 14.806	10-15.I.2017	Equipe Carste	*T. epitychia* **sp. nov.**
11148	Brasil	MG	Conceição do Mato Dentro	?	-19 13 7.786	-43 23 12.796	10-15.I.2017	Equipe Carste	*T. epitychia* **sp. nov.**
11159	Brasil	MG	Conceição do Mato Dentro	SPT 0363	-19 13 5.310	-43 23 8.609	26.VII-13.X.2016	Equipe Carste	*T. epitychia* **sp. nov.**
11160	Brasil	MG	Conceição do Mato Dentro	SPT 0569	-19 05 44.217	-43 21 22.905	29.XI-16.XII.2016	Equipe Carste	*T. epitychia* **sp. nov.**
11168	Brasil	MG	Conceição do Mato Dentro	SPT 0045	-19 10 12.648	-43 16 22.504	24-27.IV.2017	Equipe Carste	*T. epitychia* **sp. nov.**
11169	Brasil	MG	Conceição do Mato Dentro	SPT 0045	-19 10 12.648	-43 16 22.504	24-27.IV.2017	Equipe Carste	*T. epitychia* **sp. nov.**
11170	Brasil	MG	Conceição do Mato Dentro	SPT 0045	-19 10 12.648	-43 16 22.504	24-27.IV.2017	Equipe Carste	*T. epitychia* **sp. nov.**
11171	Brasil	MG	Conceição do Mato Dentro	SPT 0045	-19 10 12.648	-43 16 22.504	24-27.IV.2017	Equipe Carste	*T. epitychia* **sp. nov.**
11172	Brasil	MG	Conceição do Mato Dentro	SPT 0045	-19 10 12.648	-43 16 22.504	24-27.IV.2017	Equipe Carste	*T. epitychia* **sp. nov.**
11173	Brasil	MG	Conceição do Mato Dentro	SPT 0045	-19 10 12.648	-43 16 22.504	24-27.IV.2017	Equipe Carste	*T. epitychia* **sp. nov.**
11174	Brasil	MG	Conceição do Mato Dentro	SPT 0045	-19 10 12.648	-43 16 22.504	24-27.IV.2017	Equipe Carste	*T. epitychia* **sp. nov.**
11175	Brasil	MG	Conceição do Mato Dentro	SPT 0045	-19 10 12.648	-43 16 22.504	24-27.IV.2017	Equipe Carste	*T. epitychia* **sp. nov.**
11176	Brasil	MG	Conceição do Mato Dentro	SPT 0045	-19 10 12.648	-43 16 22.504	24-27.IV.2017	Equipe Carste	*T. epitychia* **sp. nov.**
11177	Brasil	MG	Conceição do Mato Dentro	SPT 0045	-19 10 12.648	-43 16 22.504	24-27.IV.2017	Equipe Carste	*T. epitychia* **sp. nov.**
11178	Brasil	MG	Conceição do Mato Dentro	SPT 0045	-19 10 12.648	-43 16 22.504	24-27.IV.2017	Equipe Carste	*T. epitychia* **sp. nov.**
11179	Brasil	MG	Conceição do Mato Dentro	SPT 0045	-19 10 12.648	-43 16 22.504	24-27.IV.2017	Equipe Carste	*T. epitychia* **sp. nov.**
11180	Brasil	MG	Conceição do Mato Dentro	SPT 0045	-19 10 12.648	-43 16 22.504	24-27.IV.2017	Equipe Carste	*T. epitychia* **sp. nov.**
11181	Brasil	MG	Conceição do Mato Dentro	SPT 0045	-19 10 12.648	-43 16 22.504	24-27.IV.2017	Equipe Carste	*T. epitychia* **sp. nov.**
11182	Brasil	MG	Conceição do Mato Dentro	SPT 0045	-19 10 12.648	-43 16 22.504	24-27.IV.2017	Equipe Carste	*T. epitychia* **sp. nov.**
11183	Brasil	MG	Conceição do Mato Dentro	SPT 0045	-19 10 12.648	-43 16 22.504	24-27.IV.2017	Equipe Carste	*T. epitychia* **sp. nov.**
11184	Brasil	MG	Conceição do Mato Dentro	SPT 0045	-19 10 12.648	-43 16 22.504	24-27.IV.2017	Equipe Carste	*T. epitychia* **sp. nov.**
11185	Brasil	MG	Conceição do Mato Dentro	SPT 0045	-19 10 12.648	-43 16 22.504	24-27.IV.2017	Equipe Carste	*T. epitychia* **sp. nov.**
11186	Brasil	MG	Conceição do Mato Dentro	SPT 0045	-19 10 12.648	-43 16 22.504	24-27.IV.2017	Equipe Carste	*T. epitychia* **sp. nov.**
11187	Brasil	MG	Conceição do Mato Dentro	SPT 0045	-19 10 12.648	-43 16 22.504	24-27.IV.2017	Equipe Carste	*T. epitychia* **sp. nov.**
11188	Brasil	MG	Conceição do Mato Dentro	SPT 0045	-19 10 12.648	-43 16 22.504	24-27.IV.2017	Equipe Carste	*T. epitychia* **sp. nov.**
11189	Brasil	MG	Conceição do Mato Dentro	SPT 0045	-19 10 12.648	-43 16 22.504	24-27.IV.2017	Equipe Carste	*T. epitychia* **sp. nov.**
11197	Brasil	MG	Conceição do Mato Dentro	SPT 0138	-19 05 27.611	-43 21 50.131	24-27.IV.2017	Equipe Carste	*T. epitychia* **sp. nov.**
11198	Brasil	MG	Conceição do Mato Dentro	SPT 0138	-19 05 27.611	-43 21 50.131	24-27.IV.2017	Equipe Carste	*T. epitychia* **sp. nov.**
11201	Brasil	MG	Conceição do Mato Dentro	SPT 0150	-19 05 29.679	-43 21 52.232	24-27.IV.2017	Equipe Carste	*T. epitychia* **sp. nov.**
11202	Brasil	MG	Conceição do Mato Dentro	SPT 0150	-19 05 29.679	-43 21 52.232	24-27.IV.2017	Equipe Carste	*T. epitychia* **sp. nov.**
11203	Brasil	MG	Conceição do Mato Dentro	SPT 0150	-19 05 29.679	-43 21 52.232	24-27.IV.2017	Equipe Carste	*T. epitychia* **sp. nov.**
11204	Brasil	MG	Conceição do Mato Dentro	SPT 0150	-19 05 29.679	-43 21 52.232	24-27.IV.2017	Equipe Carste	*T. epitychia* **sp. nov.**
11209	Brasil	MG	Conceição do Mato Dentro	SPT 0104	-19 09 59.525	-43 16 24.284	02-05.V.2017	Equipe Carste	*T. epitychia* **sp. nov.**
11210	Brasil	MG	Conceição do Mato Dentro	SPT 0104	-19 09 59.525	-43 16 24.284	02-05.V.2017	Equipe Carste	*T. epitychia* **sp. nov.**
11211	Brasil	MG	Conceição do Mato Dentro	SPT 0104	-19 09 59.525	-43 16 24.284	02-05.V.2017	Equipe Carste	*T. epitychia* **sp. nov.**
11212	Brasil	MG	Conceição do Mato Dentro	SPT 0104	-19 09 59.525	-43 16 24.284	02-05.V.2017	Equipe Carste	*T. epitychia* **sp. nov.**
11213	Brasil	MG	Conceição do Mato Dentro	SPT 0104	-19 09 59.525	-43 16 24.284	02-05.V.2017	Equipe Carste	*T. epitychia* **sp. nov.**
11218	Brasil	MG	Conceição do Mato Dentro	SPT 0297	-19 05 30.795	-43 21 49.689	10-13.IV.2017	Equipe Carste	*T. epitychia* **sp. nov.**
11219	Brasil	MG	Conceição do Mato Dentro	SPT 0297	-19 05 30.795	-43 21 49.689	10-13.IV.2017	Equipe Carste	*T. epitychia* **sp. nov.**
11220	Brasil	MG	Conceição do Mato Dentro	SPT 0297	-19 05 30.795	-43 21 49.689	10-13.IV.2017	Equipe Carste	*T. epitychia* **sp. nov.**
11221	Brasil	MG	Conceição do Mato Dentro	SPT 0297	-19 05 30.795	-43 21 49.689	10-13.IV.2017	Equipe Carste	*T. epitychia* **sp. nov.**
11236	Brasil	MG	Conceição do Mato Dentro	SPT 0287	-19 05 30.428	-43 21 48.666	10-13.IV.2017	Equipe Carste	*T. epitychia* **sp. nov.**
11241	Brasil	MG	Conceição do Mato Dentro	SPT 0151	-19 05 31.388	-43 21 50.539	10-13.IV.2017	Equipe Carste	*T. epitychia* **sp. nov.**
11247	Brasil	MG	Conceição do Mato Dentro	SPT 0295	-19 05 29.484	-43 21 48.573	10-13.IV.2017	Equipe Carste	*T. epitychia* **sp. nov.**
11289	Brasil	MG	Conceição do Mato Dentro	CSS-023	-18 55 36.468	-43 25 5.020	15-19.II.2016	Equipe Carste	*T. epitychia* **sp. nov.**

11293	Brasil	MG	Conceição do Mato Dentro	CSS-076	-19 05 31.626	-43 25 35.722	29.II-04.III.2016	Equipe Carste	*T. epitychia* **sp. nov.**
11330	Brasil	MG	Conceição do Mato Dentro	CMN-41	-19 00 14.556	-43 23 56.093	12-30.I.2015	Equipe Carste	*T. epitychia* **sp. nov.**
11331	Brasil	MG	Conceição do Mato Dentro	CMN-41	-19 00 14.557	-43 23 56.094	12-30.I.2015	Equipe Carste	*T. epitychia* **sp. nov.**
11344	Brasil	MG	Conceição do Mato Dentro	SPT 0150	-19 05 29.679	-43 21 52.232	26.VII-05.VIII.2016	Equipe Carste	*T. epitychia* **sp. nov.**
11345	Brasil	MG	Conceição do Mato Dentro	SPT 0150	-19 05 29.679	-43 21 52.232	26.VII-05.VIII.2016	Equipe Carste	*T. epitychia* **sp. nov.**
11346	Brasil	MG	Conceição do Mato Dentro	SPT 0150	-19 05 29.679	-43 21 52.232	26.VII-05.VIII.2016	Equipe Carste	*T. epitychia* **sp. nov.**
11347	Brasil	MG	Conceição do Mato Dentro	SPT 0150	-19 05 29.679	-43 21 52.232	26.VII-05.VIII.2016	Equipe Carste	*T. epitychia* **sp. nov.**
11348	Brasil	MG	Conceição do Mato Dentro	SPT 0150	-19 05 29.679	-43 21 52.232	26.VII-05.VIII.2016	Equipe Carste	*T. epitychia* **sp. nov.**
11354	Brasil	MG	Conceição do Mato Dentro	SPT	-19 05 29.718	-43 21 45.628	26.VII-05.VIII.2016	Equipe Carste	*T. epitychia* **sp. nov.**
11377	Brasil	MG	Conceição do Mato Dentro	SPT 0028	-19 09 56.422	-43 16 12.508	02-05.V.2017	Equipe Carste	*T. epitychia* **sp. nov.**
11378	Brasil	MG	Conceição do Mato Dentro	SPT 0028	-19 09 56.422	-43 16 12.508	02-05.V.2017	Equipe Carste	*T. epitychia* **sp. nov.**
11379	Brasil	MG	Conceição do Mato Dentro	SPT 0028	-19 09 56.422	-43 16 12.508	02-05.V.2017	Equipe Carste	*T. epitychia* **sp. nov.**
11380	Brasil	MG	Conceição do Mato Dentro	SPT 0045	-19 10 12.648	-43 16 22.504	06-10.V.2017	Equipe Carste	*T. epitychia* **sp. nov.**
11381	Brasil	MG	Conceição do Mato Dentro	SPT 0045	-19 10 12.648	-43 16 22.504	06-10.V.2017	Equipe Carste	*T. epitychia* **sp. nov.**
11581	Brasil	MG	Conceição do Mato Dentro	Serra do Sapo	-18 55 1.583	-43 25 41.337	15-27.IX.2014	Equipe Carste	*T. epitychia* **sp. nov.**
11582	Brasil	MG	Conceição do Mato Dentro	?	-18 55 1.583	-43 25 41.337	20.VII-06.VIII.2015	Equipe Carste	*T. epitychia* **sp. nov.**
11583	Brasil	MG	Conceição do Mato Dentro	Serra do Sapo	-18 55 0.596	-43 25 40.047		Equipe Carste	*T. epitychia* **sp. nov.**
11599	Brasil	MG	Conceição do Mato Dentro	SPT 0583	-19 10 21.459	-43 15 51.129	12-14.VI.2017	Equipe Carste	*T. epitychia* **sp. nov.**
11601	Brasil	MG	Conceição do Mato Dentro	SPT 0583	-19 10 21.459	-43 15 51.129	12-14.VI.2017	Equipe Carste	*T. epitychia* **sp. nov.**
11602	Brasil	MG	Conceição do Mato Dentro	SPT 0583	-19 10 21.459	-43 15 51.129	12-14.VI.2017	Equipe Carste	*T. epitychia* **sp. nov.**
11603	Brasil	MG	Conceição do Mato Dentro	SPT 0583	-19 10 21.459	-43 15 51.129	12-14.VI.2017	Equipe Carste	*T. epitychia* **sp. nov.**
11604	Brasil	MG	Conceição do Mato Dentro	SPT 0583	-19 10 21.459	-43 15 51.129	12-14.VI.2017	Equipe Carste	*T. epitychia* **sp. nov.**
11608	Brasil	MG	Conceição do Mato Dentro	SPT 0585	-19 10 26.215	-43 15 51.934	12-14.VI.2017	Equipe Carste	*T. epitychia* **sp. nov.**
11611	Brasil	MG	Conceição do Mato Dentro	SPT 0606	-19 05 14.901	-43 21 58.227	19-22.VI.2017	Equipe Carste	*T. epitychia* **sp. nov.**
11621	Brasil	MG	Conceição do Mato Dentro	CSS-009	-18 56 8.880	-43 24 44.138	13-22.XII.2017	Equipe Carste	*T. epitychia* **sp. nov.**
11622	Brasil	MG	Conceição do Mato Dentro	CSS-009	-18 56 8.880	-43 24 44.138	13-22.XII.2017	Equipe Carste	*T. epitychia* **sp. nov.**
12314	Brasil	MG	Conceição do Mato Dentro	CAI-003	-18 53 0.891	-43 25 50.460	26.II-01.III.2018	Equipe Carste	*T. epitychia* **sp. nov.**
12315	Brasil	MG	Conceição do Mato Dentro	CAI-003	-18 53 0.891	-43 25 50.460	26.II-01.III.2018	Equipe Carste	*T. epitychia* **sp. nov.**
12316	Brasil	MG	Conceição do Mato Dentro	CSS-001	-18 54 59.428	-43 25 40.331	26.II-01.III.2018	Equipe Carste	*T. epitychia* **sp. nov.**
12319	Brasil	MG	Conceição do Mato Dentro	CSS-001	-18 54 59.428	-43 25 40.331	26.II-01.III.2018	Equipe Carste	*T. epitychia* **sp. nov.**
12321	Brasil	MG	Conceição do Mato Dentro	CSS-001	-18 54 59.428	-43 25 40.331	26.II-01.III.2018	Equipe Carste	*T. epitychia* **sp. nov.**
12322	Brasil	MG	Conceição do Mato Dentro	CSS-001	-18 54 59.428	-43 25 40.331	26.II-01.III.2018	Equipe Carste	*T. epitychia* **sp. nov.**
12323	Brasil	MG	Conceição do Mato Dentro	CSS-001	-18 54 59.428	-43 25 40.331	26.II-01.III.2018	Equipe Carste	*T. epitychia* **sp. nov.**
12324	Brasil	MG	Conceição do Mato Dentro	CSS-001	-18 54 59.428	-43 25 40.331	26.II-01.III.2018	Equipe Carste	*T. epitychia* **sp. nov.**
12325	Brasil	MG	Conceição do Mato Dentro	CSS-001	-18 54 59.428	-43 25 40.331	26.II-01.III.2018	Equipe Carste	*T. epitychia* **sp. nov.**
12338	Brasil	MG	Conceição do Mato Dentro	FSS-043	-18 54 53.853	-43 25 38.846	17.VII-21.VII.2017	Equipe Carste	*T. epitychia* **sp. nov.**
12340	Brasil	MG	Conceição do Mato Dentro	CSS-001	-18 54 59.428	-43 25 40.331	17-21.VII.2017	Equipe Carste	*T. epitychia* **sp. nov.**
12350	Brasil	MG	Conceição do Mato Dentro	CAI-03	-18 53 0.891	-43 25 50.460	26.II-01.III.2018	Equipe Carste	*T. epitychia* **sp. nov.**
12353	Brasil	MG	Conceição do Mato Dentro	FSS-043	-18 54 53.853	-43 25 38.846	26.II-01.III.2018	Equipe Carste	*T. epitychia* **sp. nov.**
12354	Brasil	MG	Conceição do Mato Dentro	FSS-043	-18 54 53.853	-43 25 38.846	26.II-01.III.2018	Equipe Carste	*T. epitychia* **sp. nov.**
12355	Brasil	MG	Conceição do Mato Dentro	FSS-043	-18 54 53.853	-43 25 38.846	26.II-01.III.2018	Equipe Carste	*T. epitychia* **sp. nov.**
12356	Brasil	MG	Conceição do Mato Dentro	FSS-043	-18 54 53.853	-43 25 38.846	26.II-01.III.2018	Equipe Carste	*T. epitychia* **sp. nov.**
13117	Brasil	MG	Conceição do Mato Dentro	CSS - 0118	-18 56 15.525	-43 24 45.272	18-27.IV.2018	Equipe Carste	*T. epitychia* **sp. nov.**
13118	Brasil	MG	Conceição do Mato Dentro	CSS - 0118	-18 56 15.525	-43 24 45.272	18-27.IV.2018	Equipe Carste	*T. epitychia* **sp. nov.**
13119	Brasil	MG	Conceição do Mato Dentro	CSS - 0118	-18 56 15.525	-43 24 45.272	18-27.IV.2018	Equipe Carste	*T. epitychia* **sp. nov.**
13401	Brasil	MG	Conceição do Mato Dentro	CSS-0001	-18 55 0.421	-43 25 42.339	30.IV-05.V.2018	Equipe Carste	*T. epitychia* **sp. nov.**
13402	Brasil	MG	Conceição do Mato Dentro	CSS-0001	-18 55 0.421	-43 25 42.339	30.IV-05.V.2018	Equipe Carste	*T. epitychia* **sp. nov.**
13421	Brasil	MG	Conceição do Mato Dentro	CSS-0002	-18 55 1.186	-43 25 48.244	30.IV-05.V.2018	Equipe Carste	*T. epitychia* **sp. nov.**
13422	Brasil	MG	Conceição do Mato Dentro	CSS-0002	-18 55 1.186	-43 25 48.244	30.IV-05.V.2018	Equipe Carste	*T. epitychia* **sp. nov.**
13423	Brasil	MG	Conceição do Mato Dentro	CSS-0002	-18 55 1.186	-43 25 48.244	30.IV-05.V.2018	Equipe Carste	*T. epitychia* **sp. nov.**
13424	Brasil	MG	Conceição do Mato Dentro	CSS-0002	-18 55 1.186	-43 25 48.244	30.IV-05.V.2018	Equipe Carste	*T. epitychia* **sp. nov.**
13429	Brasil	MG	Conceição do Mato Dentro	CSS-0003	-18 55 2.172	-43 25 41.775	30.IV-05.V.2018	Equipe Carste	*T. epitychia* **sp. nov.**
13430	Brasil	MG	Conceição do Mato Dentro	CSS-0003	-18 55 2.172	-43 25 41.775	30.IV-05.V.2018	Equipe Carste	*T. epitychia* **sp. nov.**
13431	Brasil	MG	Conceição do Mato Dentro	CSS-0004	-18 54 50.854	-43 25 41.881	30.IV-05.V.2018	Equipe Carste	*T. epitychia* **sp. nov.**
13432	Brasil	MG	Conceição do Mato Dentro	CSS-0004	-18 54 50.854	-43 25 41.881	30.IV-05.V.2018	Equipe Carste	*T. epitychia* **sp. nov.**
13433	Brasil	MG	Conceição do Mato Dentro	CSS-0004	-18 54 50.854	-43 25 41.881	30.IV-05.V.2018	Equipe Carste	*T. epitychia* **sp. nov.**
13434	Brasil	MG	Conceição do Mato Dentro	CSS-0004	-18 54 50.854	-43 25 41.881	30.IV-05.V.2018	Equipe Carste	*T. epitychia* **sp. nov.**

13435	Brasil	MG	Conceição do Mato Dentro	CSS-0004	-18 54 50.854	-43 25 41.881	30.IV-05.V.2018	Equipe Carste	*T. epitychia* **sp. nov.**
13436	Brasil	MG	Conceição do Mato Dentro	CSS-0004	-18 54 50.854	-43 25 41.881	30.IV-05.V.2018	Equipe Carste	*T. epitychia* **sp. nov.**
13437	Brasil	MG	Conceição do Mato Dentro	CSS-0004	-18 54 50.854	-43 25 41.881	30.IV-05.V.2018	Equipe Carste	*T. epitychia* **sp. nov.**
13438	Brasil	MG	Conceição do Mato Dentro	CSS-0004	-18 54 50.854	-43 25 41.881	30.IV-05.V.2018	Equipe Carste	*T. epitychia* **sp. nov.**
13439	Brasil	MG	Conceição do Mato Dentro	CSS-0004	-18 54 50.854	-43 25 41.881	30.IV-05.V.2018	Equipe Carste	*T. epitychia* **sp. nov.**
13440	Brasil	MG	Conceição do Mato Dentro	CSS-0004	-18 54 50.854	-43 25 41.881	30.IV-05.V.2018	Equipe Carste	*T. epitychia* **sp. nov.**
13441	Brasil	MG	Conceição do Mato Dentro	CSS-0004	-18 54 50.854	-43 25 41.881	30.IV-05.V.2018	Equipe Carste	*T. epitychia* **sp. nov.**
13443	Brasil	MG	Conceição do Mato Dentro	CSS-0004	-18 54 50.854	-43 25 41.881	30.IV-05.V.2018	Equipe Carste	*T. epitychia* **sp. nov.**
13445	Brasil	MG	Conceição do Mato Dentro	CSS-0005	-18 55 2.136	-43 25 41.366	30.IV-05.V.2018	Equipe Carste	*T. epitychia* **sp. nov.**
13446	Brasil	MG	Conceição do Mato Dentro	CSS-0005	-18 55 2.136	-43 25 41.366	30.IV-05.V.2018	Equipe Carste	*T. epitychia* **sp. nov.**
13450	Brasil	MG	Conceição do Mato Dentro	CSS-0005	-18 55 2.136	-43 25 41.366	30.IV-05.V.2018	Equipe Carste	*T. epitychia* **sp. nov.**
13453	Brasil	MG	Conceição do Mato Dentro	CSS-0005	-18 55 2.136	-43 25 41.366	30.IV-05.V.2018	Equipe Carste	*T. epitychia* **sp. nov.**
13454	Brasil	MG	Conceição do Mato Dentro	CSS-0006	-18 55 2.632	-43 25 42.250	30.IV-05.V.2018	Equipe Carste	*T. epitychia* **sp. nov.**
13460	Brasil	MG	Conceição do Mato Dentro	CSS-0006	-18 55 2.632	-43 25 42.250	30.IV-05.V.2018	Equipe Carste	*T. epitychia* **sp. nov.**
13461	Brasil	MG	Conceição do Mato Dentro	CSS-0006	-18 55 2.632	-43 25 42.250	30.IV-05.V.2018	Equipe Carste	*T. epitychia* **sp. nov.**
13462	Brasil	MG	Conceição do Mato Dentro	CSS-0006	-18 55 2.632	-43 25 42.250	30.IV-05.V.2018	Equipe Carste	*T. epitychia* **sp. nov.**
13466	Brasil	MG	Conceição do Mato Dentro	CSS-0006	-18 55 2.632	-43 25 42.250	30.IV-05.V.2018	Equipe Carste	*T. epitychia* **sp. nov.**
13467	Brasil	MG	Conceição do Mato Dentro	CSS-0006	-18 55 2.632	-43 25 42.250	30.IV-05.V.2018	Equipe Carste	*T. epitychia* **sp. nov.**
13477	Brasil	MG	Conceição do Mato Dentro	CSS-0024	-18 55 1.461	-43 25 46.157	30.IV-05.V.2018	Equipe Carste	*T. epitychia* **sp. nov.**
13478	Brasil	MG	Conceição do Mato Dentro	CSS-0024	-18 55 1.461	-43 25 46.157	30.IV-05.V.2018	Equipe Carste	*T. epitychia* **sp. nov.**
13479	Brasil	MG	Conceição do Mato Dentro	CSS-0024	-18 55 1.461	-43 25 46.157	30.IV-05.V.2018	Equipe Carste	*T. epitychia* **sp. nov.**
13483	Brasil	MG	Conceição do Mato Dentro	CSS-0024	-18 55 1.461	-43 25 46.157	30.IV-05.V.2018	Equipe Carste	*T. epitychia* **sp. nov.**
13487	Brasil	MG	Conceição do Mato Dentro	CSS-0025	-18 55 1.464	-43 25 46.498	30.IV-05.V.2018	Equipe Carste	*T. epitychia* **sp. nov.**
13488	Brasil	MG	Conceição do Mato Dentro	CSS-0025	-18 55 1.464	-43 25 46.498	30.IV-05.V.2018	Equipe Carste	*T. epitychia* **sp. nov.**
13490	Brasil	MG	Conceição do Mato Dentro	CSS-0074	-18 56 24.097	-43 24 24.545	30.IV-05.V.2018	Equipe Carste	*T. epitychia* **sp. nov.**
13497	Brasil	MG	Conceição do Mato Dentro	CAI-0003	-18 53 1.748	-43 25 51.818	18 -27.IV.2018	Equipe Carste	*T. epitychia* **sp. nov.**
13498	Brasil	MG	Conceição do Mato Dentro	CAI-0003	-18 53 1.748	-43 25 51.818	18 -27.IV.2018	Equipe Carste	*T. epitychia* **sp. nov.**
13500	Brasil	MG	Conceição do Mato Dentro	CAI-0003	-18 53 1.748	-43 25 51.818	18 -27.IV.2018	Equipe Carste	*T. epitychia* **sp. nov.**
13515	Brasil	MG	Conceição do Mato Dentro	CSS-0112	-18 56 14.833	-43 24 51.841	18 -27.IV.2018	Equipe Carste	*T. epitychia* **sp. nov.**
13516	Brasil	MG	Conceição do Mato Dentro	CSS-0113	-18 56 15.896	-43 24 42.978	18 -27.IV.2018	Equipe Carste	*T. epitychia* **sp. nov.**
13518	Brasil	MG	Conceição do Mato Dentro	CSS-0113	-18 56 15.896	-43 24 42.978	18 -27.IV.2018	Equipe Carste	*T. epitychia* **sp. nov.**
13519	Brasil	MG	Conceição do Mato Dentro	CSS-0113	-18 56 15.896	-43 24 42.978	18 -27.IV.2018	Equipe Carste	*T. epitychia* **sp. nov.**
13520	Brasil	MG	Conceição do Mato Dentro	CSS-0113	-18 56 15.896	-43 24 42.978	18 -27.IV.2018	Equipe Carste	*T. epitychia* **sp. nov.**
13521	Brasil	MG	Conceição do Mato Dentro	CSS-0115	-18 56 15.117	-43 24 43.122	18 -27.IV.2018	Equipe Carste	*T. epitychia* **sp. nov.**
13522	Brasil	MG	Conceição do Mato Dentro	Anglo Monitoramento	-18 56 15.525	-43 24 45.272	18 -27.IV.2018	Equipe Carste	*T. epitychia* **sp. nov.**
13523	Brasil	MG	Conceição do Mato Dentro	Anglo Monitoramento	-18 56 15.525	-43 24 45.272	18 -27.IV.2018	Equipe Carste	*T. epitychia* **sp. nov.**
13525	Brasil	MG	Conceição do Mato Dentro	Anglo Monitoramento	-18 56 15.525	-43 24 45.272	18 -27.IV.2018	Equipe Carste	*T. epitychia* **sp. nov.**
13526	Brasil	MG	Conceição do Mato Dentro	Anglo Monitoramento	-18 56 15.525	-43 24 45.272	18 -27.IV.2018	Equipe Carste	*T. epitychia* **sp. nov.**
13527	Brasil	MG	Conceição do Mato Dentro	Anglo Monitoramento	-18 56 15.525	-43 24 45.272	18 -27.IV.2018	Equipe Carste	*T. epitychia* **sp. nov.**
13564	Brasil	MG	Conceição do Mato Dentro	ASS-007	-18 56 40.714	-43 24 24.114	20-29.XI.2018	Equipe Carste	*T. epitychia* **sp. nov.**
13565	Brasil	MG	Conceição do Mato Dentro	CSS-009	-18 56 10.161	-43 24 45.493	20-29.XI.2018	Equipe Carste	*T. epitychia* **sp. nov.**
13566	Brasil	MG	Conceição do Mato Dentro	CSS-009	-18 56 10.161	-43 24 45.493	20-29.XI.2018	Equipe Carste	*T. epitychia* **sp. nov.**
13569	Brasil	MG	Conceição do Mato Dentro	CSS-009	-18 56 10.161	-43 24 45.493	20-29.XI.2018	Equipe Carste	*T. epitychia* **sp. nov.**
13570	Brasil	MG	Conceição do Mato Dentro	CSS-009	-18 56 10.161	-43 24 45.493	20-29.XI.2018	Equipe Carste	*T. epitychia* **sp. nov.**
13571	Brasil	MG	Conceição do Mato Dentro	CSS-009	-18 56 10.161	-43 24 45.493	20-29.XI.2018	Equipe Carste	*T. epitychia* **sp. nov.**
13572	Brasil	MG	Conceição do Mato Dentro	CSS-009	-18 56 10.161	-43 24 45.493	20-29.XI.2018	Equipe Carste	*T. epitychia* **sp. nov.**
13577	Brasil	MG	Conceição do Mato Dentro	CSS-009	-18 56 10.161	-43 24 45.493	20-29.XI.2018	Equipe Carste	*T. epitychia* **sp. nov.**
13579	Brasil	MG	Conceição do Mato Dentro	CSS-112	-18 56 14.833	-43 24 51.841	20-29.XI.2018	Equipe Carste	*T. epitychia* **sp. nov.**
13580	Brasil	MG	Conceição do Mato Dentro	CSS-113	-18 56 15.896	-43 24 42.978	20-29.XI.2018	Equipe Carste	*T. epitychia* **sp. nov.**
13581	Brasil	MG	Conceição do Mato Dentro	CSS-113	-18 56 15.896	-43 24 42.978	20-29.XI.2018	Equipe Carste	*T. epitychia* **sp. nov.**
13583	Brasil	MG	Conceição do Mato Dentro	CSS-114	-18 56 14.713	-43 24 49.176	20-29.XI.2018	Equipe Carste	*T. epitychia* **sp. nov.**
13585	Brasil	MG	Conceição do Mato Dentro	CSS-118	-18 56 14.713	-43 24 49.176	20-29.XI.2018	Equipe Carste	*T. epitychia* **sp. nov.**
13588	Brasil	MG	Conceição do Mato Dentro	CSS-118	-18 56 14.713	-43 24 49.176	20-29.XI.2018	Equipe Carste	*T. epitychia* **sp. nov.**
13589	Brasil	MG	Conceição do Mato Dentro	CSS-118	-18 56 14.713	-43 24 49.176	20-29.XI.2018	Equipe Carste	*T. epitychia* **sp. nov.**
13590	Brasil	MG	Conceição do Mato Dentro	CSS-118	-18 56 14.713	-43 24 49.176	20-29.XI.2018	Equipe Carste	*T. epitychia* **sp. nov.**
13591	Brasil	MG	Conceição do Mato Dentro	CSS-118	-18 56 14.713	-43 24 49.176	20-29.XI.2018	Equipe Carste	*T. epitychia* **sp. nov.**
13593	Brasil	MG	Conceição do Mato Dentro	CSS-118	-18 56 14.713	-43 24 49.176	20-29.XI.2018	Equipe Carste	*T. epitychia* **sp. nov.**

13594	Brasil	MG	Conceição do Mato Dentro	CSS-118	-18 56 14.713	-43 24 49.176	20-29.XI.2018	Equipe Carste	*T. epitychia* **sp. nov.**
13595	Brasil	MG	Conceição do Mato Dentro	CSS-118	-18 56 14.713	-43 24 49.176	20-29.XI.2018	Equipe Carste	*T. epitychia* **sp. nov.**
13597	Brasil	MG	Conceição do Mato Dentro	CSS-118	-18 56 14.713	-43 24 49.176	20-29.XI.2018	Equipe Carste	*T. epitychia* **sp. nov.**
13598	Brasil	MG	Conceição do Mato Dentro	CSS-118	-18 56 14.713	-43 24 49.176	20-29.XI.2018	Equipe Carste	*T. epitychia* **sp. nov.**
13599	Brasil	MG	Conceição do Mato Dentro	CSS-118	-18 56 14.713	-43 24 49.176	20-29.XI.2018	Equipe Carste	*T. epitychia* **sp. nov.**
13600	Brasil	MG	Conceição do Mato Dentro	CSS-118	-18 56 14.713	-43 24 49.176	20-29.XI.2018	Equipe Carste	*T. epitychia* **sp. nov.**
13601	Brasil	MG	Conceição do Mato Dentro	CSS-118	-18 56 14.713	-43 24 49.176	20-29.XI.2018	Equipe Carste	*T. epitychia* **sp. nov.**
13602	Brasil	MG	Conceição do Mato Dentro	CSS-118	-18 56 14.713	-43 24 49.176	20-29.XI.2018	Equipe Carste	*T. epitychia* **sp. nov.**
13604	Brasil	MG	Conceição do Mato Dentro	CSS-118	-18 56 14.713	-43 24 49.176	20-29.XI.2018	Equipe Carste	*T. epitychia* **sp. nov.**
13607	Brasil	MG	Conceição do Mato Dentro	CSS-118	-18 56 14.713	-43 24 49.176	20-29.XI.2018	Equipe Carste	*T. epitychia* **sp. nov.**
13608	Brasil	MG	Conceição do Mato Dentro	CSS-118	-18 56 14.713	-43 24 49.176	20-29.XI.2018	Equipe Carste	*T. epitychia* **sp. nov.**
13609	Brasil	MG	Conceição do Mato Dentro	CSS-118	-18 56 14.713	-43 24 49.176	20-29.XI.2018	Equipe Carste	*T. epitychia* **sp. nov.**
13618	Brasil	MG	Conceição do Mato Dentro	CAI-0003	-18 53 1.748	-43 25 51.818	25-26.X.2018	Equipe Carste	*T. epitychia* **sp. nov.**
13619	Brasil	MG	Conceição do Mato Dentro	CAI-0003	-18 53 1.748	-43 25 51.818	25-26.X.2018	Equipe Carste	*T. epitychia* **sp. nov.**
13620	Brasil	MG	Conceição do Mato Dentro	CAI-0003	-18 53 1.748	-43 25 51.818	25-26.X.2018	Equipe Carste	*T. epitychia* **sp. nov.**
14041	Brasil	MG	Conceição do Mato Dentro	CAI-0003	-18 53 1.748	-43 25 51.818	22-24.IV.2019	Equipe Carste	*T. epitychia* **sp. nov.**
14042	Brasil	MG	Conceição do Mato Dentro	CAI-0003	-18 53 1.748	-43 25 51.818	22-24.IV.2019	Equipe Carste	*T. epitychia* **sp. nov.**
14043	Brasil	MG	Conceição do Mato Dentro	CAI-0003	-18 53 1.748	-43 25 51.818	22-24.IV.2019	Equipe Carste	*T. epitychia* **sp. nov.**
14045	Brasil	MG	Conceição do Mato Dentro	CAI-0003	-18 53 1.748	-43 25 51.818	22-24.IV.2019	Equipe Carste	*T. epitychia* **sp. nov.**
14050	Brasil	MG	Conceição do Mato Dentro	CSS-0030	-18 55 28.247	-43 25 44.300	16.III.2019	Equipe Carste	*T. epitychia* **sp. nov.**
12492	Brasil	MG	Itambé do Mato Dentro	MLog	-19 20 32.368	-43 18 30.117	26.VI.06.VII.2018	Equipe Carste	*T. epitychia* **sp. nov.**
16394	Brasil	MG	São Gonçalo do Rio Abaixo	MDIR-0028	-19 52 48.690	-43 26 13.599	10-14.II.2020	Equipe Spelayon	*T. epitychia* **sp. nov.**
16395	Brasil	MG	São Gonçalo do Rio Abaixo	MDIR-0028	-19 52 48.690	-43 26 13.599	10-14.II.2020	Equipe Spelayon	*T. epitychia* **sp. nov.**
16396	Brasil	MG	São Gonçalo do Rio Abaixo	MDIR-0028	-19 52 48.690	-43 26 13.599	10-14.II.2020	Equipe Spelayon	*T. epitychia* **sp. nov.**
16444	Brasil	MG	São Gonçalo do Rio Abaixo	MDIR-0028	-19 52 48.690	-43 26 13.599	10-14.II.2020	Equipe Spelayon	*T. epitychia* **sp. nov.**
11801	Brasil	SP	Ribeira	MTD-02	-24 37 27.291	-48 57 35.747	26.VII-06.VIII.2016	Equipe Carste	*T. zampauloi* **sp. nov.**
11830	Brasil	SP	Ribeira	MTD-13	-24 38 47.404	-48 57 52.616	26.VII-06.VIII.2016	Equipe Carste	*T. zampauloi* **sp. nov.**
11851*	Brasil	SP	Ribeira	MTD-13	-24 38 47.404	-48 57 52.616	26.VII-06.VIII.2016	Equipe Carste	*T. zampauloi* **sp. nov.**
11874	Brasil	SP	Ribeira	MTD-02	-24 37 27.291	-48 57 35.747	26.VII-06.VIII.2016	Equipe Carste	*T. zampauloi* **sp. nov.**
11875**	Brasil	SP	Ribeira	MTD-02	-24 37 27.291	-48 57 35.747	08-20.III.2016	Equipe Carste	*T. zampauloi* **sp. nov.**
11876**	Brasil	SP	Ribeira	MTD-02	-24 37 27.291	-48 57 35.747	26.VII-06.VIII.2016	Equipe Carste	*T. zampauloi* **sp. nov.**
11877**	Brasil	SP	Ribeira	MTD-02	-24 37 27.291	-48 57 35.747	08-20.III.2016	Equipe Carste	*T. zampauloi* **sp. nov.**
11878**	Brasil	SP	Ribeira	MTD-02	-24 37 27.291	-48 57 35.747	08-20.III.2016	Equipe Carste	*T. zampauloi* **sp. nov.**

Supplementary material SM2

References for taxonomic authorities

Arlé, R. (1939) Collemboles nouveaux de Rio de Janeiro. *Anais da Academia Brasileira de Ciências*, **11**, 25–32.

Arlé, R. & Guimarães, A.E. (1979) Nova especie do genero *Paronella* Schott, 1893 do Rio de Janeiro (Collembola). *Revista Brasileira de Entomologia*, **23**(4), 213–217.

Bellini, B.C. & Cipola, N.G. (2017) The Neotropical genera of Paronellinae (Collembola, Entomobryoidea, Paronellidae) with description of two new species and redescription of *Campylothorax mitrai*. *Zootaxa*, **4300**(2), 151–179. doi.org/10.11646/zootaxa.4300.2.1

Börner, C. (1906) Das System der Collembolen nebst Beschreibung neuer Collembolen des Hamburger Naturhistorischen Museums. *Mitteilungen aus dem Naturhistorischen Museum in Hamburg*, **23**, 147–188.

Brito, R.A., Lima, E.C.A. & Zeppelini, D. (2019) Three new species of Collembola (Arthropoda: Hexapoda) from Brazil. *Zootaxa*, **4700**, 401–430. doi.org/10.11646/zootaxa.4700.4.1

Gruia, M.M. (1987) Deux nouvelles espècies de Collemboles du Venezuela. Fauna hipógea y hemiedáfica de Venezuela. *Fauna hipógea y hemiedáfica de Venezuela y de otros paises de America del Sur* (ed. by Decu, V., Orghidan, T., Dancau, D., Bordon, C., Linares, O., Urbani, F., Tronchoni, J. & Bosque, C.), pp.151–156. Editura Academiei Republicii Socialiste Rômania.

Handschin, E. (1924) Neue myrmecophile und termitophile Collembolenformen aus Süd-Amerika. *Neue Beiträge zur systematischen Insektenkund*, **3**, 13–28.

Lima, E.C.A., Stievano, L. & Zeppelini, D. (2019) A new species of *Acherontides* (Collembola: Hypogastruridae) from Brazil, with an identification key. *Zootaxa*, **4623**, 141–150. doi.org/10.11646/zootaxa.4623.1.9

Mills, H.B. (1938) Collembola from Yucatan Caves. In: *Fauna of the caves of Yucatan*, 183–190.

Nunes, R.C. & Bellini, B.C. (2018) Three new species of Entomobryoidea (Collembola: Entomobryomorpha) from Brazilian Caatinga-Cerrado transition, with identification keys to Brazilian *Cyphoderus*, *Pseudosinella* and *Trogolaphysa* species. *Zootaxa* **4420**, 71. doi.org/10.11646/zootaxa.4420.1.4

Ojeda, M. & Palacios-Vargas, J.G. (1984) A new species of *Troglopedetes* (Collembola: Paronellidae) from Guerrero, Mexico. *Entomological news*, **95**, 16–20.

Oliveira, J.V.L.C., Alves, J.L.S. & Zeppelini, D. (2017) Two new *Cyphoderus* (Collembola: Paronellidae) of “tridenticulati” and “bidenticulate” groups from Brazilian Amazon. *Zootaxa*, **4350**(1), 47–60. doi.org/10.11646/zootaxa.4350.1.2

Palacios-Vargas, J., Ojeda, M. & Christiansen, K. (1985) Taxonomía y biogeografía de *Troglopedetes* (Collembola: Paronellidae) en América, con énfasis en las especies cavernícolas. *Folia entomologica mexicana*, 3–35.

Palacios-Vargas, J.G. & Gnaspini-Netto, P. (1992) A new Brazilian species of *Acherontides* (Collembola: Hypogastruridae) with notes on its ecology. *Journal of The Kansas Entomological Society*, **65**, 443–447.

Palacios-Vargas, J.G. & Zeppelini, D. (1995a) Seven new *Arrhopalites* (Hexapoda: Collembola) from Brazilian and Mexican caves. *Folia Entomológica Mexicana*, **93**, 21–37.

Palacios-Vargas, J.G. & Zeppelini, D. (1995b) A new species of *Troglobius* (Collembola, Paronellidae) from Brazil. *International Journal of Speleology*, **23**(3–4), 173–177.

Palacios-Vargas, J.G. & Thibaud, J.M. (1997) New cave Collembola from Mexico and Belize. *Southwestern Entomologist*, **22**, 323–329.

Silva, D.D. & Bellini, B.C. (2015) *Trogolaphysa formosensis* **sp. nov.** (Collembola: Paronellidae) from Atlantic Forest, Northeast Region of Brazil. *Zoologia*, **32**(1), 53–58. doi.org/10.1590/S1984-46702015000100008

Soto-Adames, F.N. & Taylor, S.J. (2013) The dorsal chaetotaxy of *Trogolaphysa* (Collembola, Paronellidae), with descriptions of two new species from caves in Belize. *ZooKeys*, **323**, 35–74. doi.org/10.3897/zookeys.323.4950

Soto-Adames, F.N. (2015) The dorsal chaetotaxy of first instar *Trogolaphysa jataca*, with description of twelve new species of Neotropical *Trogolaphysa* (Hexapoda: Collembola: Paronellidae). *Zootaxa*, **4032**, 1–41. doi.org/10.11646/zootaxa.4032.1.1

Thibaud, J. & Najt, J. (1988) Collemboles (Insecta) de l'Équateur IV. Paronellidae avec rérision de quatre genres. *Bulletin du Muséum national d'histoire naturelle*, **10**, 719–730.

Yoshii, R. (1988) Paronellid Collembola from caves of Central and South America collected by P. Strinati. *Revue Suisse de Zoologie*, **95**, 449–459. doi.org/10.5962/bhl.part.82022

Zeppelini, D. & Palacios-Vargas, J.G. (1999) A new troglomorphic species of *Arrhopalites* (Collembola: Arrhopalitidae) from Brazil. *Journal of the New York Entomological Society*, **107**(1), 78–81.

Zeppelini, D., Silva, D. D. & Palacios-Vargas, J.G (2014). A new species of *Troglobius* (Collembola, Paronellidae, Cyphoderinae) from a Brazilian iron cave. *Subterranean Biology*, **14**, 1–13. doi.org/10.3897/subtbiol.14.7355

Zeppelini D. & Oliveira, J.V.L.C. (2016) Chaetotaxy of Neotropical *Cyphoderus caetetus* **sp. nov.** with comments on the taxonomic position of Cyphoderinae within Paronellidae (Collembola, Entomobryoidea). *Zootaxa*, **4098**(3), 560–570. doi.org/10.11646/zootaxa.4098.3.8
