# Supplementary material for: Hotspot in ferruginous rock may have serious implications in Brazilian conservation policy
Source: Sci Rep. 2022 Sep 1;12:14871. doi: 10.1038/s41598-022-18798-1 (PMC9437091; doi:10.1038/s41598-022-18798-1)
Supplement: Supplementary file 2 — Supplementary Information 2. [file 41598_2022_18798_MOESM2_ESM.docx]

**S1 -** Additional Records for the new species of *Trogolaphysa* deposited in the *Coleção de Referência de Fauna de Solo* (CRFS-UEPB).

**S2 –** References of Taxonomic authorities.
